# Supplementary figures and images for: GABARAP proteins regulate the packaging of HIV-1 genomic RNA into virions (part 1 of 2)
Source: EMBO Rep. 2025 Oct 31;26(23):5826–58. doi: 10.1038/s44319-025-00607-1 (PMC12678799; doi:10.1038/s44319-025-00607-1)

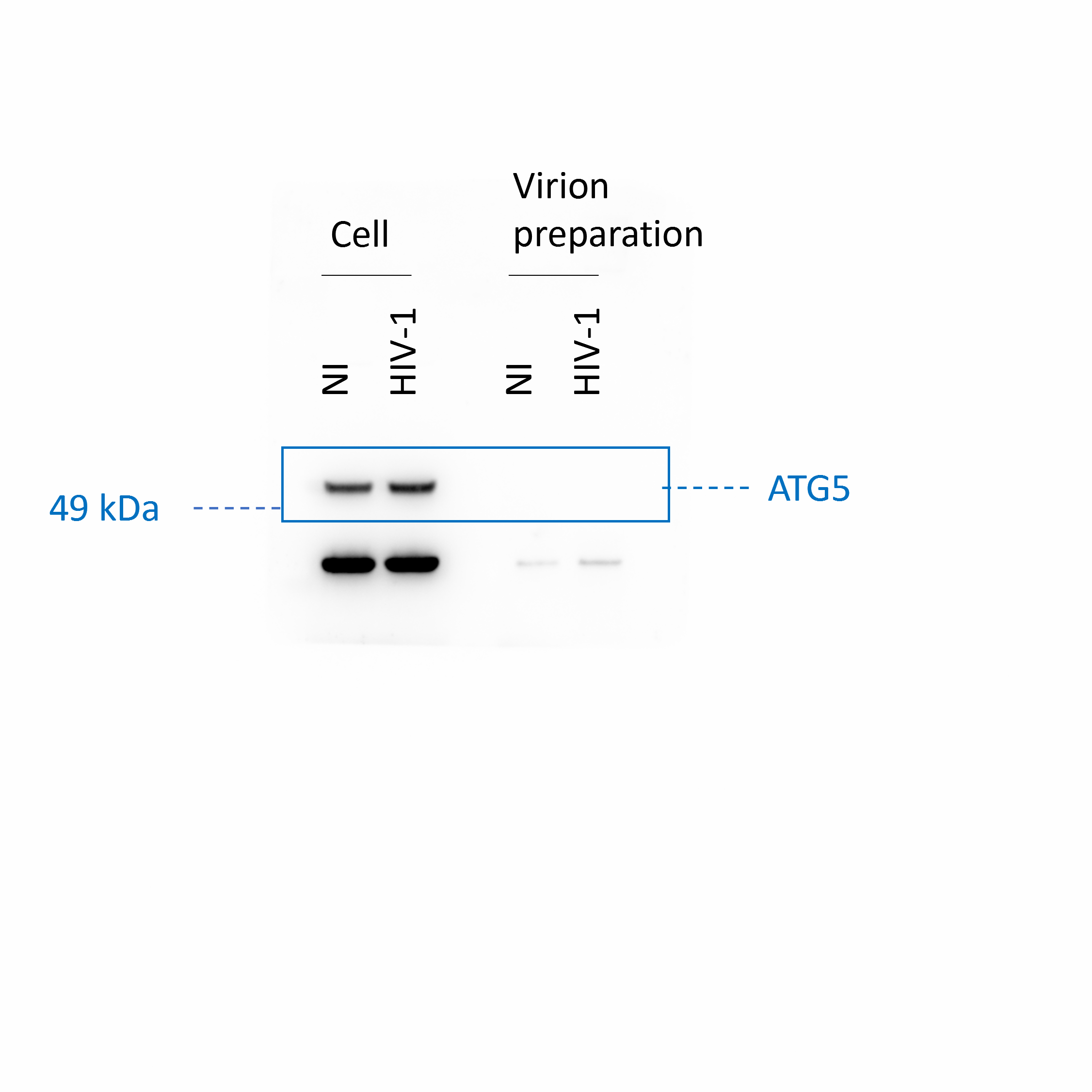

Supplement: Supplementary file 4 — Source data Fig. 2 [file 44319_2025_607_MOESM4_ESM.zip › Figure 2C/fig2C_ATG5.tif]

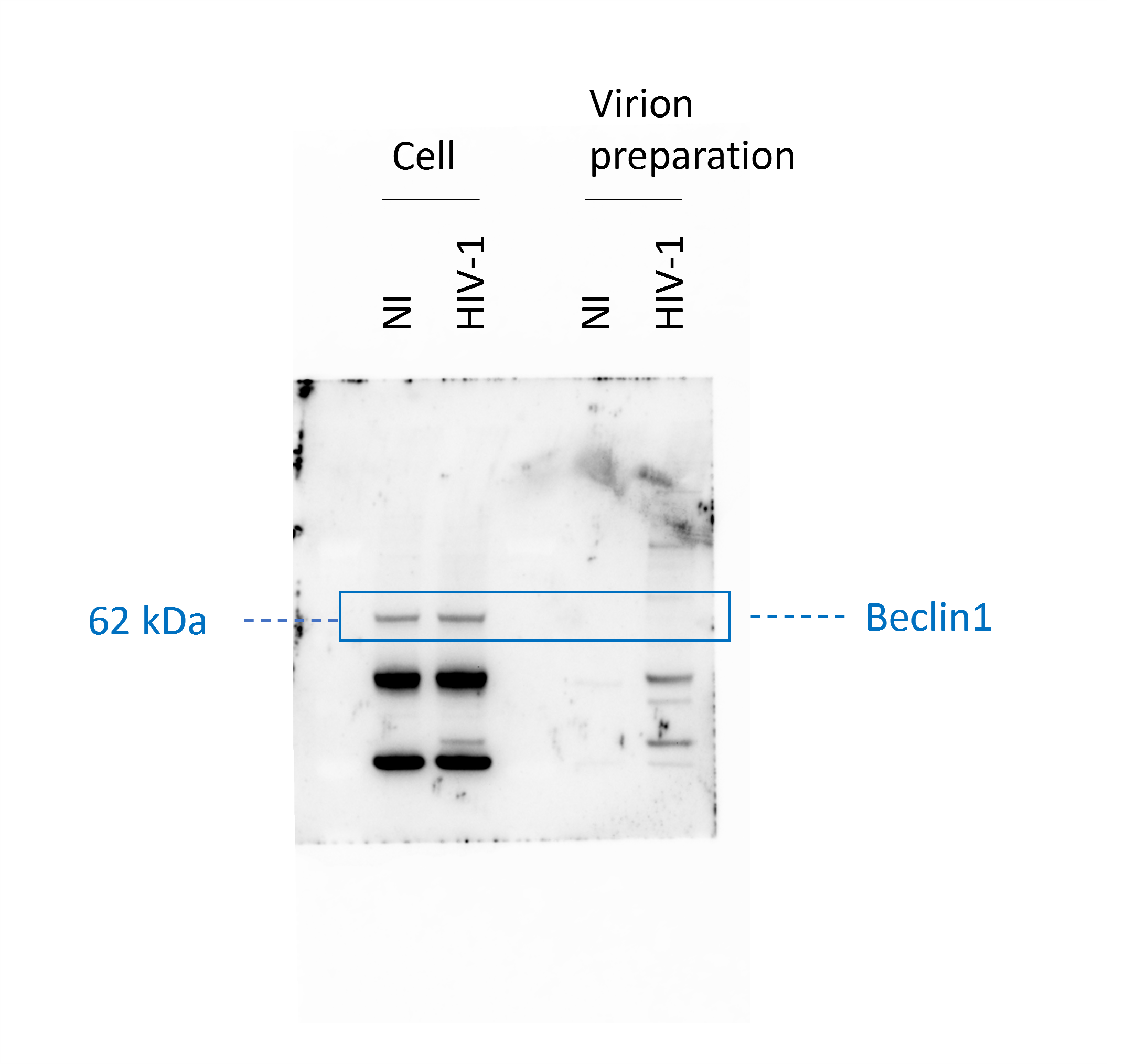

Supplement: Supplementary file 4 — Source data Fig. 2 [file 44319_2025_607_MOESM4_ESM.zip › Figure 2C/fig2C_Beclin1.tif]

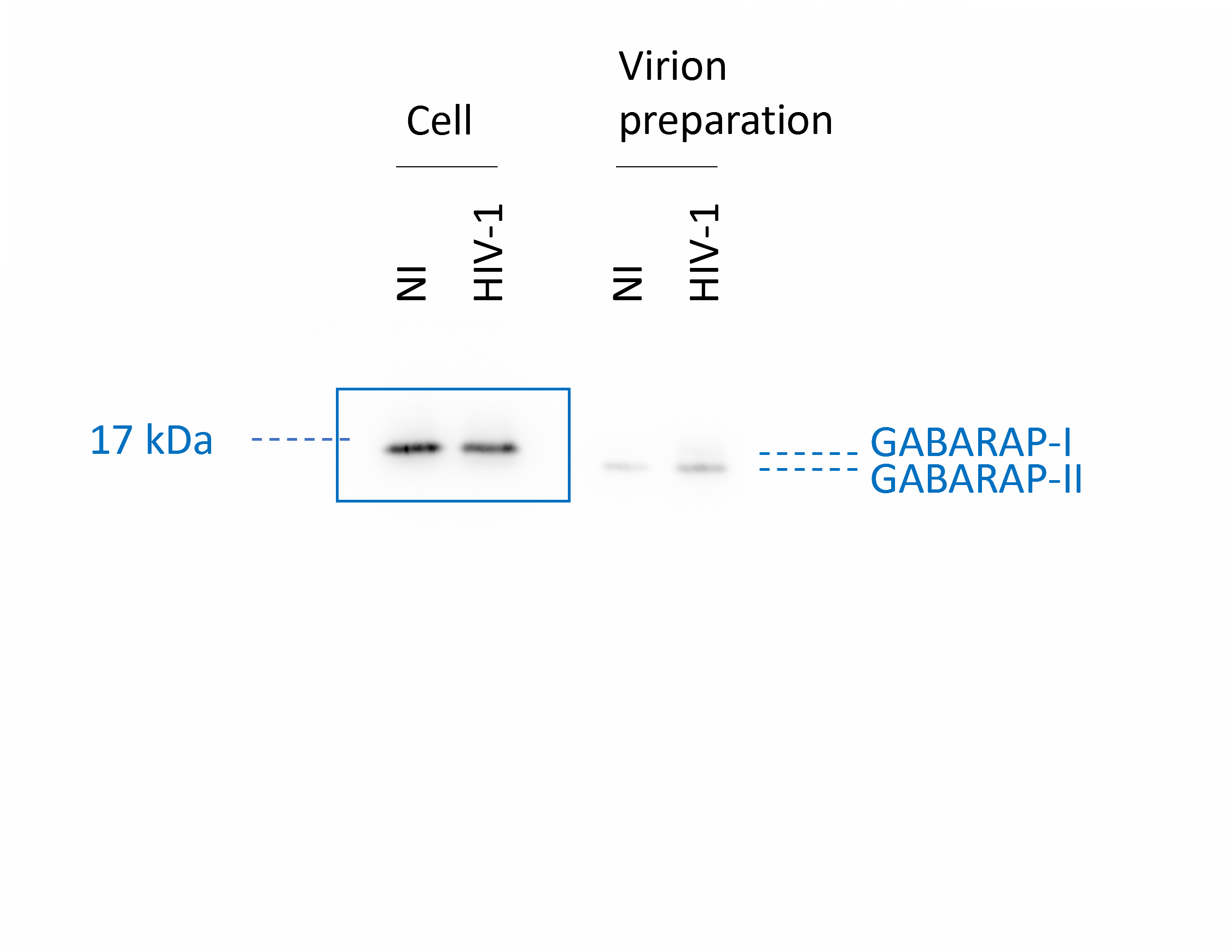

Supplement: Supplementary file 4 — Source data Fig. 2 [file 44319_2025_607_MOESM4_ESM.zip › Figure 2C/fig2C_GABARAP_cell.tif]

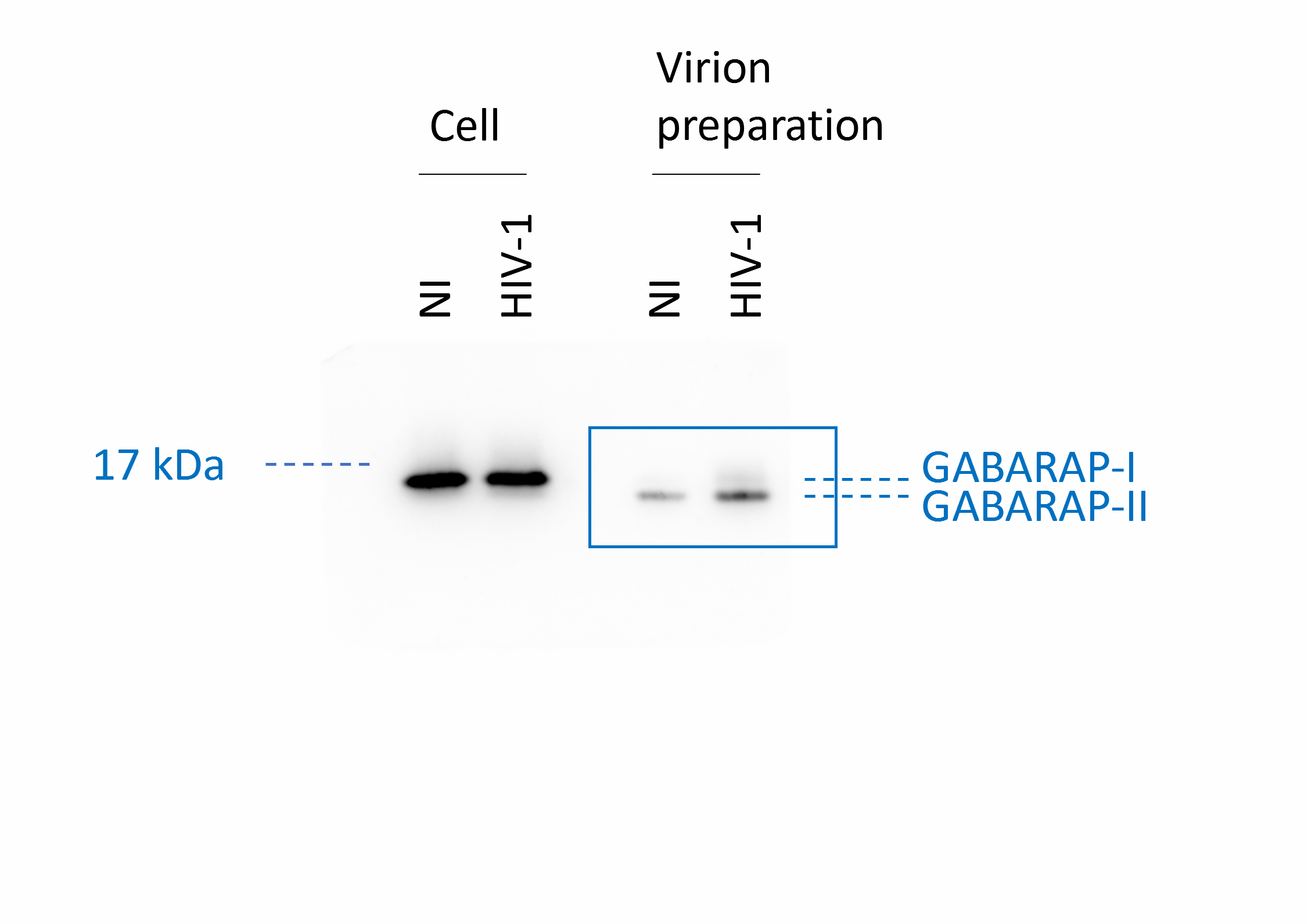

Supplement: Supplementary file 4 — Source data Fig. 2 [file 44319_2025_607_MOESM4_ESM.zip › Figure 2C/fig2C_GABARAP_virion prep.tif]

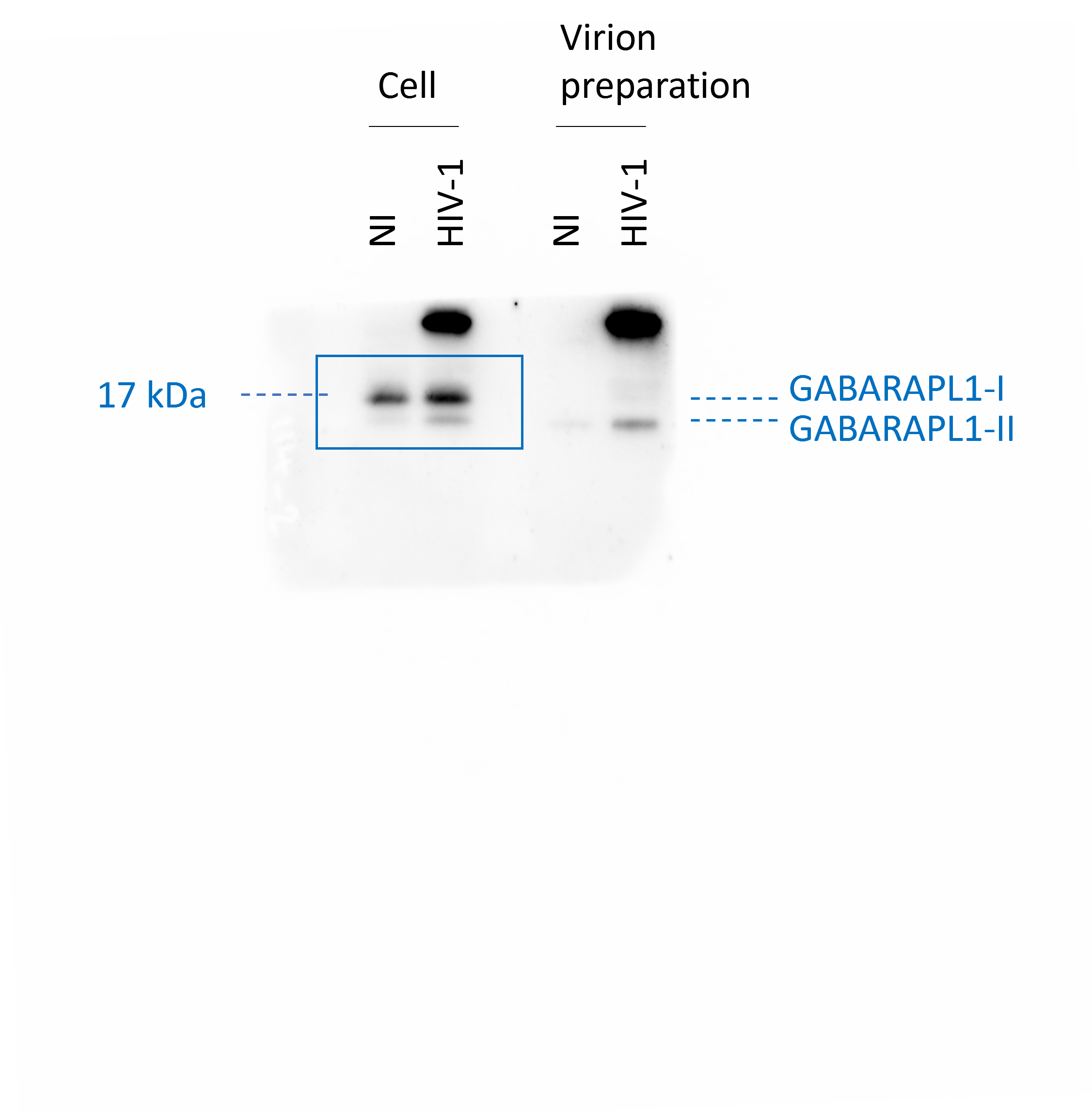

Supplement: Supplementary file 4 — Source data Fig. 2 [file 44319_2025_607_MOESM4_ESM.zip › Figure 2C/fig2C_GABARAPL1_cell.tif]

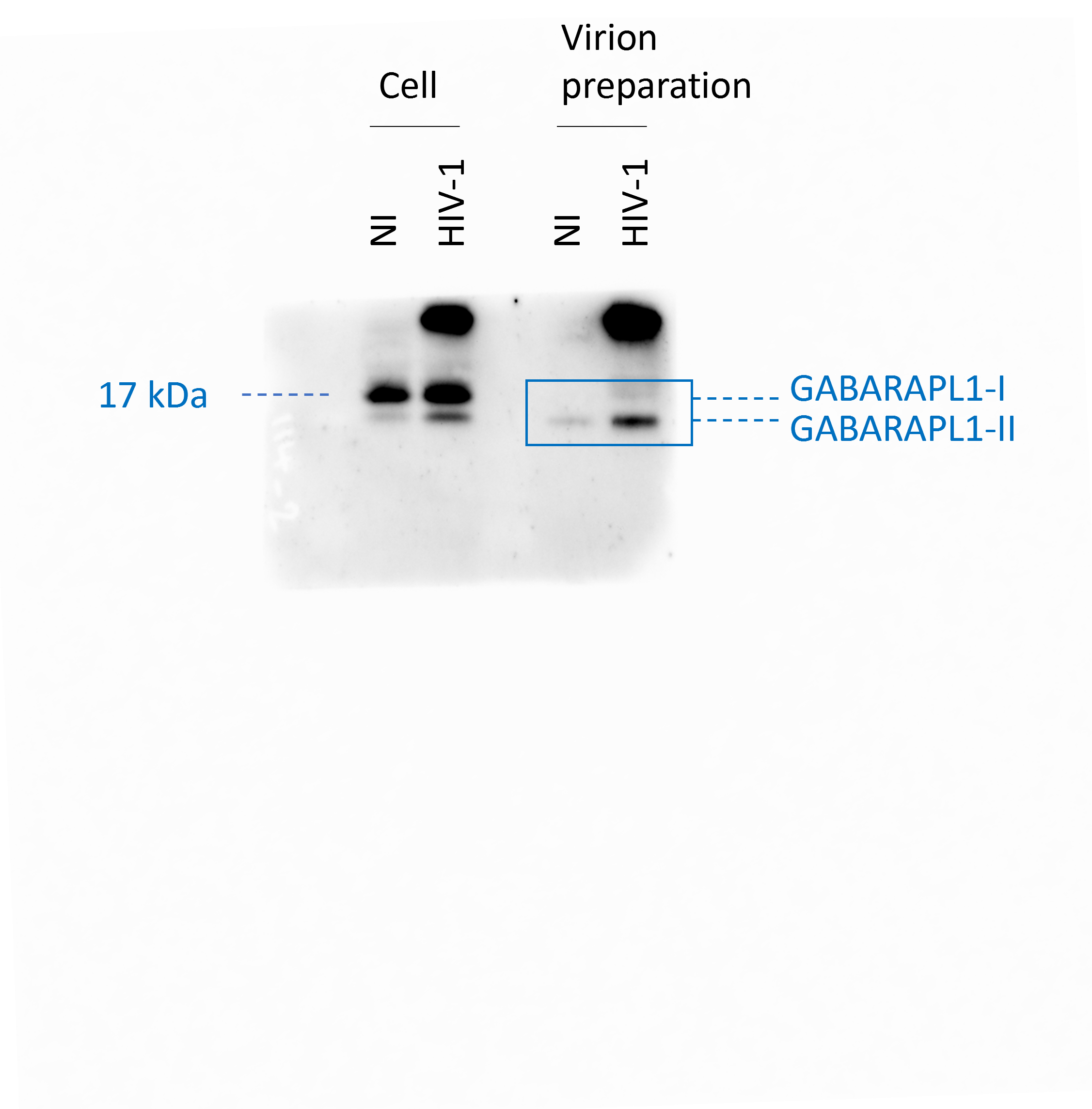

Supplement: Supplementary file 4 — Source data Fig. 2 [file 44319_2025_607_MOESM4_ESM.zip › Figure 2C/fig2C_GABARAPL1_virion prep.tif]

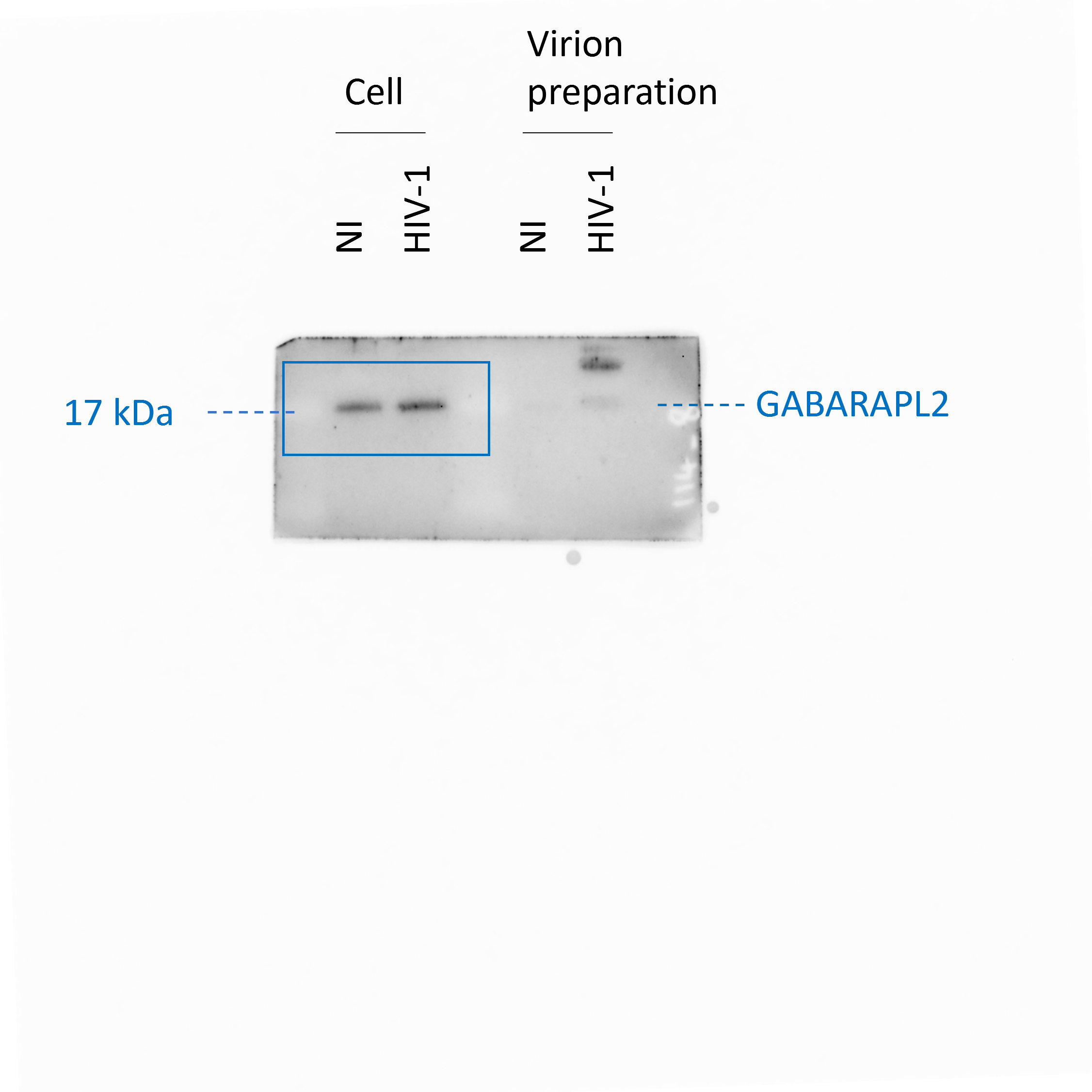

Supplement: Supplementary file 4 — Source data Fig. 2 [file 44319_2025_607_MOESM4_ESM.zip › Figure 2C/fig2C_GABARAPL2_cell.tif]

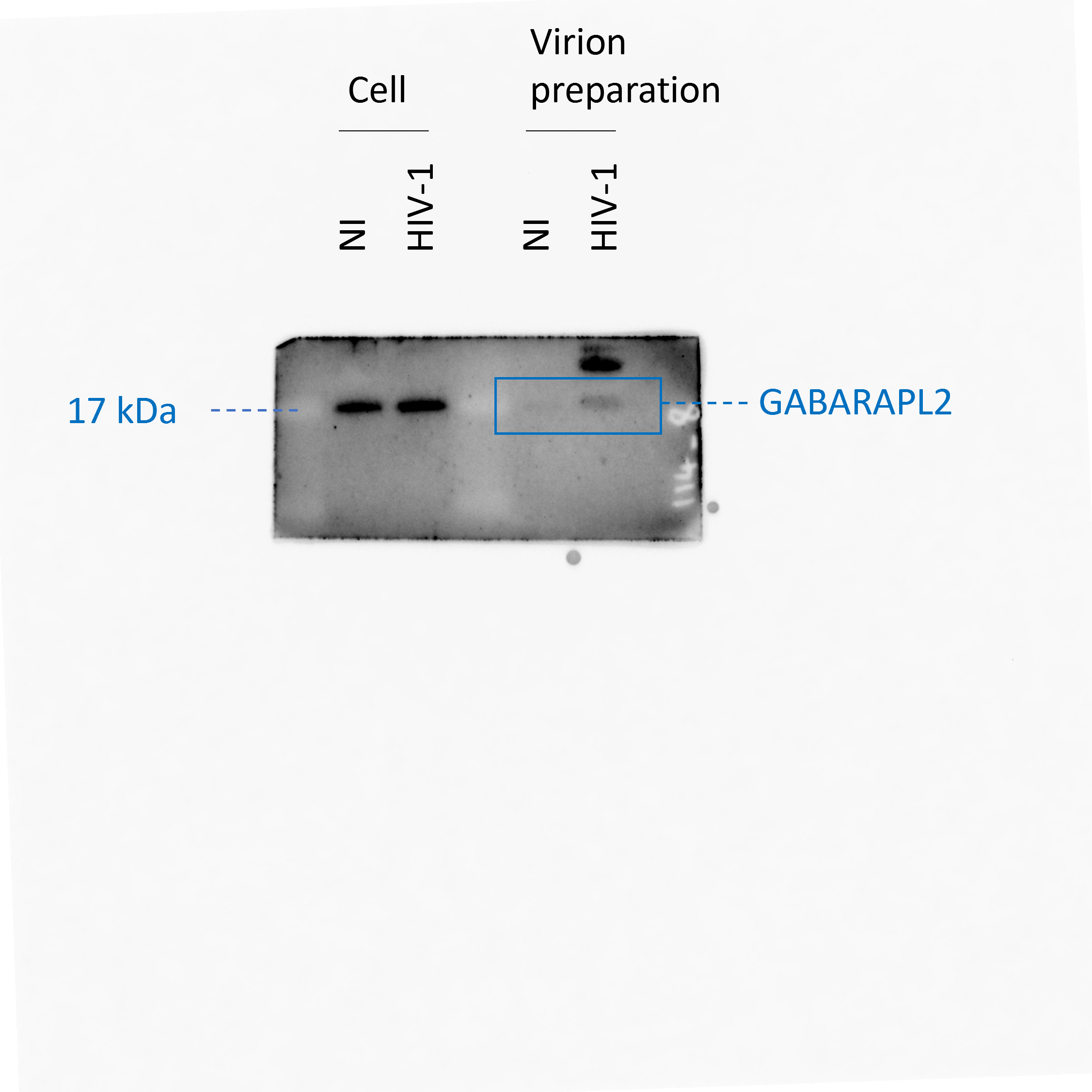

Supplement: Supplementary file 4 — Source data Fig. 2 [file 44319_2025_607_MOESM4_ESM.zip › Figure 2C/fig2C_GABARAPL2_virion prep.tif]

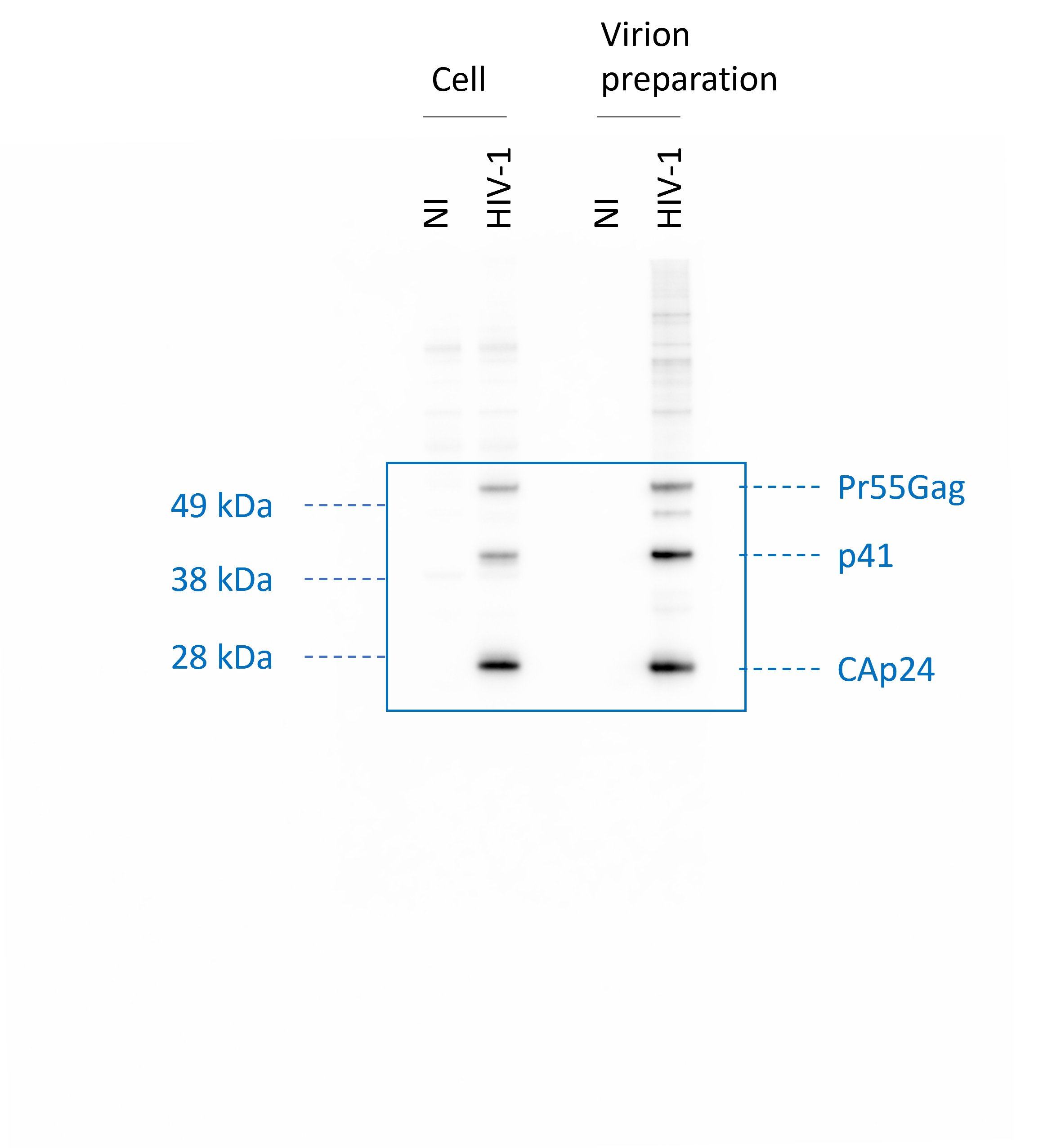

Supplement: Supplementary file 4 — Source data Fig. 2 [file 44319_2025_607_MOESM4_ESM.zip › Figure 2C/fig2C_Gag.tif]

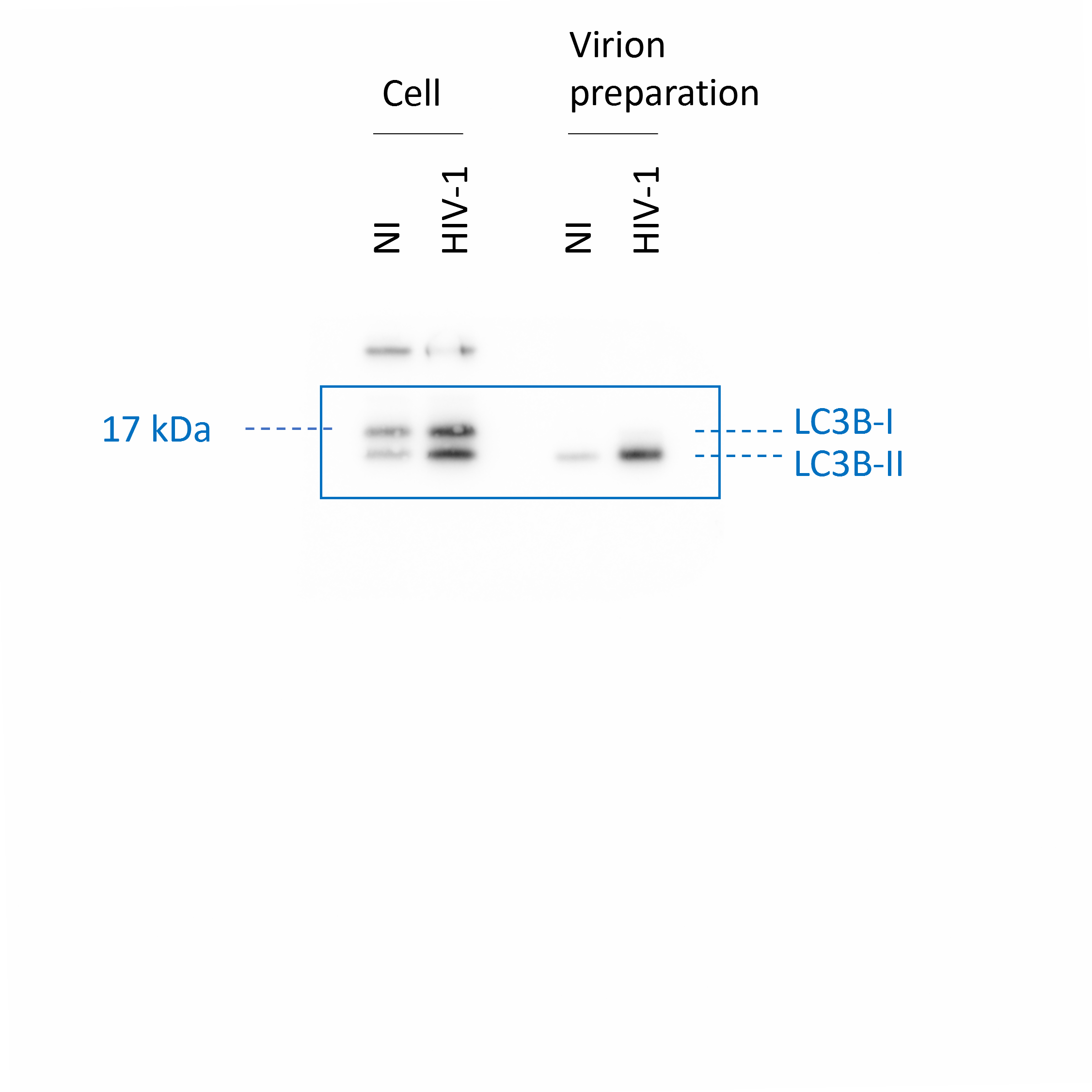

Supplement: Supplementary file 4 — Source data Fig. 2 [file 44319_2025_607_MOESM4_ESM.zip › Figure 2C/fig2C_LC3B.tif]

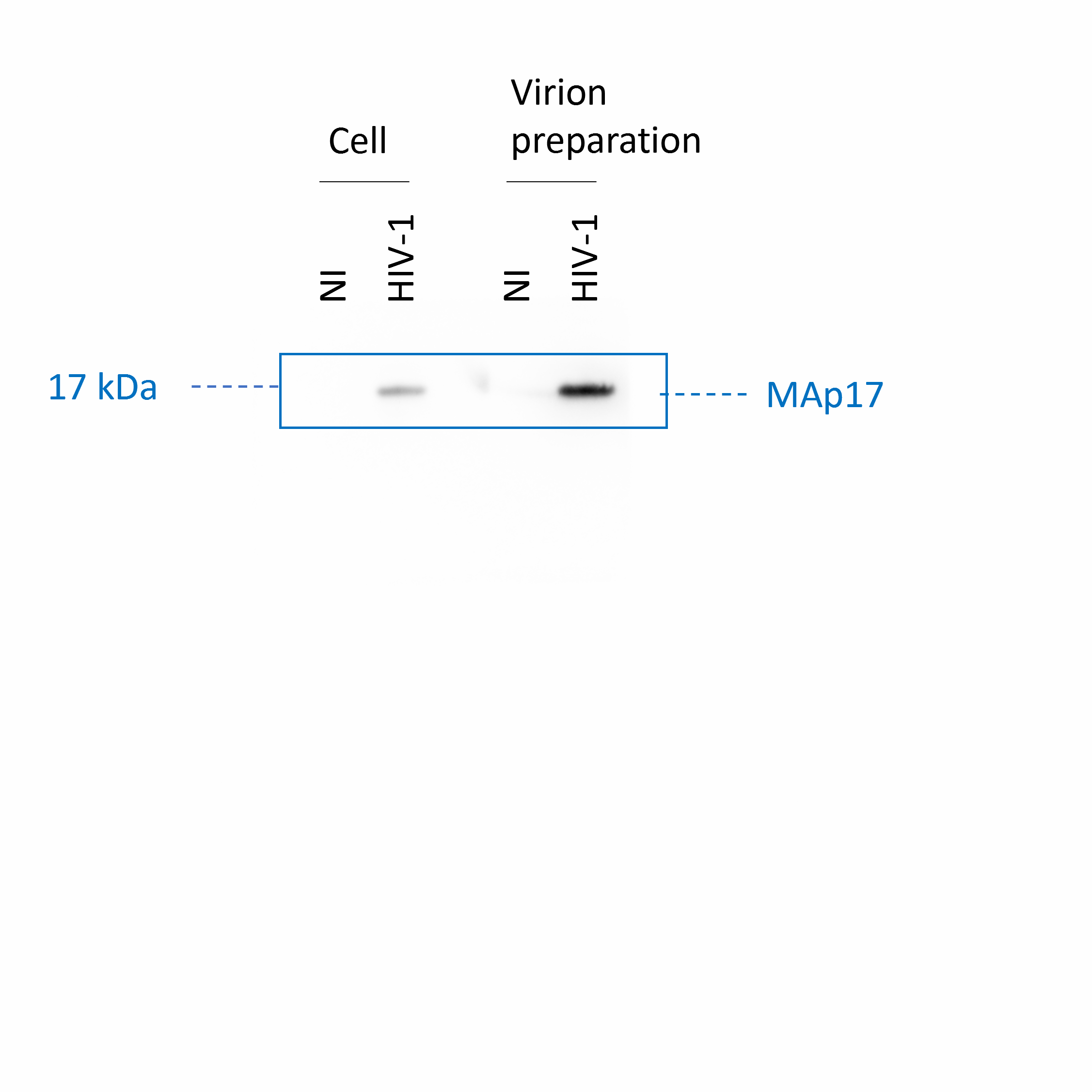

Supplement: Supplementary file 4 — Source data Fig. 2 [file 44319_2025_607_MOESM4_ESM.zip › Figure 2C/fig2C_MAp17.tif]

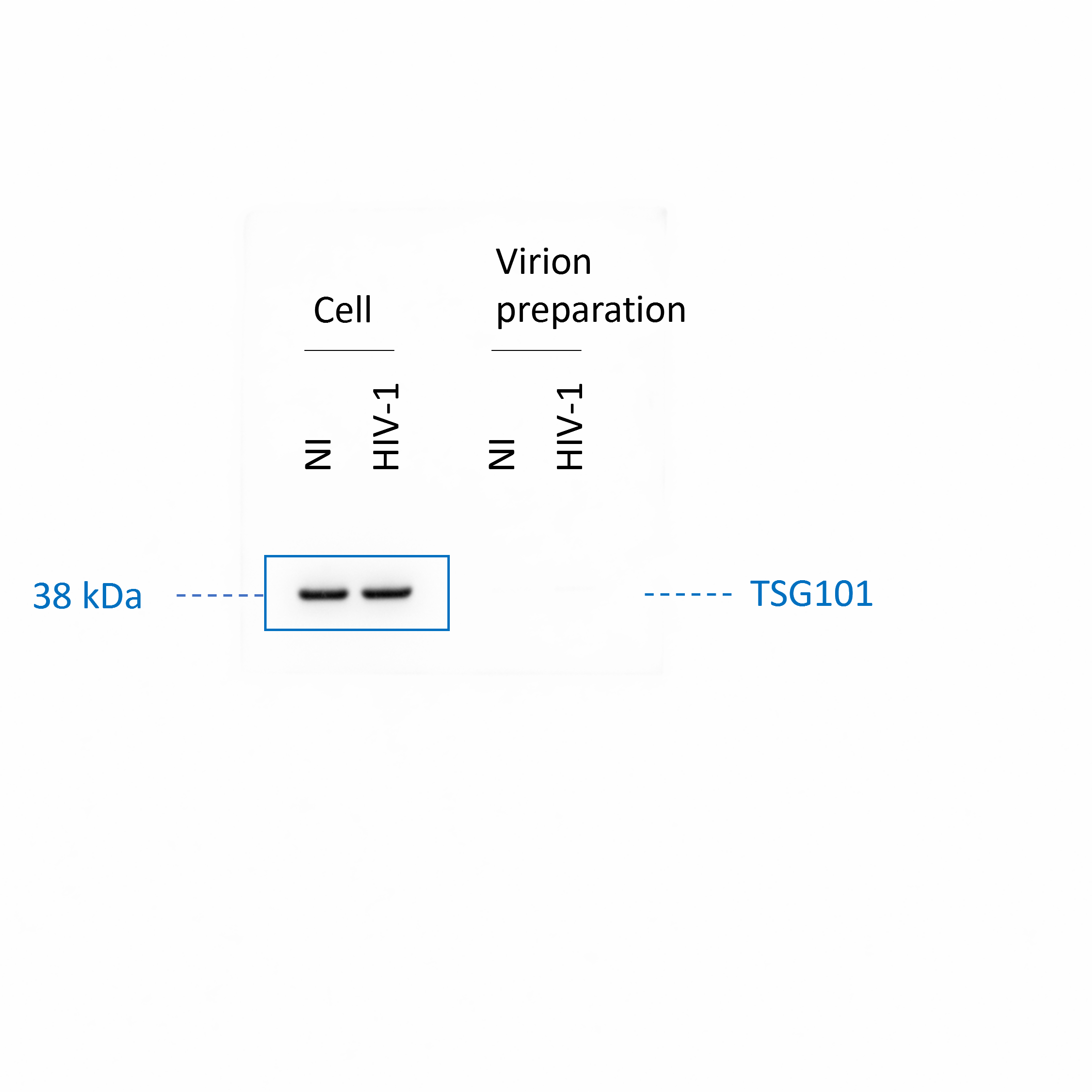

Supplement: Supplementary file 4 — Source data Fig. 2 [file 44319_2025_607_MOESM4_ESM.zip › Figure 2C/fig2C_TSG101_cell.tif]

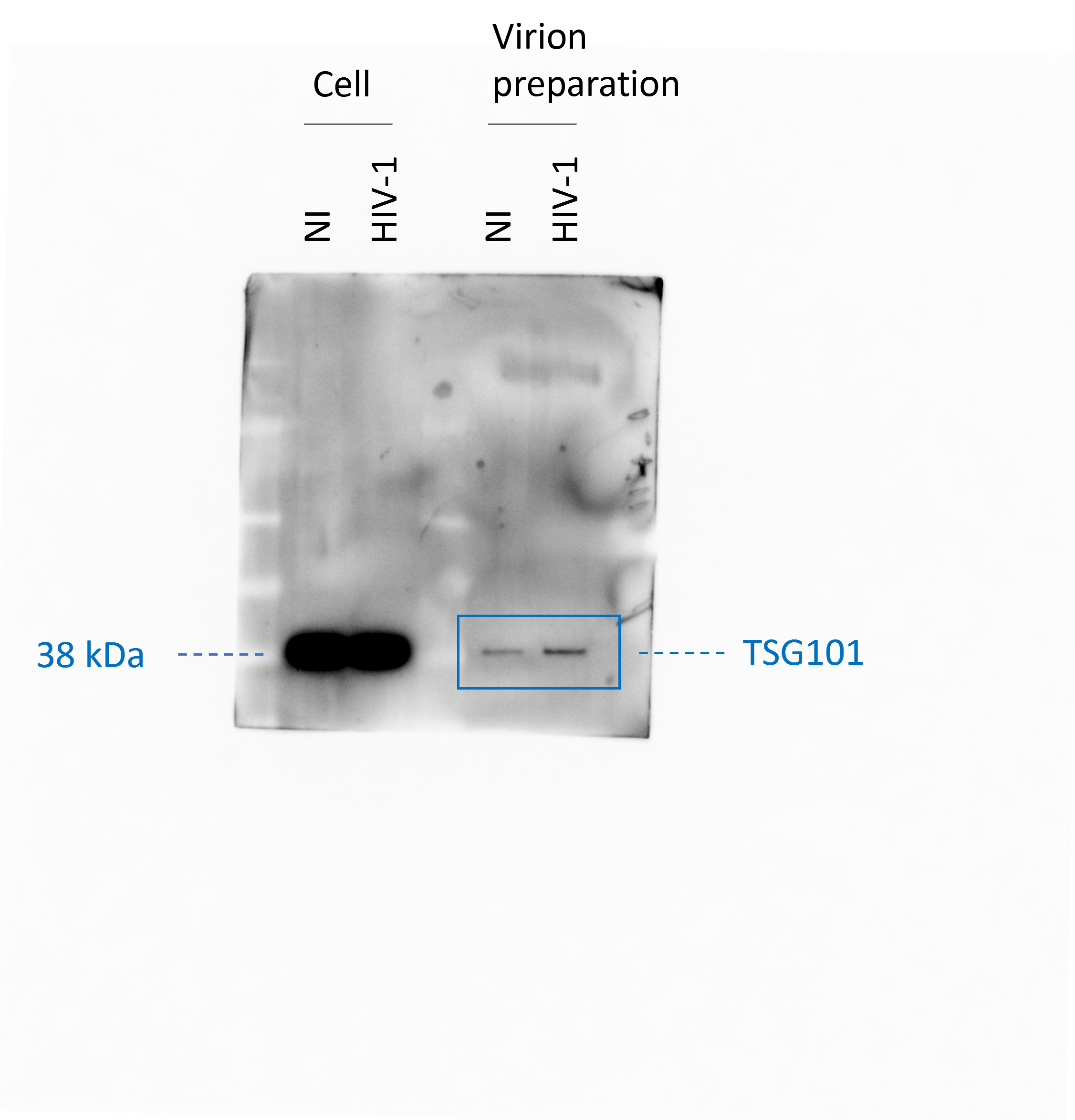

Supplement: Supplementary file 4 — Source data Fig. 2 [file 44319_2025_607_MOESM4_ESM.zip › Figure 2C/fig2C_TSG101_virion prep.tif]

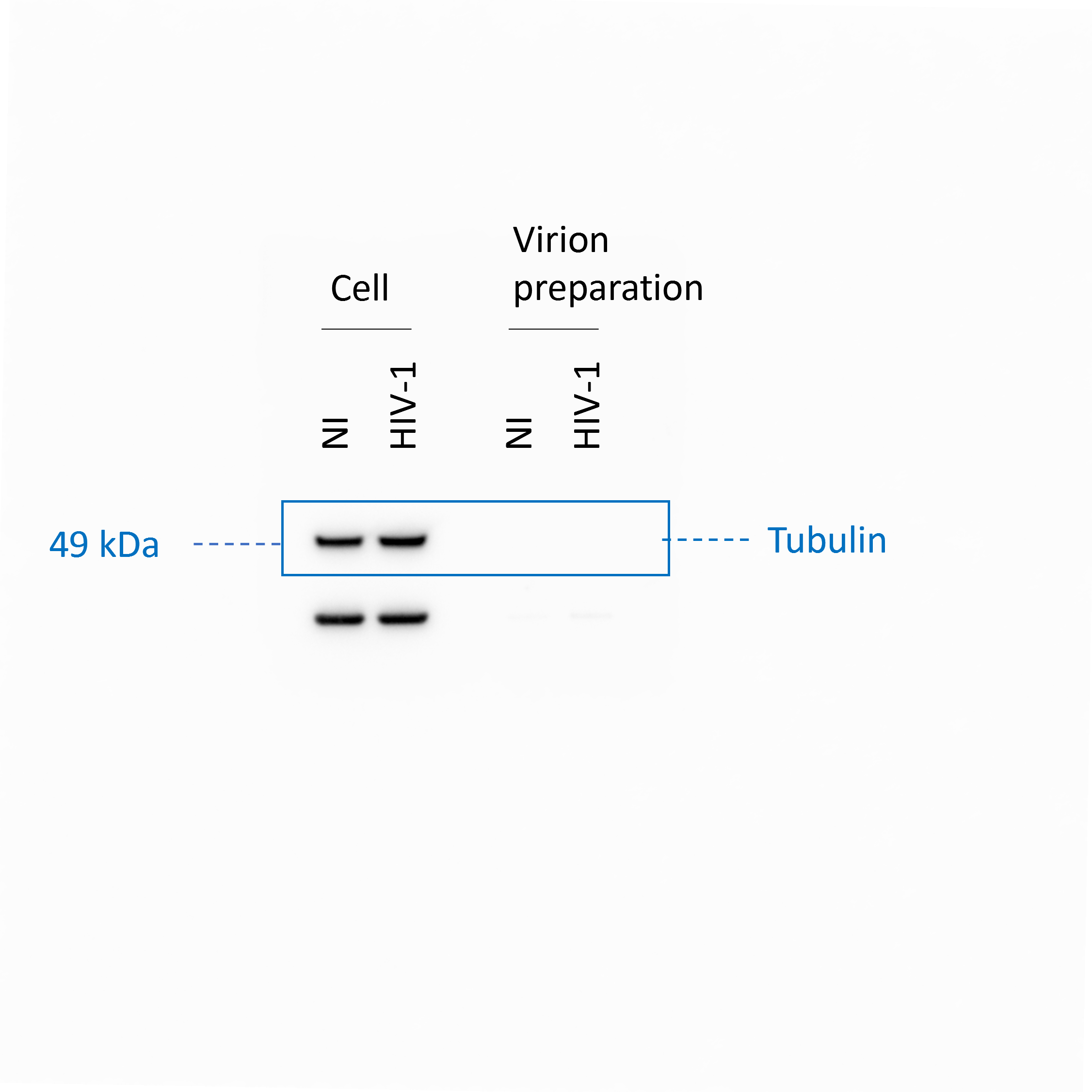

Supplement: Supplementary file 4 — Source data Fig. 2 [file 44319_2025_607_MOESM4_ESM.zip › Figure 2C/fig2C_Tubulin.tif]

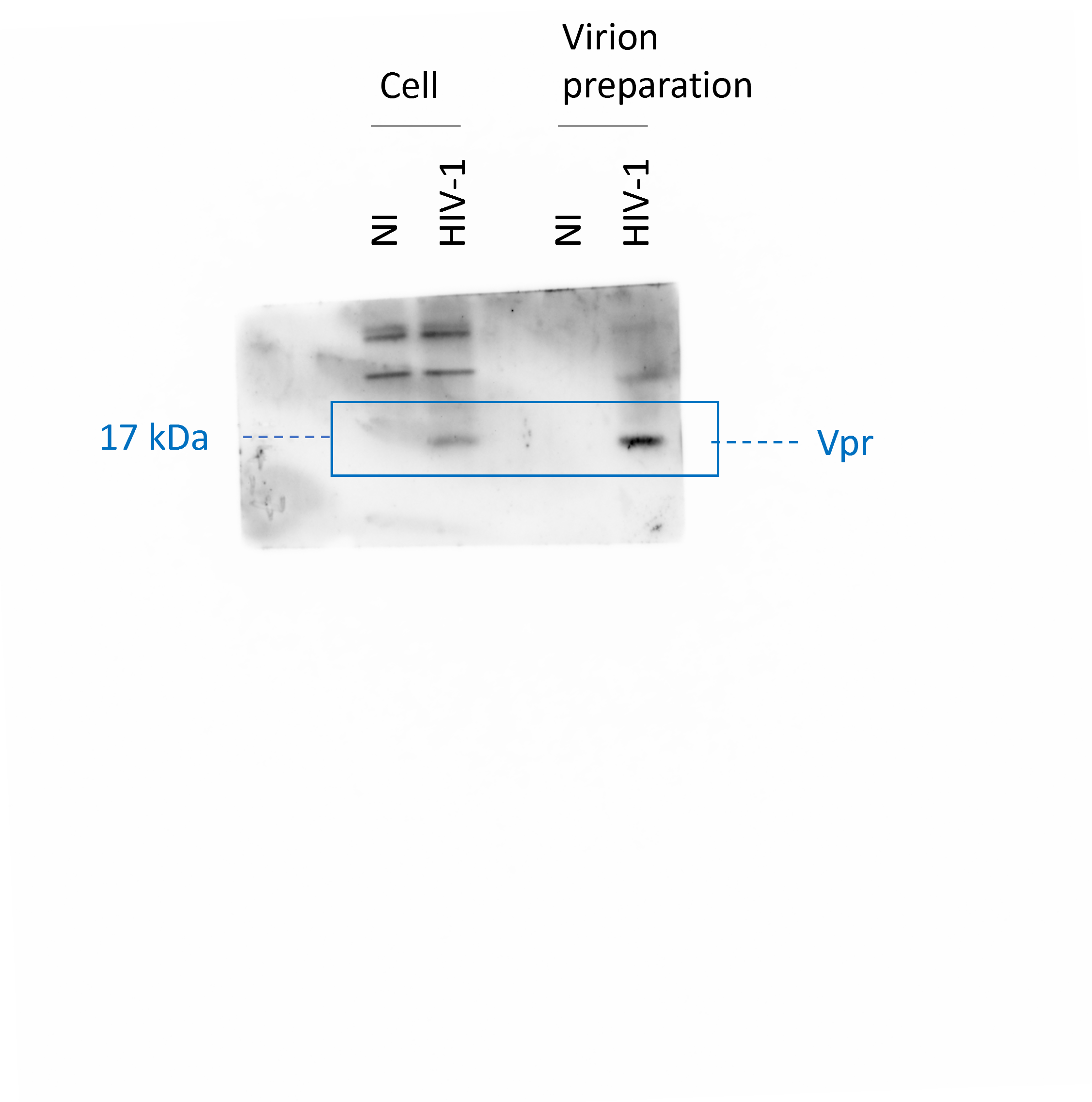

Supplement: Supplementary file 4 — Source data Fig. 2 [file 44319_2025_607_MOESM4_ESM.zip › Figure 2C/fig2C_Vpr.tif]

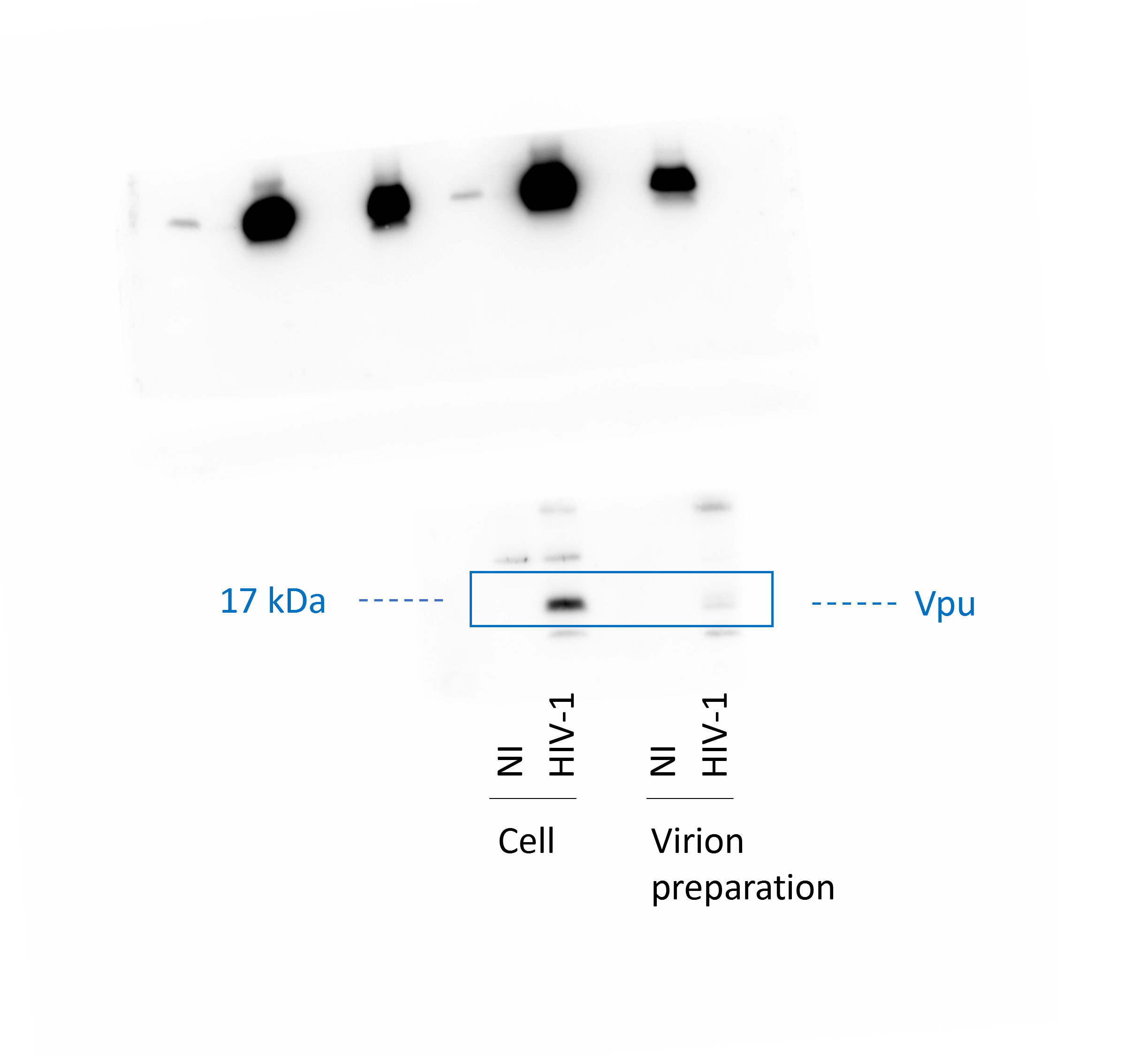

Supplement: Supplementary file 4 — Source data Fig. 2 [file 44319_2025_607_MOESM4_ESM.zip › Figure 2C/fig2C_Vpu.tif]

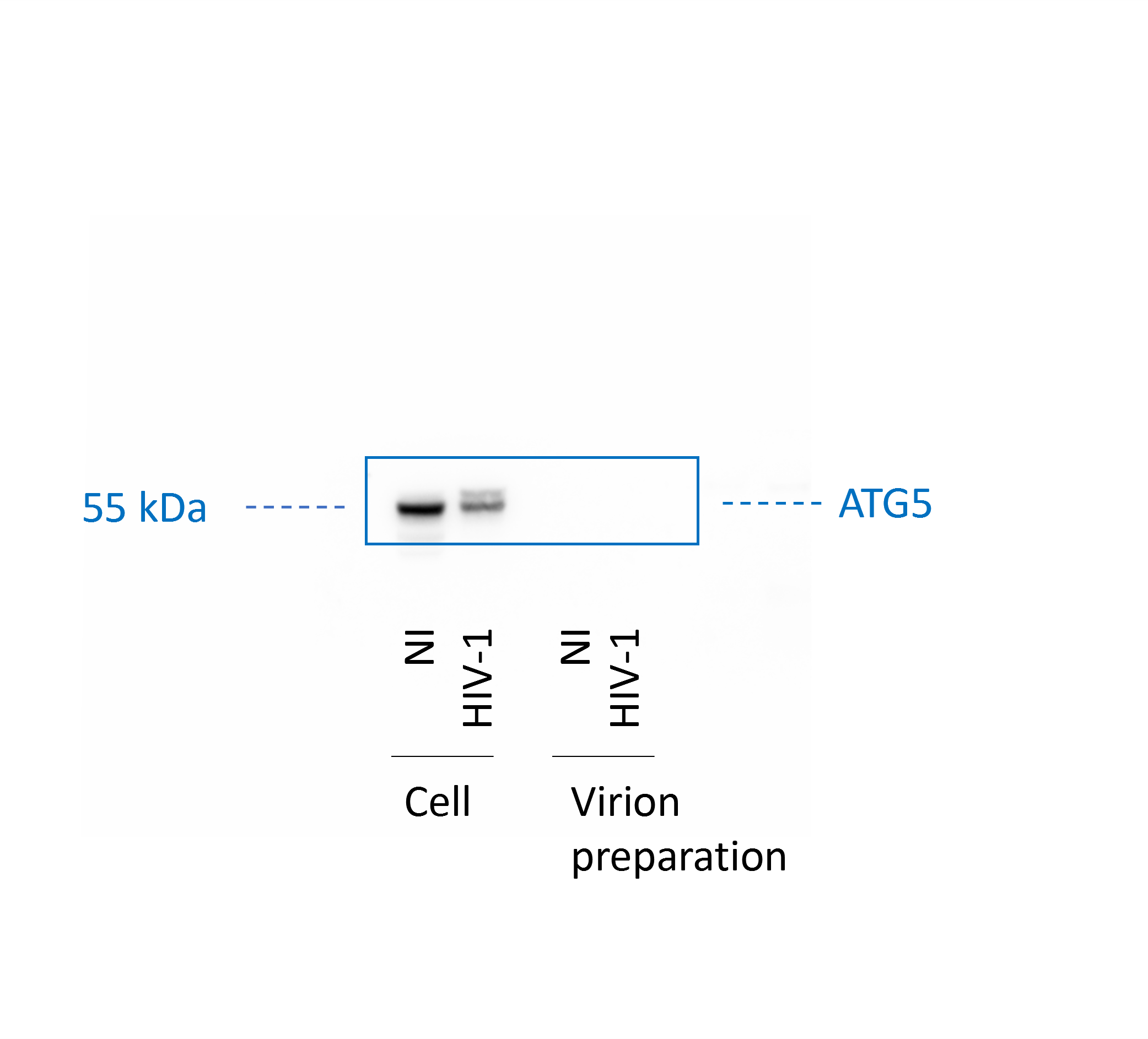

Supplement: Supplementary file 4 — Source data Fig. 2 [file 44319_2025_607_MOESM4_ESM.zip › Figure 2D/fig2D_ATG5.tif]

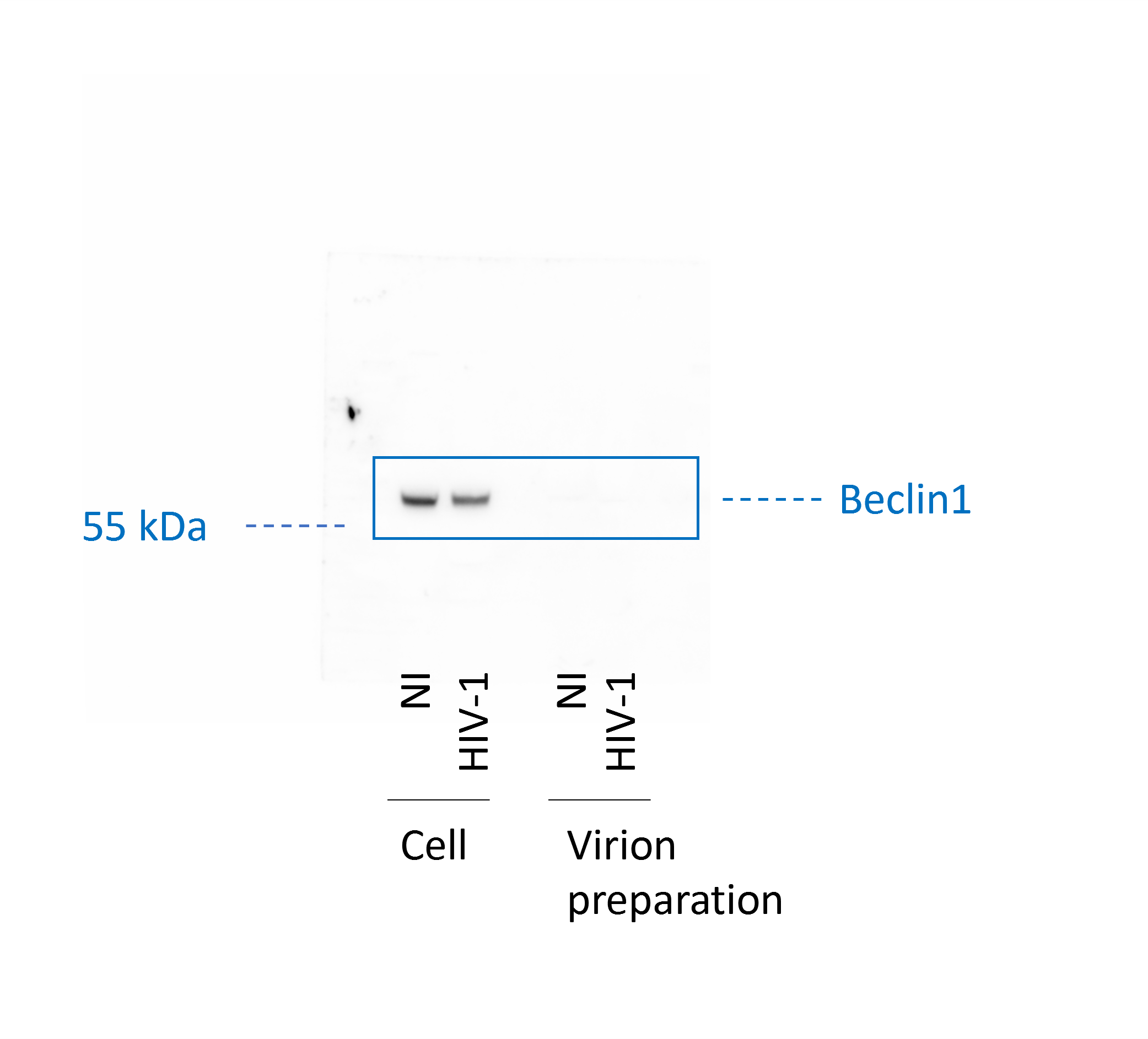

Supplement: Supplementary file 4 — Source data Fig. 2 [file 44319_2025_607_MOESM4_ESM.zip › Figure 2D/fig2D_Beclin1.tif]

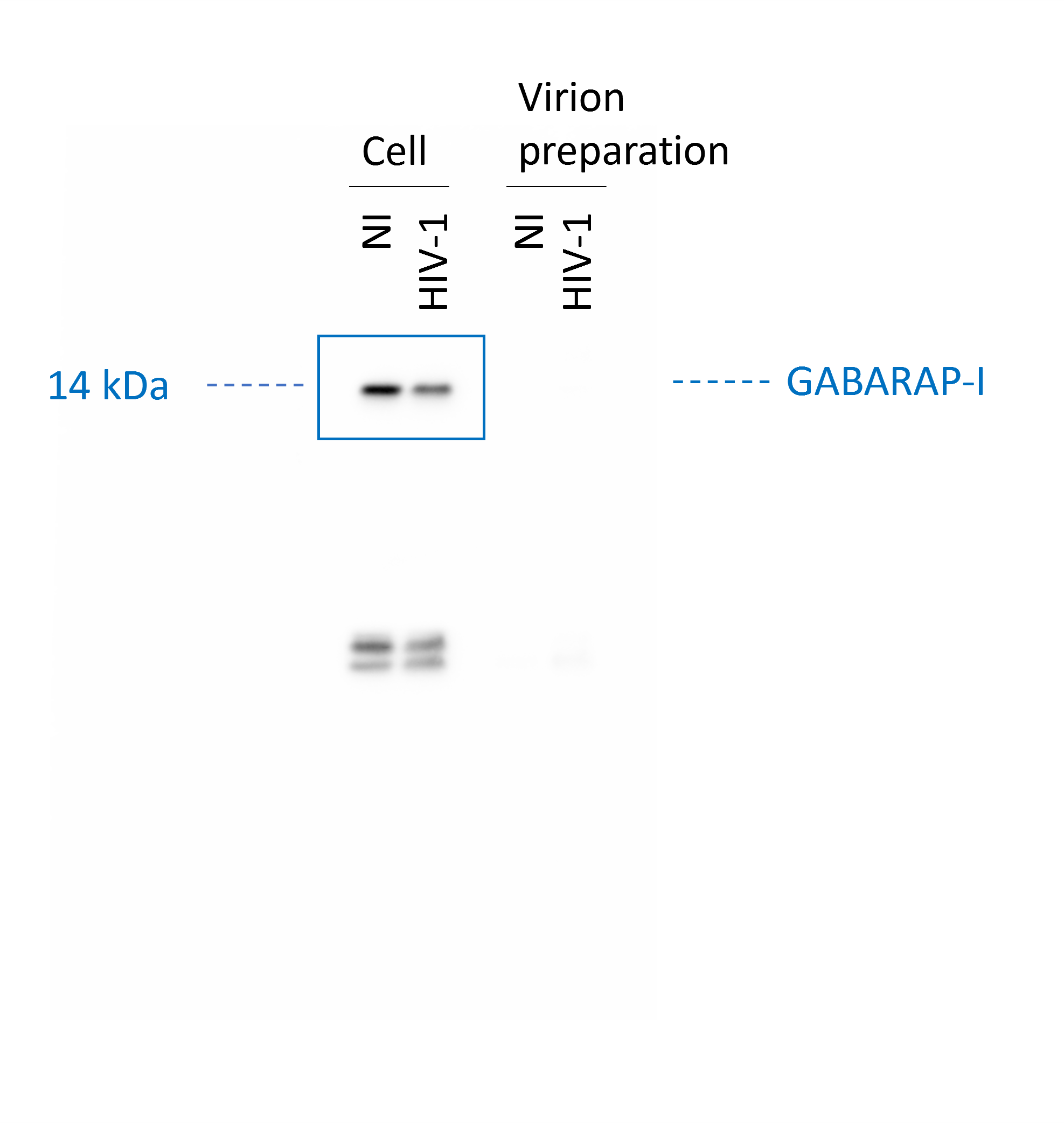

Supplement: Supplementary file 4 — Source data Fig. 2 [file 44319_2025_607_MOESM4_ESM.zip › Figure 2D/fig2D_GABARAP_cell.tif]

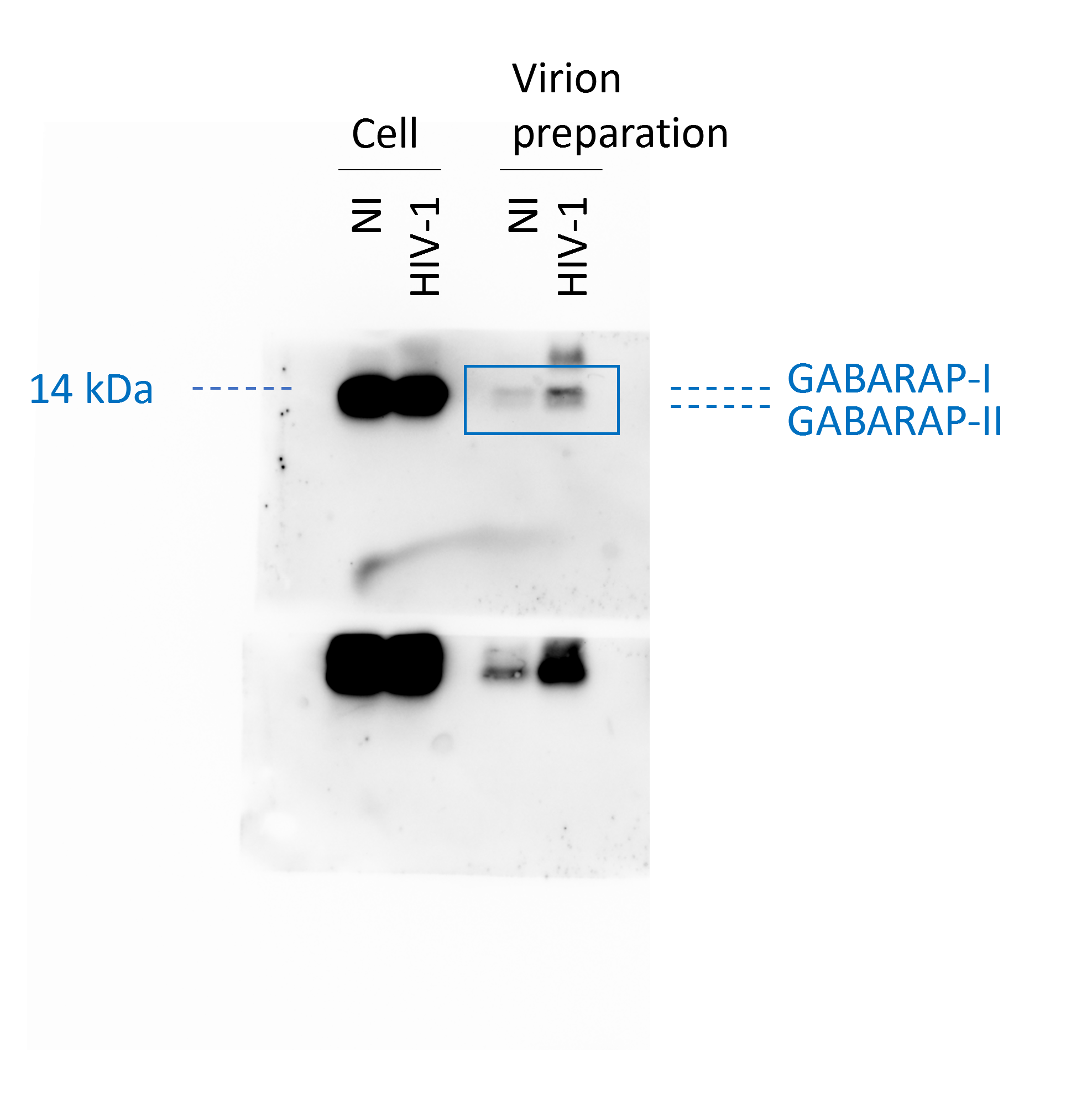

Supplement: Supplementary file 4 — Source data Fig. 2 [file 44319_2025_607_MOESM4_ESM.zip › Figure 2D/fig2D_GABARAP_virion prep.tif]

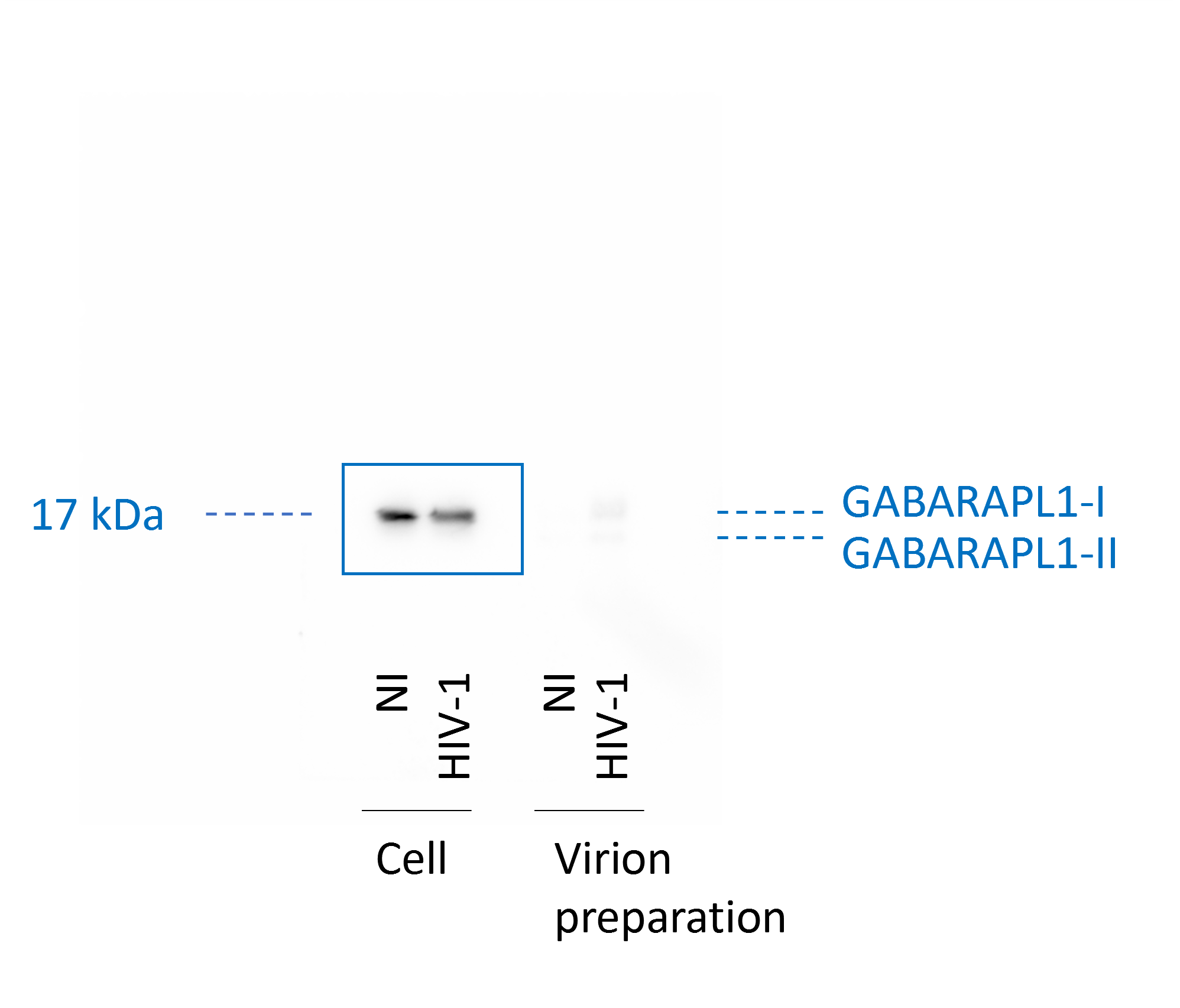

Supplement: Supplementary file 4 — Source data Fig. 2 [file 44319_2025_607_MOESM4_ESM.zip › Figure 2D/fig2D_GABARAPL1_cell.tif]

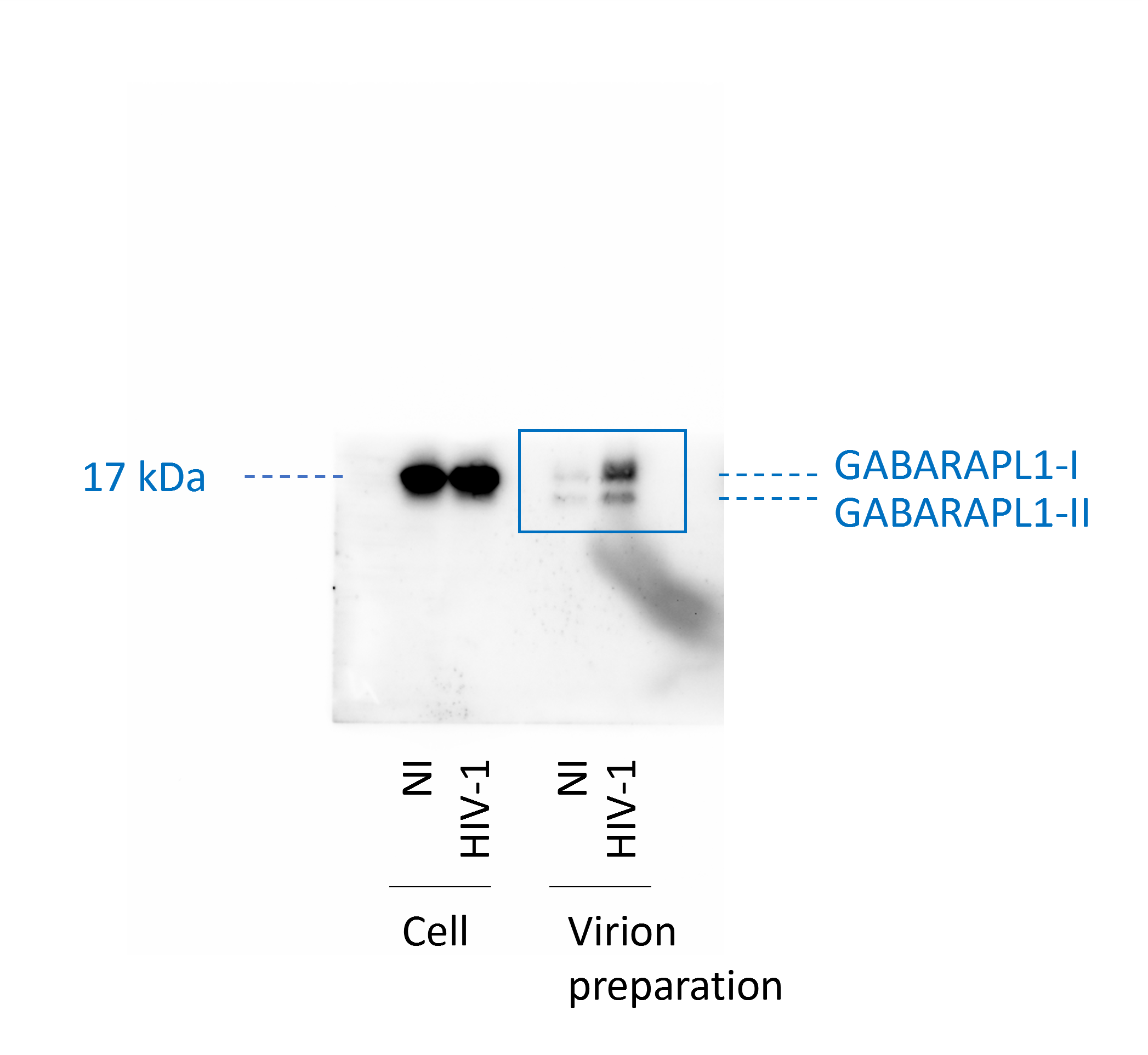

Supplement: Supplementary file 4 — Source data Fig. 2 [file 44319_2025_607_MOESM4_ESM.zip › Figure 2D/fig2D_GABARAPL1_virion prep.tif]

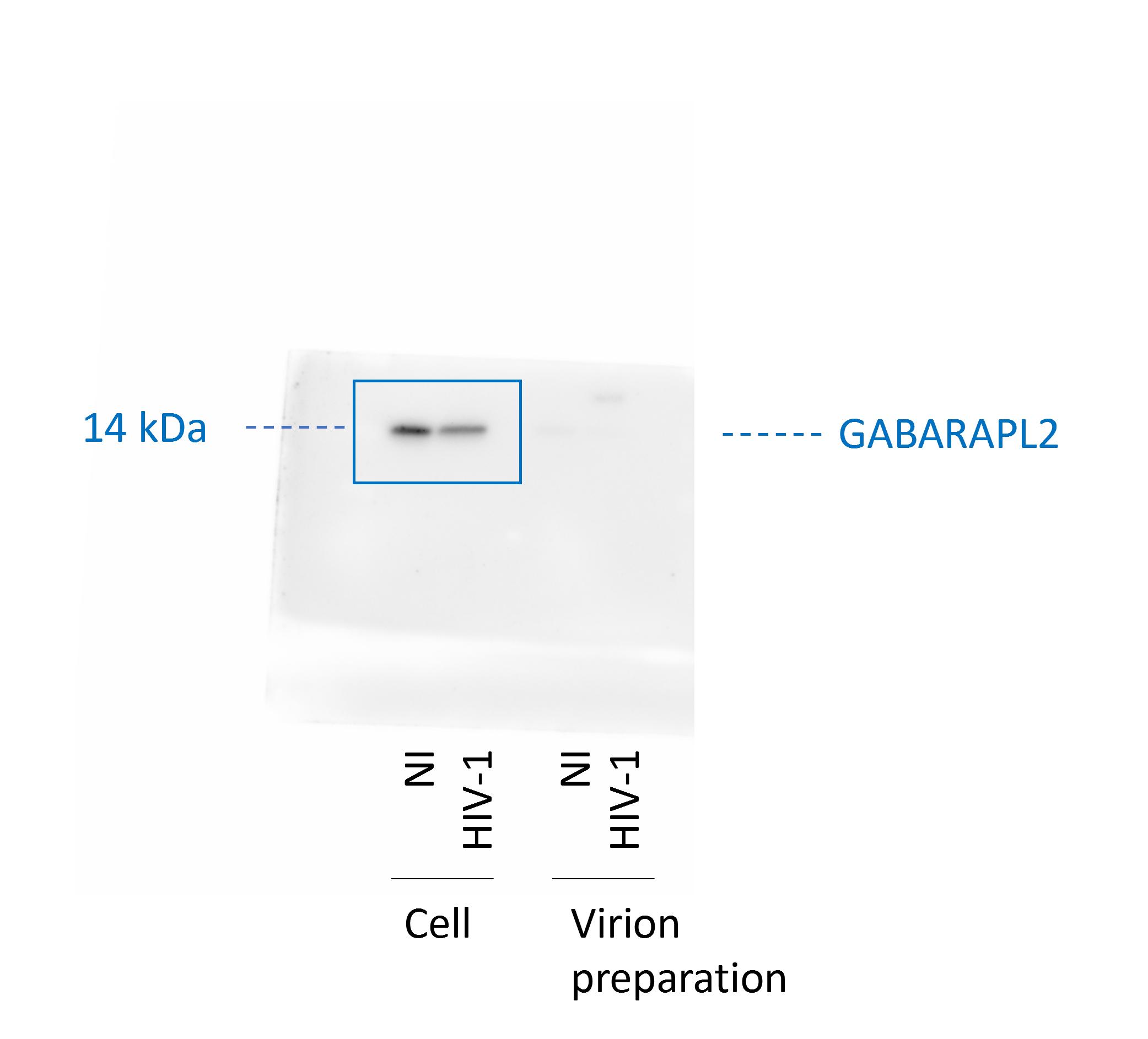

Supplement: Supplementary file 4 — Source data Fig. 2 [file 44319_2025_607_MOESM4_ESM.zip › Figure 2D/fig2D_GABARAPL2_cell.tif]

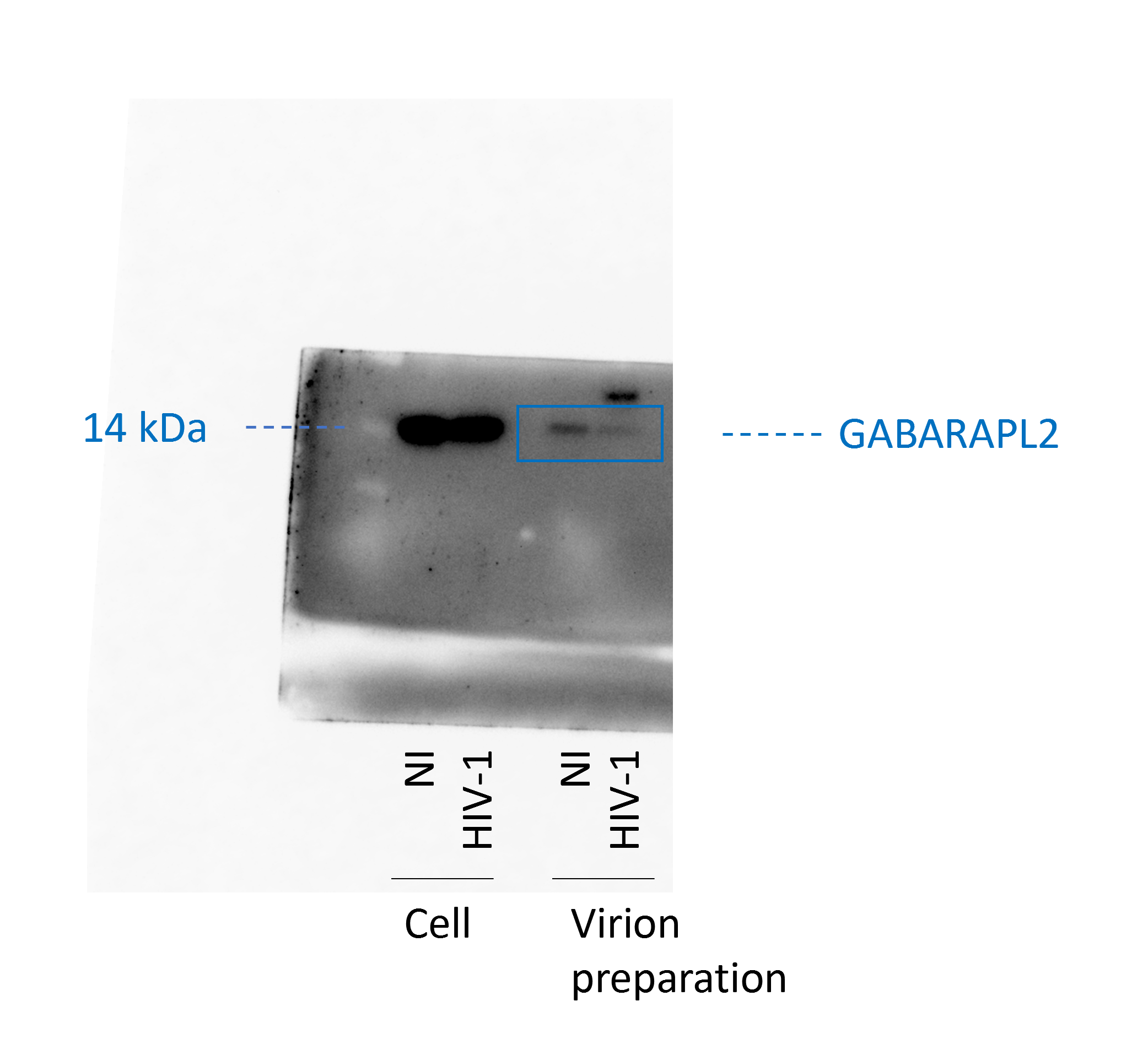

Supplement: Supplementary file 4 — Source data Fig. 2 [file 44319_2025_607_MOESM4_ESM.zip › Figure 2D/fig2D_GABARAPL2_virion prep.tif]

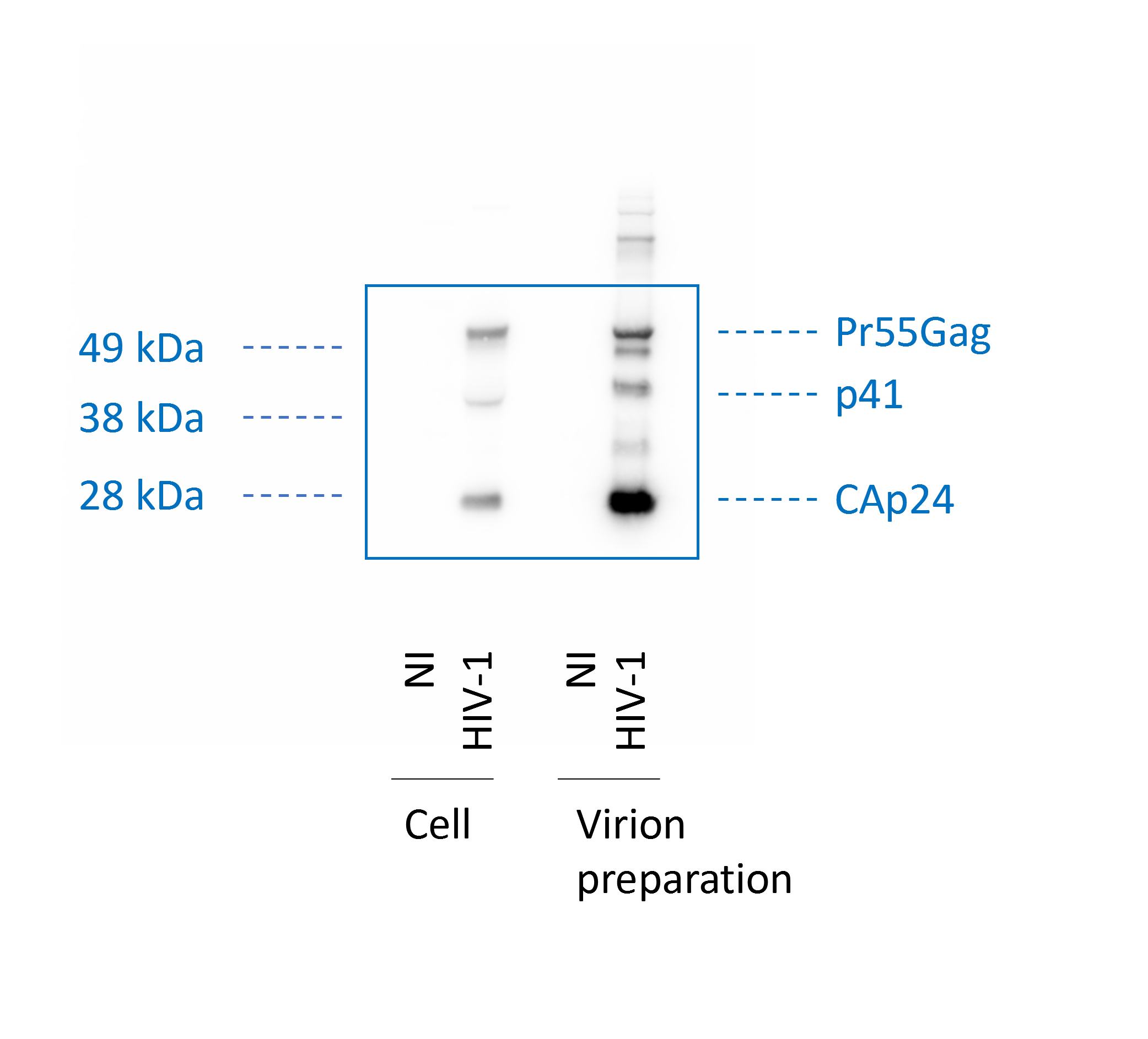

Supplement: Supplementary file 4 — Source data Fig. 2 [file 44319_2025_607_MOESM4_ESM.zip › Figure 2D/fig2D_Gag.tif]

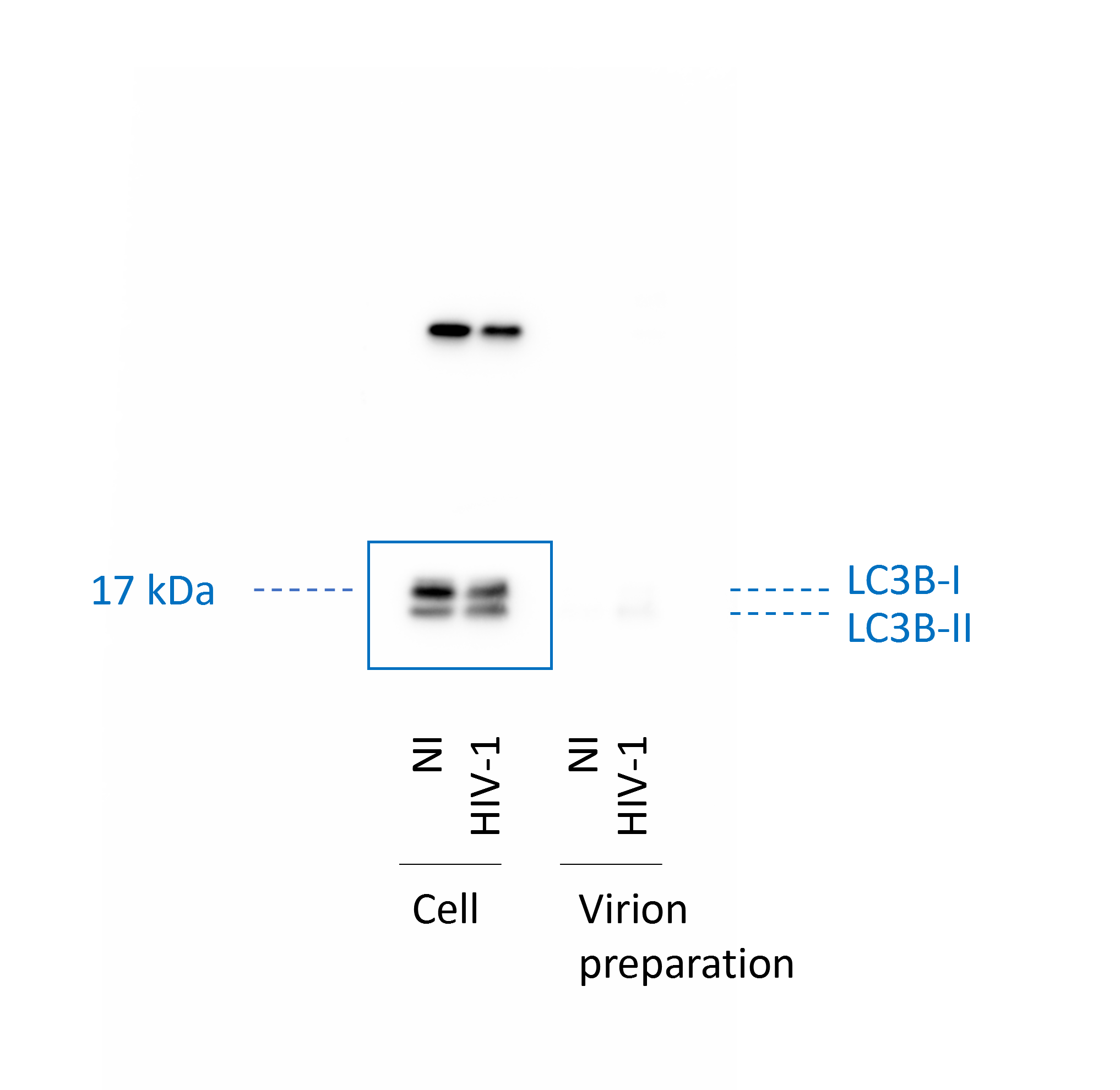

Supplement: Supplementary file 4 — Source data Fig. 2 [file 44319_2025_607_MOESM4_ESM.zip › Figure 2D/fig2D_LC3B_cell.tif]

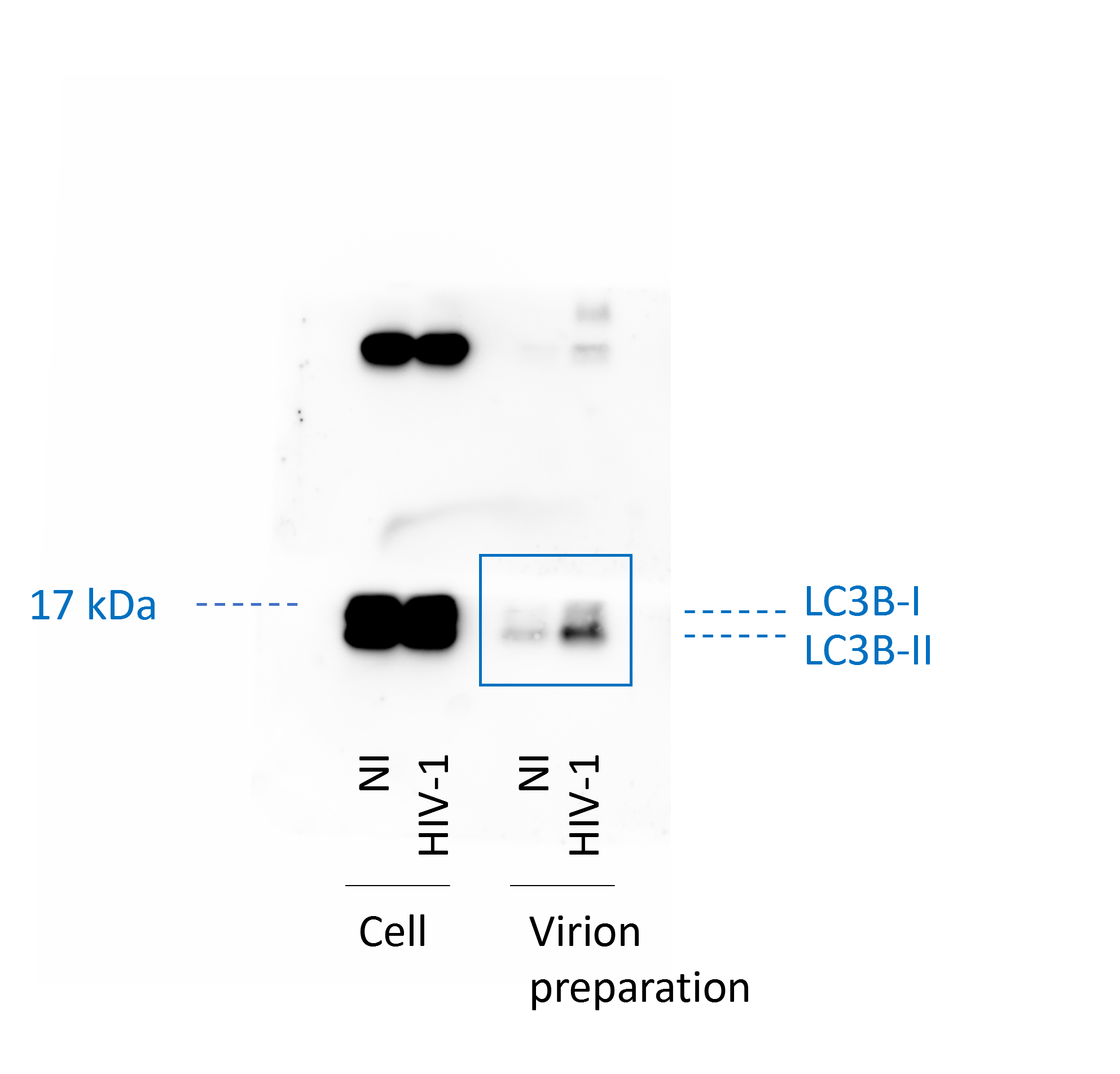

Supplement: Supplementary file 4 — Source data Fig. 2 [file 44319_2025_607_MOESM4_ESM.zip › Figure 2D/fig2D_LC3B_virion prep.tif]

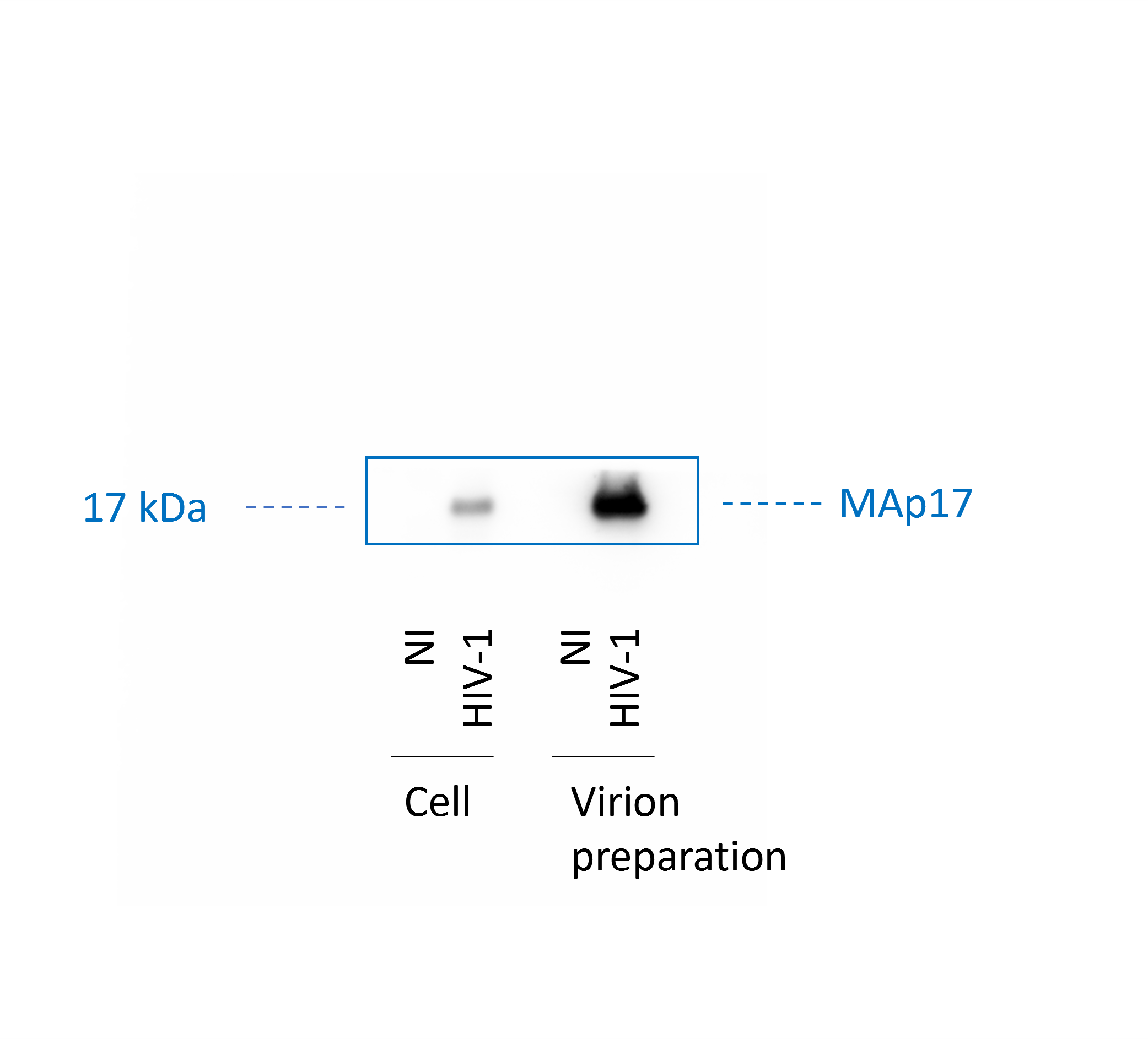

Supplement: Supplementary file 4 — Source data Fig. 2 [file 44319_2025_607_MOESM4_ESM.zip › Figure 2D/fig2D_MAp17.tif]

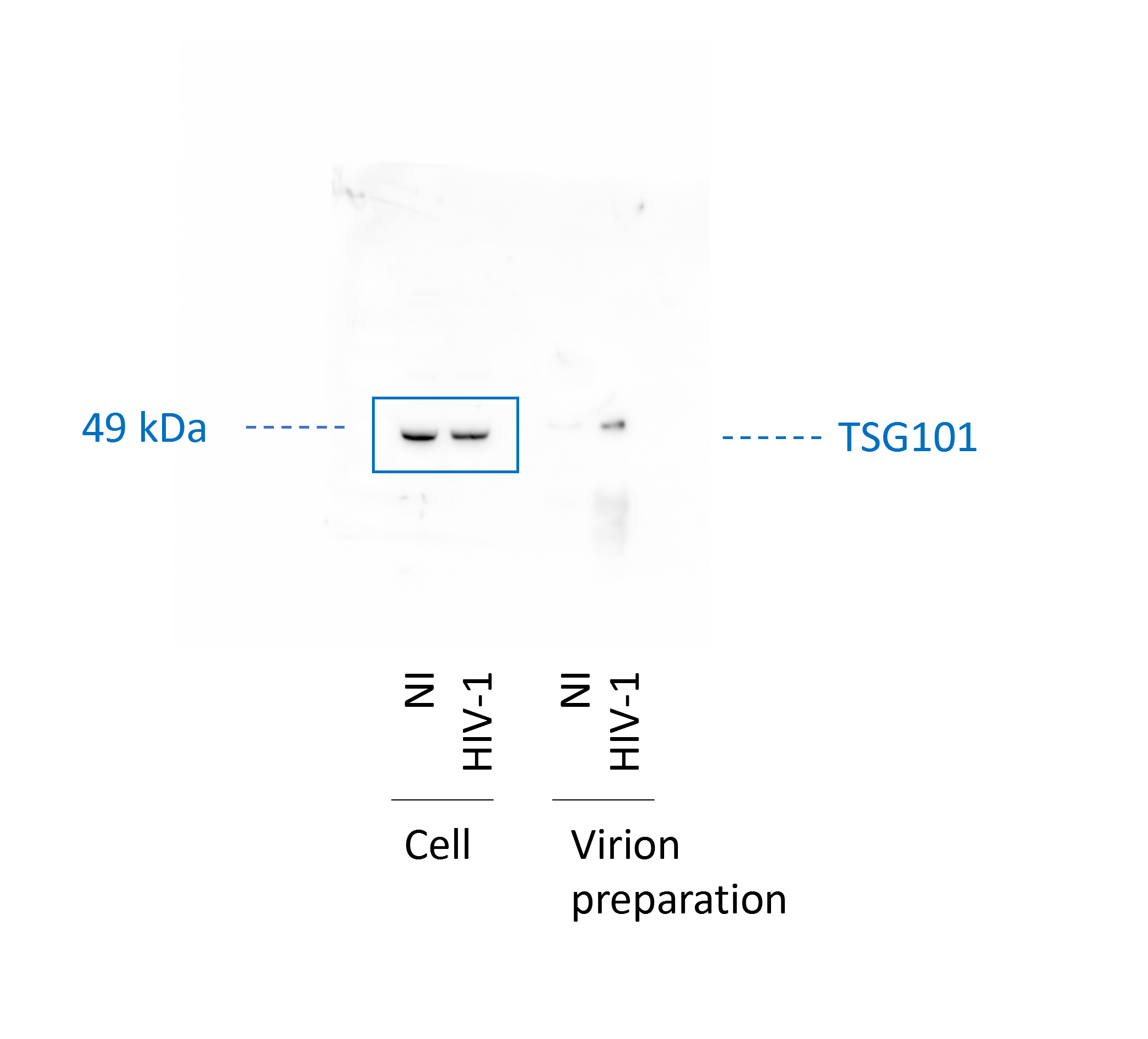

Supplement: Supplementary file 4 — Source data Fig. 2 [file 44319_2025_607_MOESM4_ESM.zip › Figure 2D/fig2D_TSG101_cell.tif]

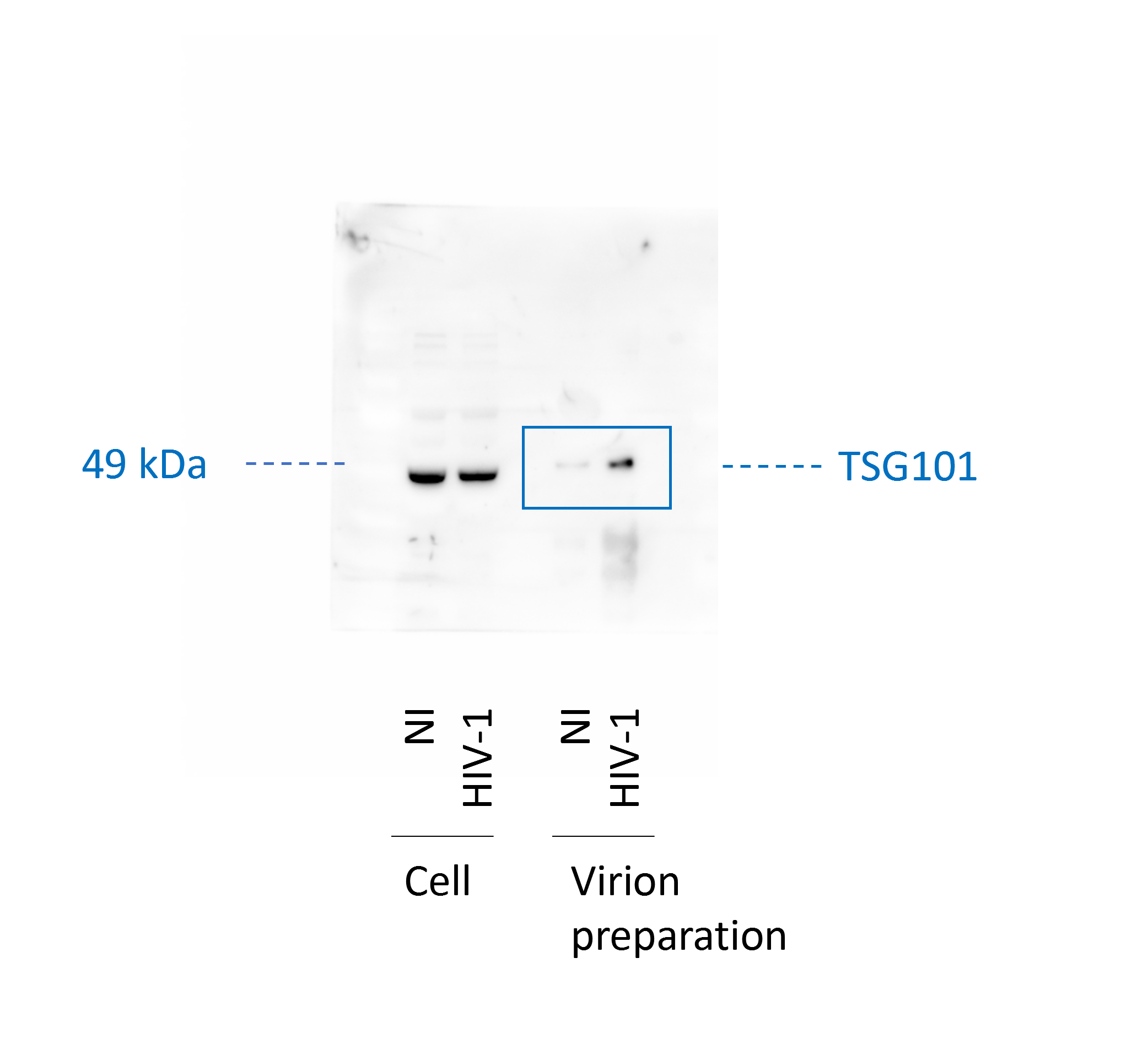

Supplement: Supplementary file 4 — Source data Fig. 2 [file 44319_2025_607_MOESM4_ESM.zip › Figure 2D/fig2D_TSG101_virion prep.tif]

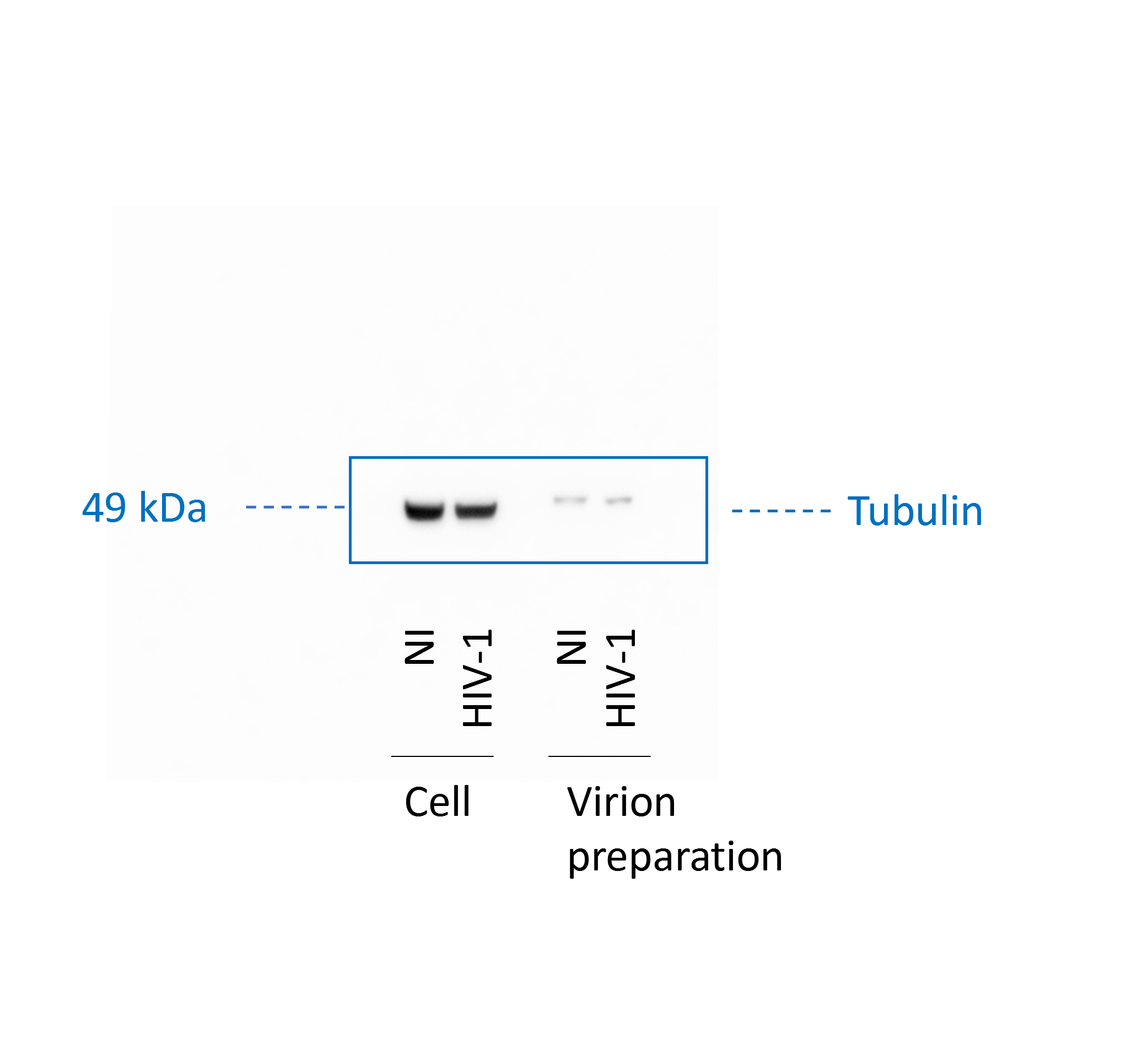

Supplement: Supplementary file 4 — Source data Fig. 2 [file 44319_2025_607_MOESM4_ESM.zip › Figure 2D/fig2D_Tubulin.tif]

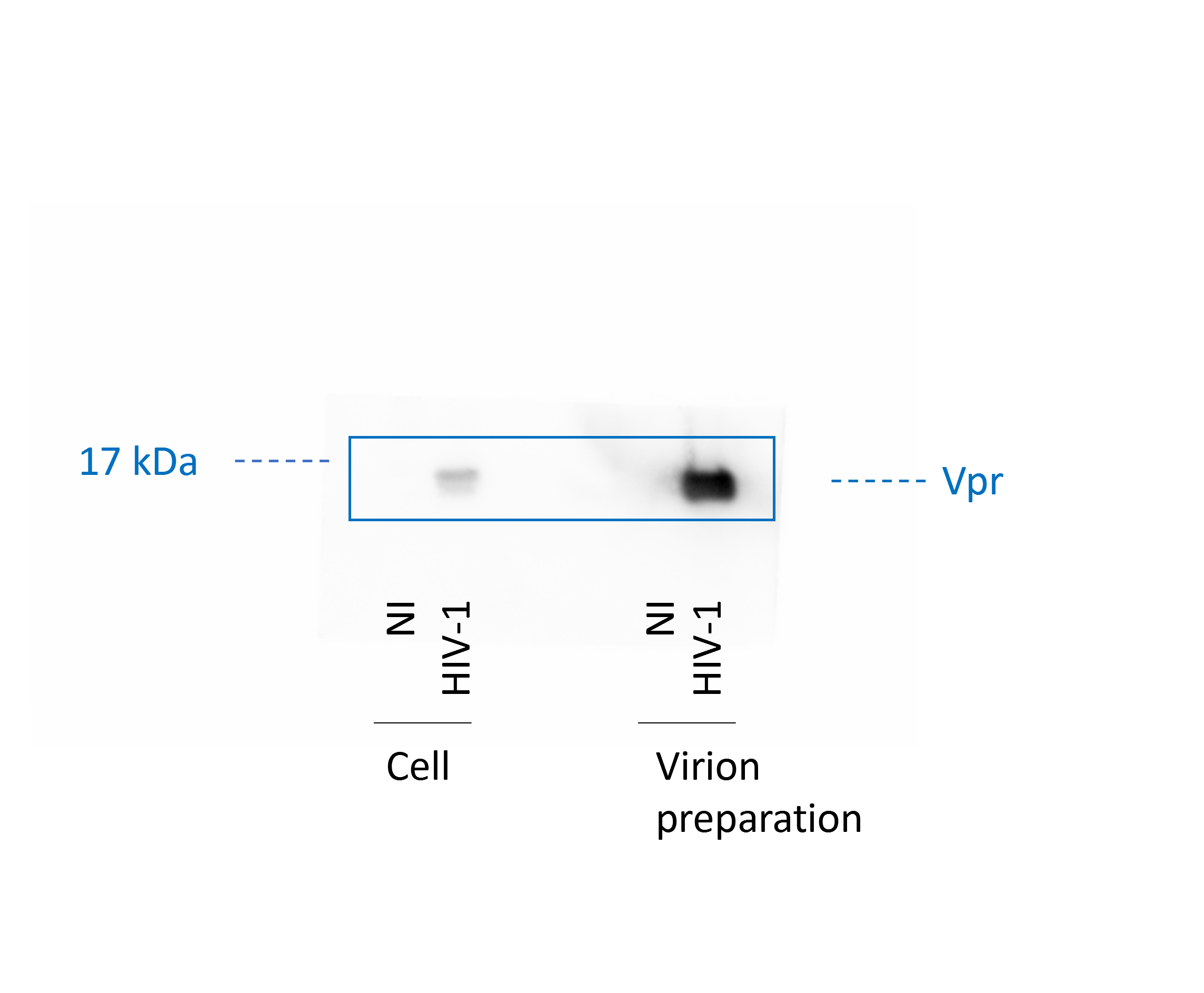

Supplement: Supplementary file 4 — Source data Fig. 2 [file 44319_2025_607_MOESM4_ESM.zip › Figure 2D/fig2D_Vpr.tif]

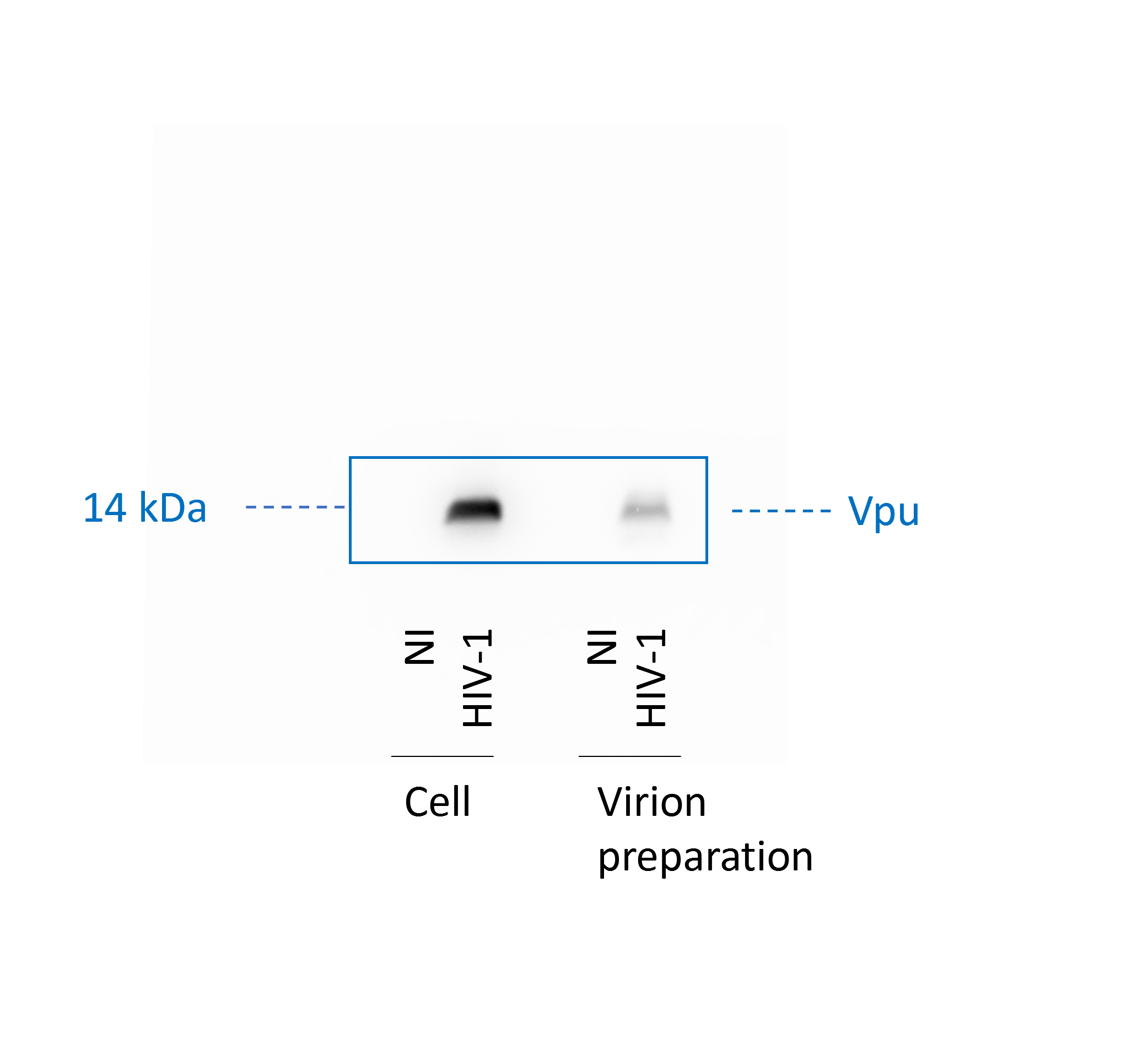

Supplement: Supplementary file 4 — Source data Fig. 2 [file 44319_2025_607_MOESM4_ESM.zip › Figure 2D/fig2D_Vpu.tif]

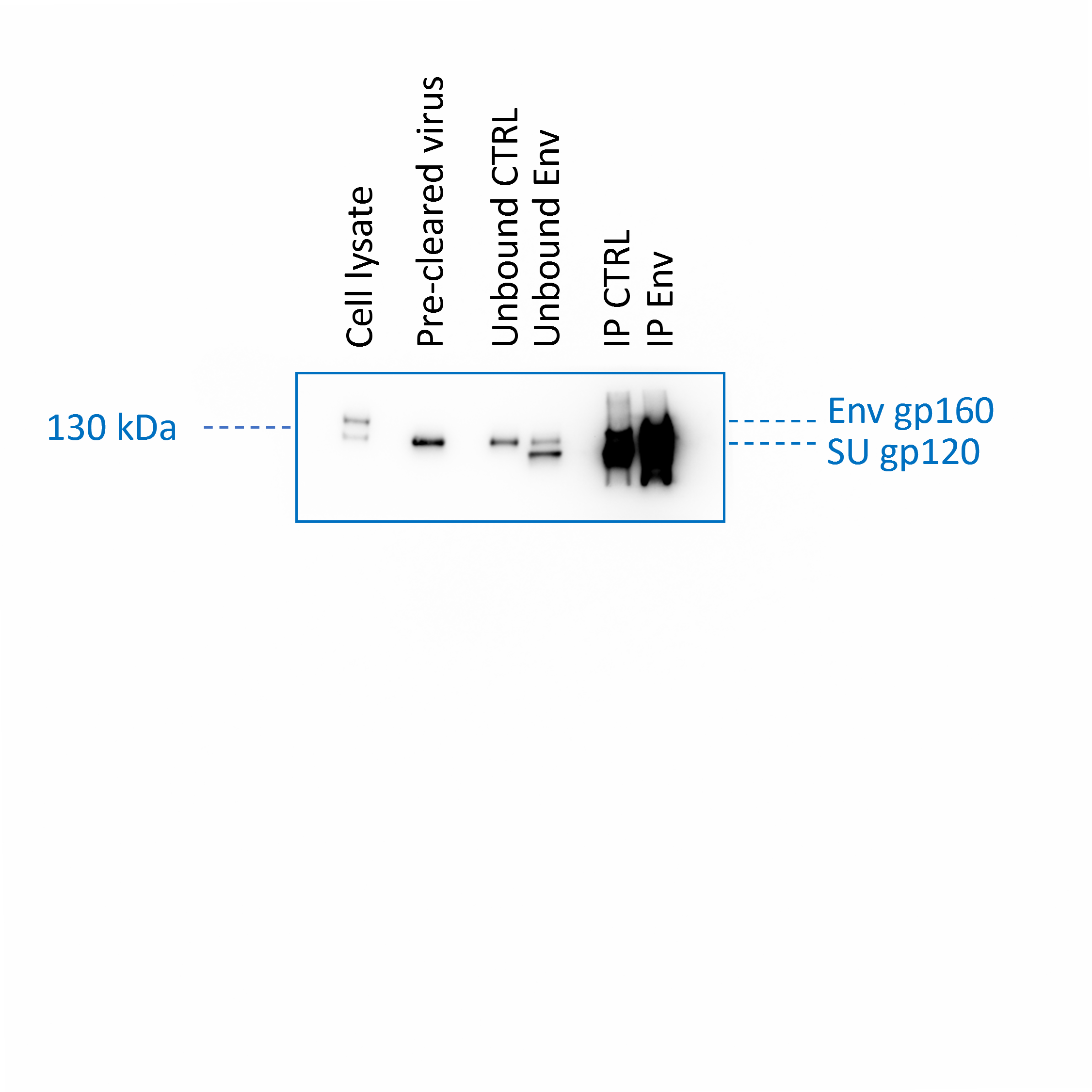

Supplement: Supplementary file 4 — Source data Fig. 2 [file 44319_2025_607_MOESM4_ESM.zip › Figure 2E/fig2E_Env_high exp.tif]

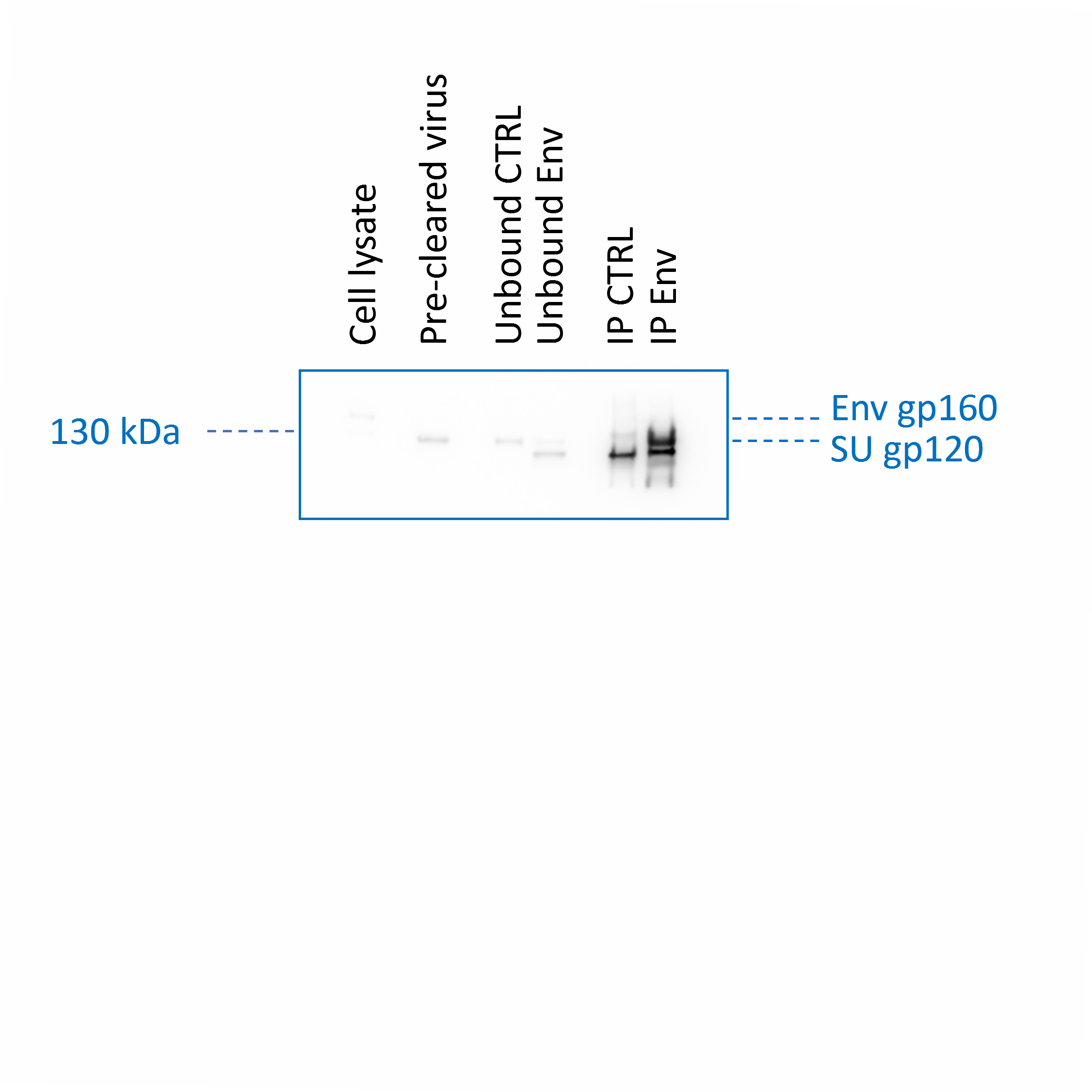

Supplement: Supplementary file 4 — Source data Fig. 2 [file 44319_2025_607_MOESM4_ESM.zip › Figure 2E/fig2E_Env_low exp.tif]

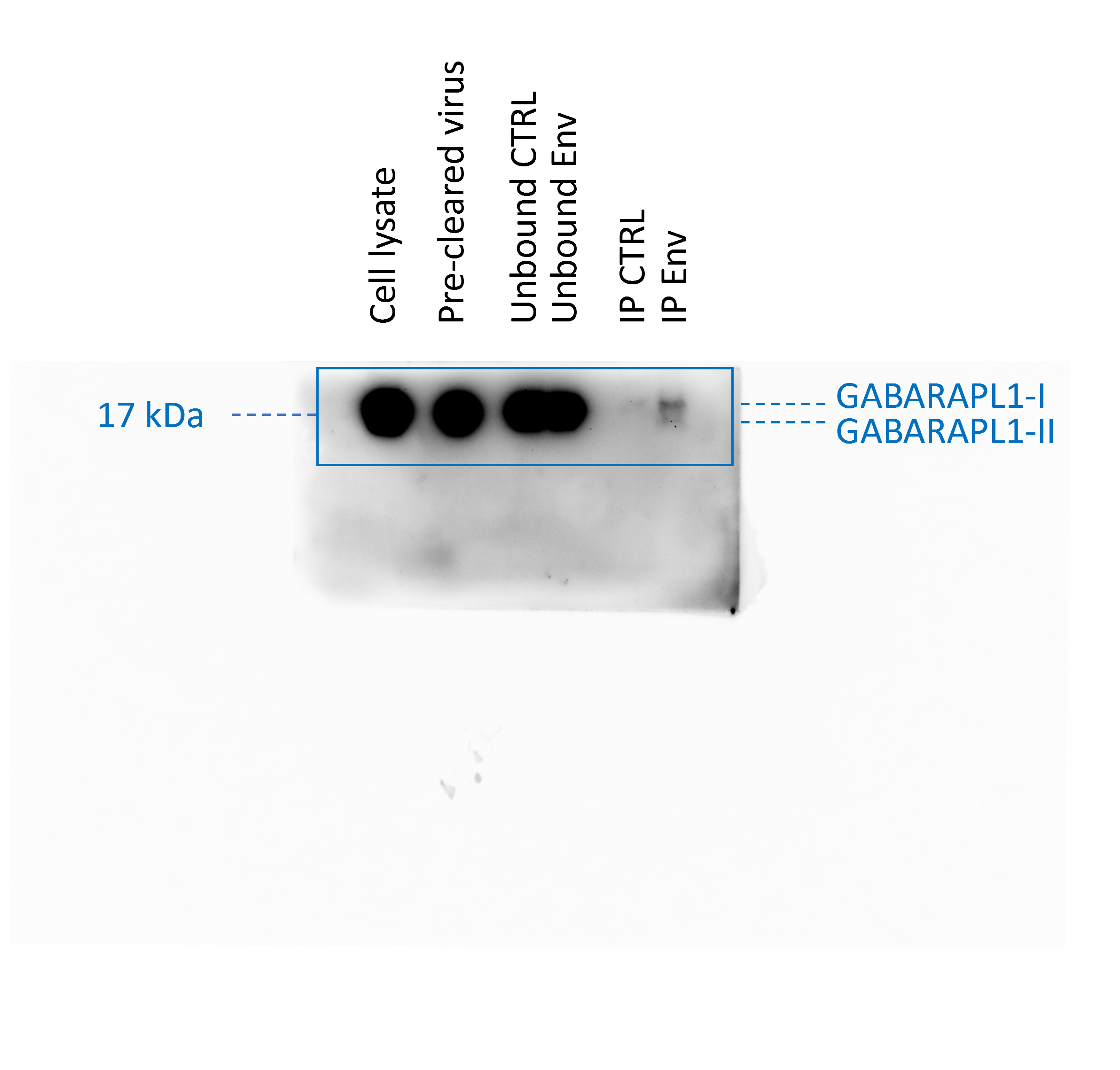

Supplement: Supplementary file 4 — Source data Fig. 2 [file 44319_2025_607_MOESM4_ESM.zip › Figure 2E/fig2E_GABARAPL1.tif]

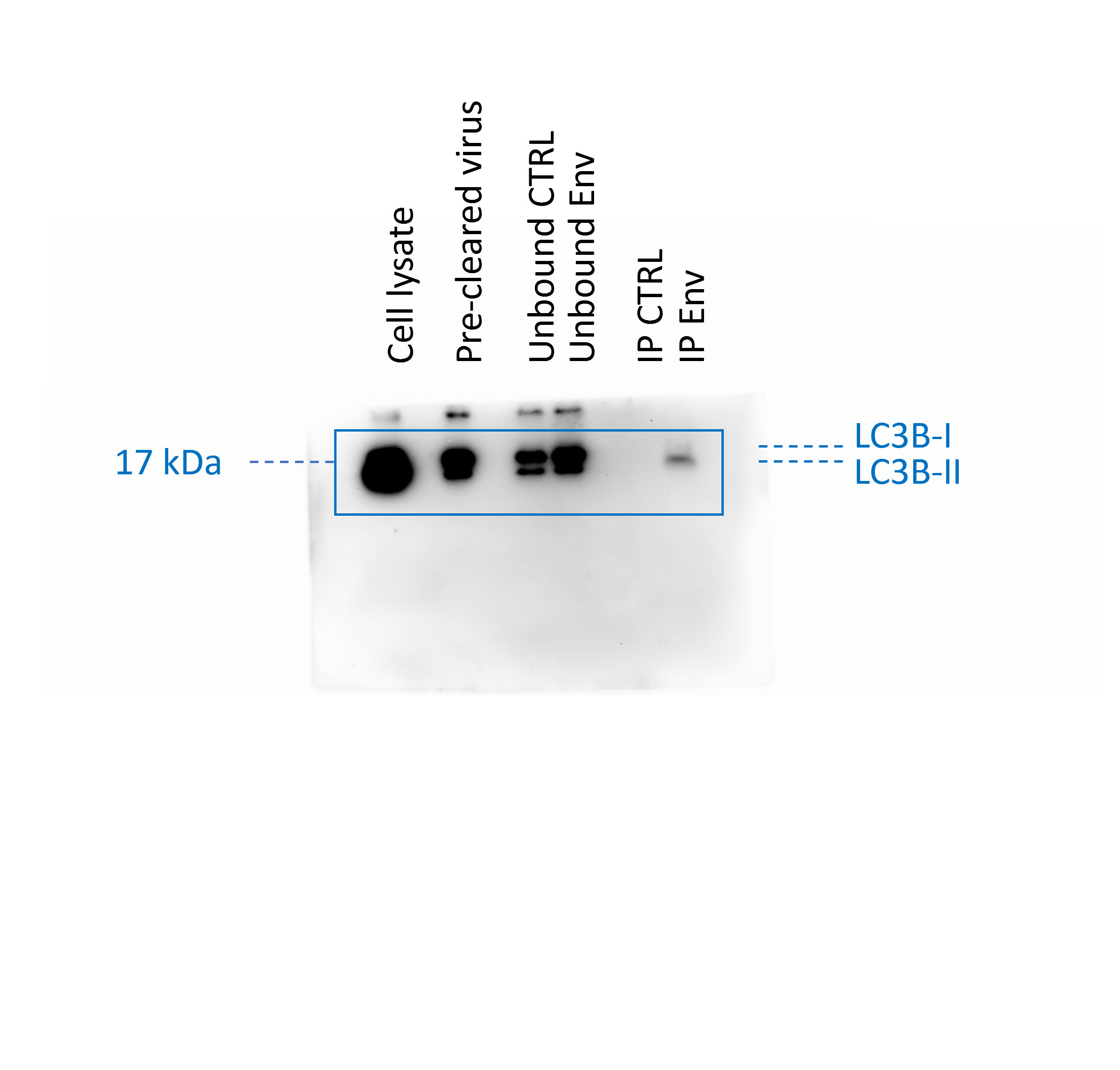

Supplement: Supplementary file 4 — Source data Fig. 2 [file 44319_2025_607_MOESM4_ESM.zip › Figure 2E/fig2E_LC3B.tif]

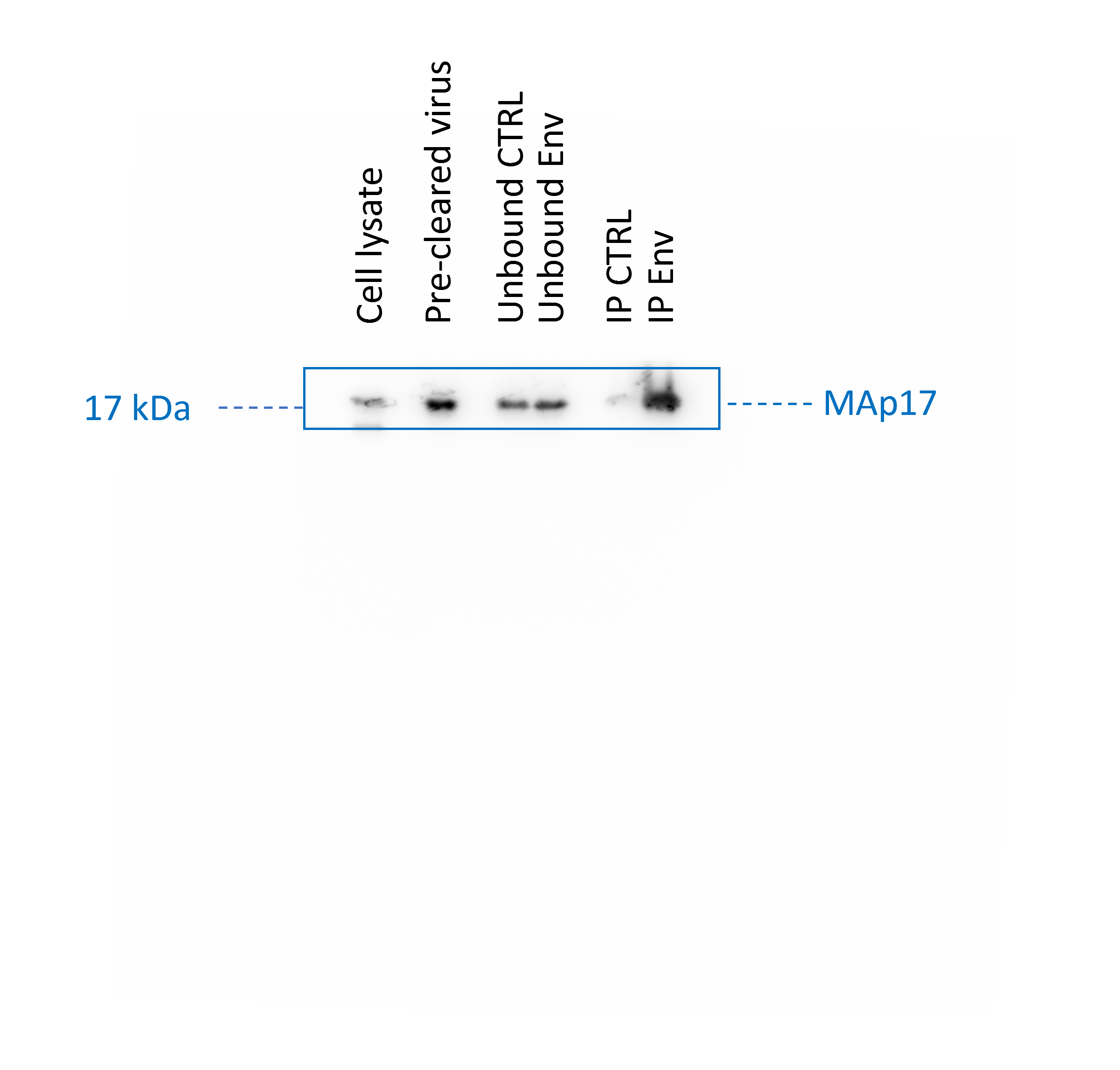

Supplement: Supplementary file 4 — Source data Fig. 2 [file 44319_2025_607_MOESM4_ESM.zip › Figure 2E/fig2E_MAp17.tif]

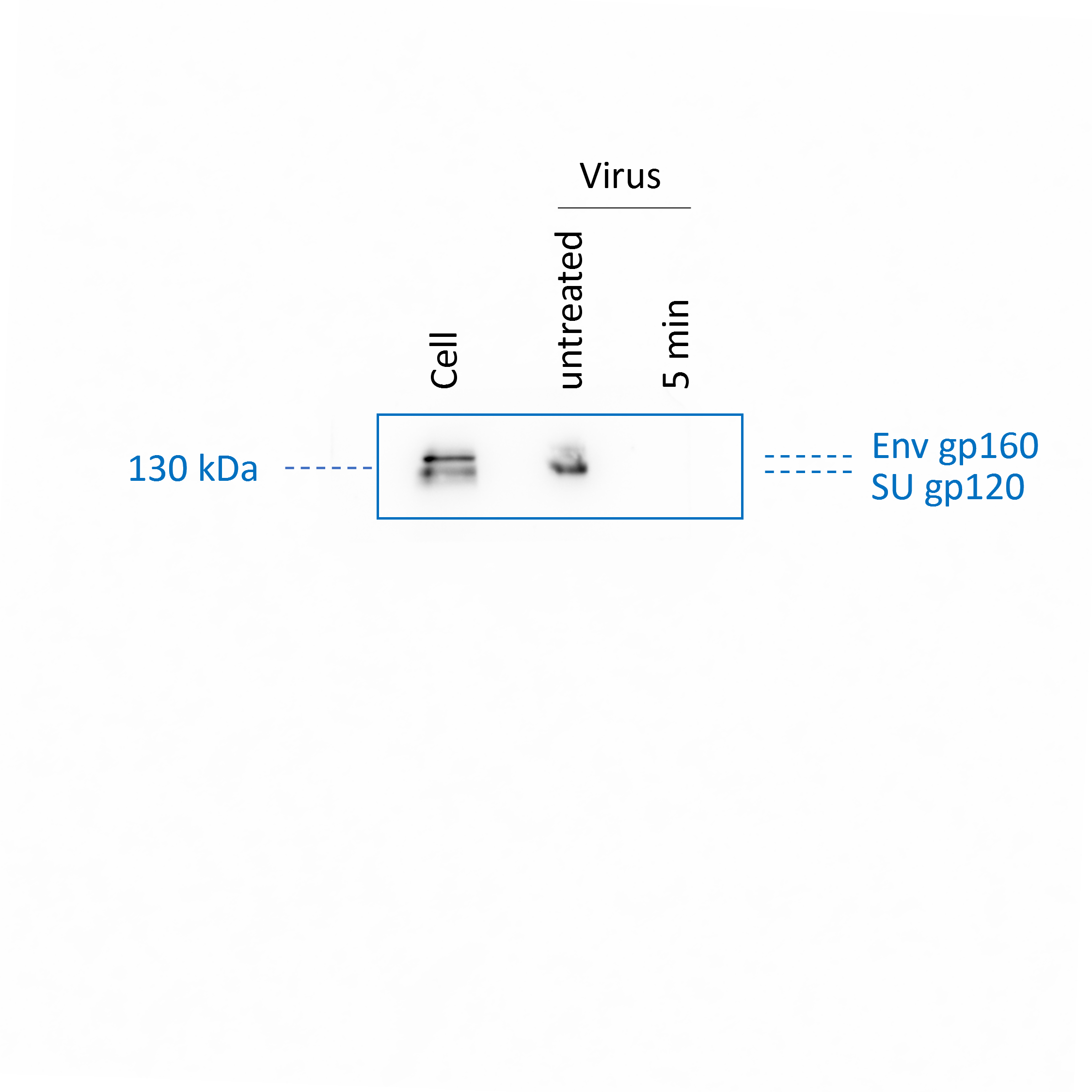

Supplement: Supplementary file 4 — Source data Fig. 2 [file 44319_2025_607_MOESM4_ESM.zip › Figure 2F/fig2F_Env.tif]

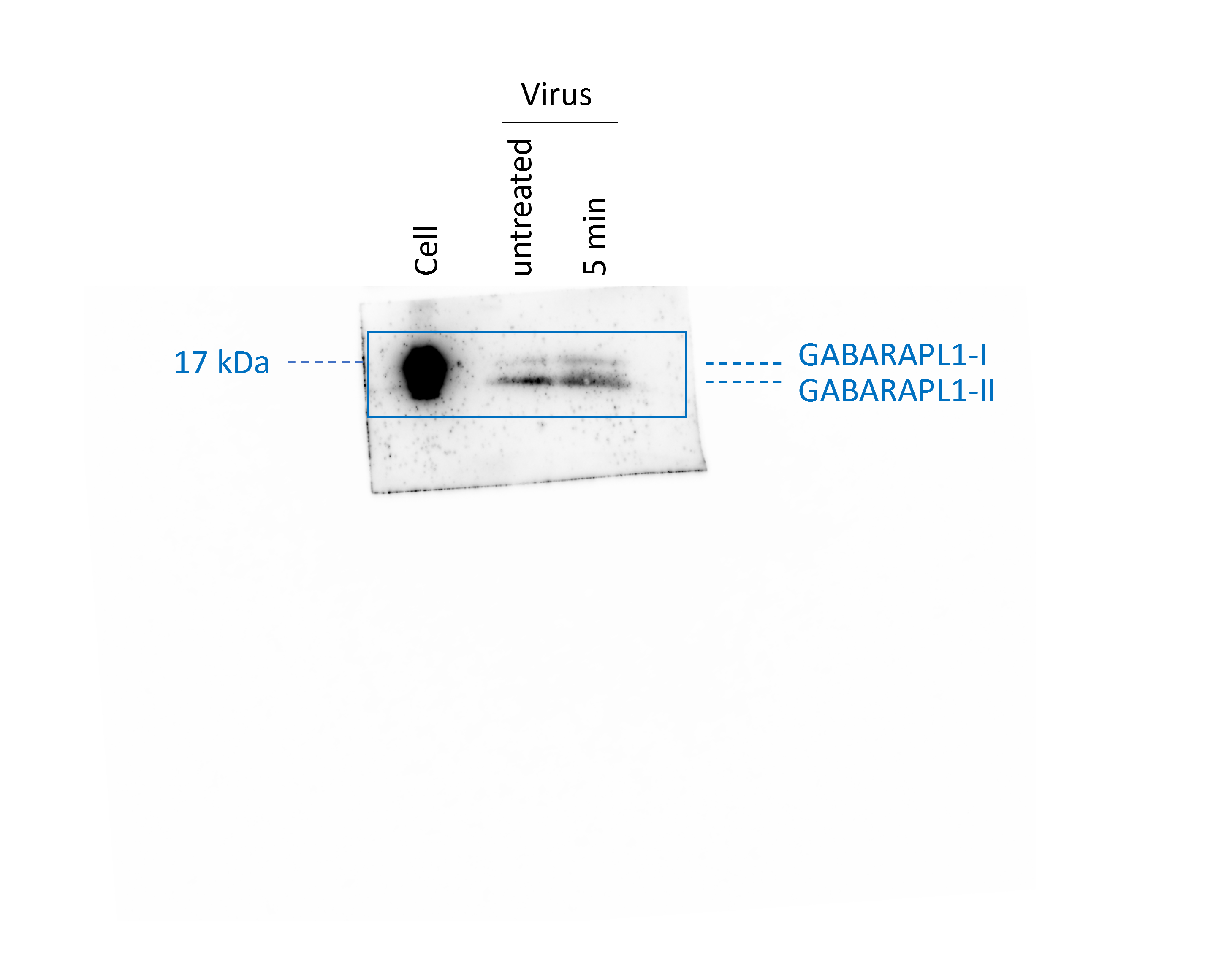

Supplement: Supplementary file 4 — Source data Fig. 2 [file 44319_2025_607_MOESM4_ESM.zip › Figure 2F/fig2F_GABARAPL1.tif]

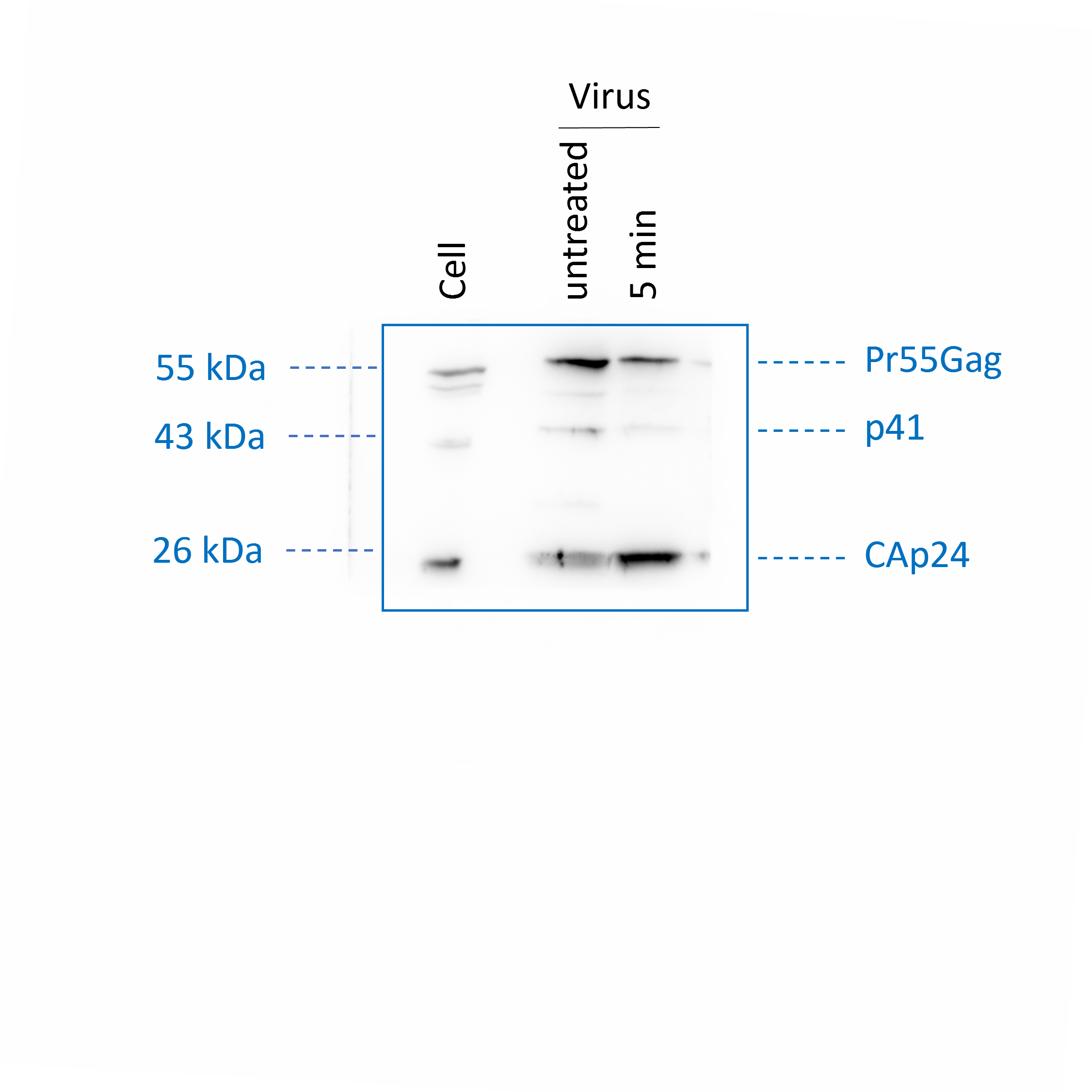

Supplement: Supplementary file 4 — Source data Fig. 2 [file 44319_2025_607_MOESM4_ESM.zip › Figure 2F/fig2F_Gag.tif]

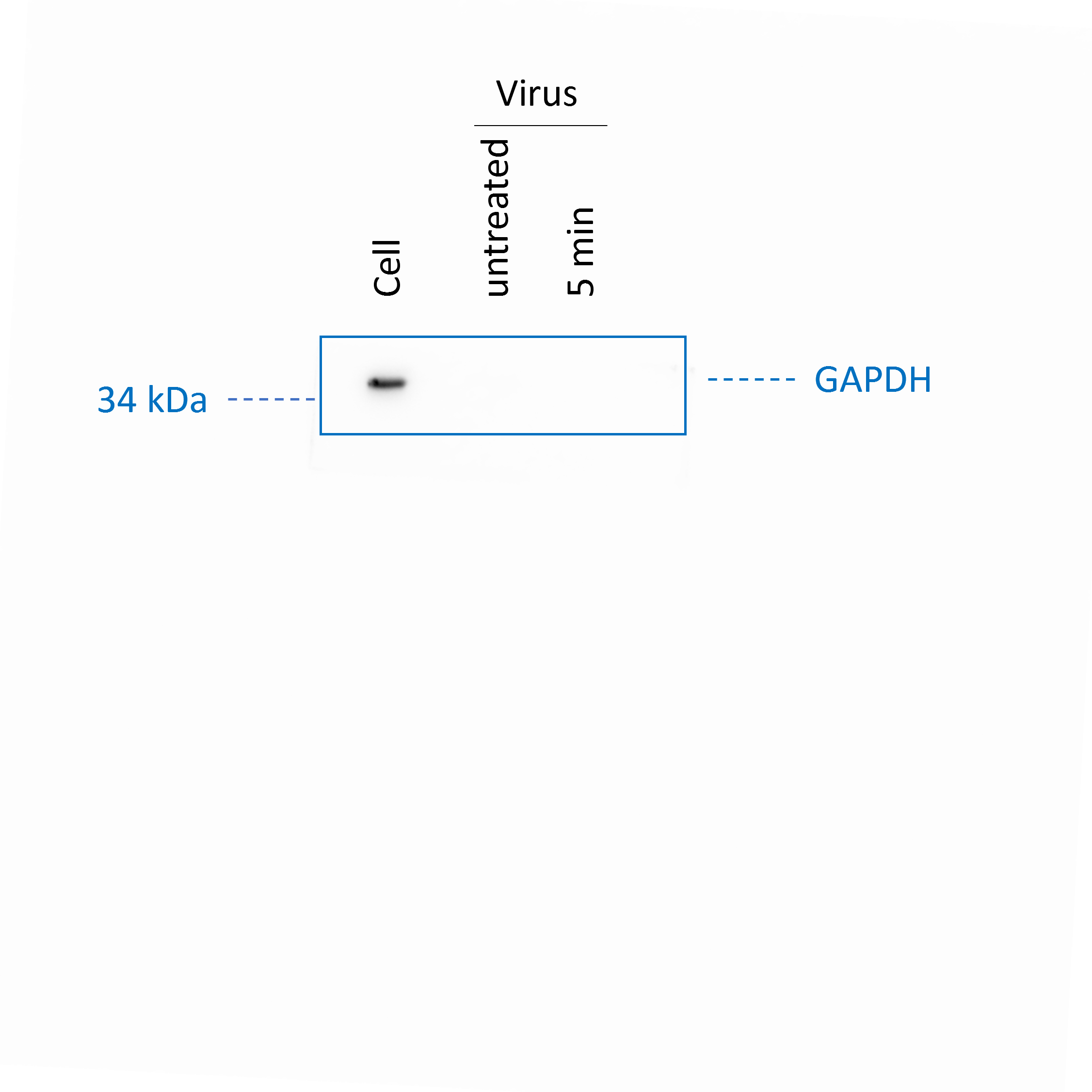

Supplement: Supplementary file 4 — Source data Fig. 2 [file 44319_2025_607_MOESM4_ESM.zip › Figure 2F/fig2F_GAPDH.tif]

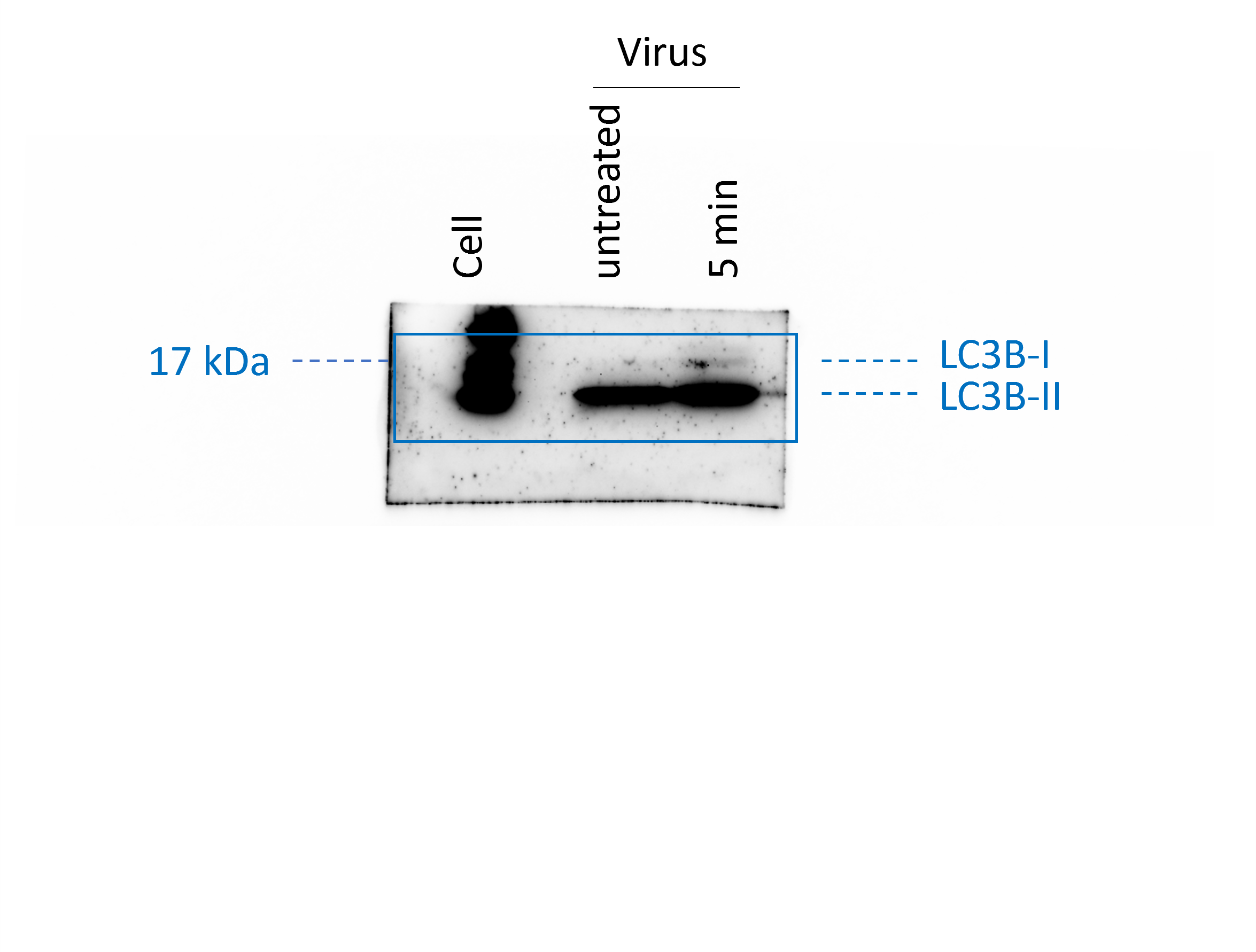

Supplement: Supplementary file 4 — Source data Fig. 2 [file 44319_2025_607_MOESM4_ESM.zip › Figure 2F/fig2F_LC3B.tif]

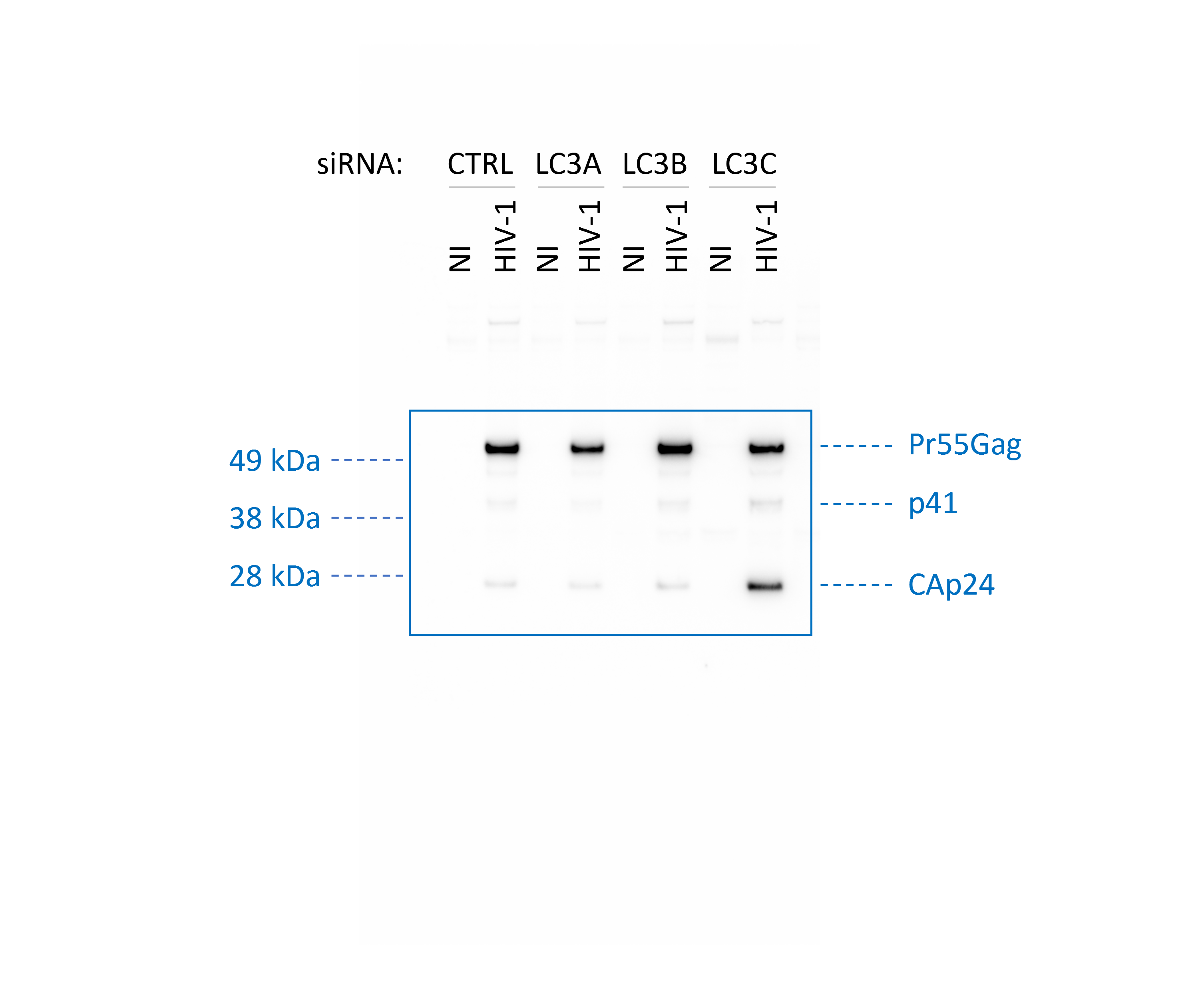

Supplement: Supplementary file 5 — Source data Fig. 3 [file 44319_2025_607_MOESM5_ESM.zip › Figure 3A/fig3A_Gag_cell.tif]

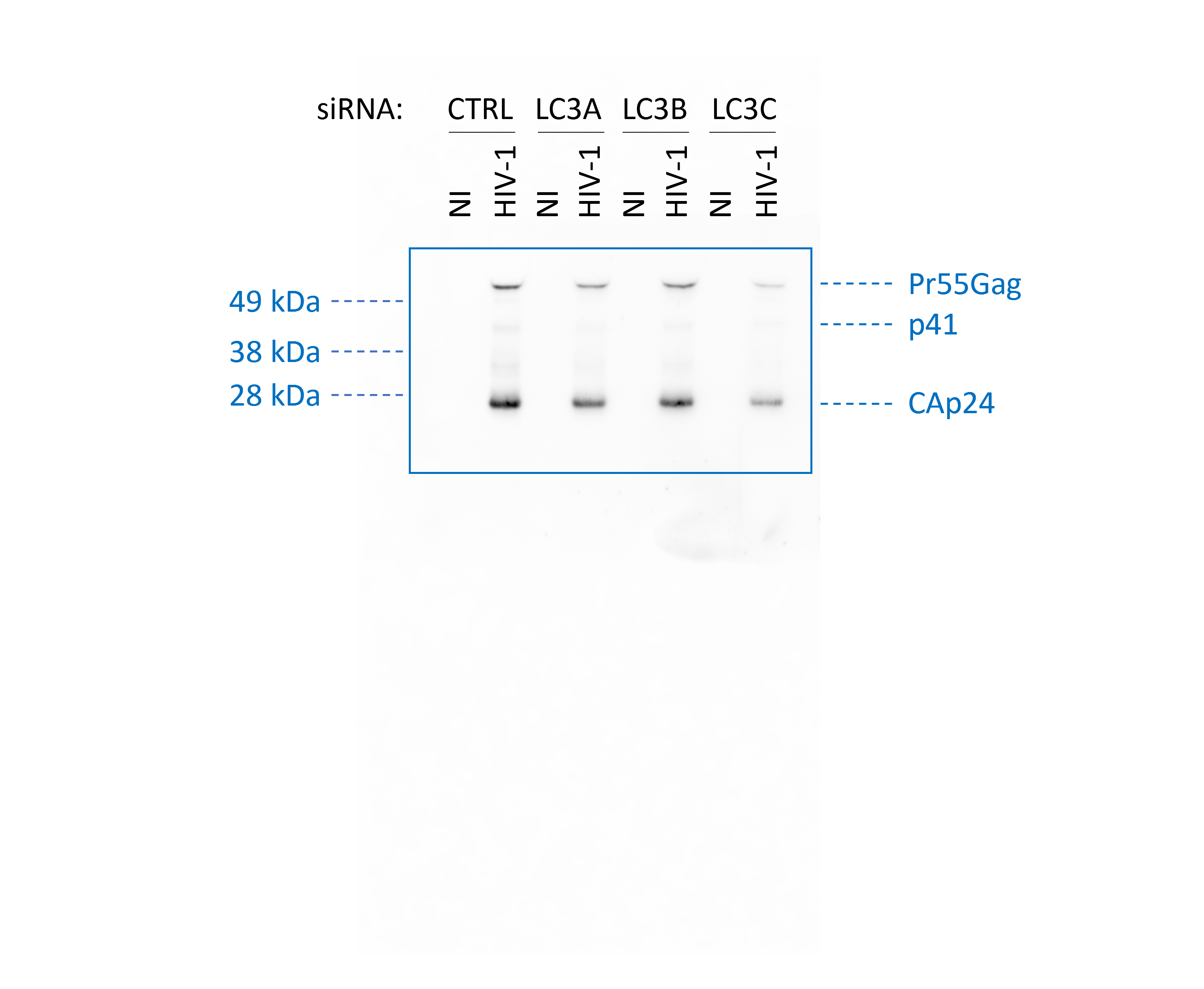

Supplement: Supplementary file 5 — Source data Fig. 3 [file 44319_2025_607_MOESM5_ESM.zip › Figure 3A/fig3A_Gag_virion prep.tif]

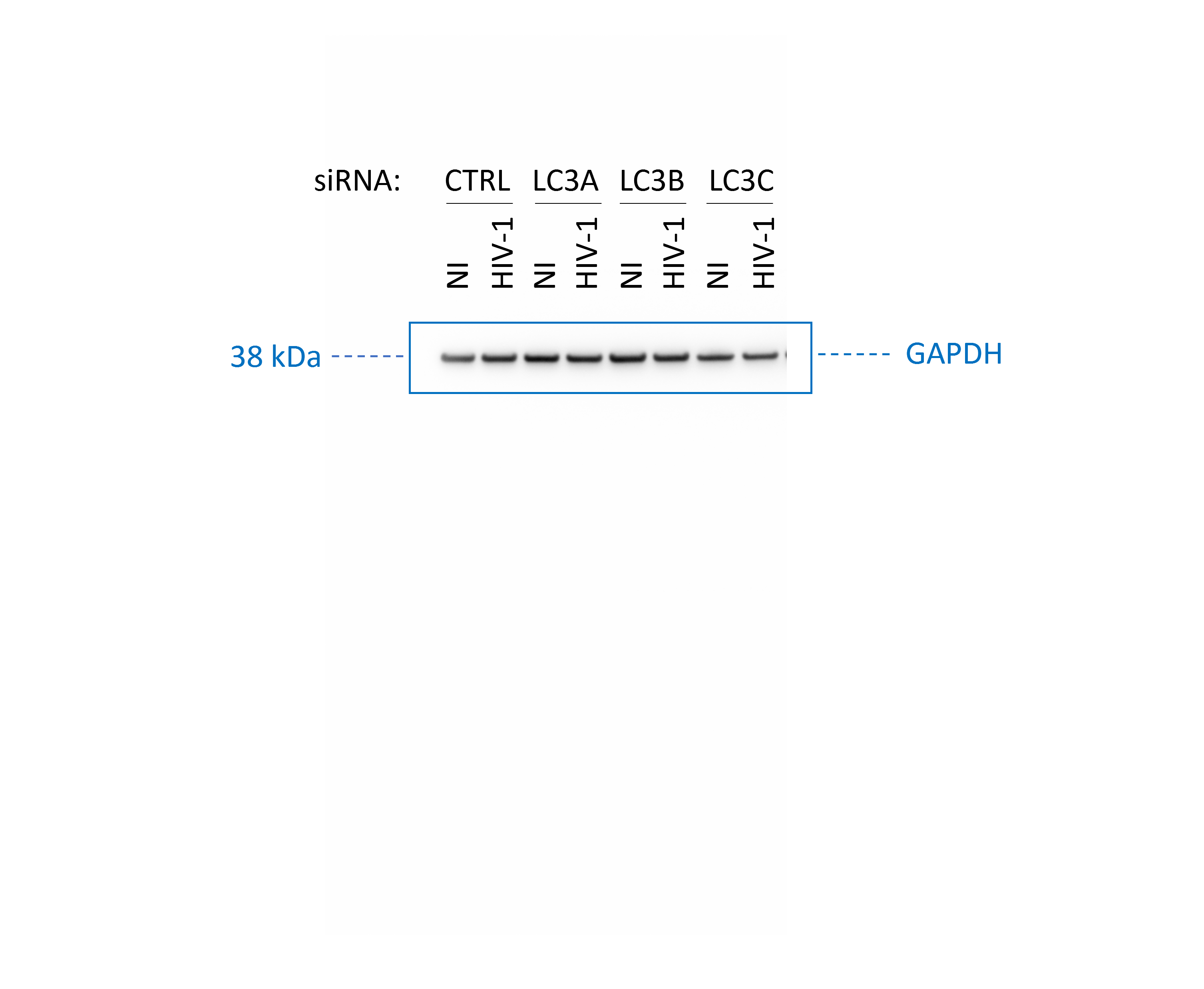

Supplement: Supplementary file 5 — Source data Fig. 3 [file 44319_2025_607_MOESM5_ESM.zip › Figure 3A/fig3A_GAPDH.tif]

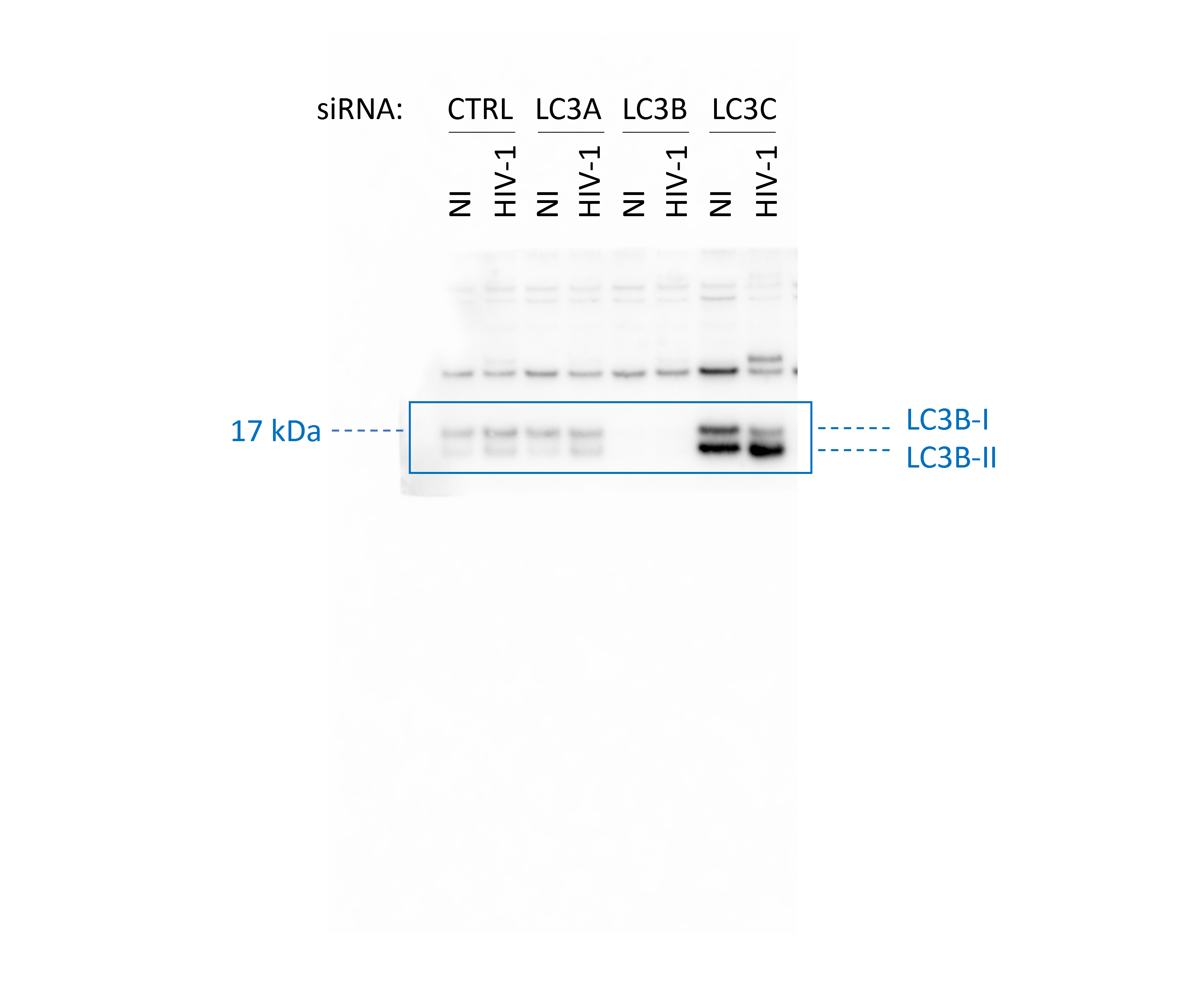

Supplement: Supplementary file 5 — Source data Fig. 3 [file 44319_2025_607_MOESM5_ESM.zip › Figure 3A/fig3A_LC3B.tif]

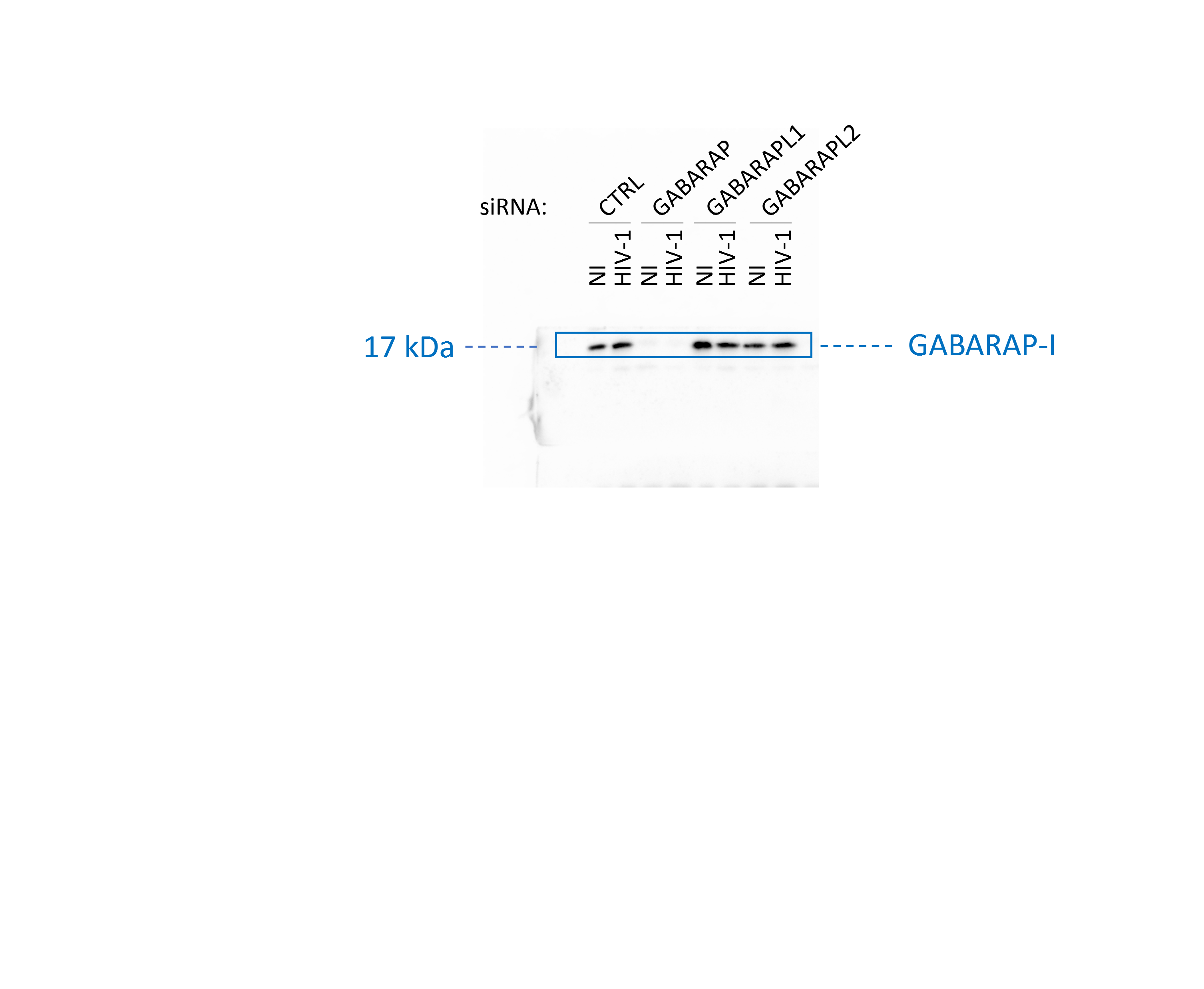

Supplement: Supplementary file 5 — Source data Fig. 3 [file 44319_2025_607_MOESM5_ESM.zip › Figure 3B/fig3B_GABARAP.tif]

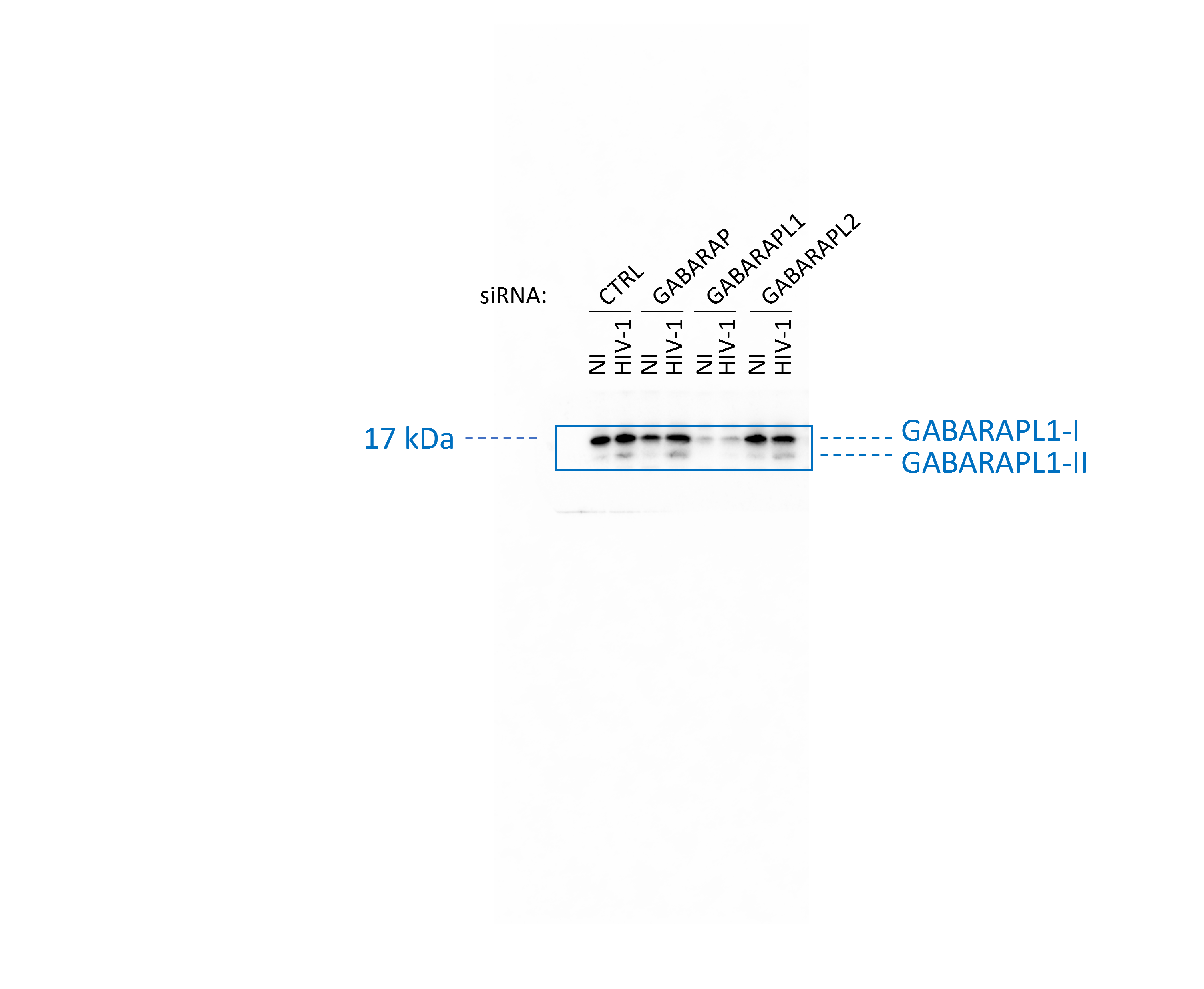

Supplement: Supplementary file 5 — Source data Fig. 3 [file 44319_2025_607_MOESM5_ESM.zip › Figure 3B/fig3B_GABARAPL1.tif]

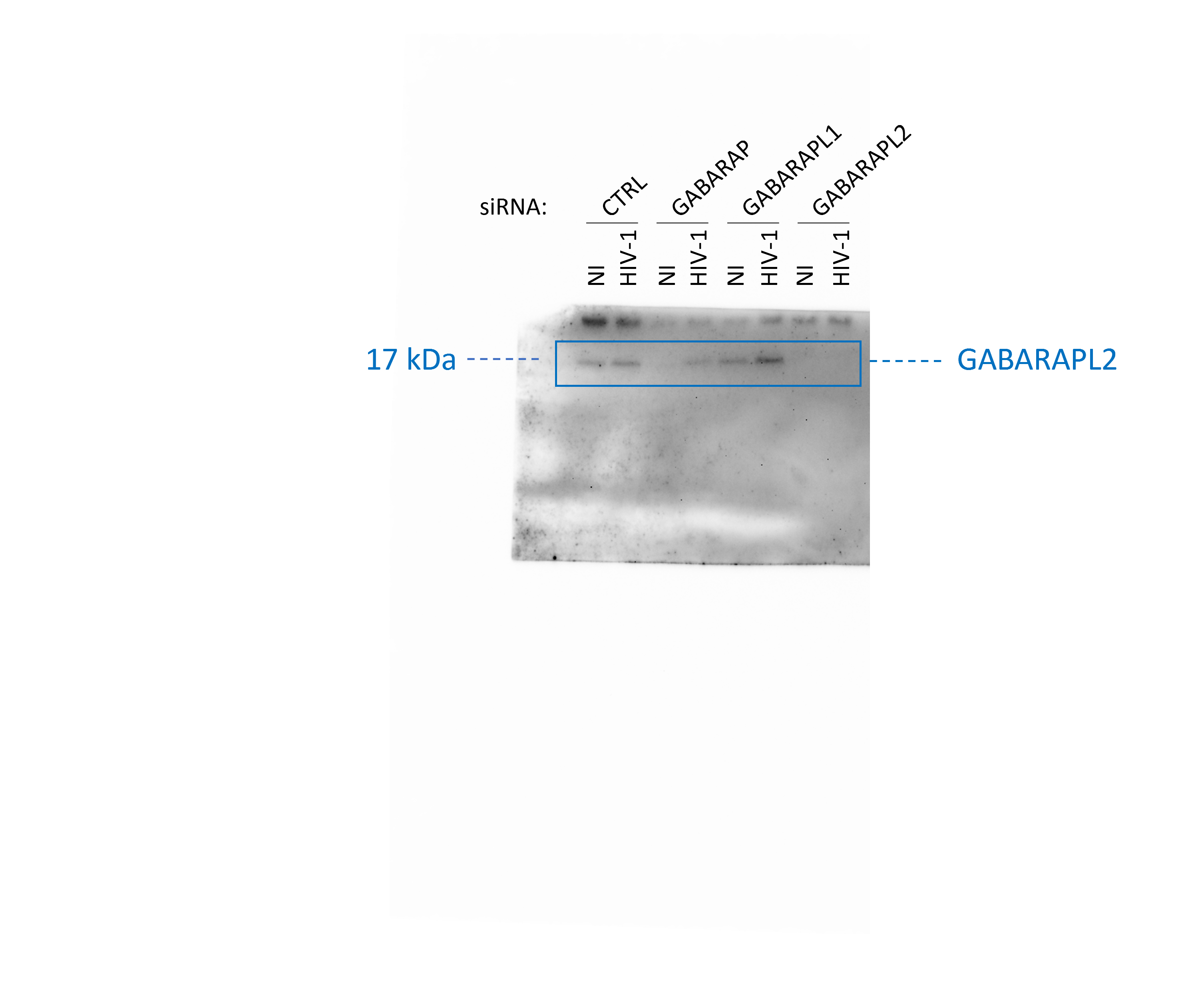

Supplement: Supplementary file 5 — Source data Fig. 3 [file 44319_2025_607_MOESM5_ESM.zip › Figure 3B/fig3B_GABARAPL2.tif]

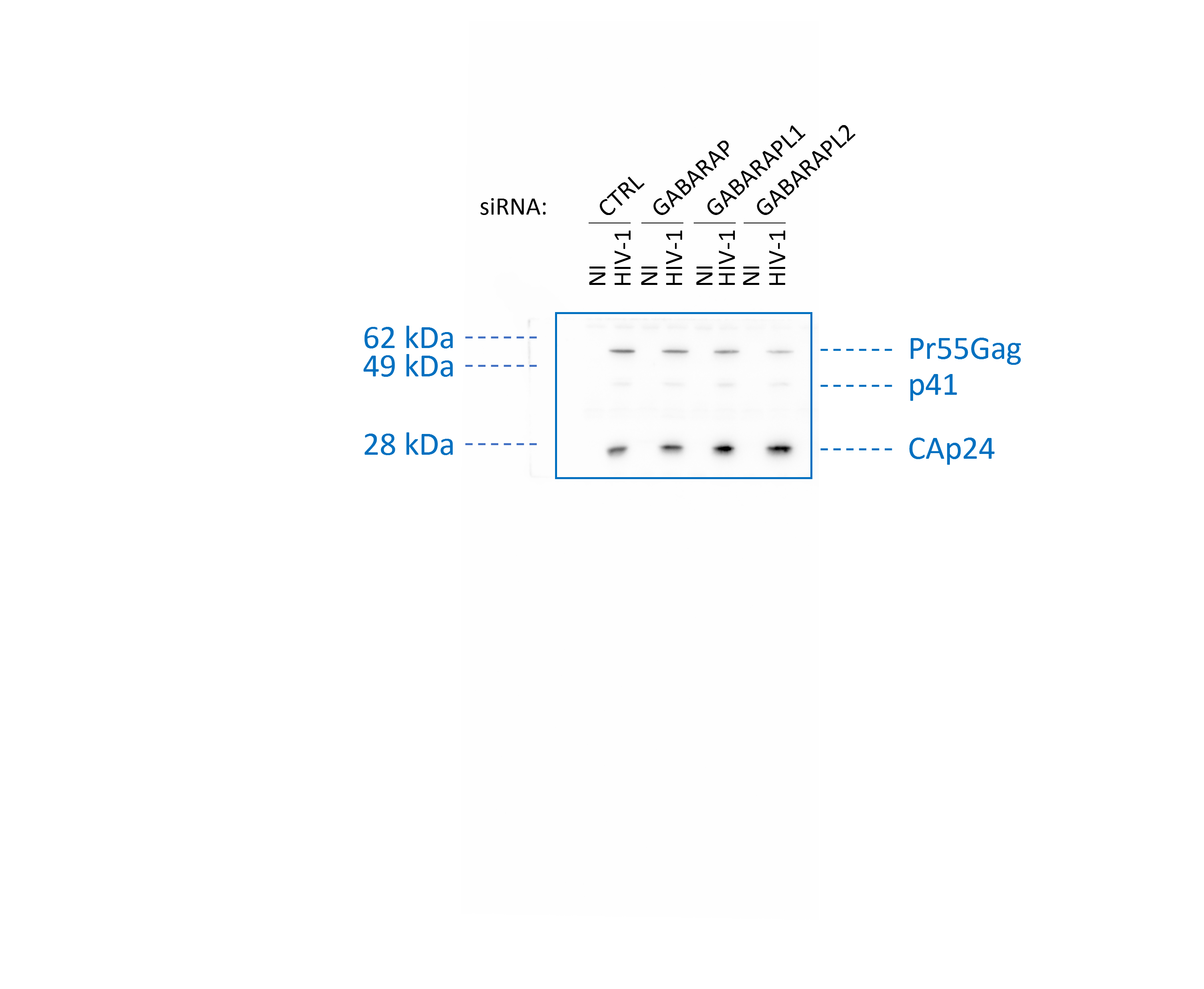

Supplement: Supplementary file 5 — Source data Fig. 3 [file 44319_2025_607_MOESM5_ESM.zip › Figure 3B/fig3B_Gag_cell.tif]

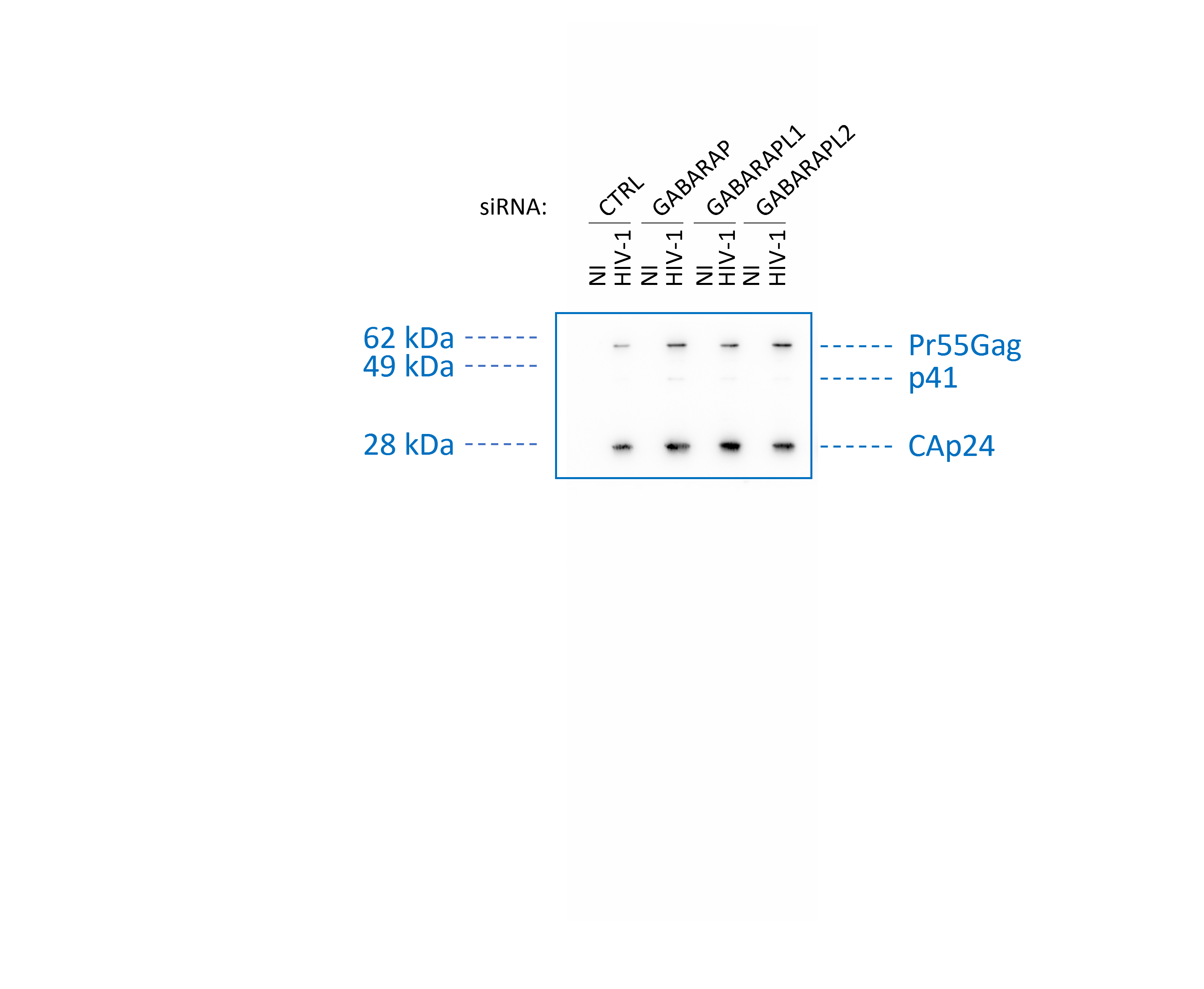

Supplement: Supplementary file 5 — Source data Fig. 3 [file 44319_2025_607_MOESM5_ESM.zip › Figure 3B/fig3B_Gag_virion prep.tif]

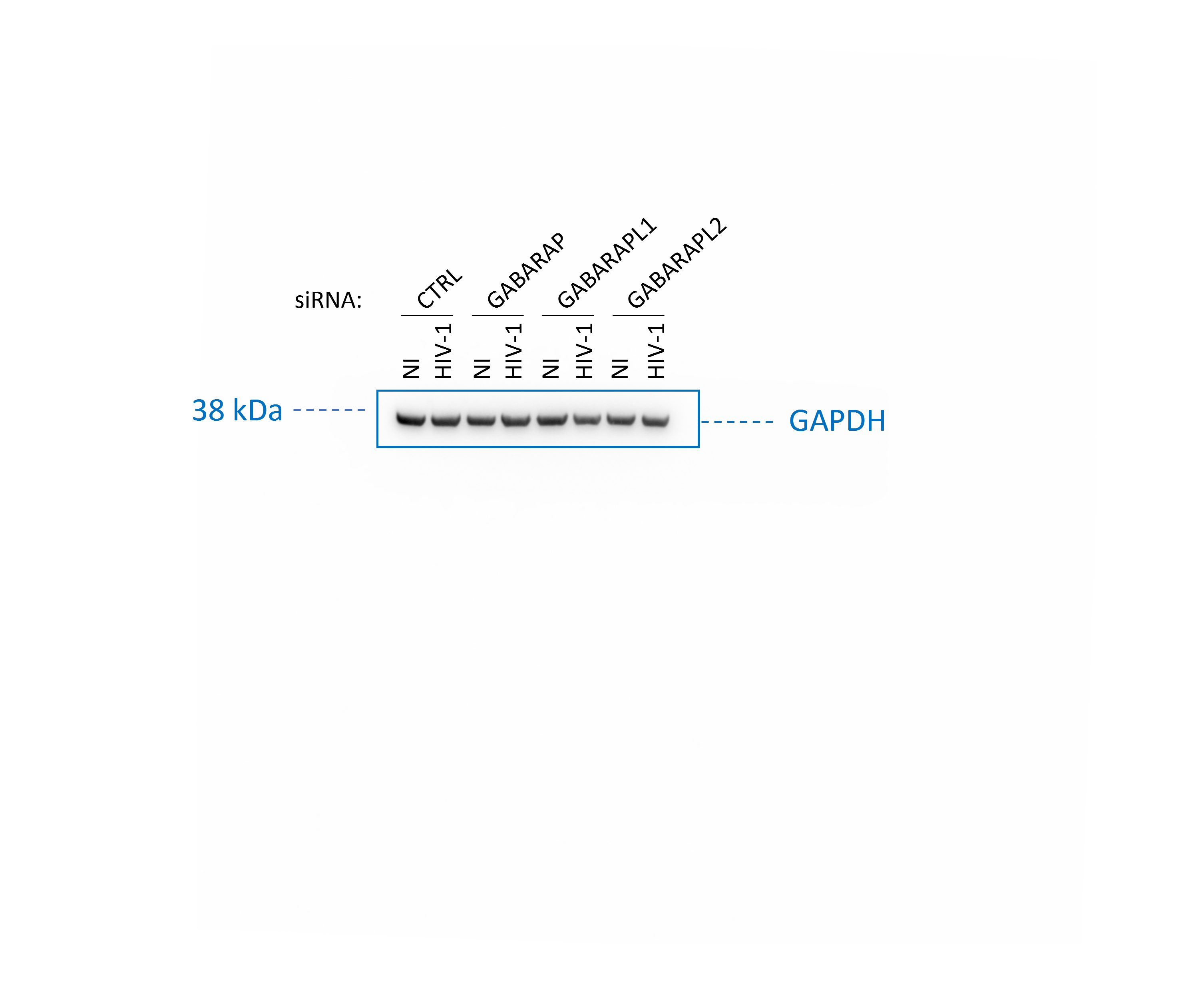

Supplement: Supplementary file 5 — Source data Fig. 3 [file 44319_2025_607_MOESM5_ESM.zip › Figure 3B/fig3B_GAPDH.tif]

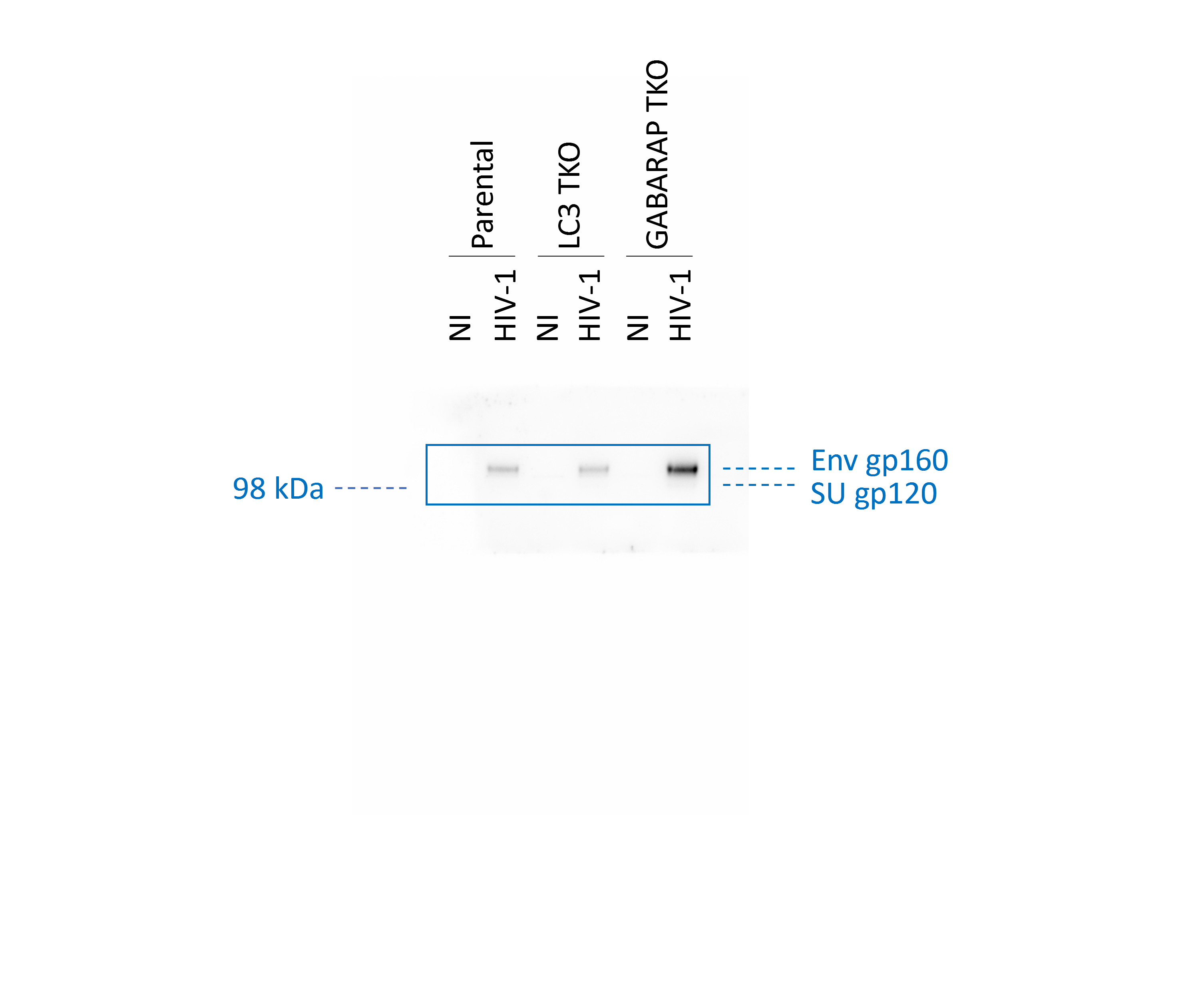

Supplement: Supplementary file 6 — Source data Fig. 4 [file 44319_2025_607_MOESM6_ESM.zip › Figure 4A/fig4A_Env_cell.tif]

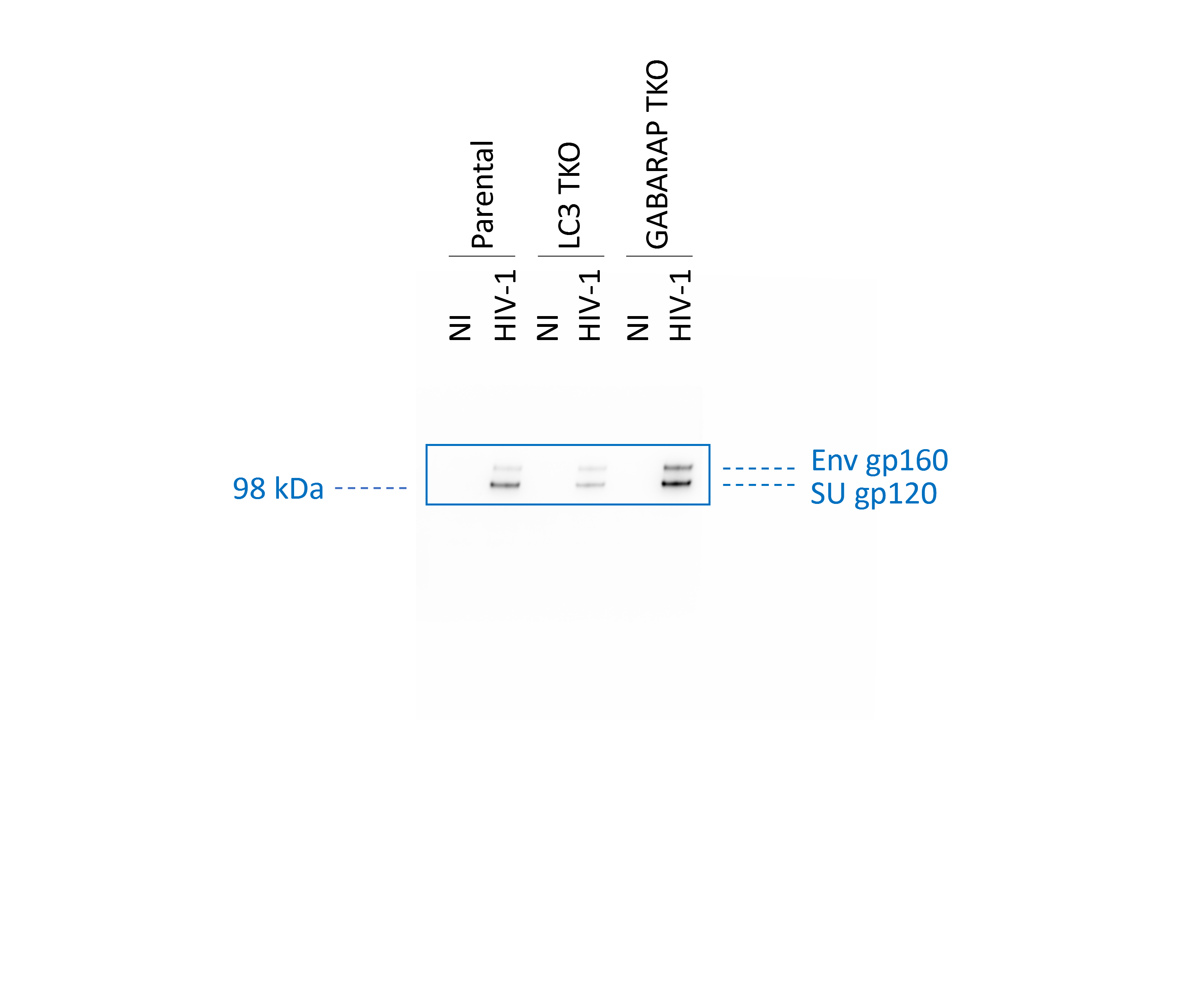

Supplement: Supplementary file 6 — Source data Fig. 4 [file 44319_2025_607_MOESM6_ESM.zip › Figure 4A/fig4A_Env_virion prep.tif]

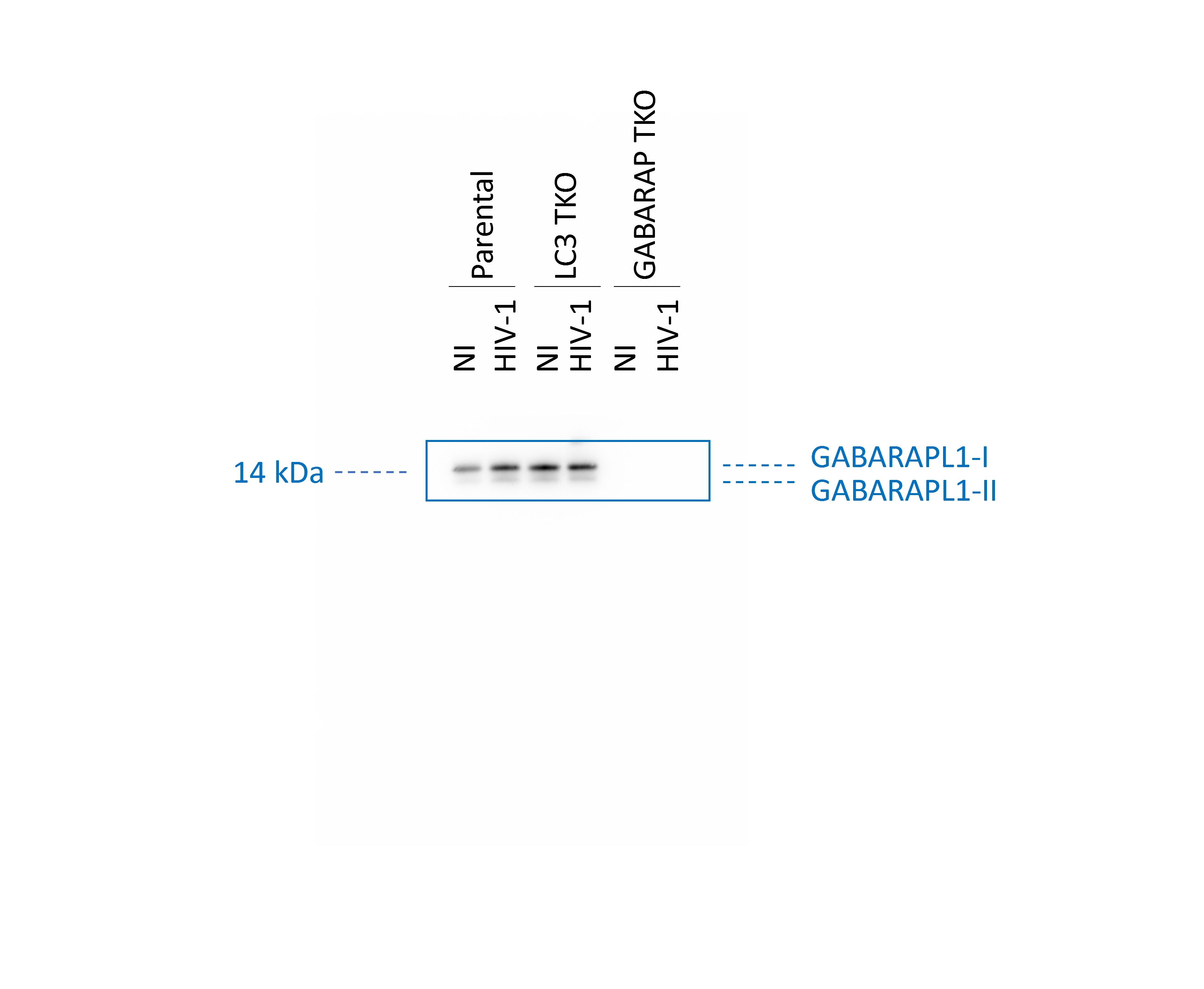

Supplement: Supplementary file 6 — Source data Fig. 4 [file 44319_2025_607_MOESM6_ESM.zip › Figure 4A/fig4A_GABARAP_cell.tif]

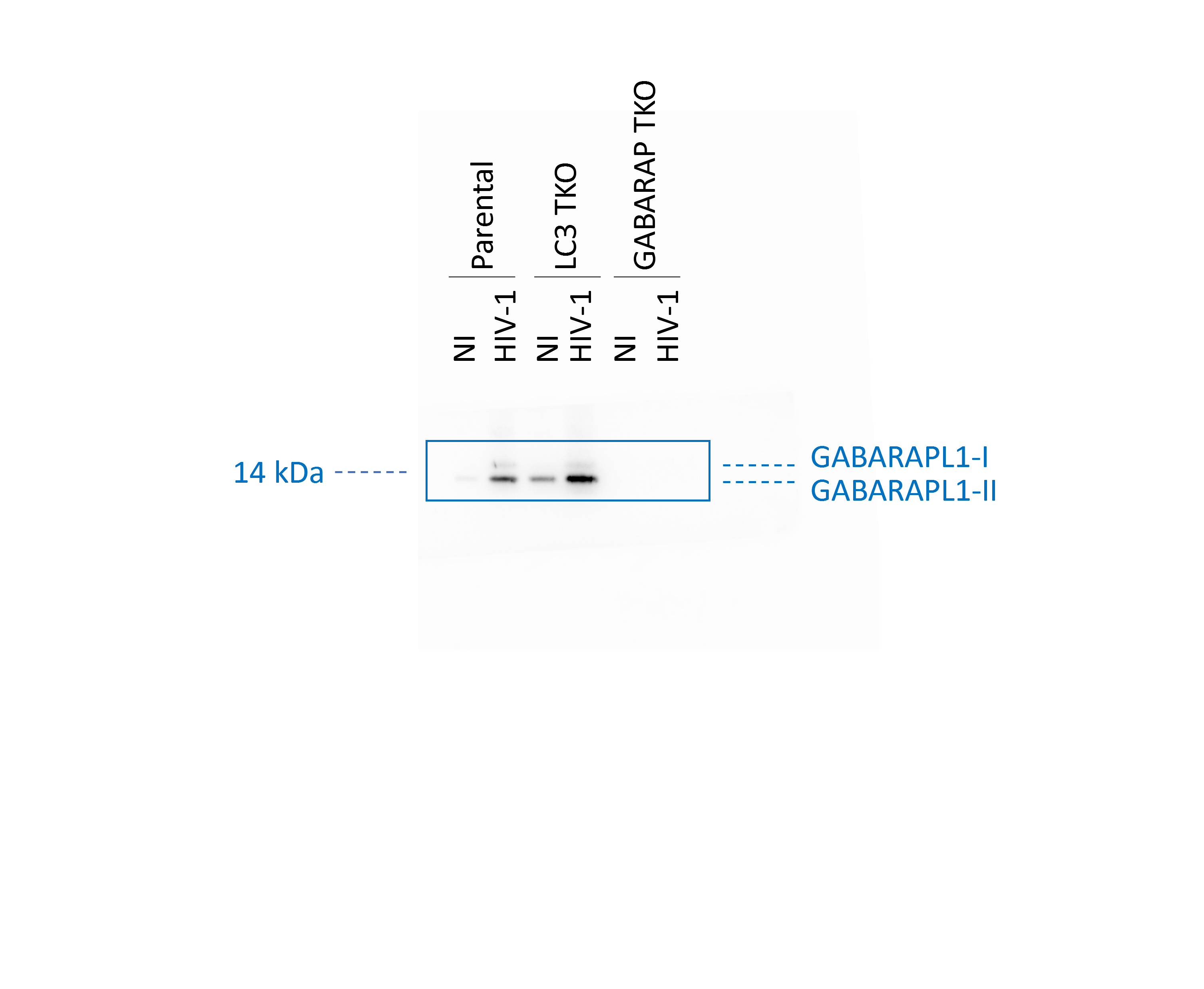

Supplement: Supplementary file 6 — Source data Fig. 4 [file 44319_2025_607_MOESM6_ESM.zip › Figure 4A/fig4A_GABARAP_virion prep.tif]

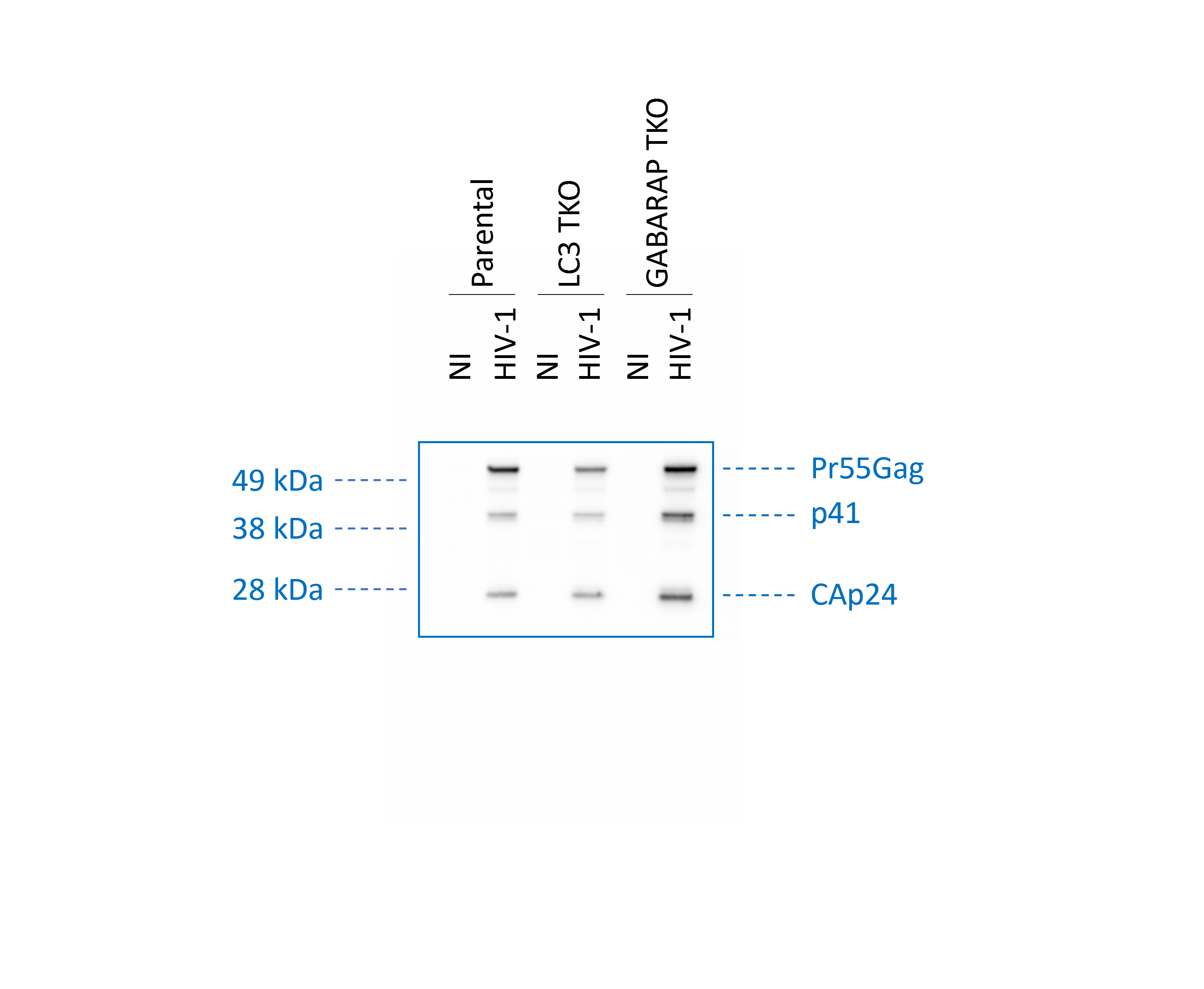

Supplement: Supplementary file 6 — Source data Fig. 4 [file 44319_2025_607_MOESM6_ESM.zip › Figure 4A/fig4A_Gag_cell.tif]

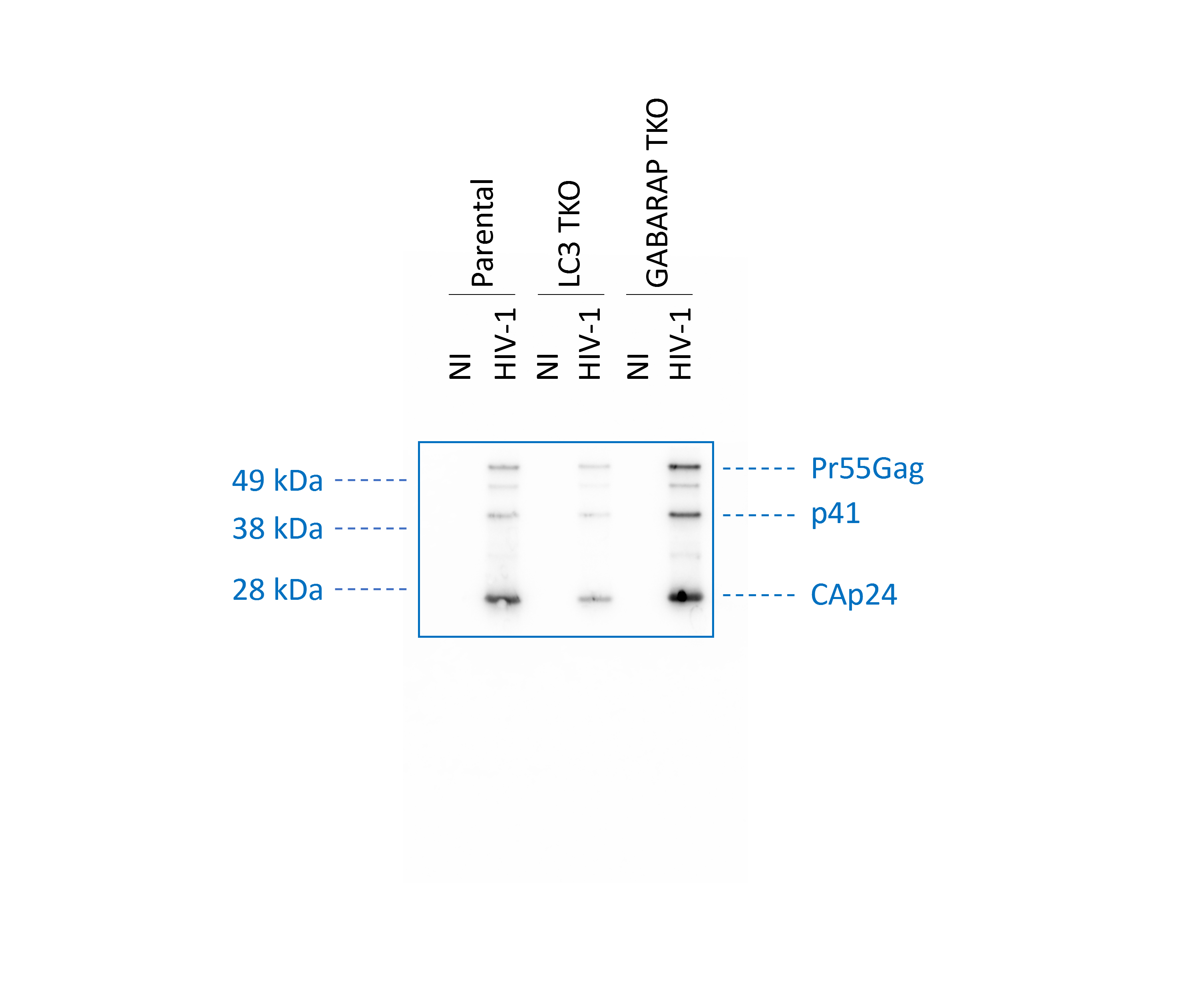

Supplement: Supplementary file 6 — Source data Fig. 4 [file 44319_2025_607_MOESM6_ESM.zip › Figure 4A/fig4A_Gag_virion prep.tif]

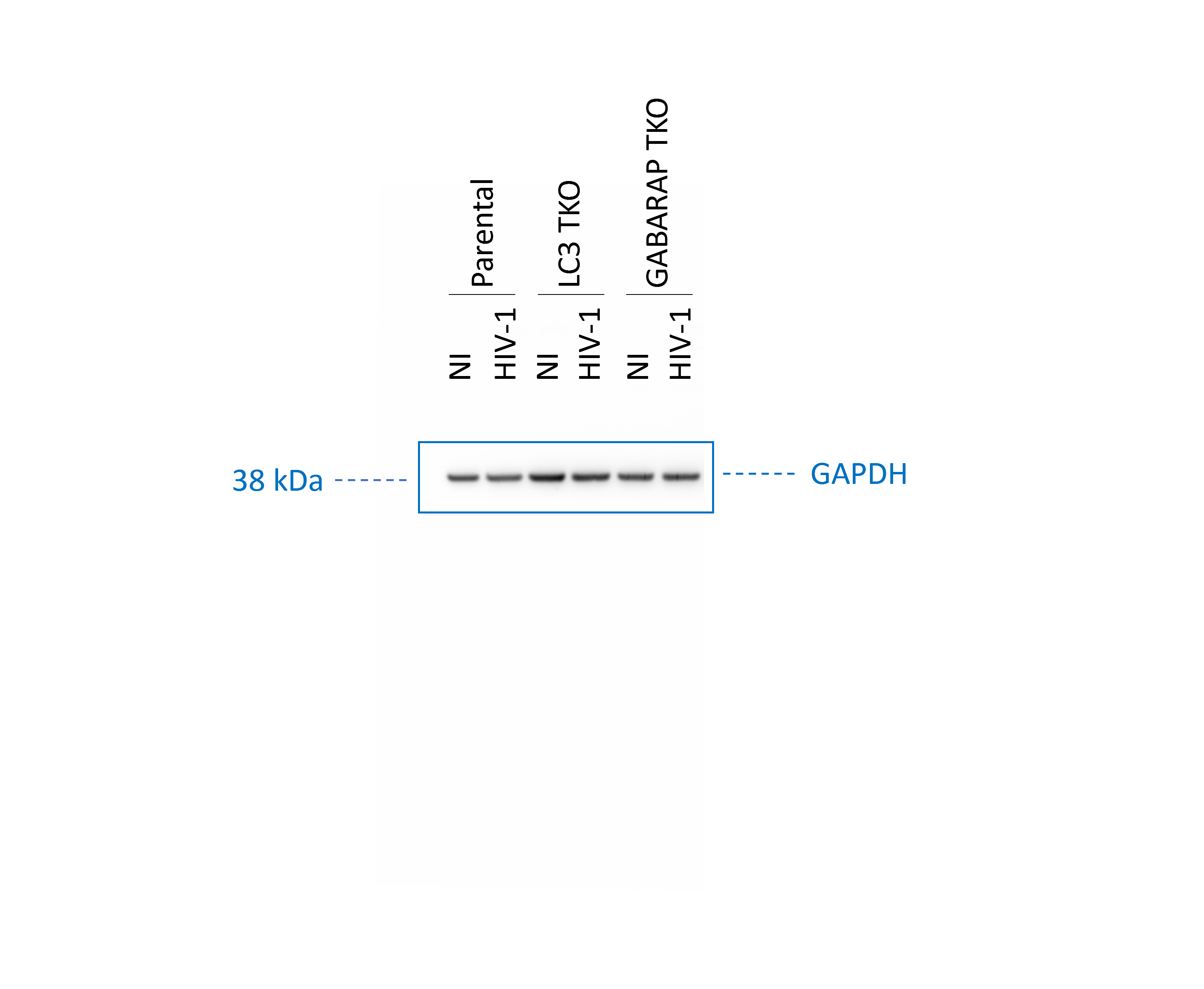

Supplement: Supplementary file 6 — Source data Fig. 4 [file 44319_2025_607_MOESM6_ESM.zip › Figure 4A/fig4A_GAPDH.tif]

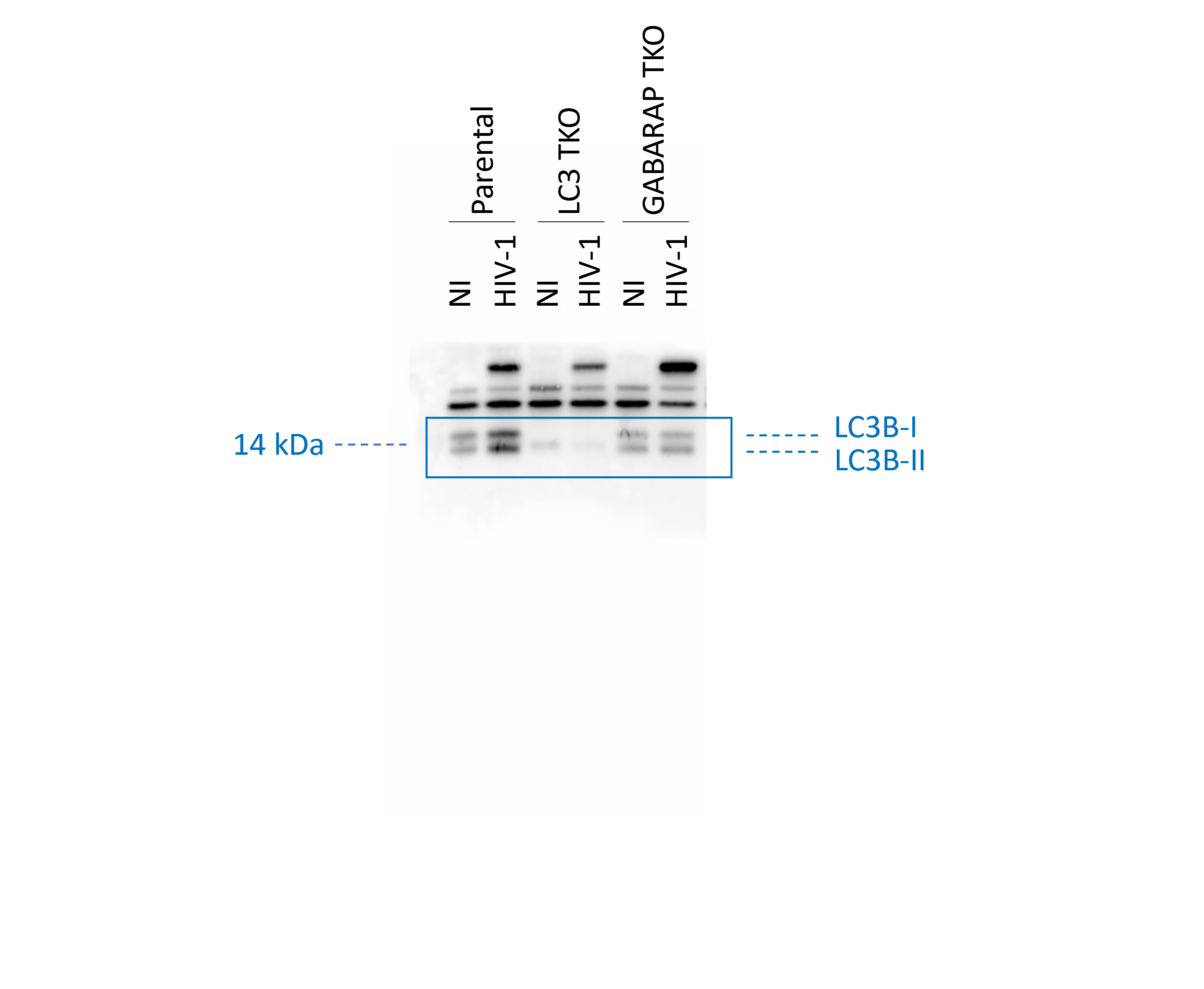

Supplement: Supplementary file 6 — Source data Fig. 4 [file 44319_2025_607_MOESM6_ESM.zip › Figure 4A/fig4A_LC3B_cell.tif]

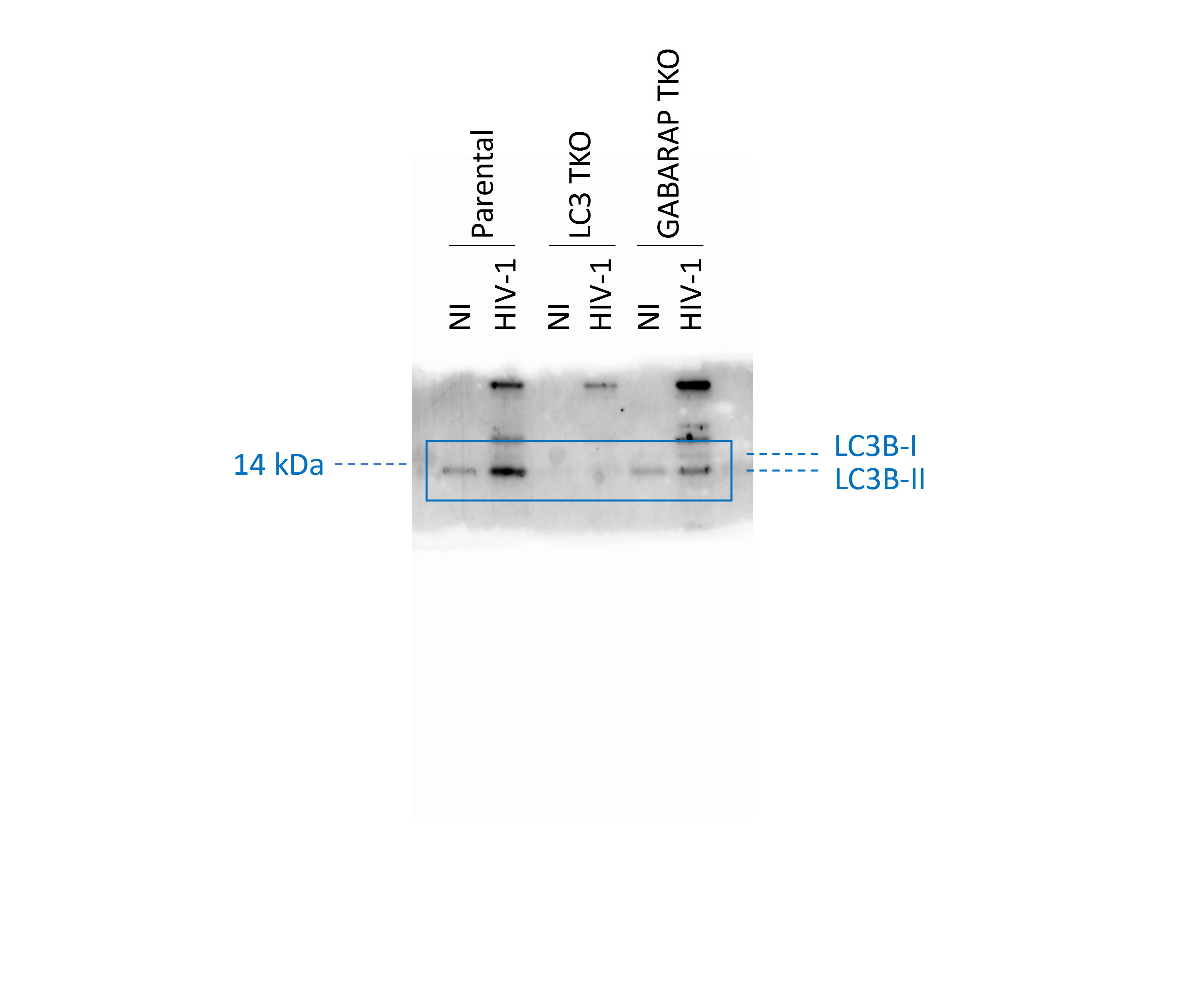

Supplement: Supplementary file 6 — Source data Fig. 4 [file 44319_2025_607_MOESM6_ESM.zip › Figure 4A/fig4A_LC3B_virion prep.tif]

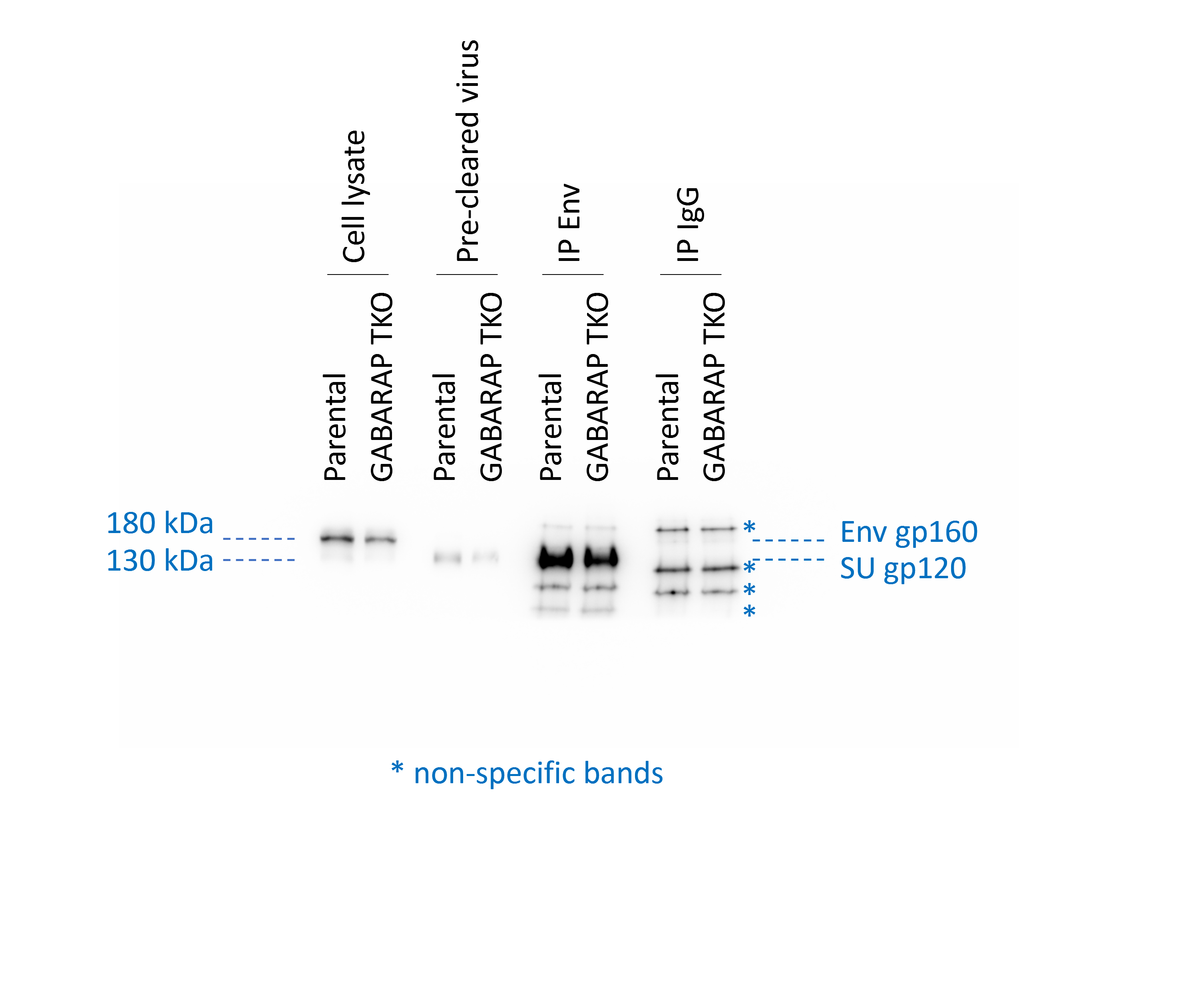

Supplement: Supplementary file 7 — Source data Fig. 5 [file 44319_2025_607_MOESM7_ESM.zip › Figure 5_New/Figure 5F/fig5F_Env.tif]

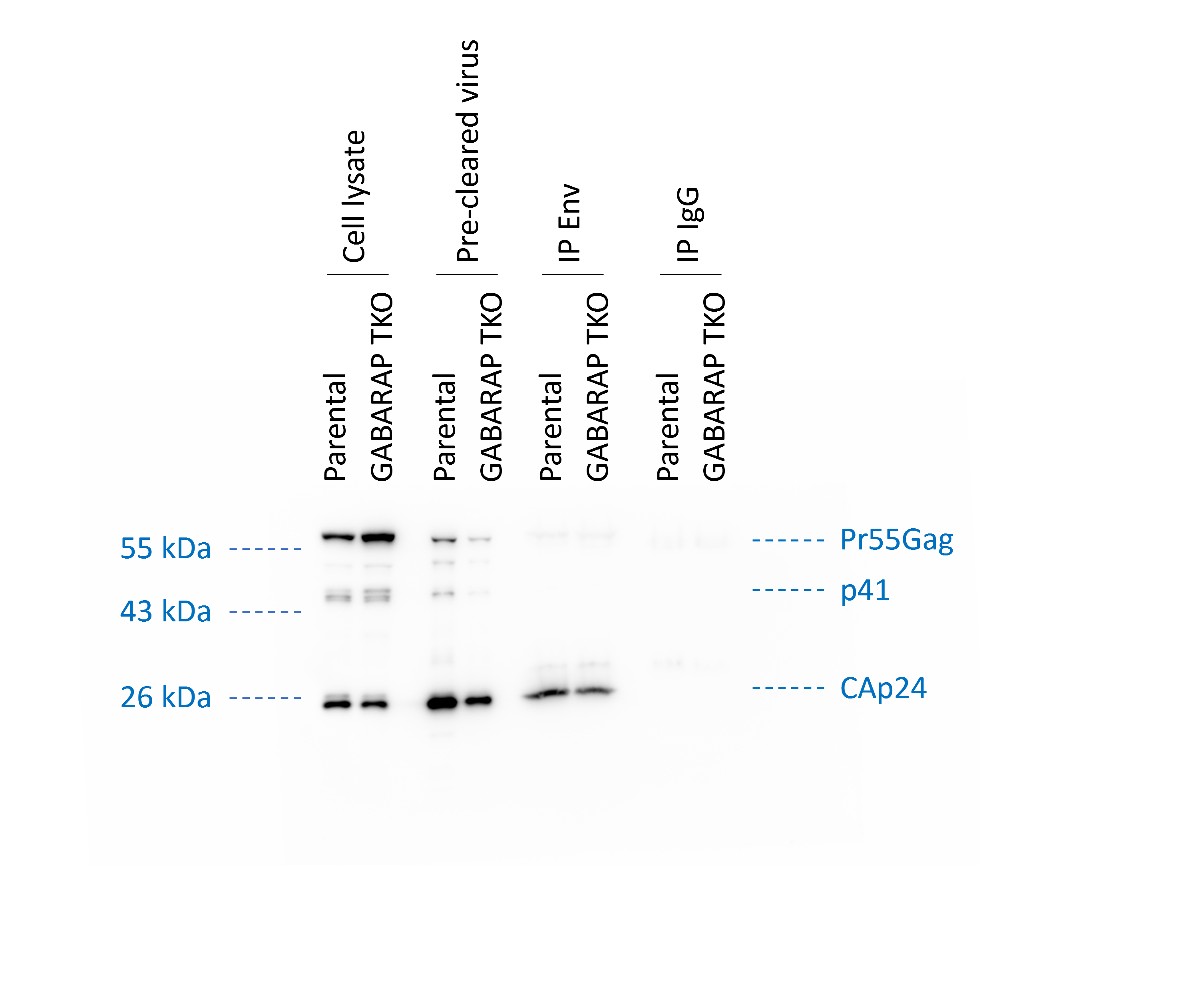

Supplement: Supplementary file 7 — Source data Fig. 5 [file 44319_2025_607_MOESM7_ESM.zip › Figure 5_New/Figure 5F/fig5F_Gag.tif]

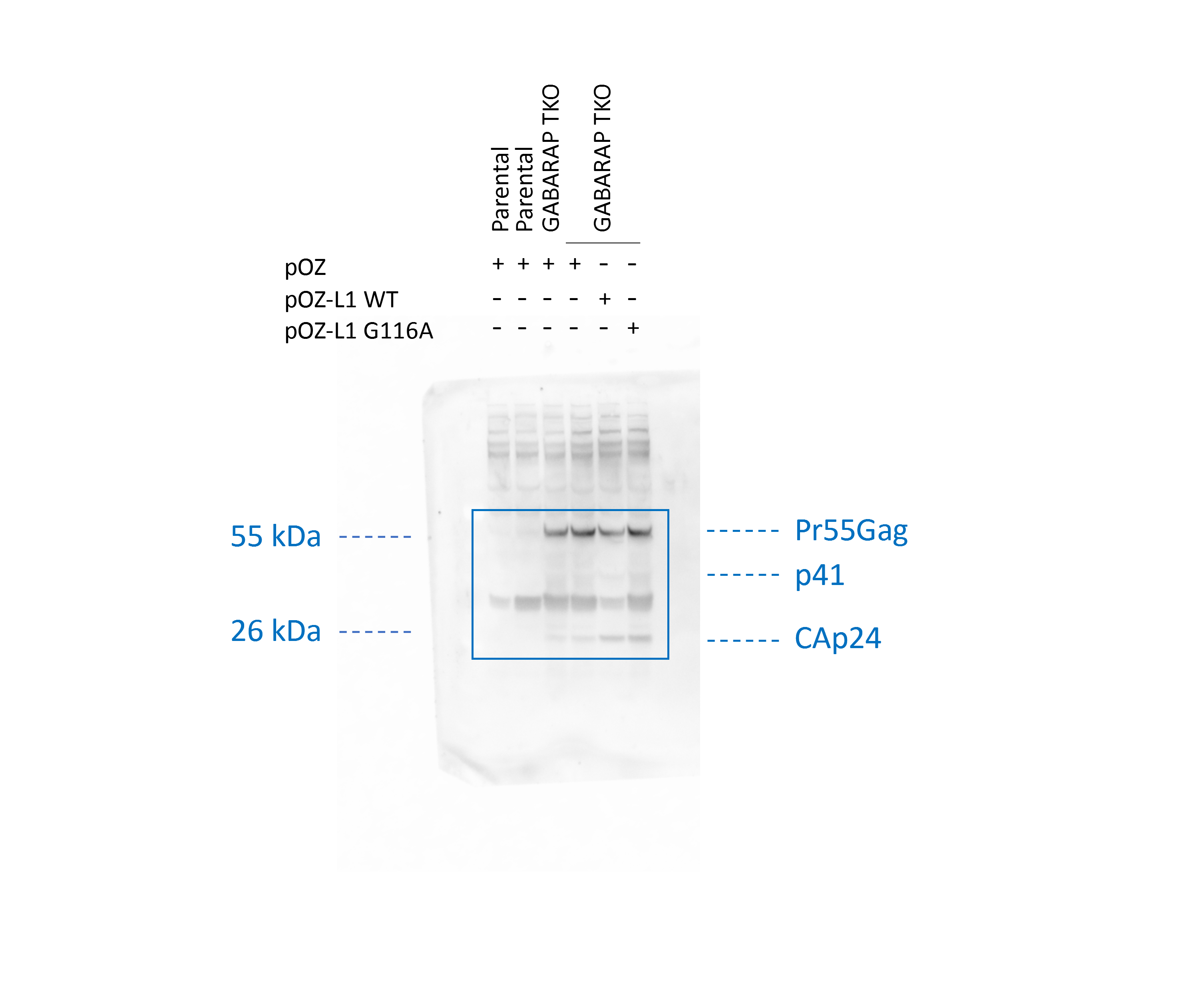

Supplement: Supplementary file 7 — Source data Fig. 5 [file 44319_2025_607_MOESM7_ESM.zip › Figure 5_New/Figure 5G/fig5G_Gag_cell.tif]

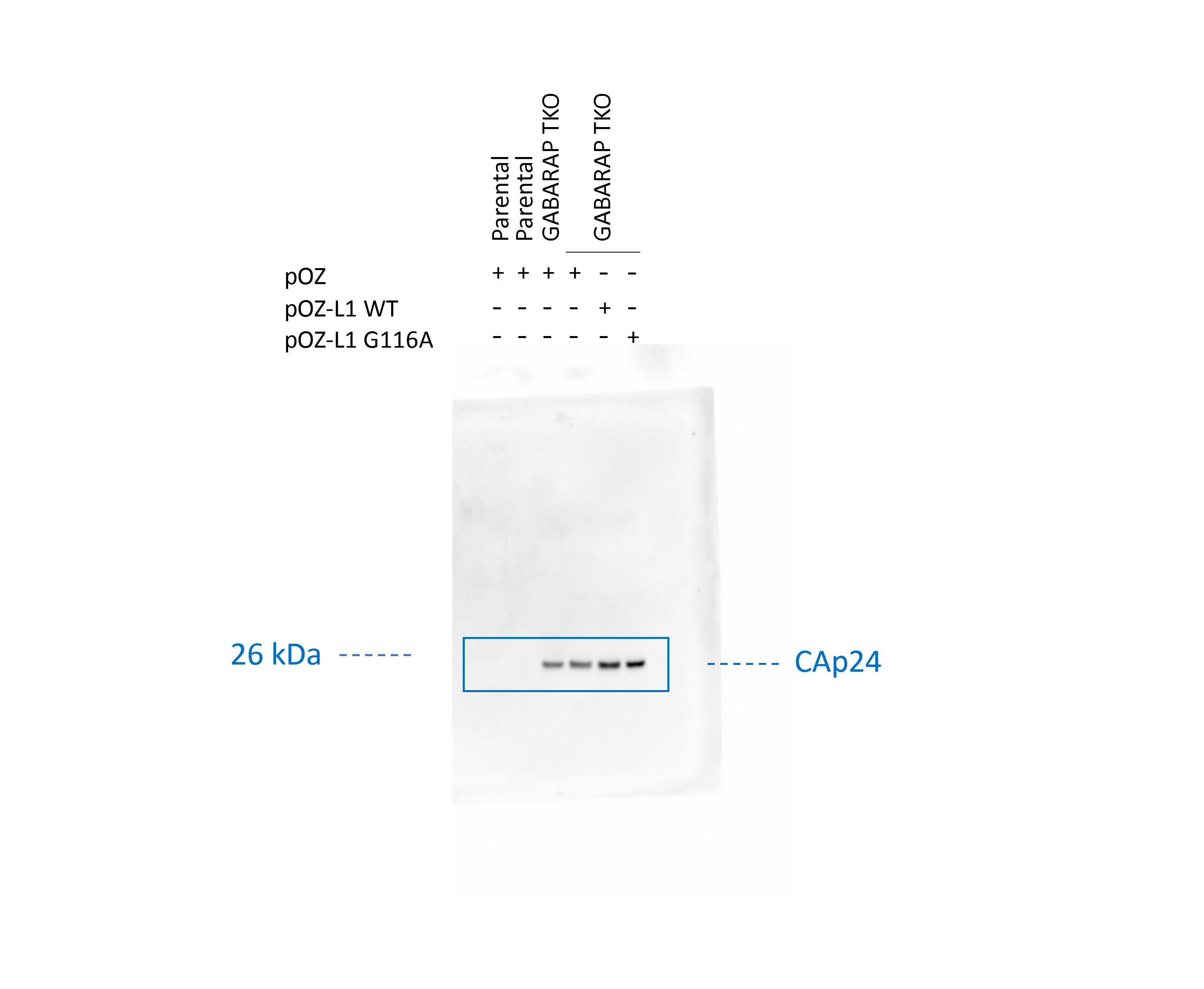

Supplement: Supplementary file 7 — Source data Fig. 5 [file 44319_2025_607_MOESM7_ESM.zip › Figure 5_New/Figure 5G/fig5G_Gag_virion preparation.tif]

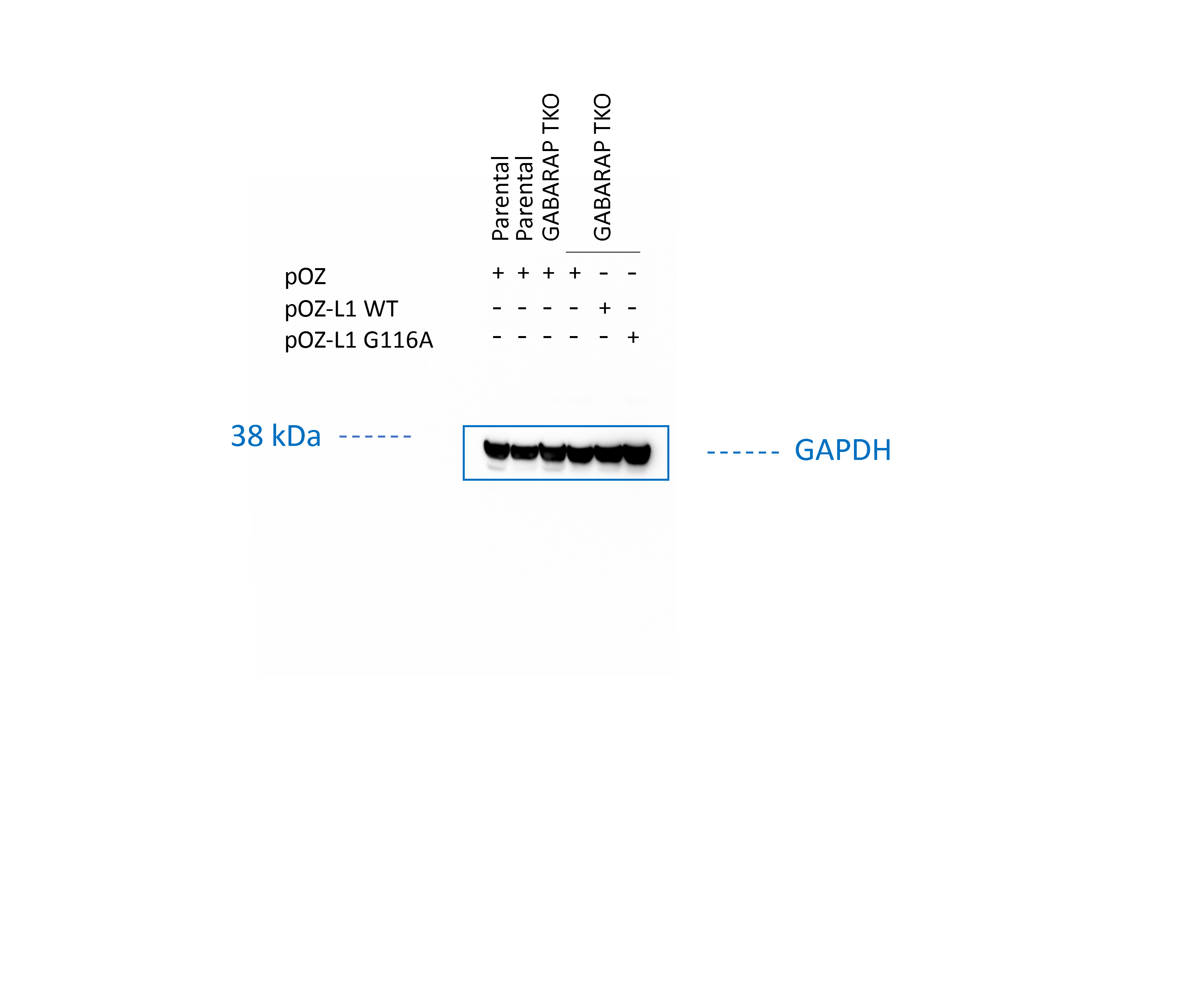

Supplement: Supplementary file 7 — Source data Fig. 5 [file 44319_2025_607_MOESM7_ESM.zip › Figure 5_New/Figure 5G/fig5G_GAPDH.tif]

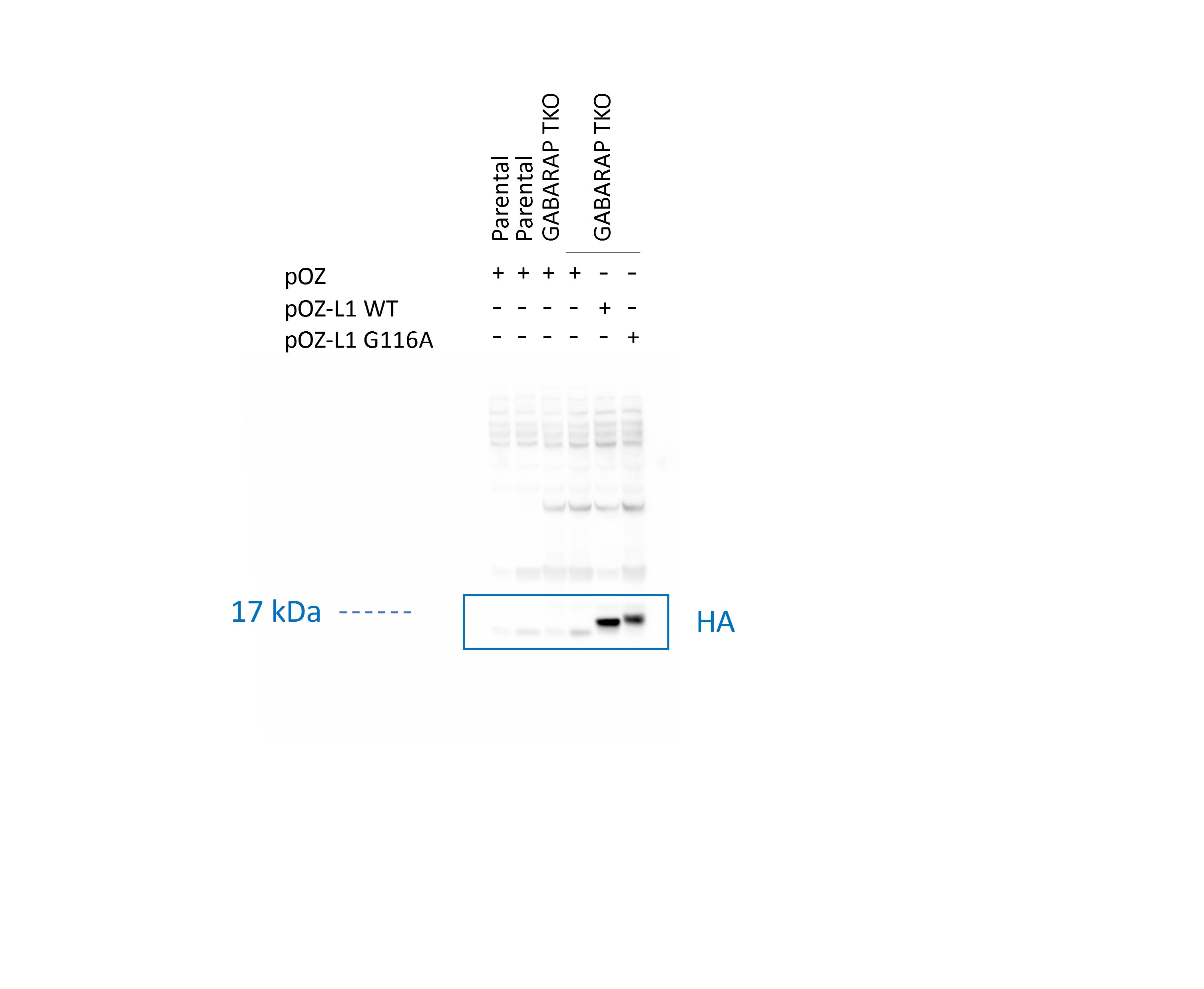

Supplement: Supplementary file 7 — Source data Fig. 5 [file 44319_2025_607_MOESM7_ESM.zip › Figure 5_New/Figure 5G/fig5G_HA.tif]

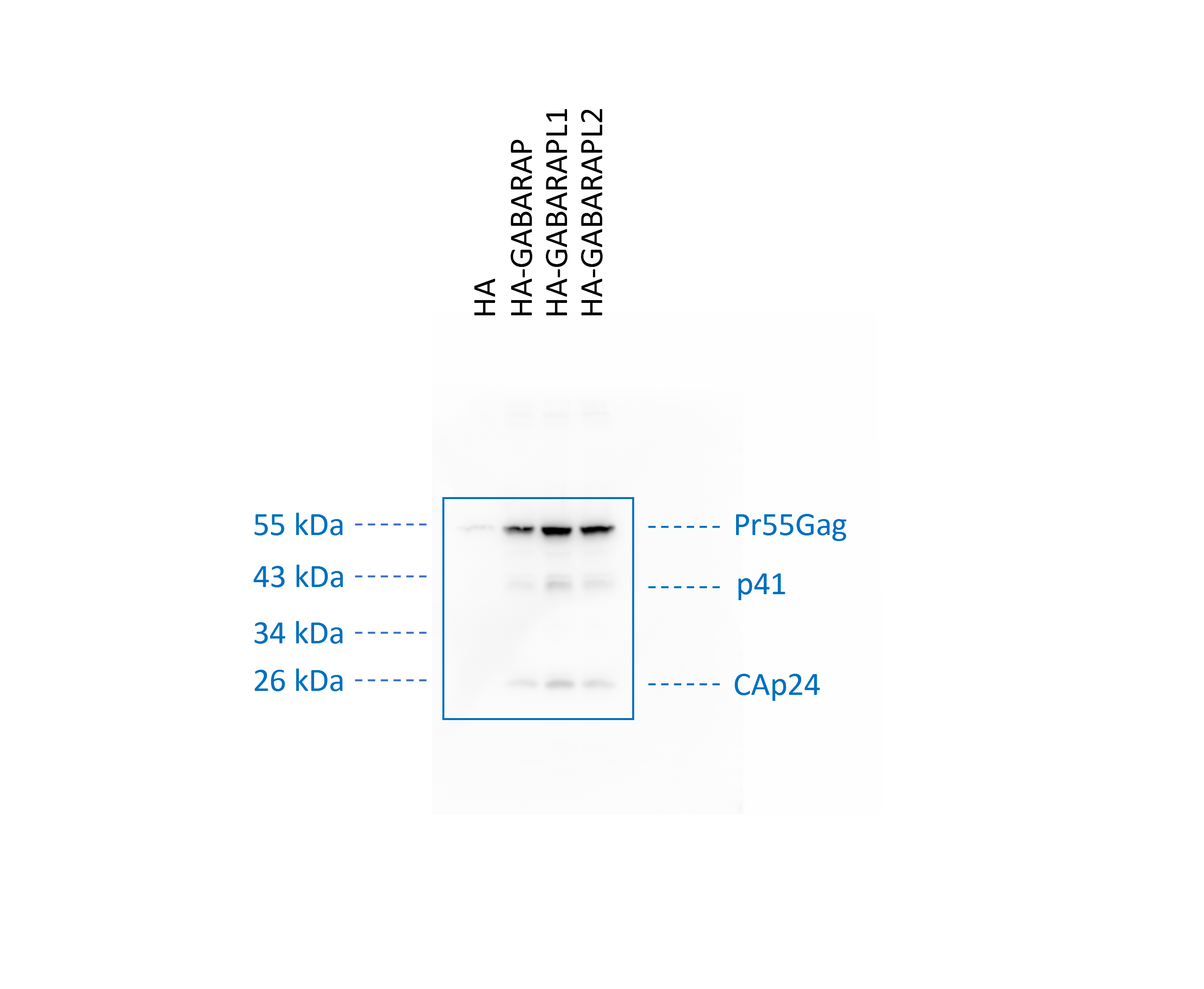

Supplement: Supplementary file 8 — Source data Fig. 6 [file 44319_2025_607_MOESM8_ESM.zip › Figure 6A/fig6A_Gag_HA-trap.tif]

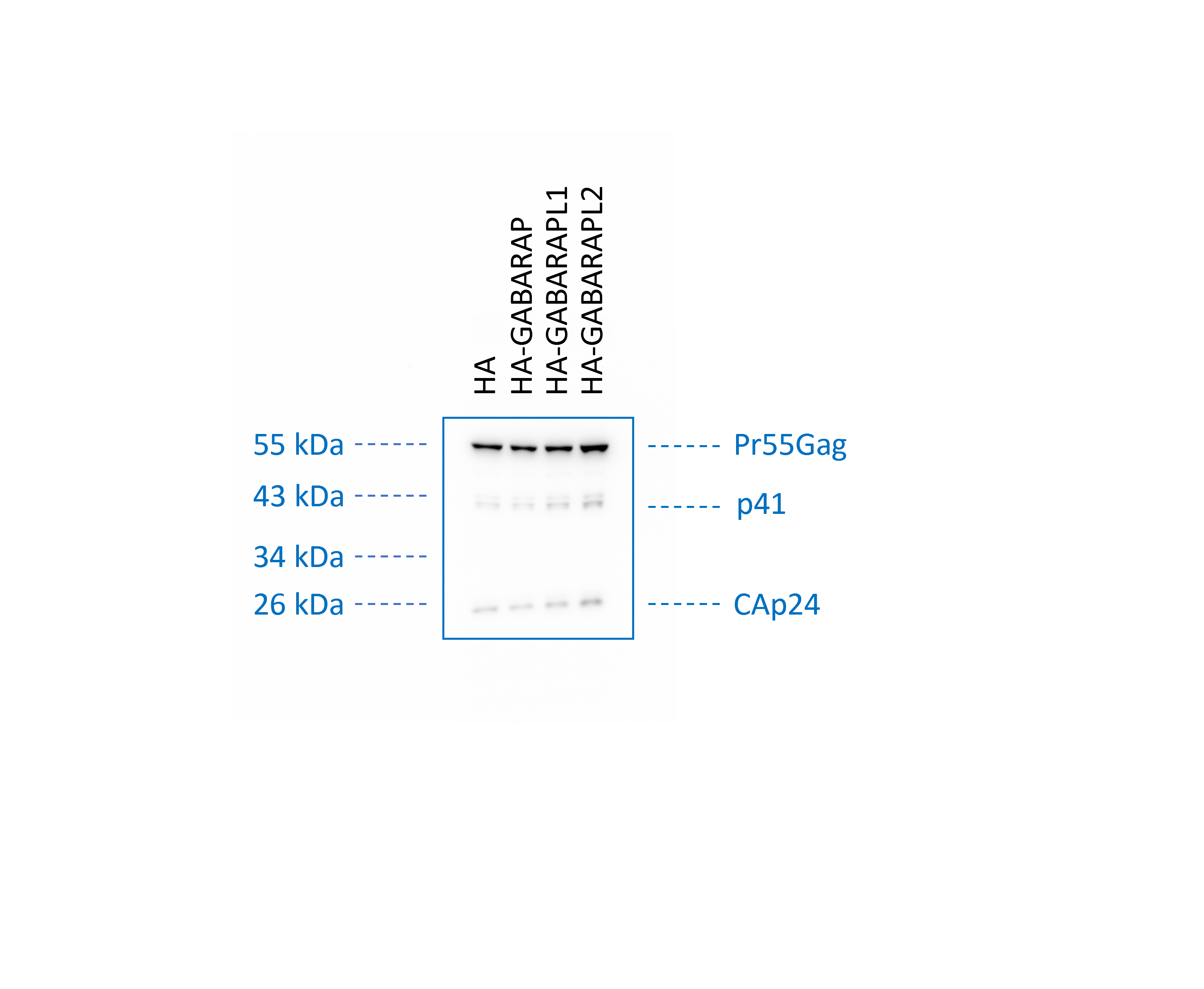

Supplement: Supplementary file 8 — Source data Fig. 6 [file 44319_2025_607_MOESM8_ESM.zip › Figure 6A/fig6A_Gag_input.tif]

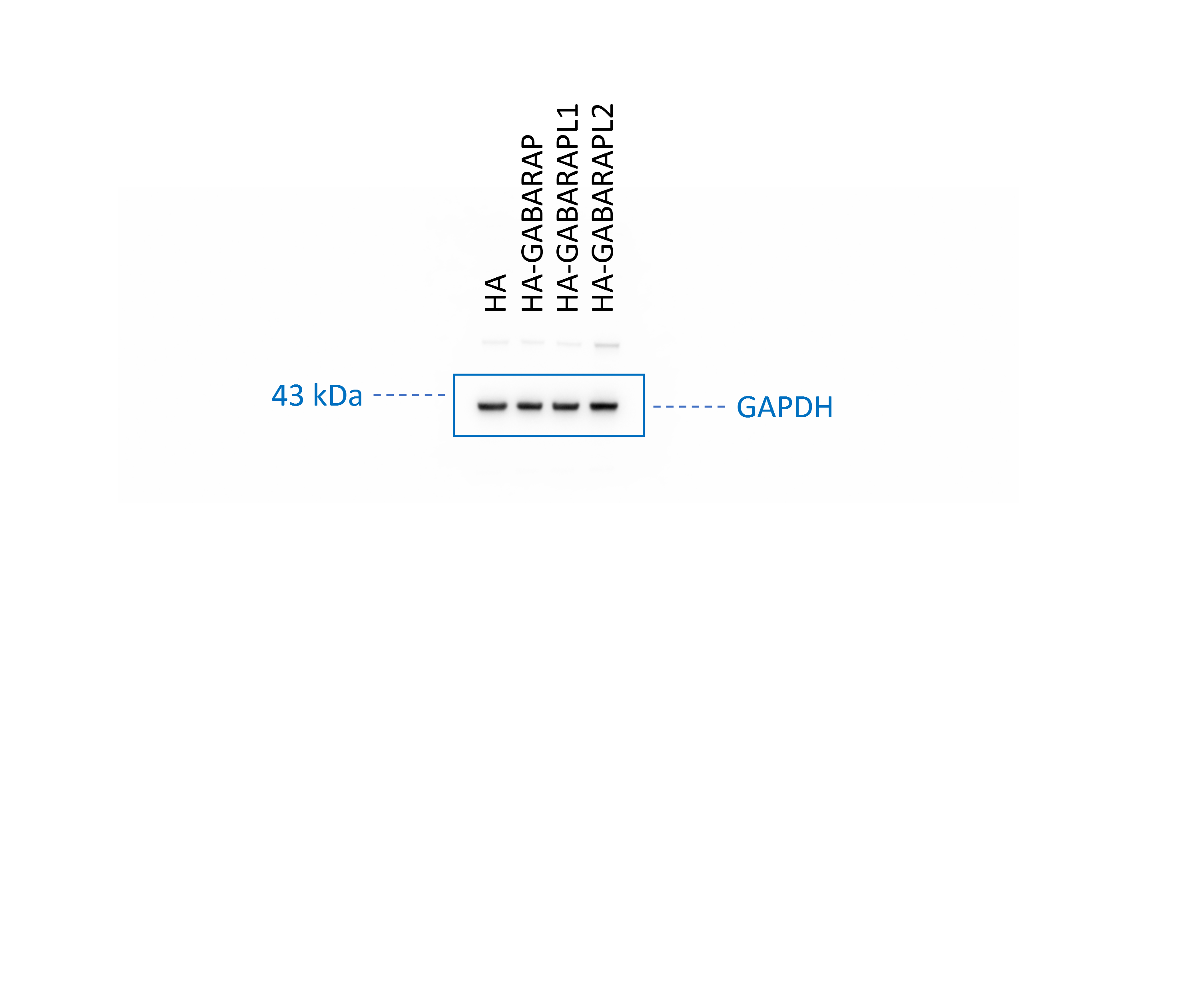

Supplement: Supplementary file 8 — Source data Fig. 6 [file 44319_2025_607_MOESM8_ESM.zip › Figure 6A/fig6A_GAPDH_input.tif]

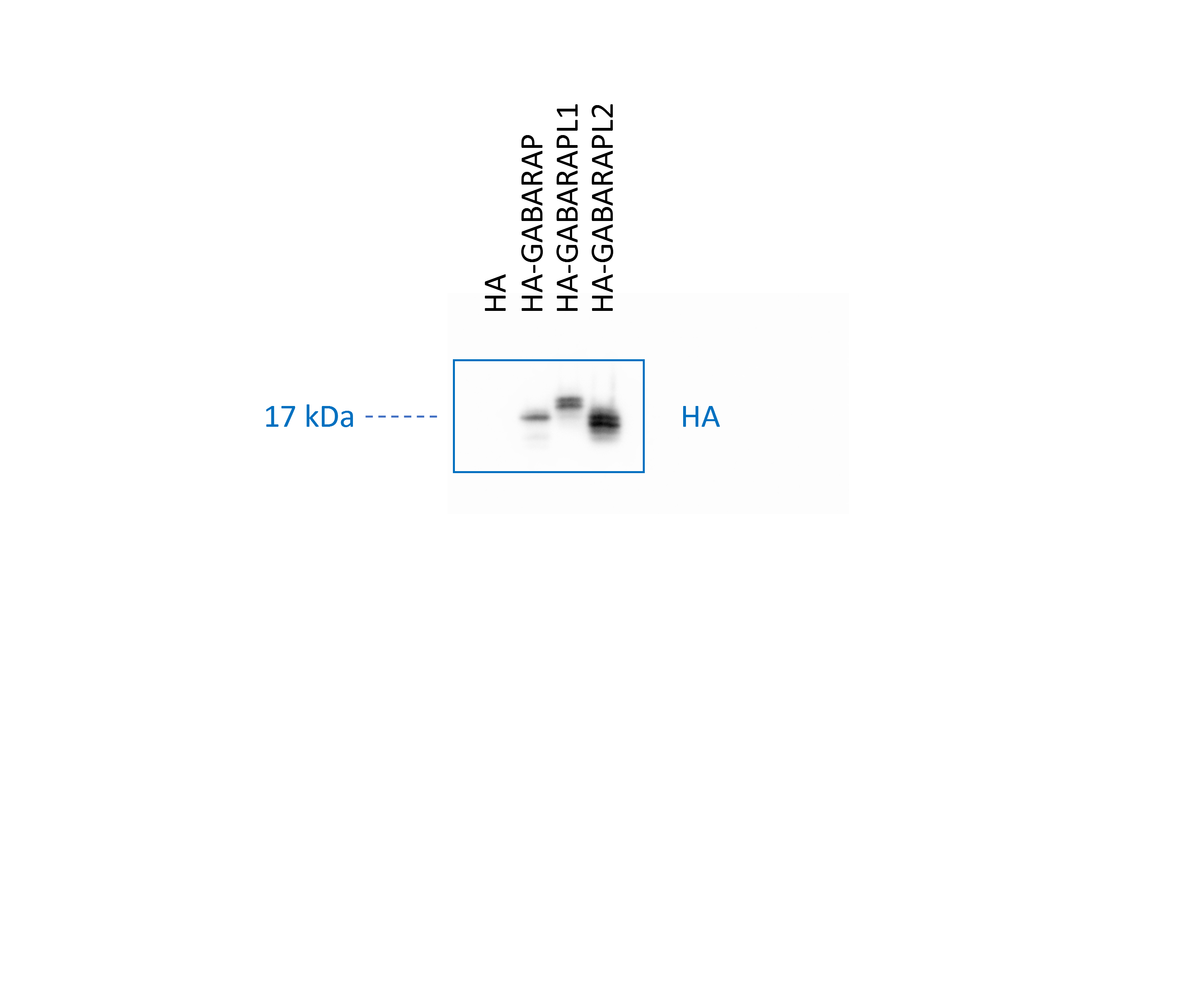

Supplement: Supplementary file 8 — Source data Fig. 6 [file 44319_2025_607_MOESM8_ESM.zip › Figure 6A/fig6A_HA_HA-trap.tif]

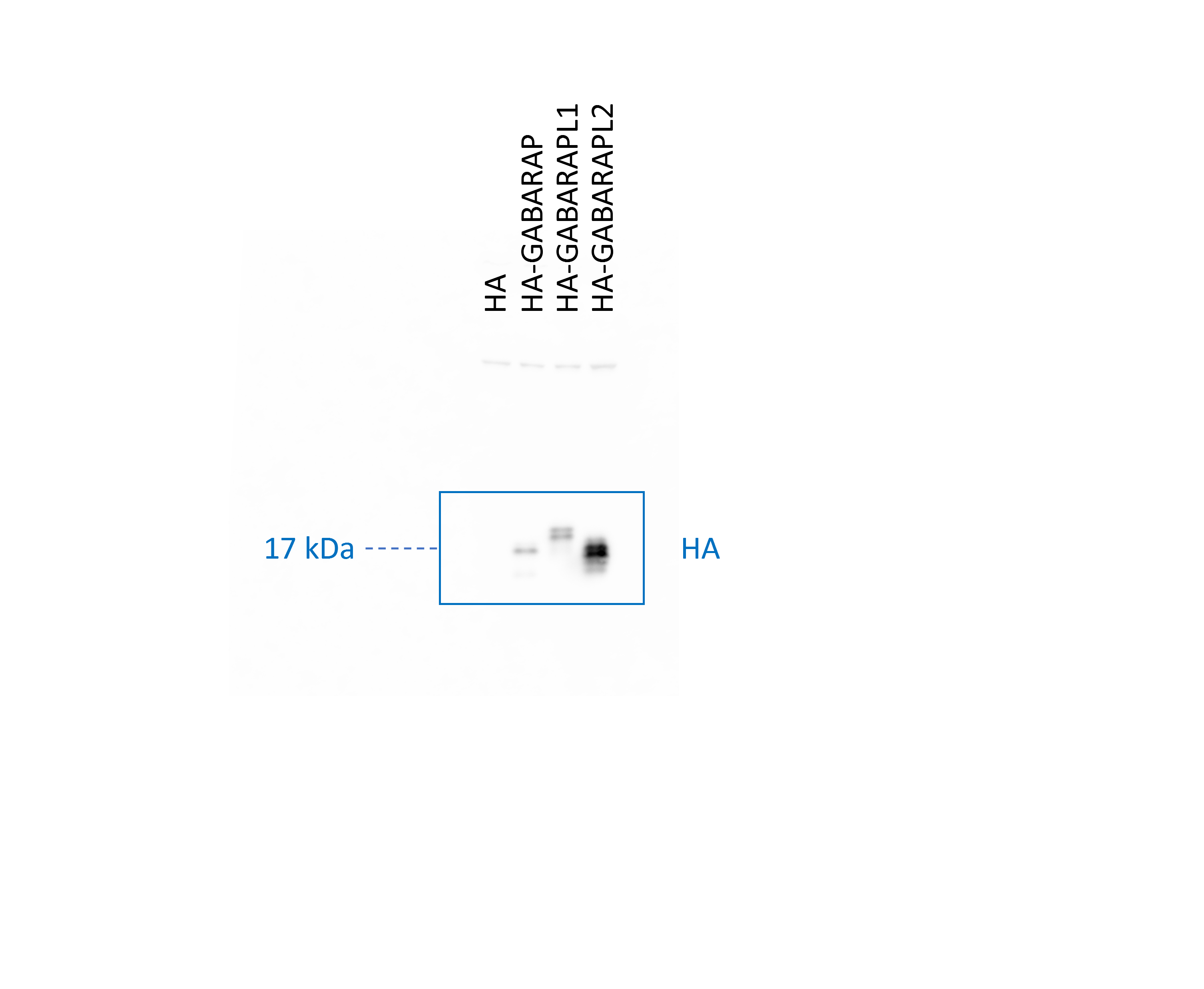

Supplement: Supplementary file 8 — Source data Fig. 6 [file 44319_2025_607_MOESM8_ESM.zip › Figure 6A/fig6A_HA_input.tif]

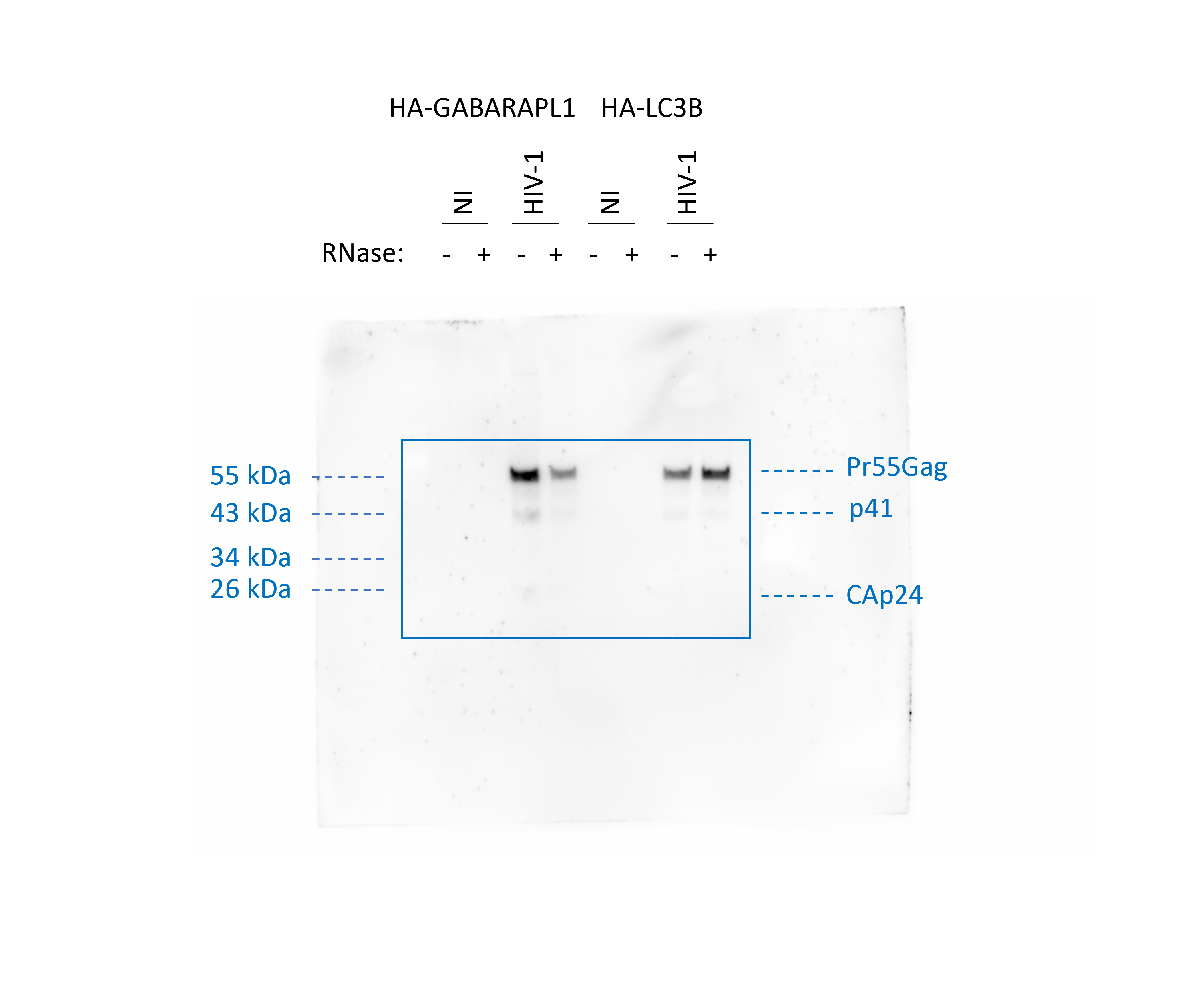

Supplement: Supplementary file 8 — Source data Fig. 6 [file 44319_2025_607_MOESM8_ESM.zip › Figure 6B/fig6B_Gag_HA-trap.tif]

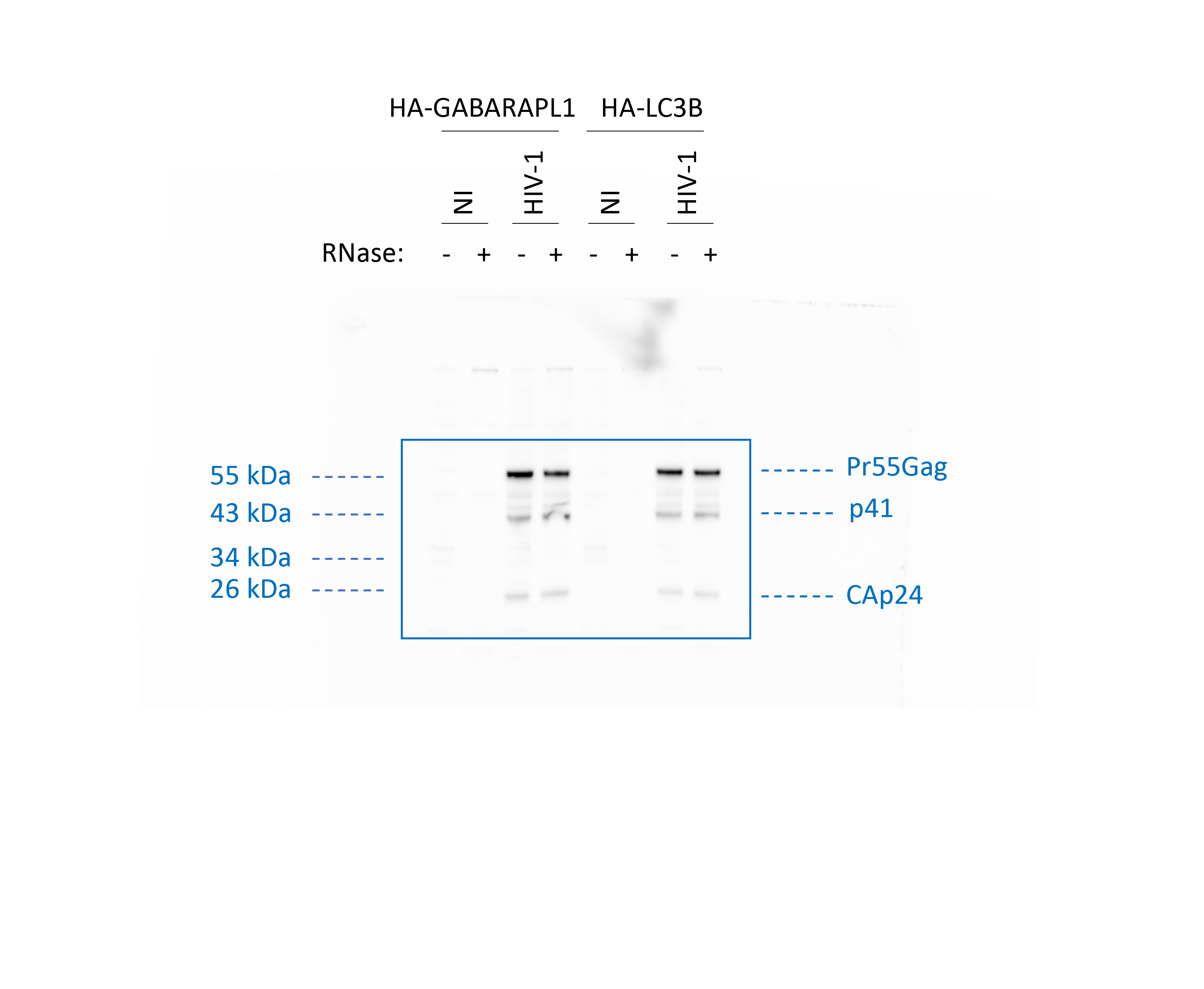

Supplement: Supplementary file 8 — Source data Fig. 6 [file 44319_2025_607_MOESM8_ESM.zip › Figure 6B/fig6B_Gag_input.tif]

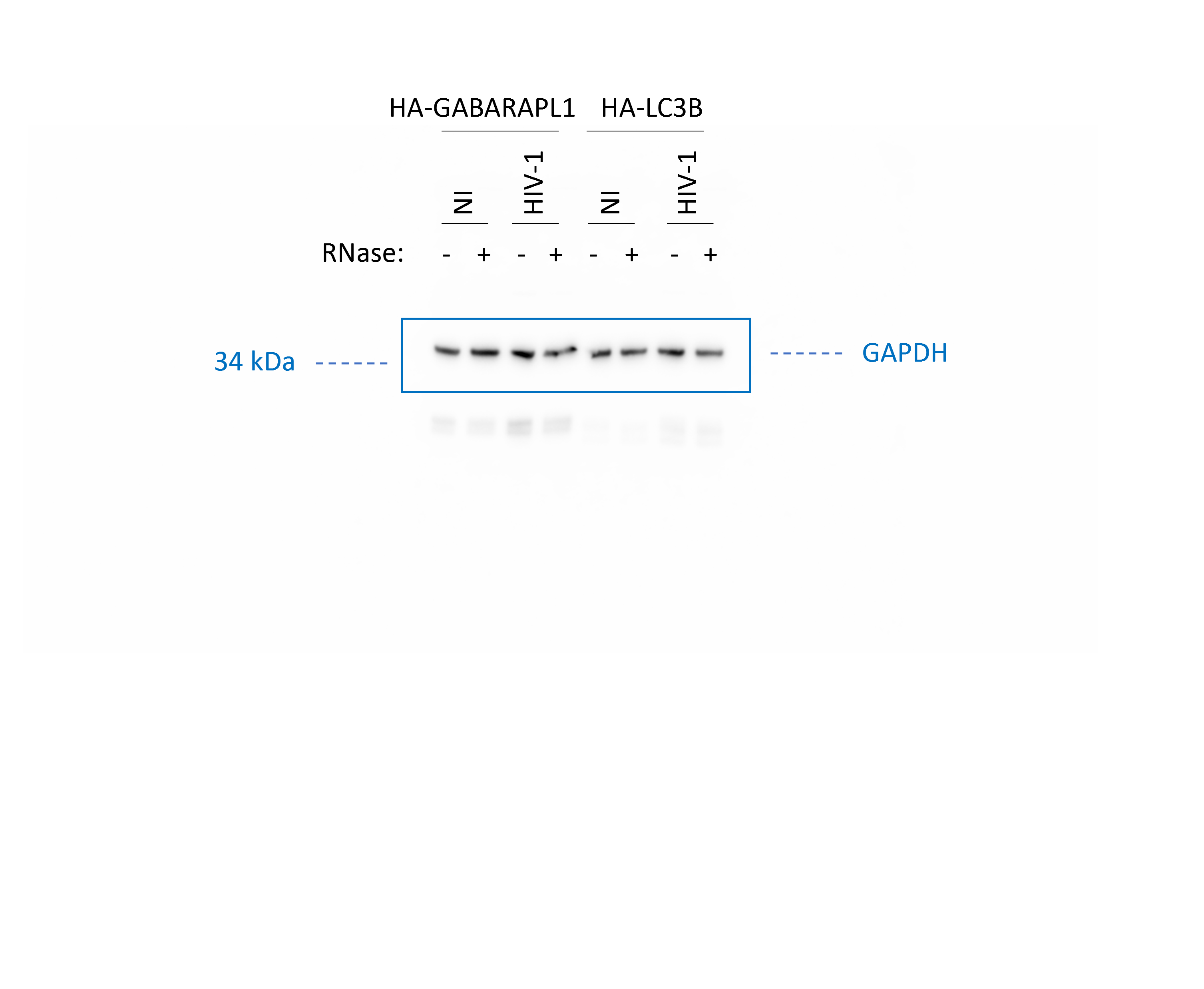

Supplement: Supplementary file 8 — Source data Fig. 6 [file 44319_2025_607_MOESM8_ESM.zip › Figure 6B/fig6B_GAPDH_input.tif]

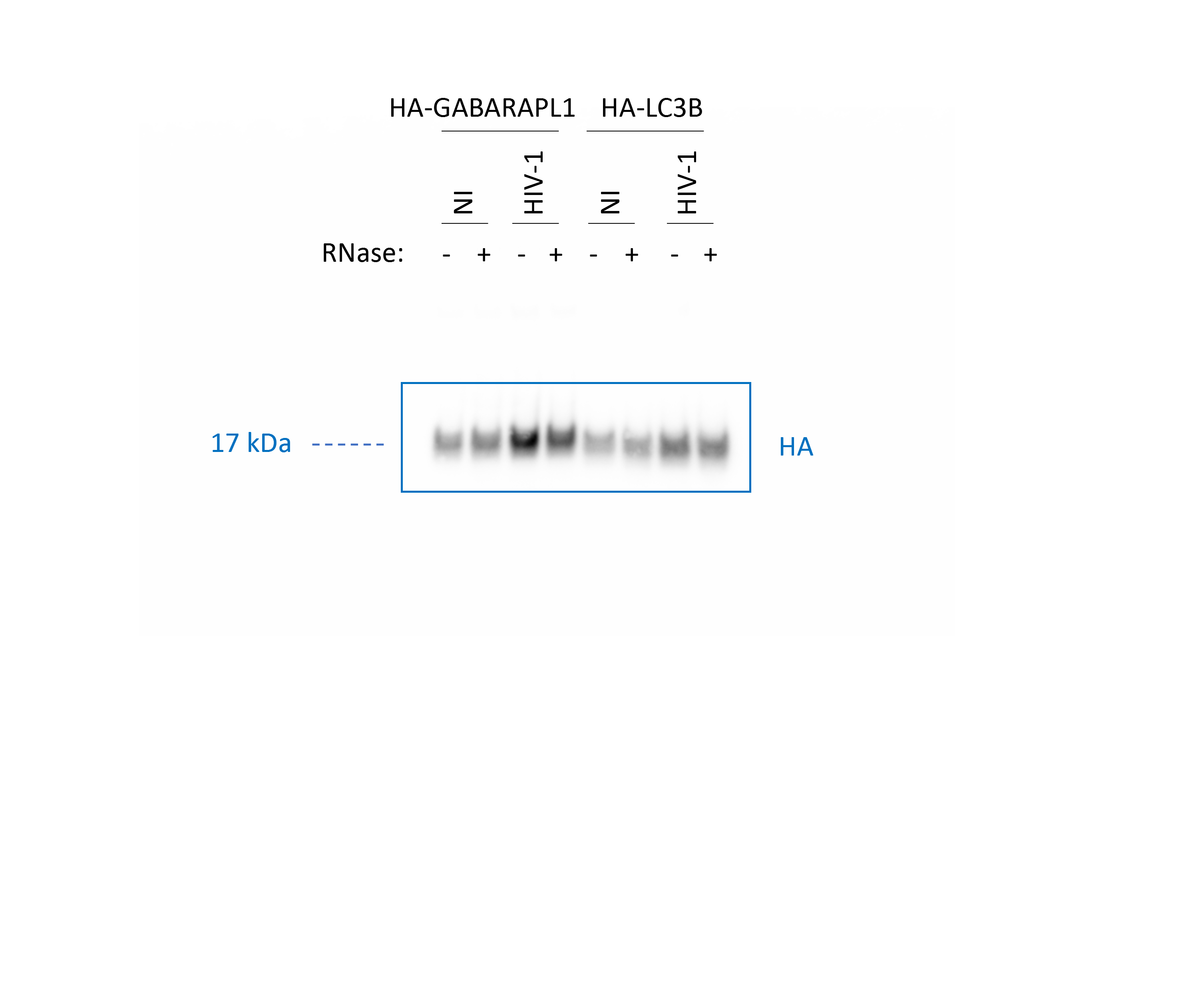

Supplement: Supplementary file 8 — Source data Fig. 6 [file 44319_2025_607_MOESM8_ESM.zip › Figure 6B/fig6B_HA_HA-trap.tif]

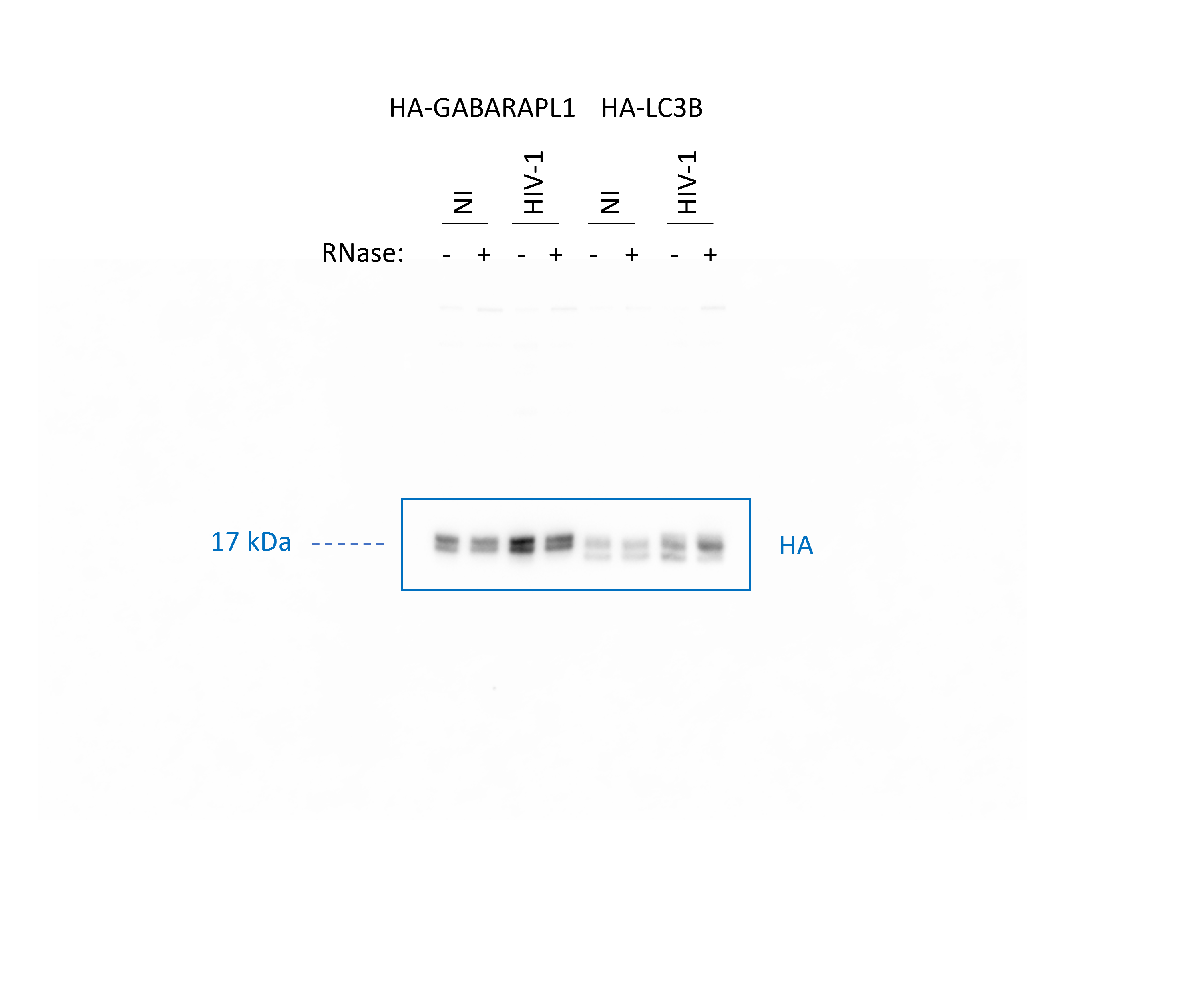

Supplement: Supplementary file 8 — Source data Fig. 6 [file 44319_2025_607_MOESM8_ESM.zip › Figure 6B/fig6B_HA_input.tif]

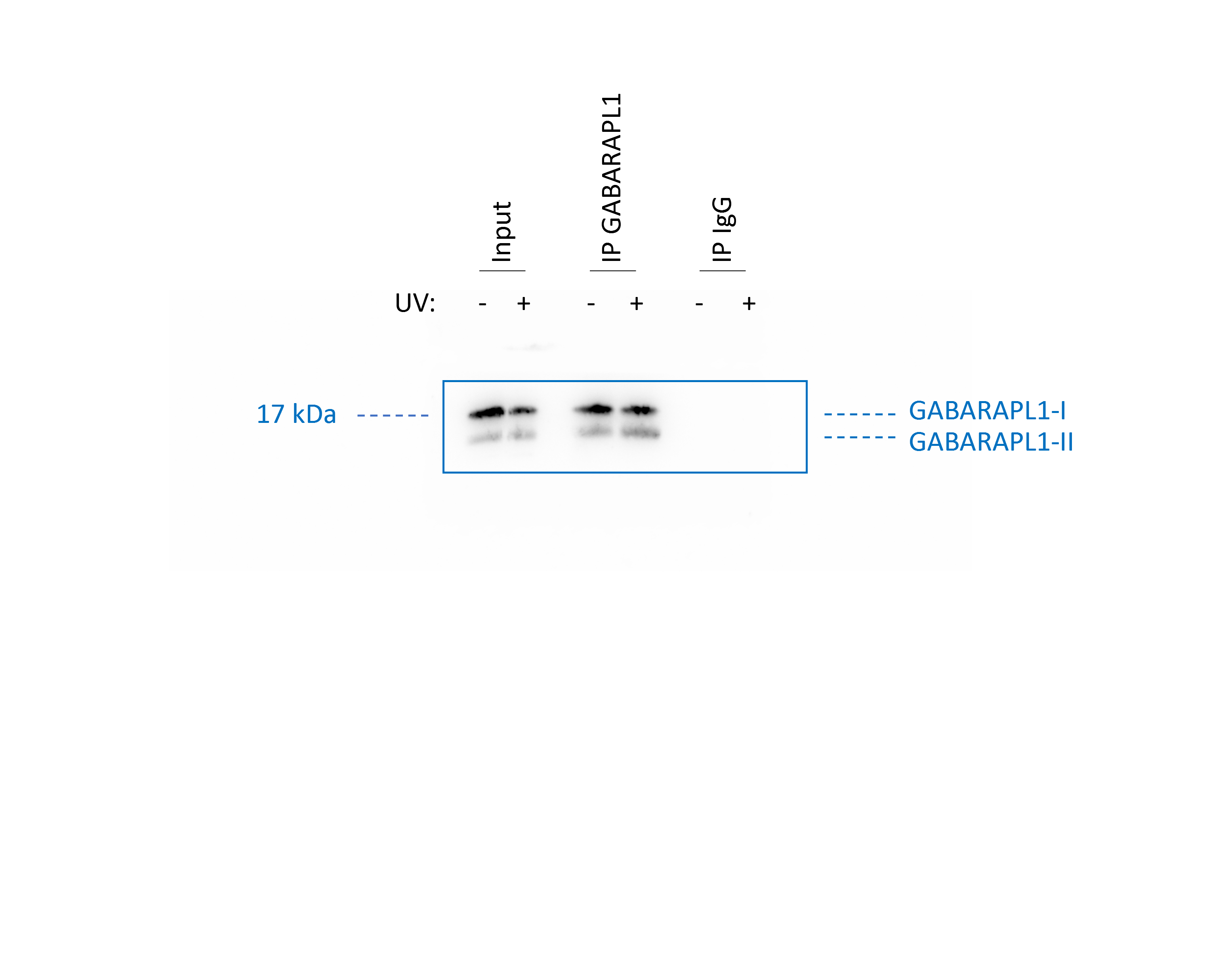

Supplement: Supplementary file 8 — Source data Fig. 6 [file 44319_2025_607_MOESM8_ESM.zip › Figure 6E/fig6D_GABARAPL1.tif]

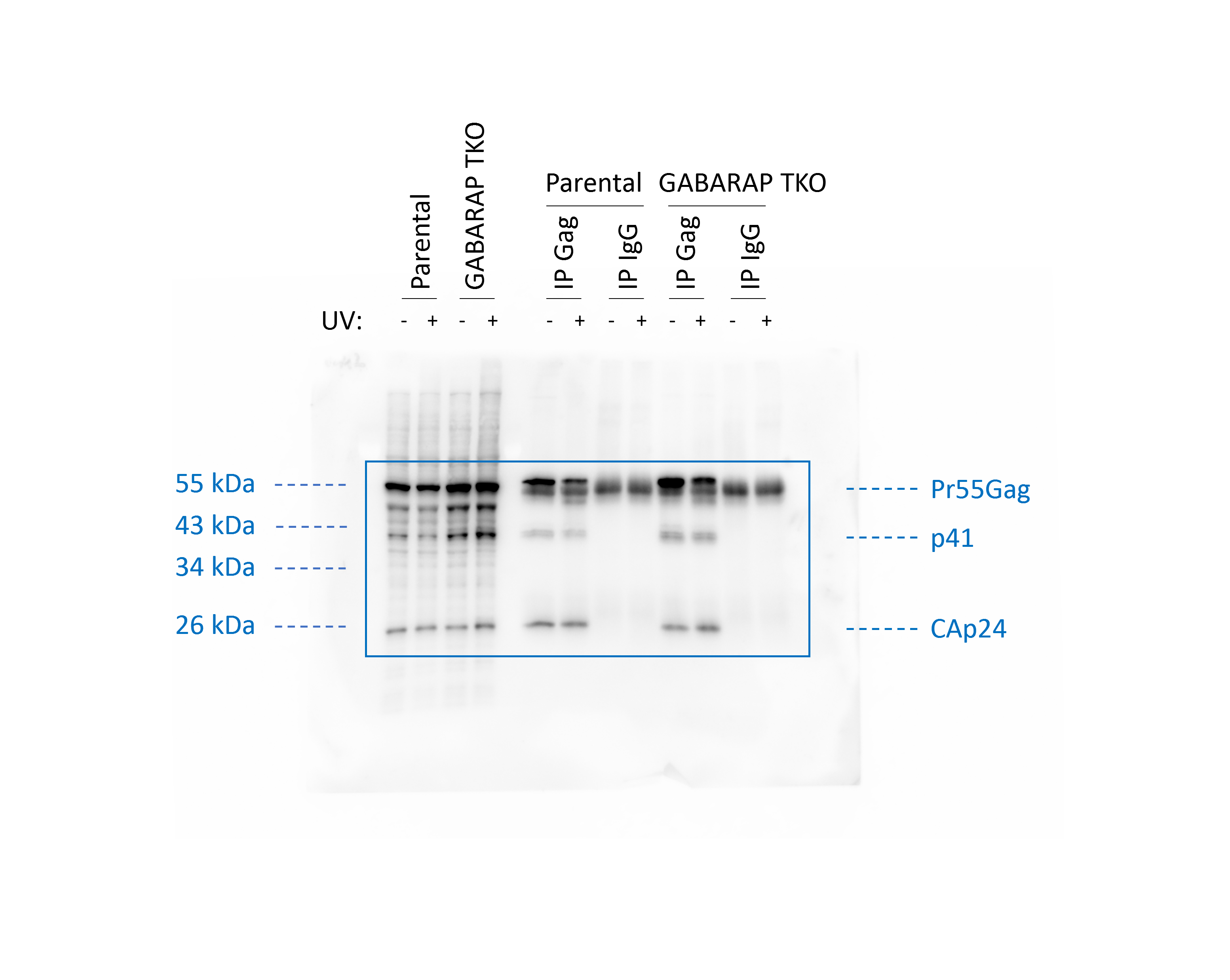

Supplement: Supplementary file 8 — Source data Fig. 6 [file 44319_2025_607_MOESM8_ESM.zip › Figure 6G/fig6F_Gag.tif]

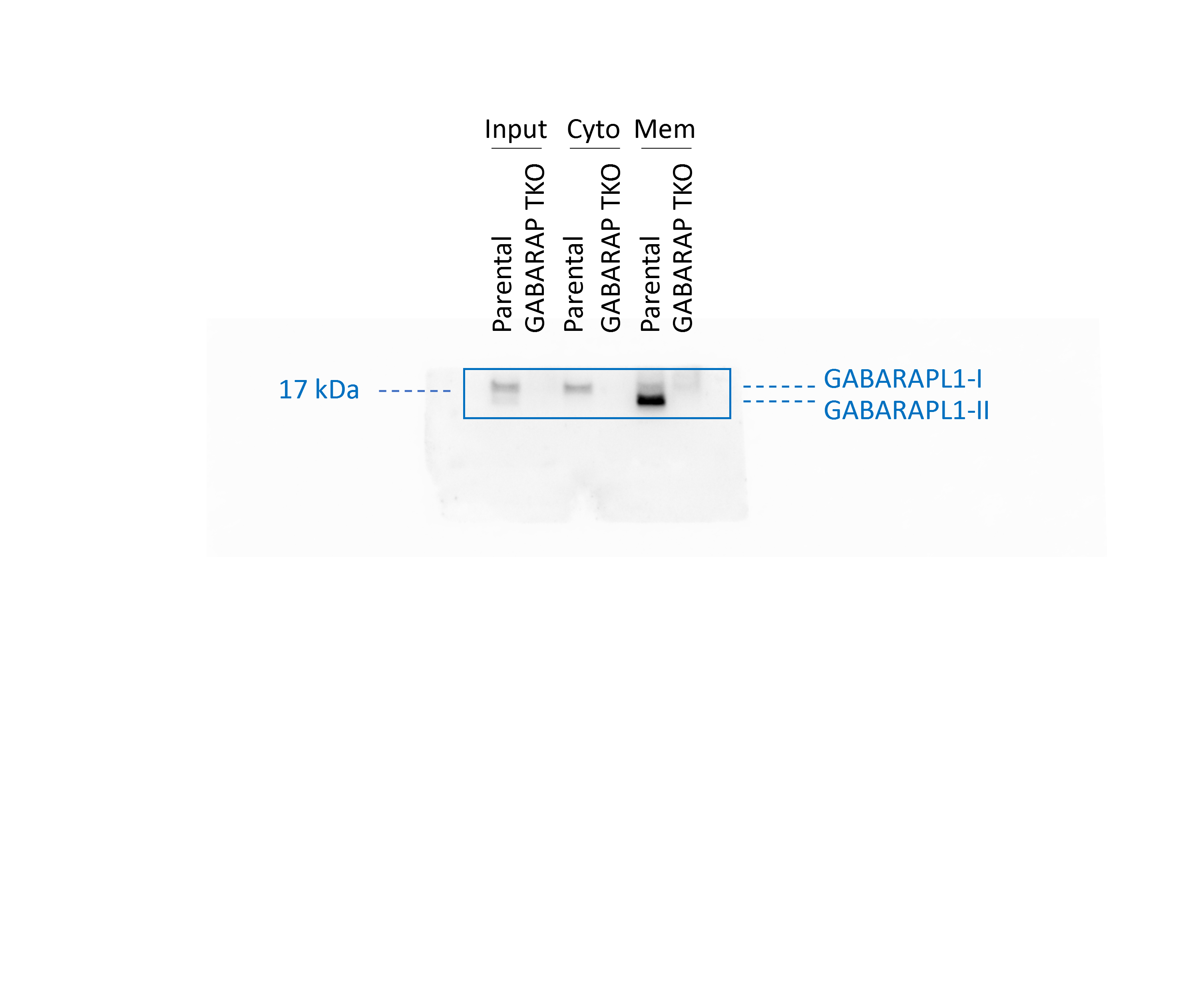

Supplement: Supplementary file 9 — Source data Fig. 7 [file 44319_2025_607_MOESM9_ESM.zip › Figure 7/Figure 7A/fig7A_GABARAPL1.tif]

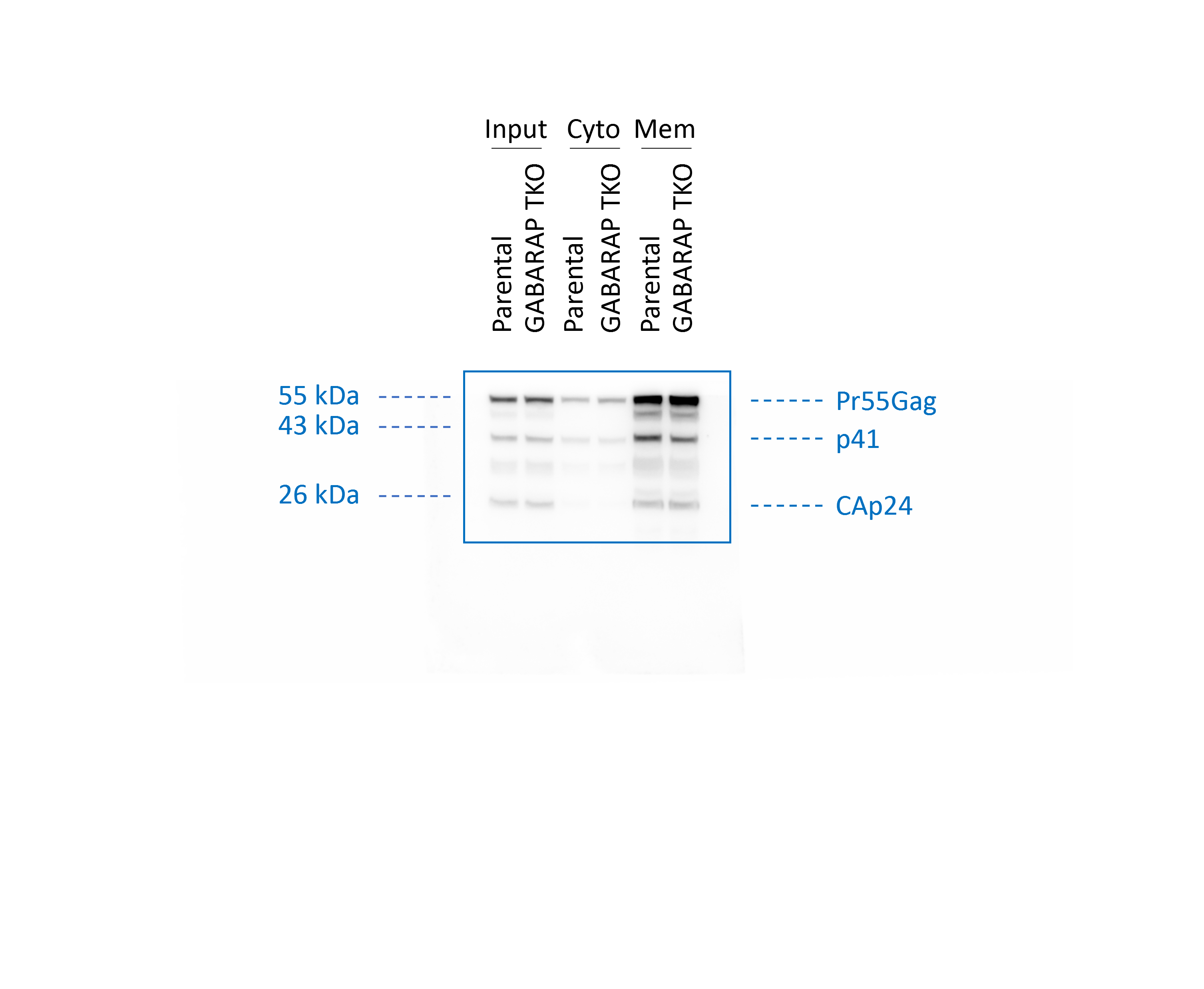

Supplement: Supplementary file 9 — Source data Fig. 7 [file 44319_2025_607_MOESM9_ESM.zip › Figure 7/Figure 7A/fig7A_Gag.tif]

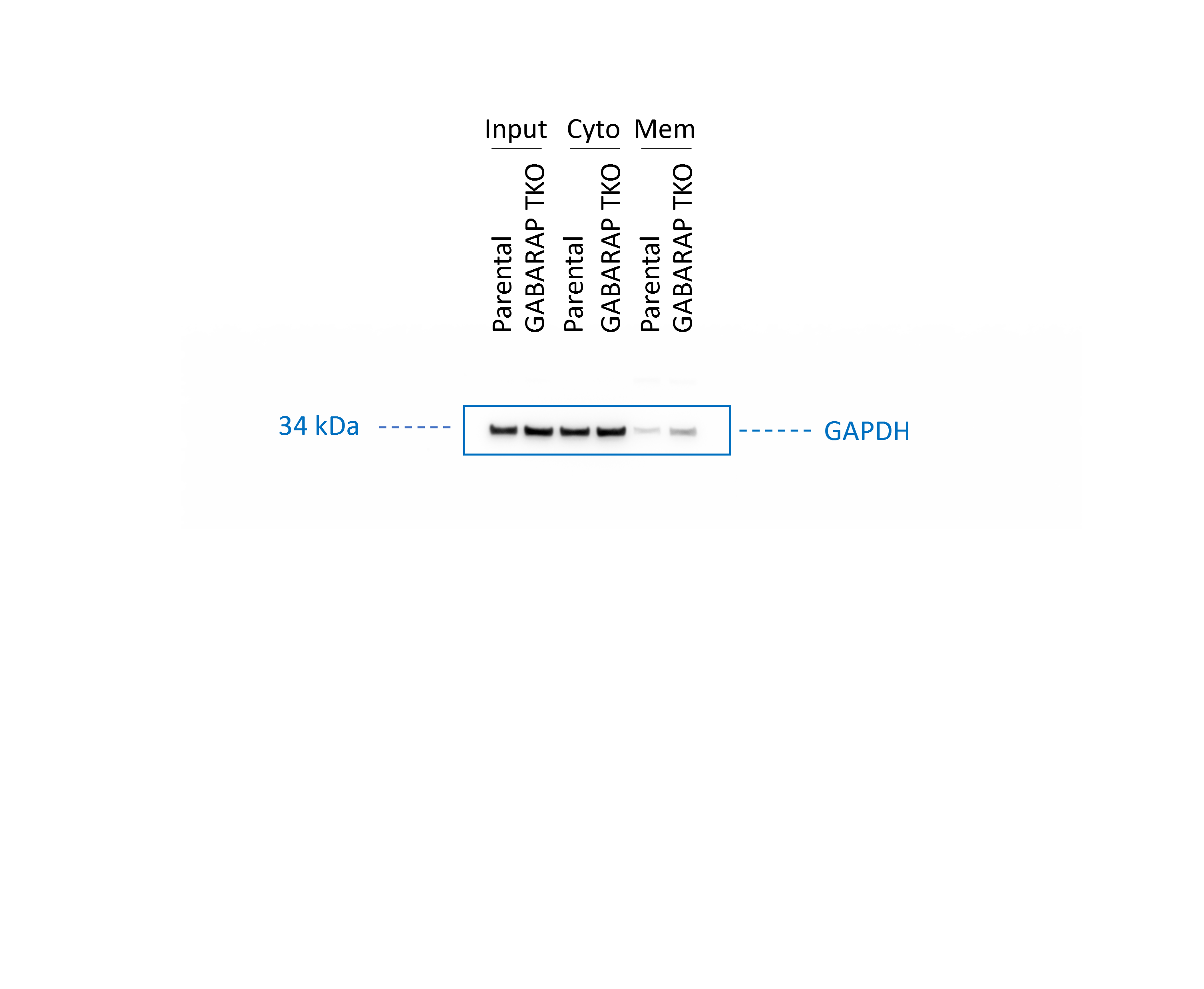

Supplement: Supplementary file 9 — Source data Fig. 7 [file 44319_2025_607_MOESM9_ESM.zip › Figure 7/Figure 7A/fig7A_GAPDH.tif]

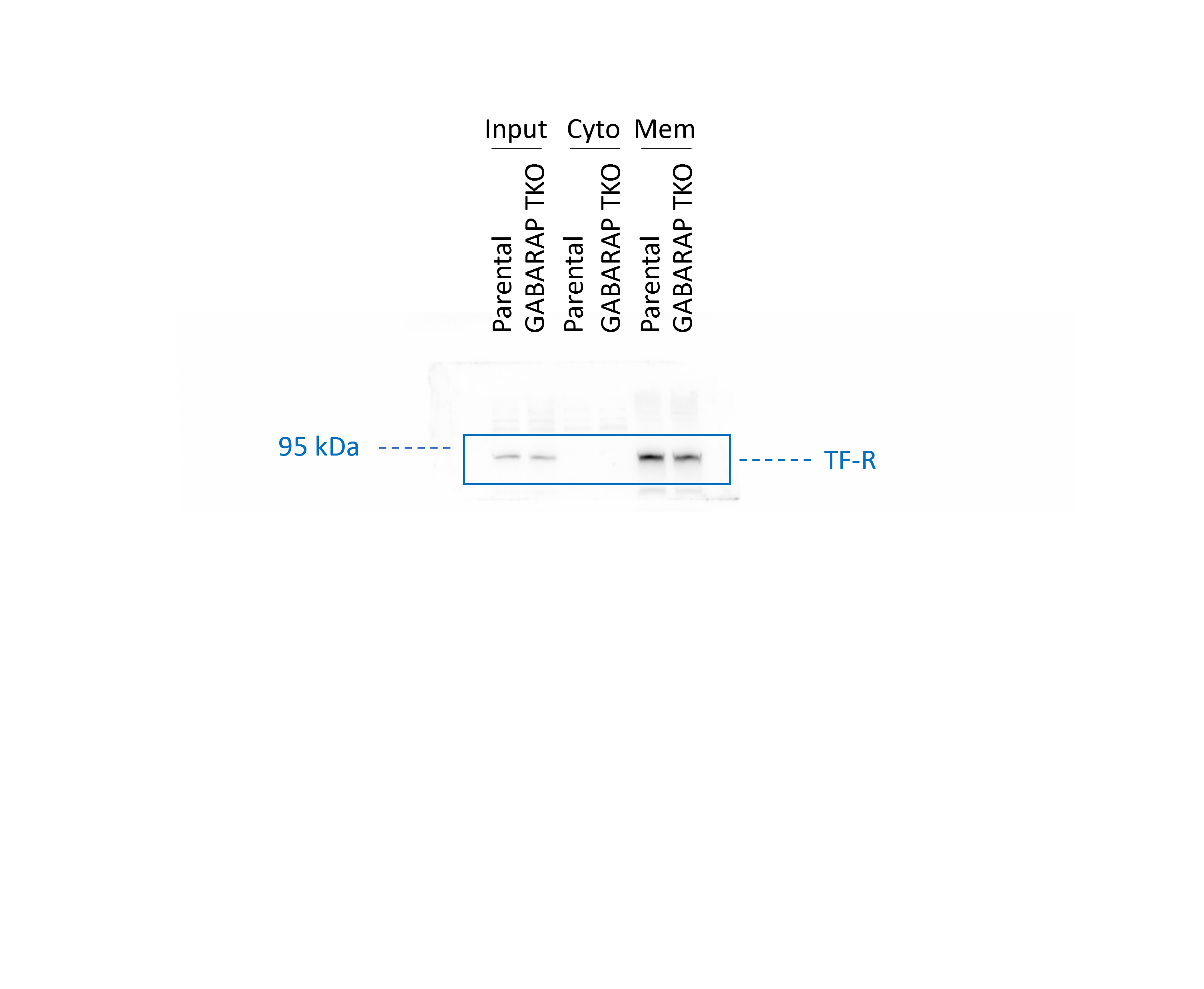

Supplement: Supplementary file 9 — Source data Fig. 7 [file 44319_2025_607_MOESM9_ESM.zip › Figure 7/Figure 7A/fig7A_TF-R.tif]

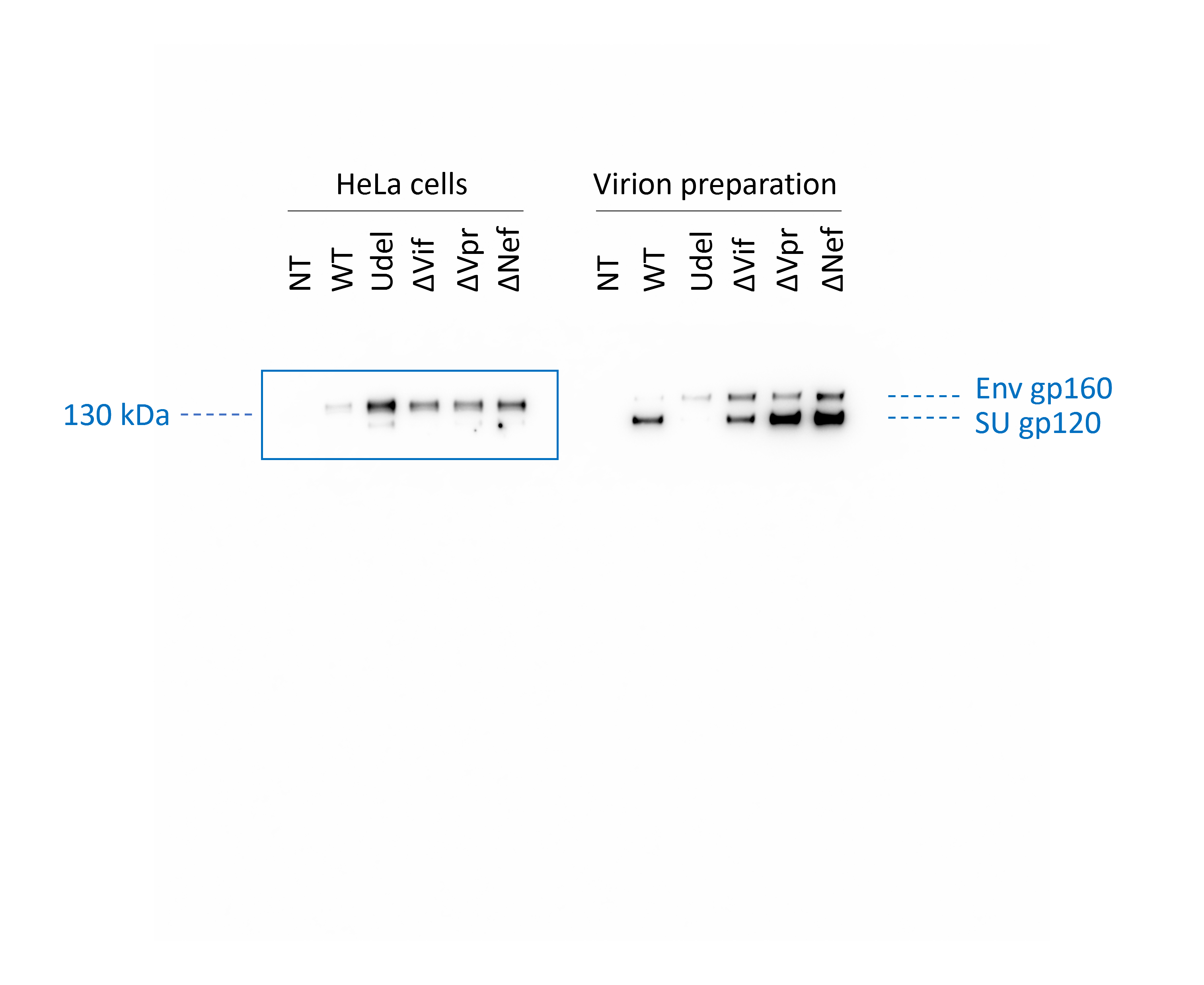

Supplement: Supplementary file 11 — Figure EV2 Source Data [file 44319_2025_607_MOESM11_ESM.zip › Figure EV2/figEV2_Env_cell.tif]

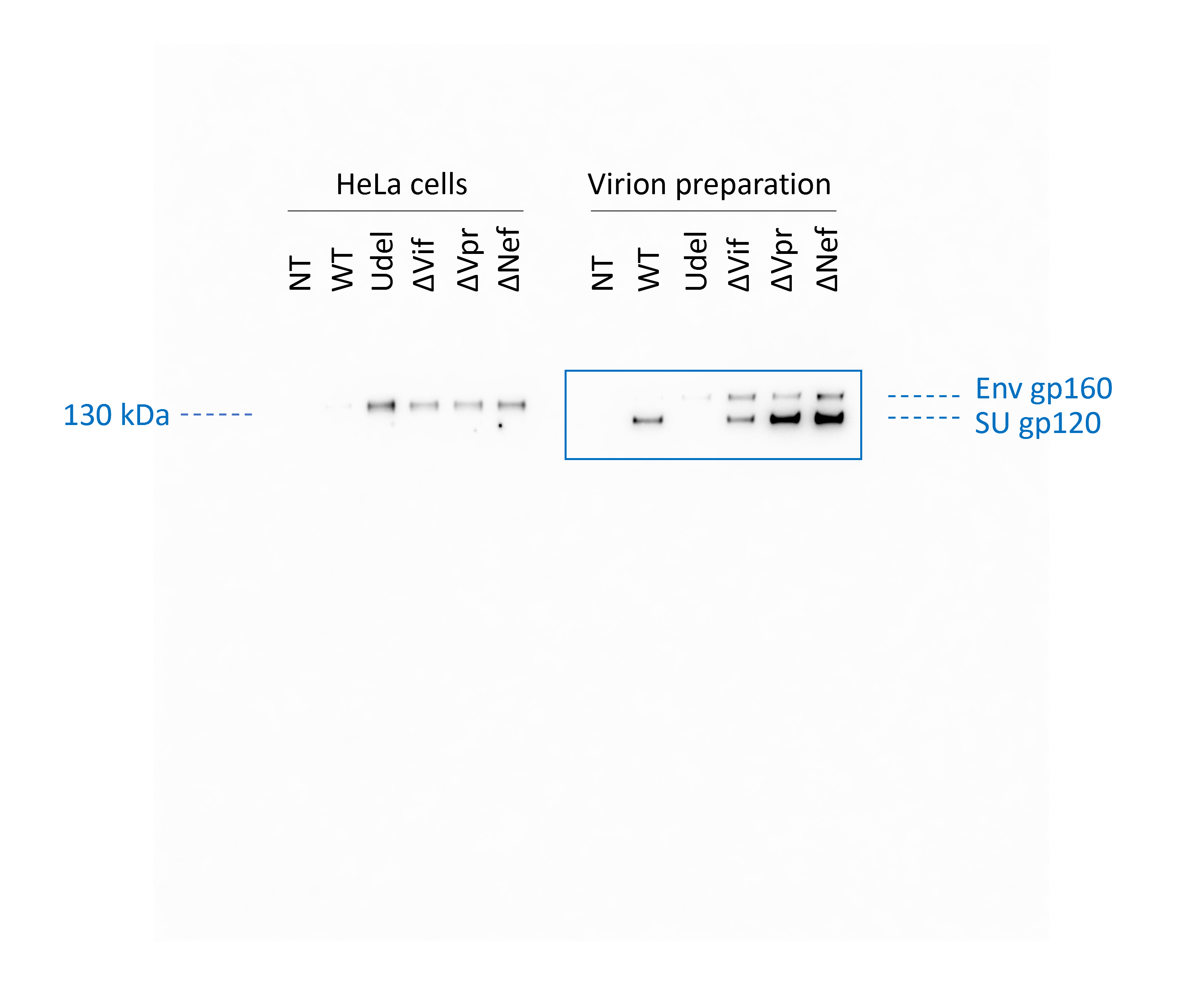

Supplement: Supplementary file 11 — Figure EV2 Source Data [file 44319_2025_607_MOESM11_ESM.zip › Figure EV2/figEV2_Env_virion prep.tif]

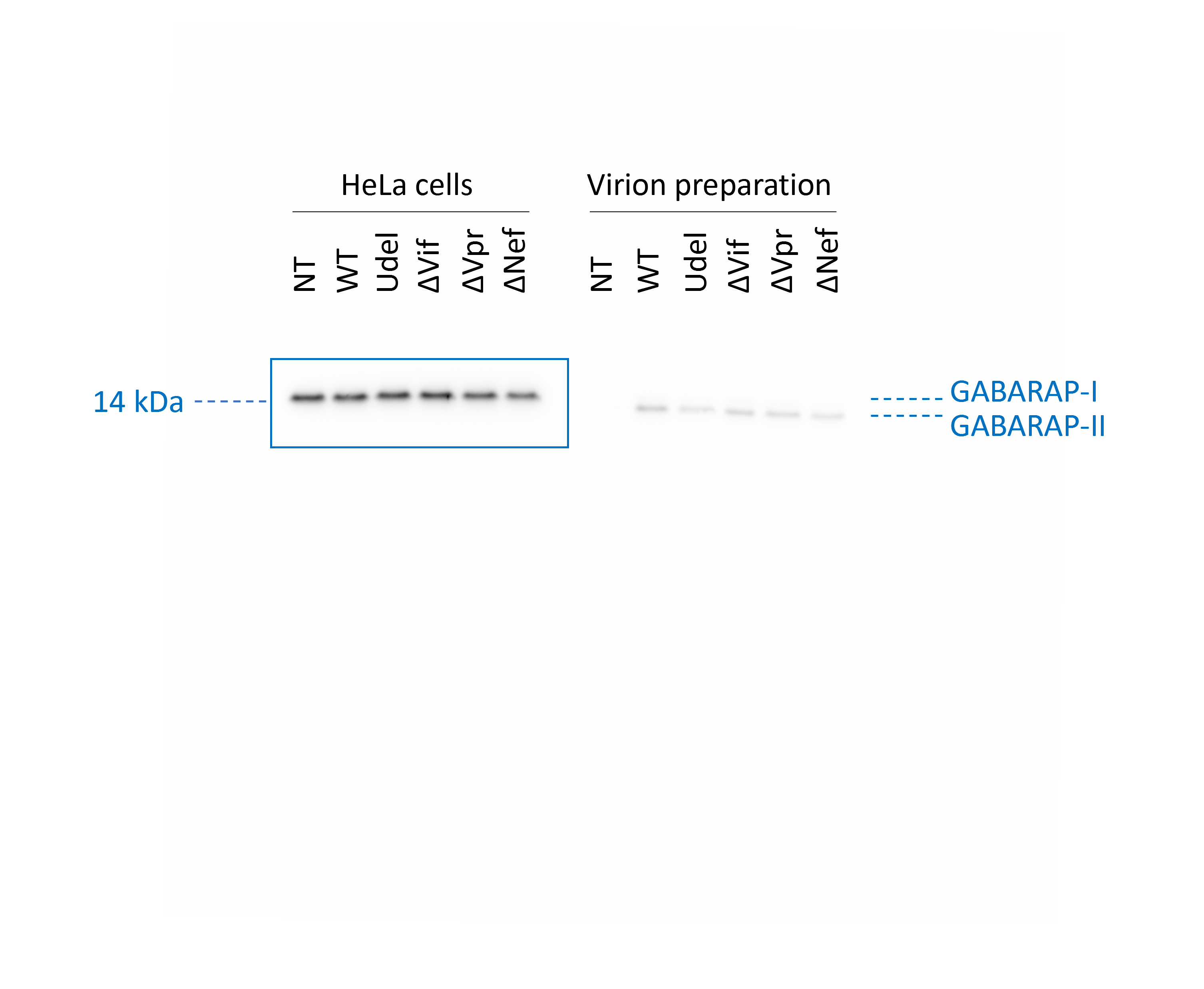

Supplement: Supplementary file 11 — Figure EV2 Source Data [file 44319_2025_607_MOESM11_ESM.zip › Figure EV2/figEV2_GABARAP_cell.tif]

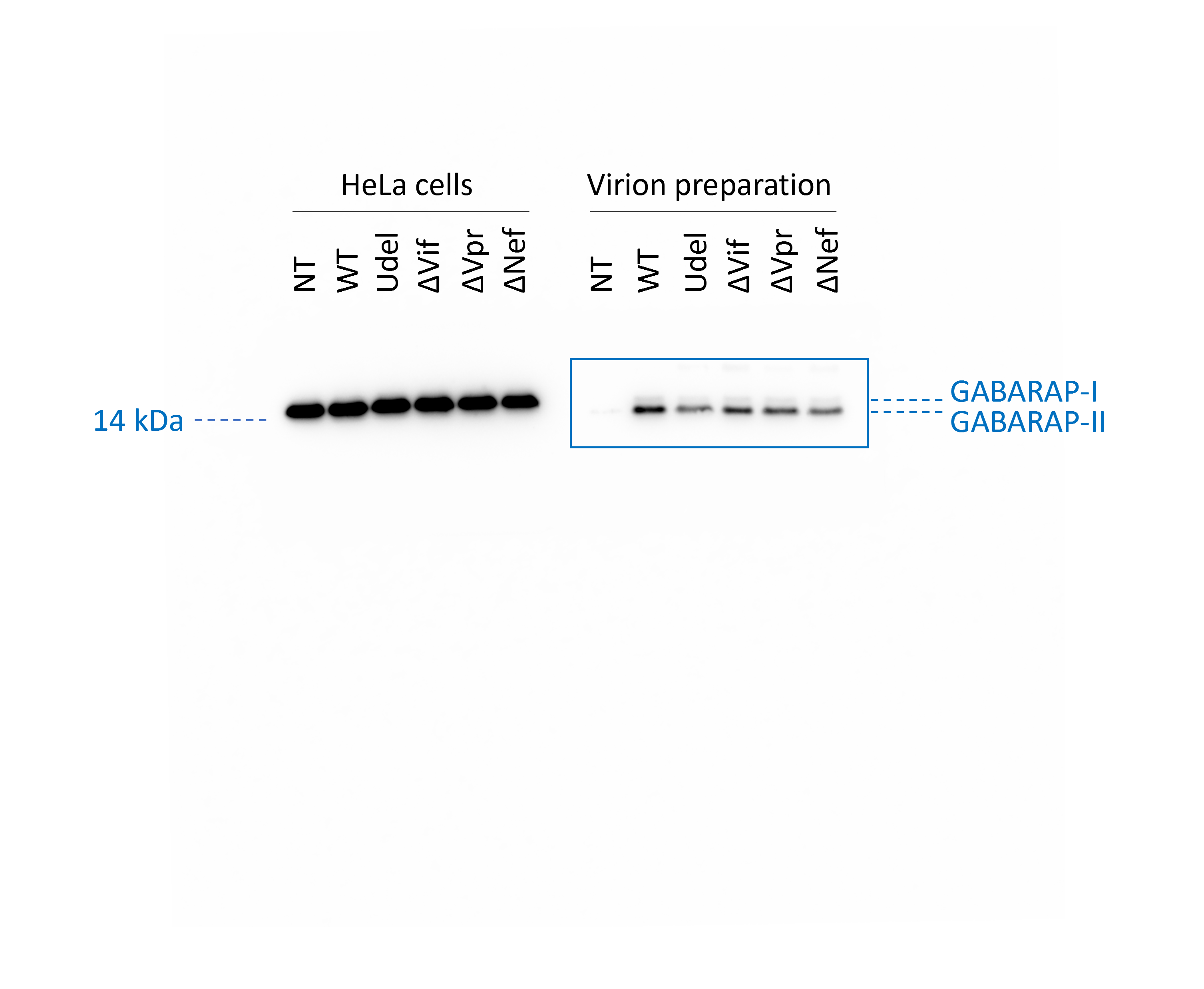

Supplement: Supplementary file 11 — Figure EV2 Source Data [file 44319_2025_607_MOESM11_ESM.zip › Figure EV2/figEV2_GABARAP_virion prep.tif]

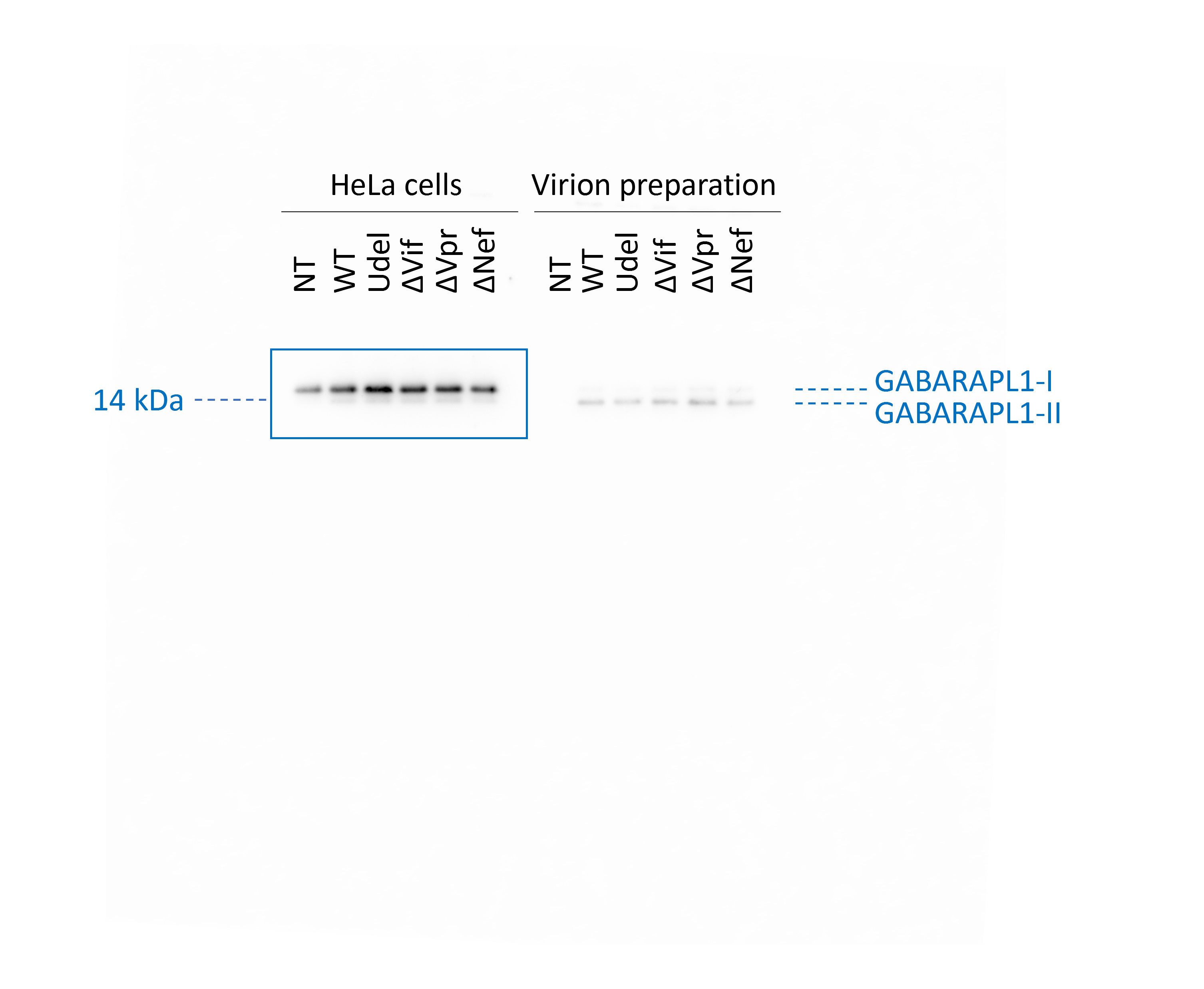

Supplement: Supplementary file 11 — Figure EV2 Source Data [file 44319_2025_607_MOESM11_ESM.zip › Figure EV2/figEV2_GABARAPL1_cell.tif]

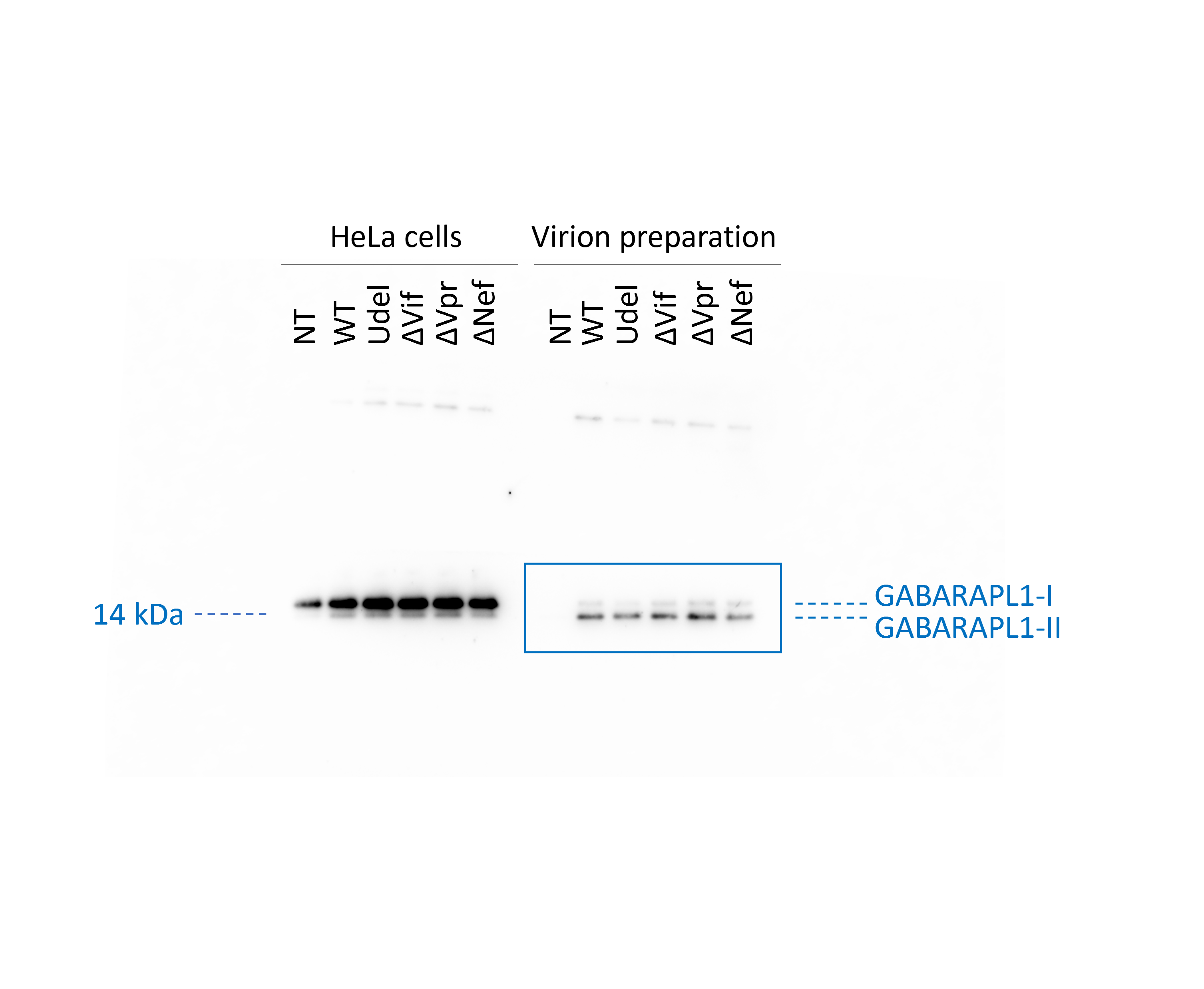

Supplement: Supplementary file 11 — Figure EV2 Source Data [file 44319_2025_607_MOESM11_ESM.zip › Figure EV2/figEV2_GABARAPL1_virion prep.tif]

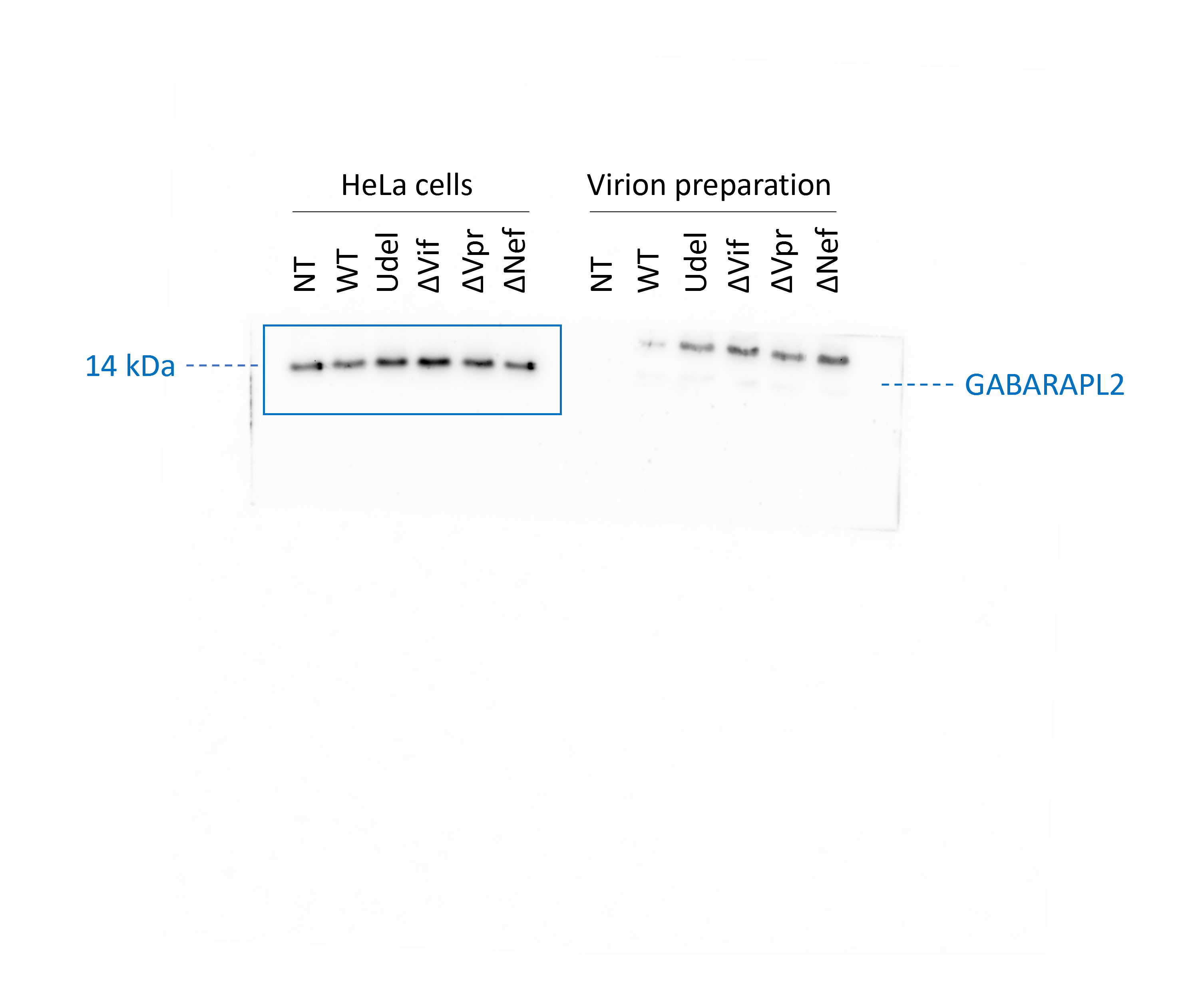

Supplement: Supplementary file 11 — Figure EV2 Source Data [file 44319_2025_607_MOESM11_ESM.zip › Figure EV2/figEV2_GABARAPL2_cell.tif]

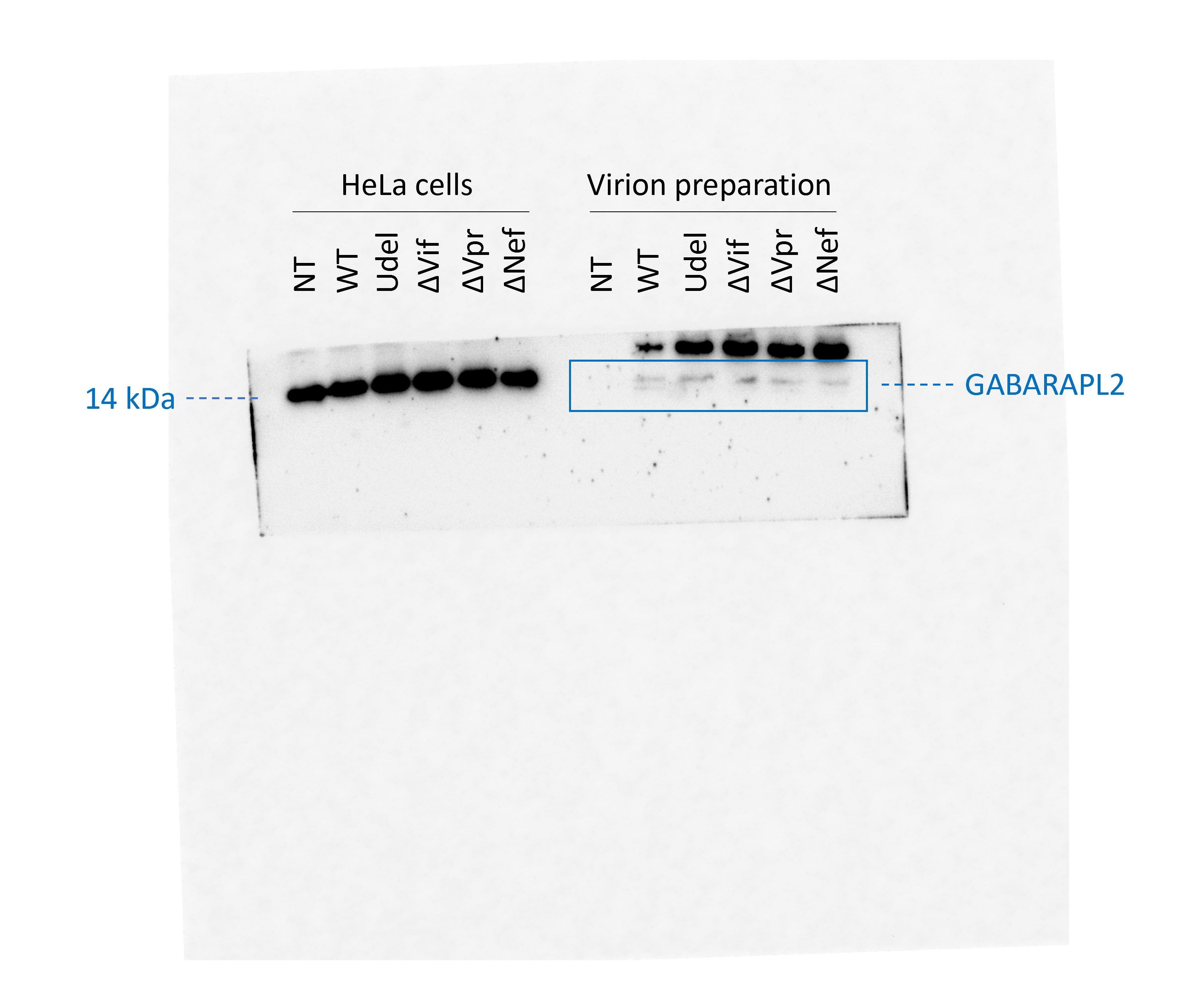

Supplement: Supplementary file 11 — Figure EV2 Source Data [file 44319_2025_607_MOESM11_ESM.zip › Figure EV2/figEV2_GABARAPL2_virion prep.tif]

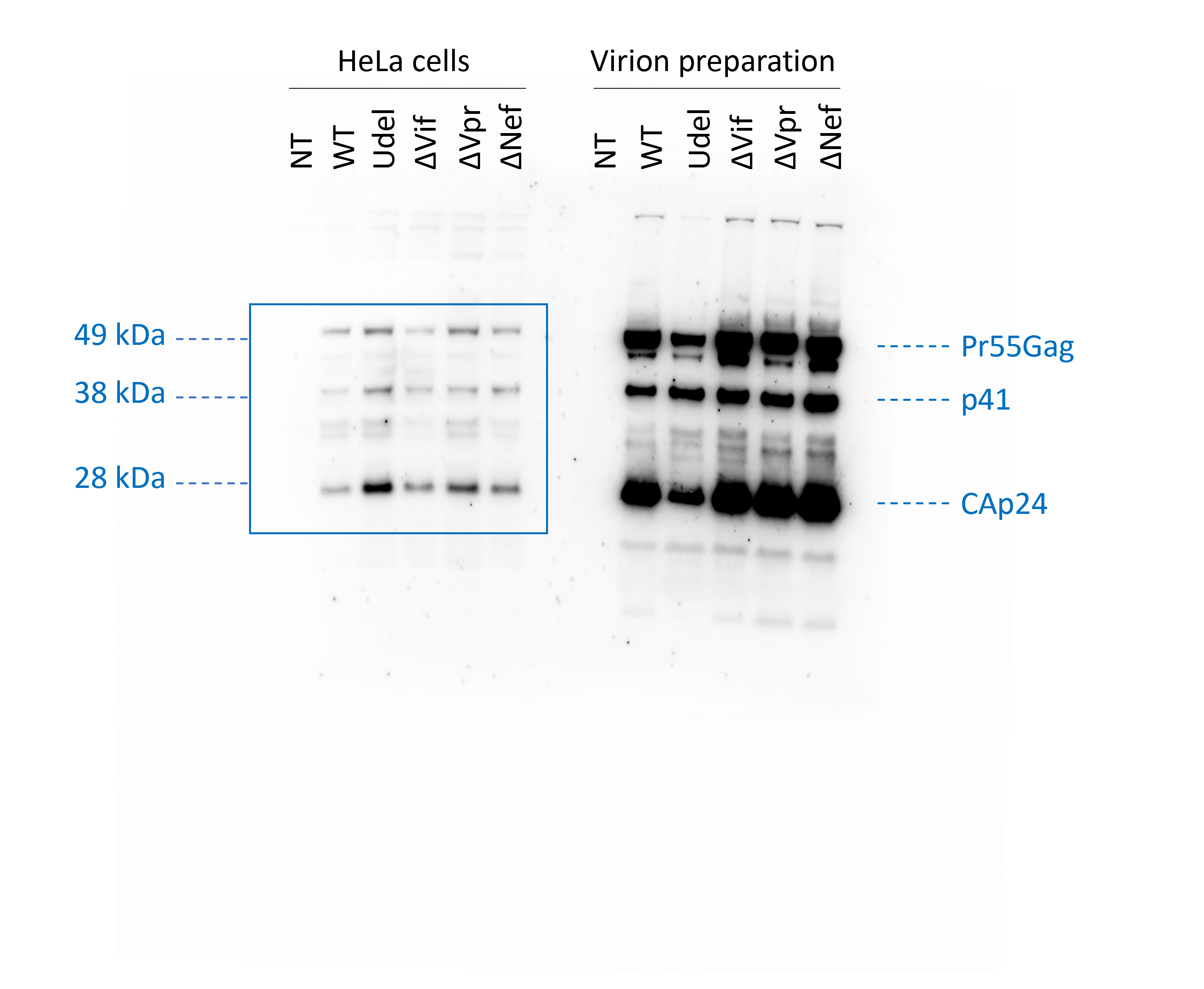

Supplement: Supplementary file 11 — Figure EV2 Source Data [file 44319_2025_607_MOESM11_ESM.zip › Figure EV2/figEV2_Gag_cell.tif]

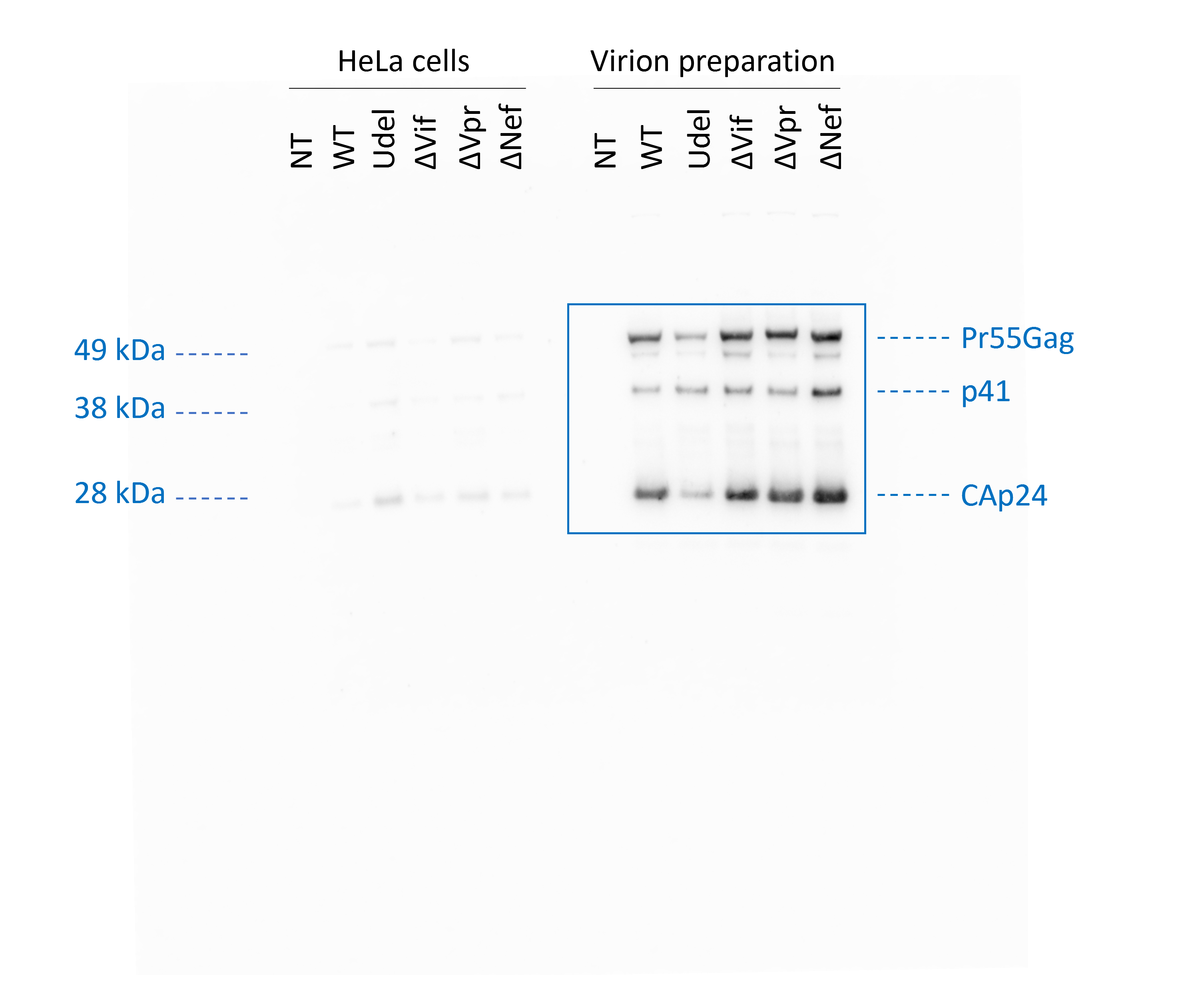

Supplement: Supplementary file 11 — Figure EV2 Source Data [file 44319_2025_607_MOESM11_ESM.zip › Figure EV2/figEV2_Gag_virion prep.tif]

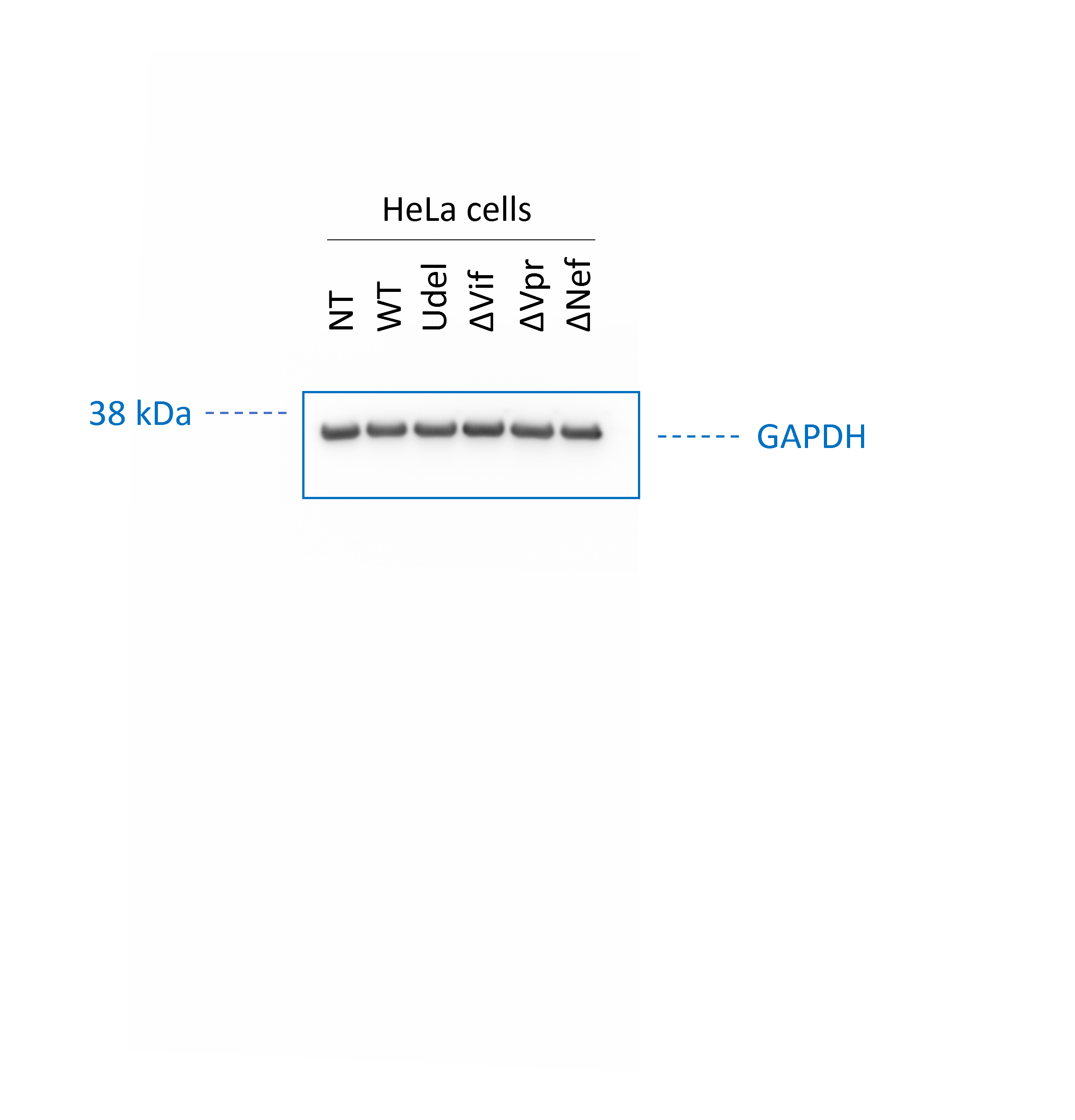

Supplement: Supplementary file 11 — Figure EV2 Source Data [file 44319_2025_607_MOESM11_ESM.zip › Figure EV2/figEV2_GAPDH.tif]

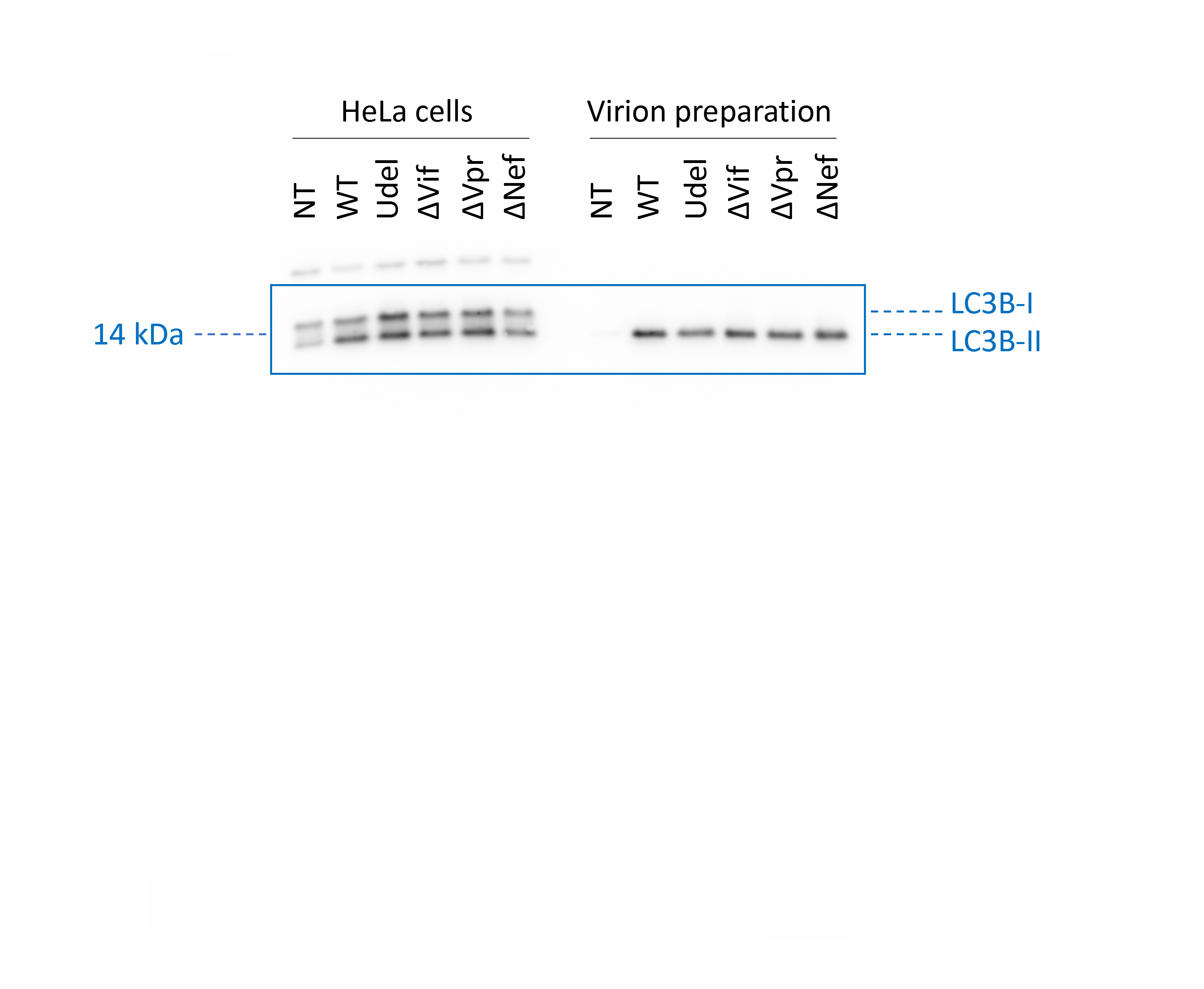

Supplement: Supplementary file 11 — Figure EV2 Source Data [file 44319_2025_607_MOESM11_ESM.zip › Figure EV2/figEV2_LC3B.tif]

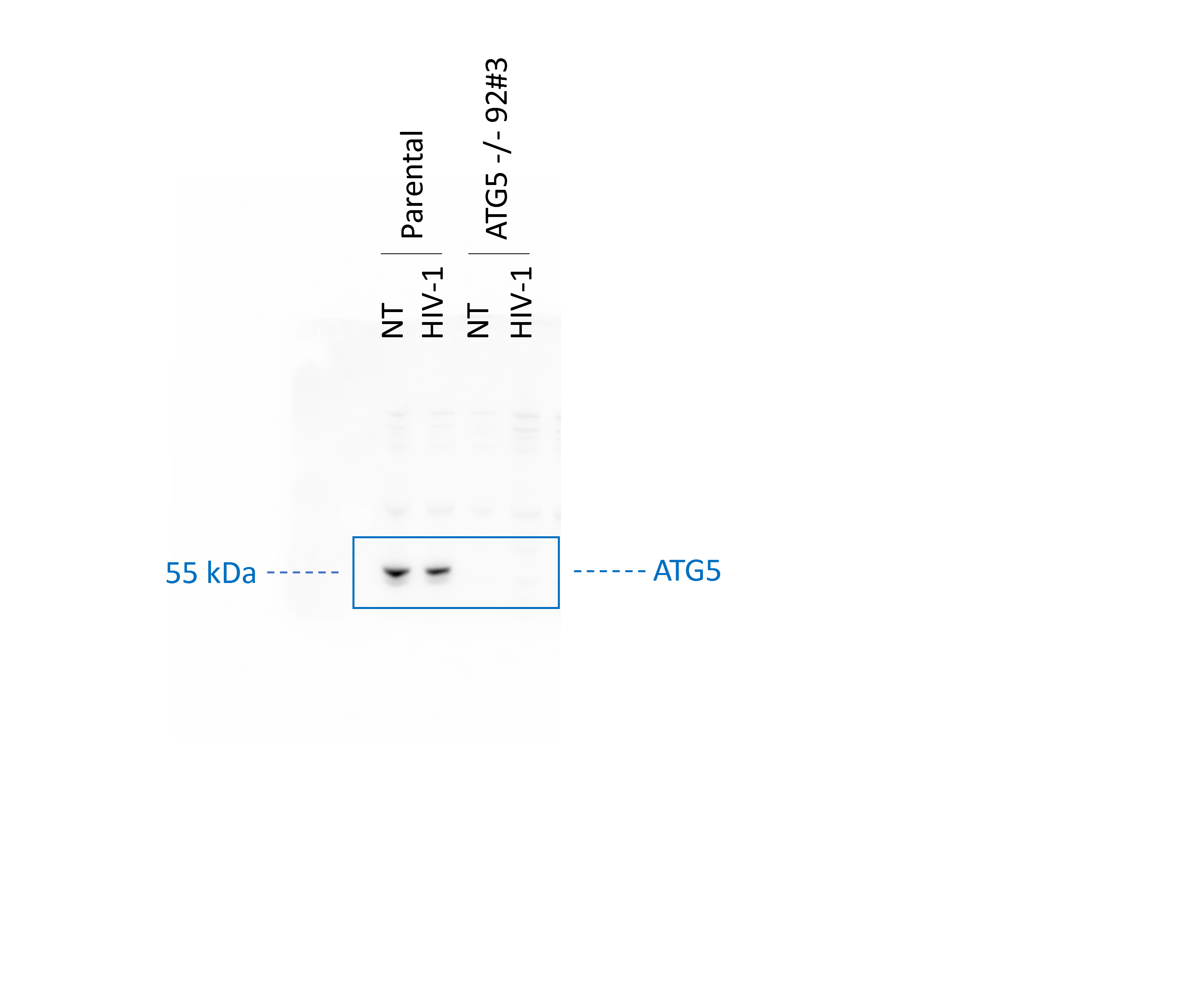

Supplement: Supplementary file 13 — Figure EV4 Source Data [file 44319_2025_607_MOESM13_ESM.zip › Figure EV4 B/figEV4 B_ATG5.tif]

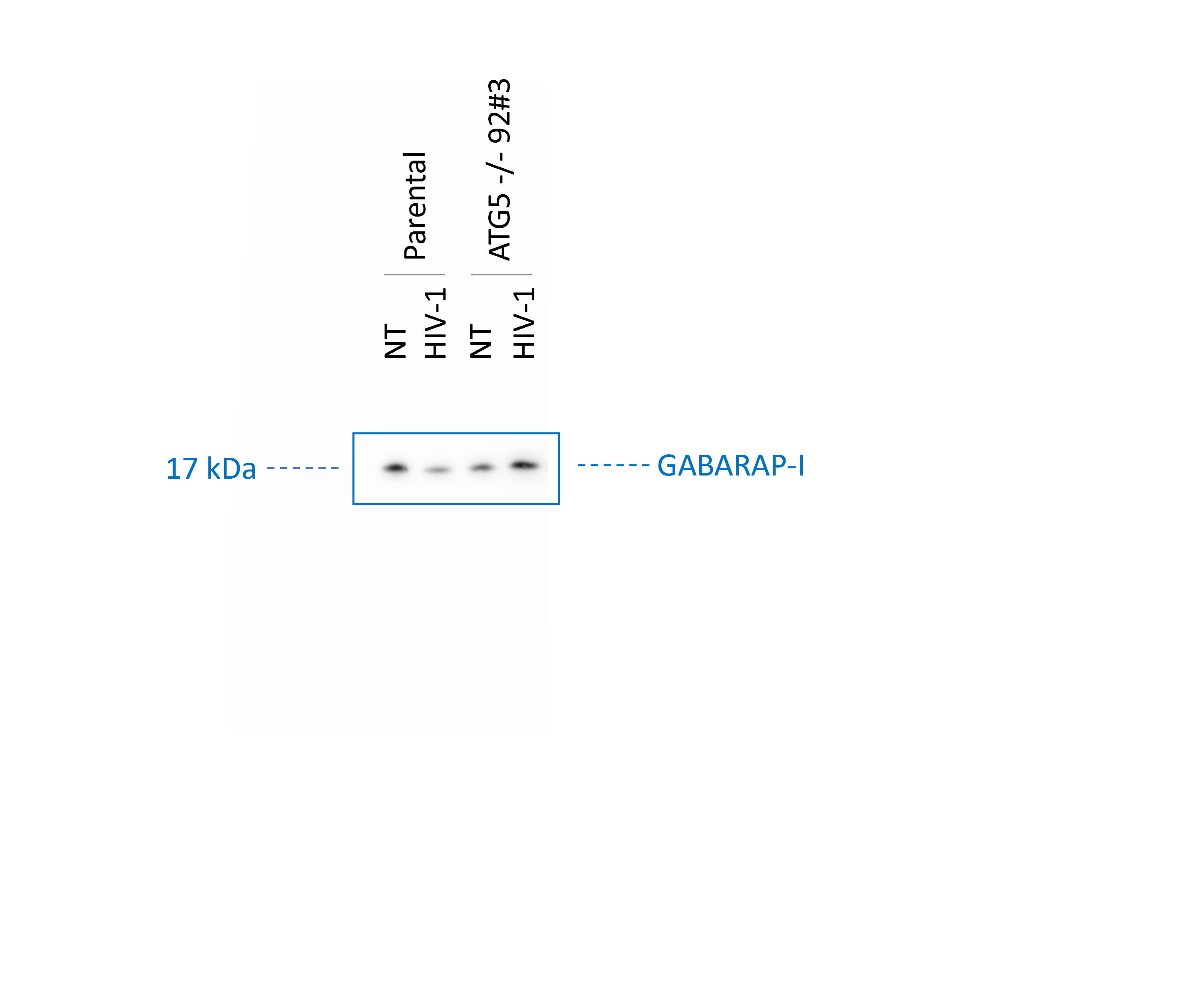

Supplement: Supplementary file 13 — Figure EV4 Source Data [file 44319_2025_607_MOESM13_ESM.zip › Figure EV4 B/figEV4 B_GABARAP_cell.tif]

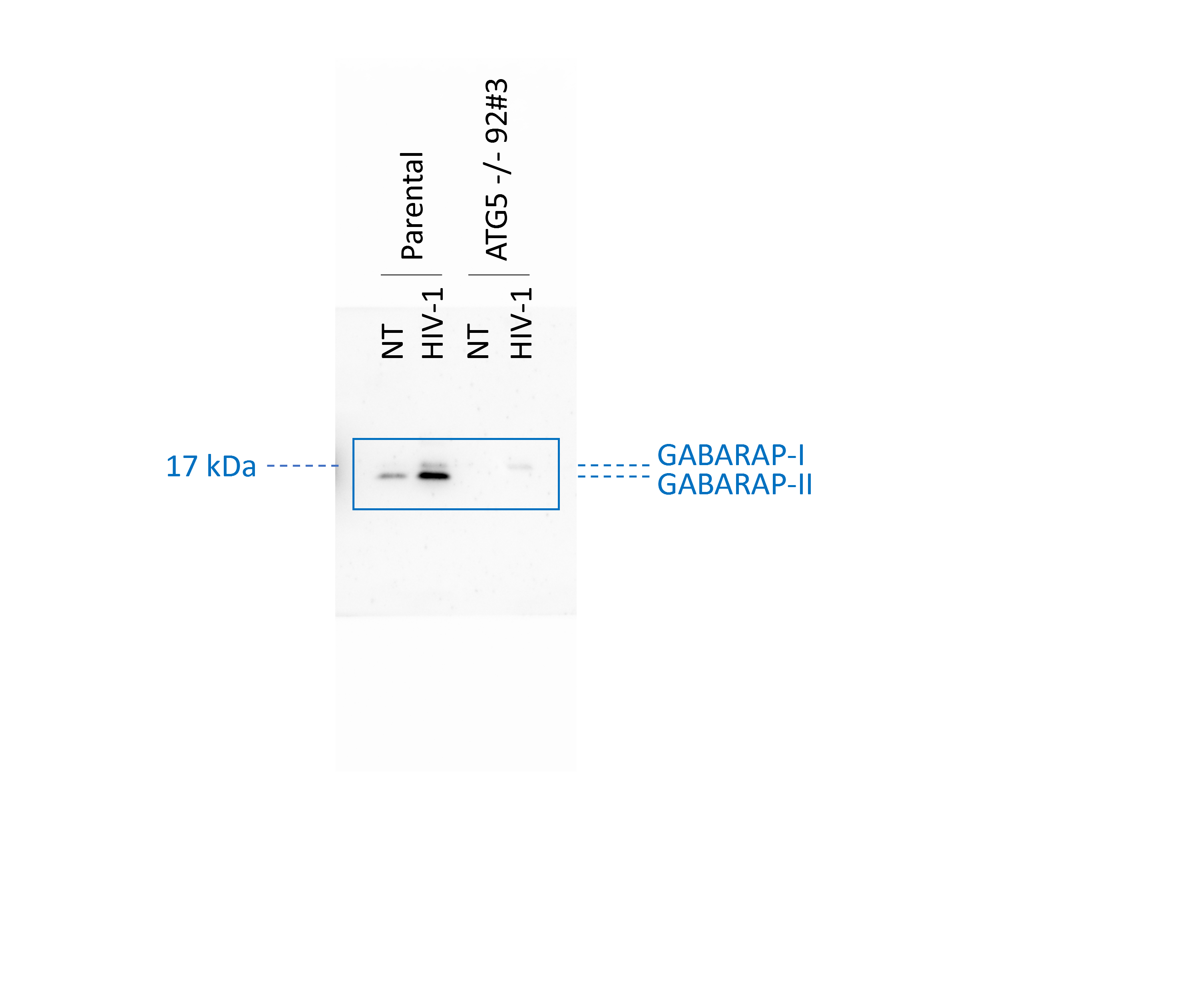

Supplement: Supplementary file 13 — Figure EV4 Source Data [file 44319_2025_607_MOESM13_ESM.zip › Figure EV4 B/figEV4 B_GABARAP_virion prep.tif]

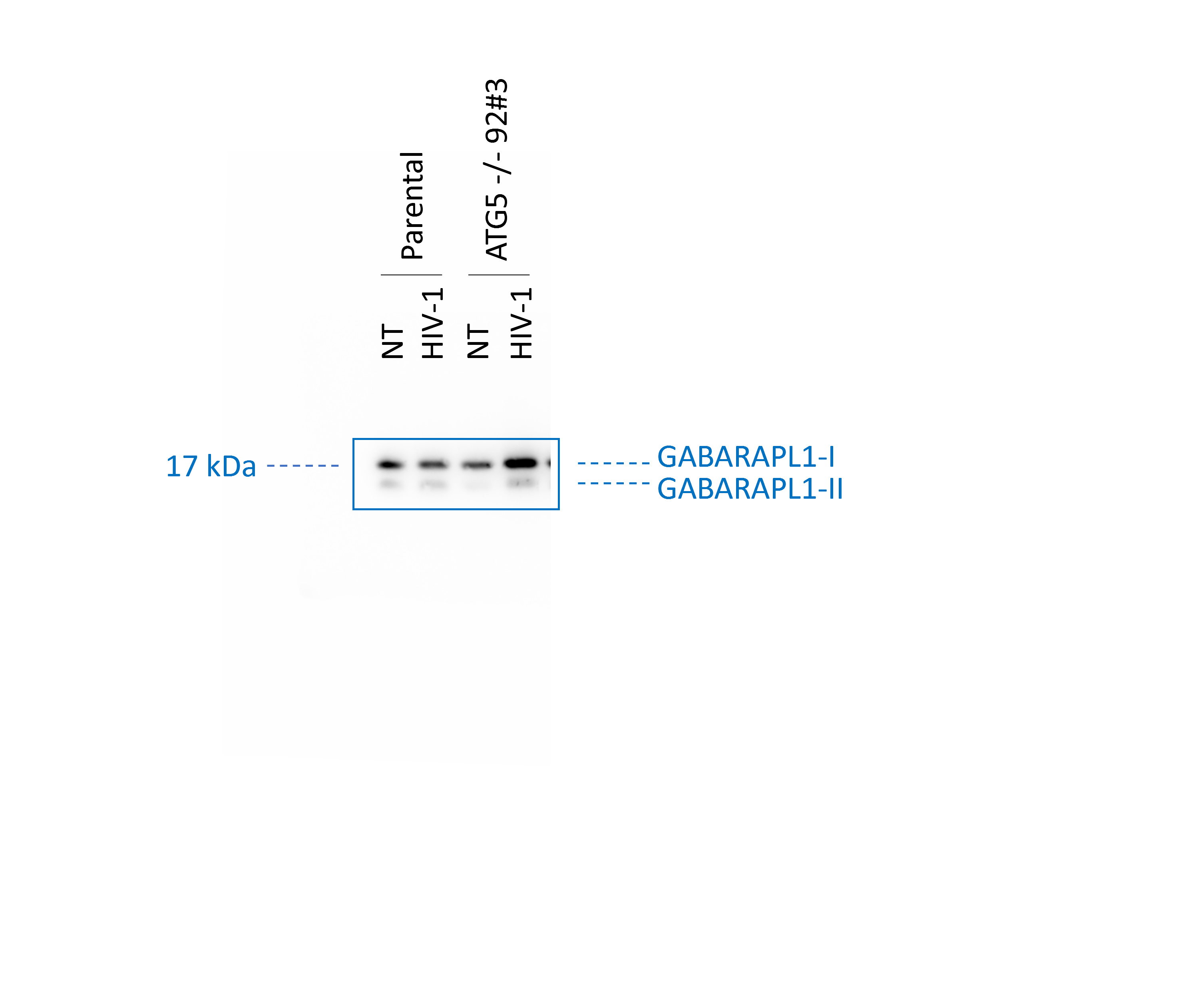

Supplement: Supplementary file 13 — Figure EV4 Source Data [file 44319_2025_607_MOESM13_ESM.zip › Figure EV4 B/figEV4 B_GABARAPL1_cell.tif]
