# Supplementary material for: GABARAP proteins regulate the packaging of HIV-1 genomic RNA into virions
Source: EMBO Rep. 2025 Oct 31;26(23):5826–58. doi: 10.1038/s44319-025-00607-1 (PMC12678799; doi:10.1038/s44319-025-00607-1)
Supplement: Supplementary file 9 — Source data Fig. 7 [file 44319_2025_607_MOESM9_ESM.zip › Figure 7/Figure 7C/README_Fig7C_Microscopic images_EMBO reports.docx]

**Fig 7C - Processing Steps**

**Raw Images data provided are the images after Image resolution adjustment**

1. **Image projection**
   - The *mean intensity projection* function was applied, producing a single image in which each pixel represents the average intensity value across the entire z-stack at that location using Fiji software.
   - Each image was saved as Jpg.

This approach ensures that the resulting image accurately reflects the overall signal distribution throughout the sample’s depth.

1. **Image Resolution Adjustment** (using Photoshop CS5, Adobe)
   - Each image was resized to a resolution of 300dpi.
   - Each image was saved as Tif.

This uniform resolution was set to standardize image quality

1. **Cropping** (using Photoshop CS5, Adobe)
   - All images were cropped to remove unnecessary areas not required for presentation.
   - The cropping area were applied consistently across all images.
2. **Brightness and Contrast Adjustment** (using Photoshop CS5, Adobe)
   - Brightness and contrast were adjusted using the same values
   - The adjustments were applied globally to every image to preserve color consistency.
