# Supplementary figures and images for: GABARAP proteins regulate the packaging of HIV-1 genomic RNA into virions (part 2 of 2)
Source: EMBO Rep. 2025 Oct 31;26(23):5826–58. doi: 10.1038/s44319-025-00607-1 (PMC12678799; doi:10.1038/s44319-025-00607-1)

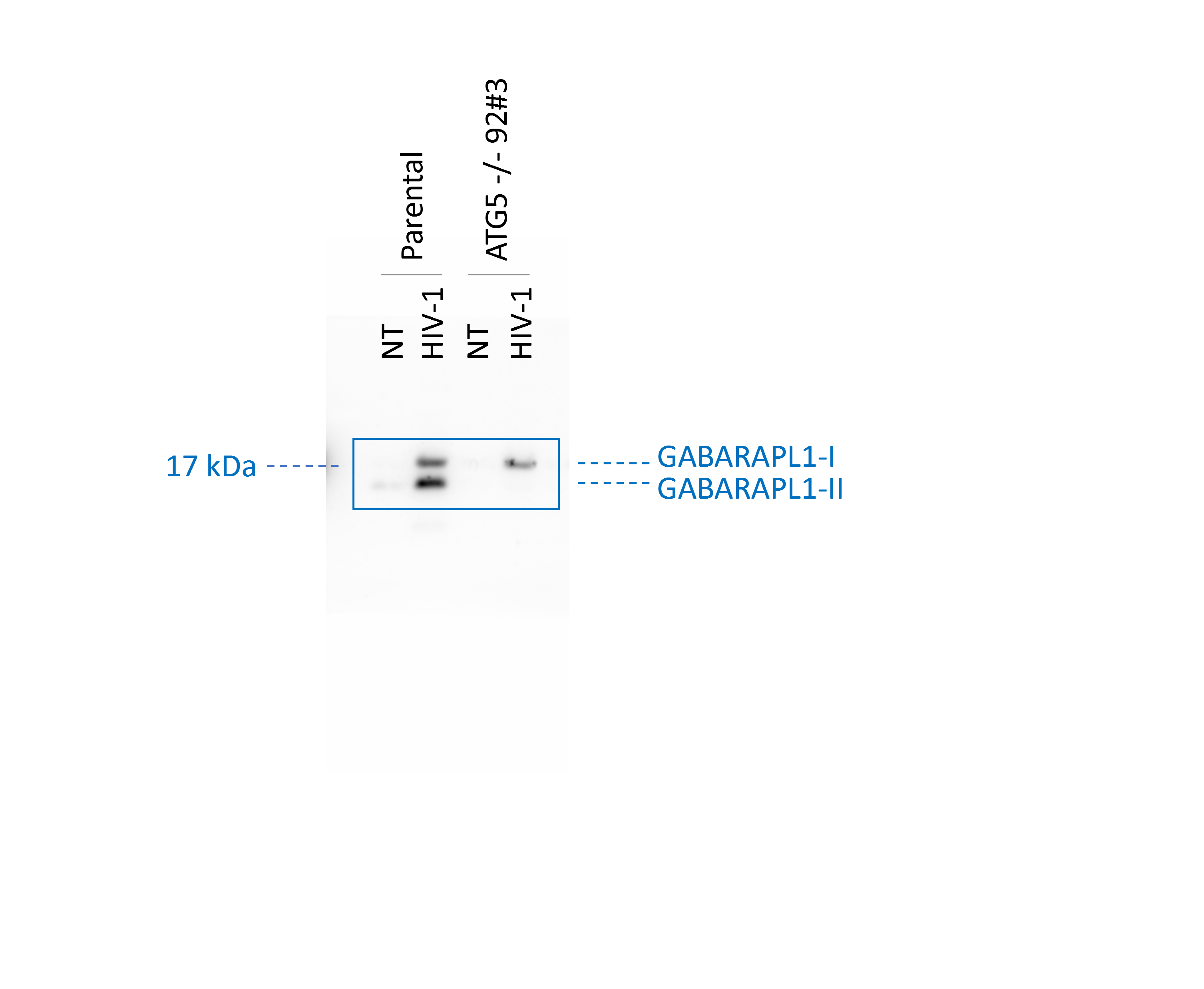

Supplement: Supplementary file 13 — Figure EV4 Source Data [file 44319_2025_607_MOESM13_ESM.zip › Figure EV4 B/figEV4 B_GABARAPL1_virion prep.tif]

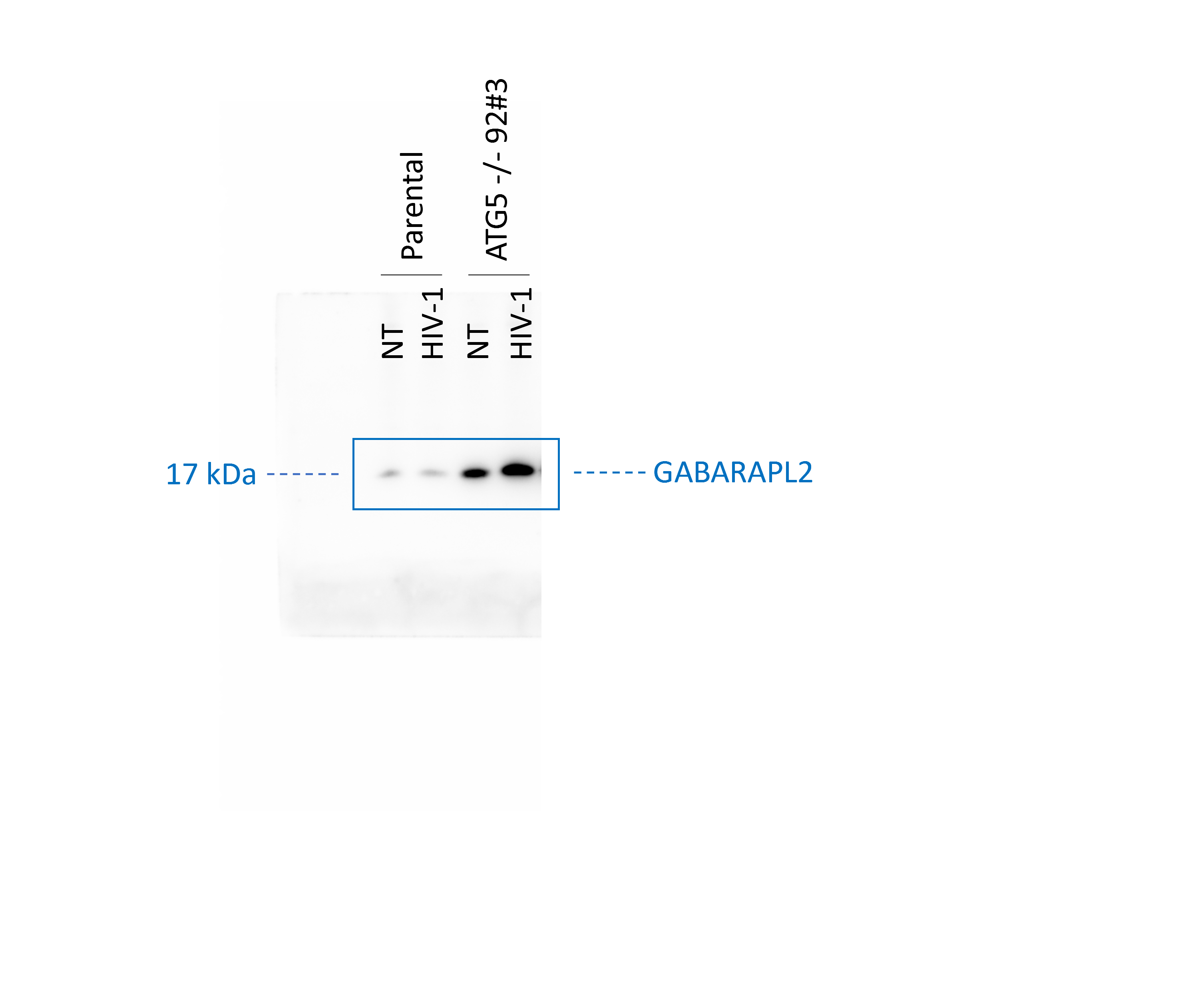

Supplement: Supplementary file 13 — Figure EV4 Source Data [file 44319_2025_607_MOESM13_ESM.zip › Figure EV4 B/figEV4 B_GABARAPL2_cell.tif]

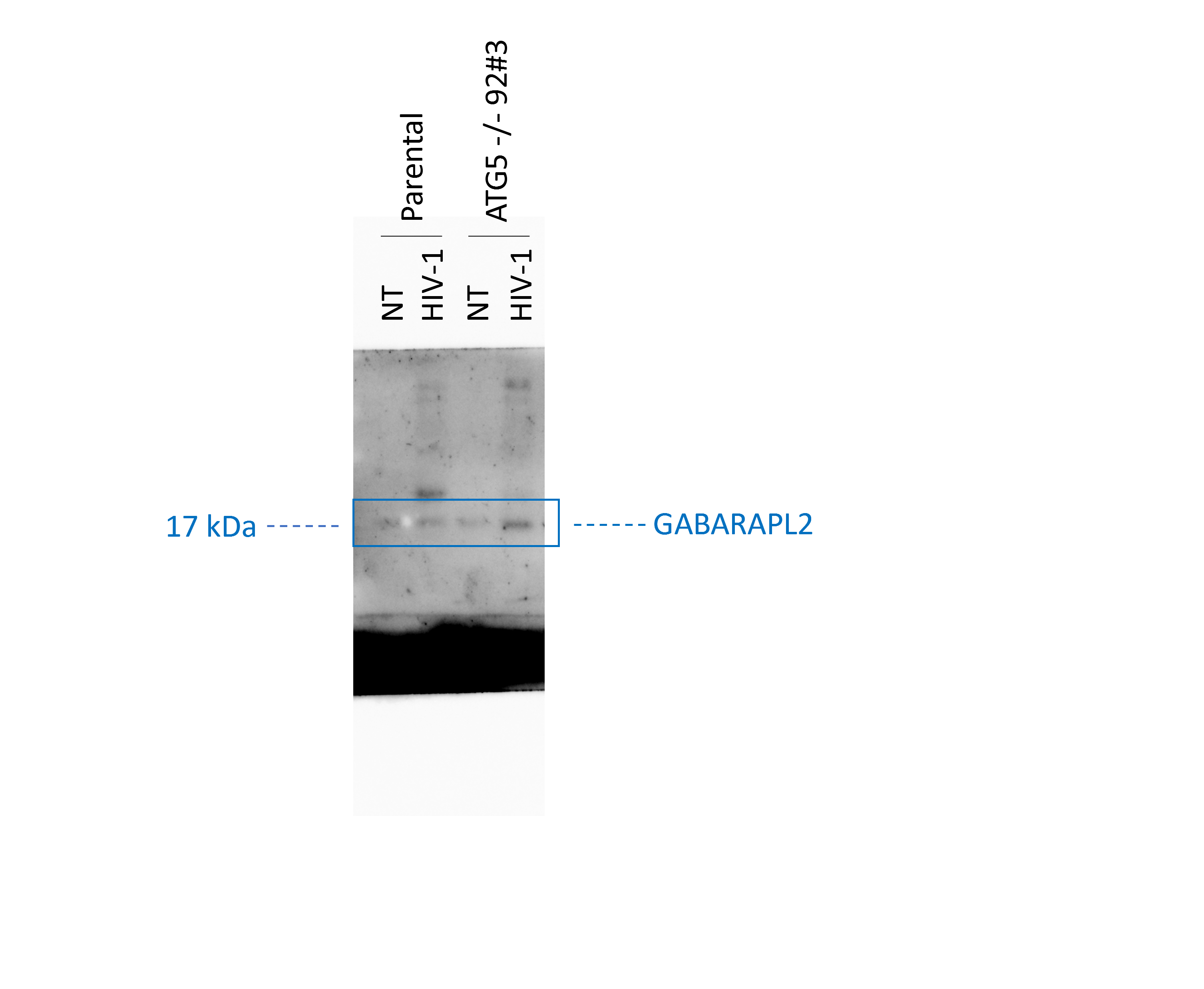

Supplement: Supplementary file 13 — Figure EV4 Source Data [file 44319_2025_607_MOESM13_ESM.zip › Figure EV4 B/figEV4 B_GABARAPL2_virion prep.tif]

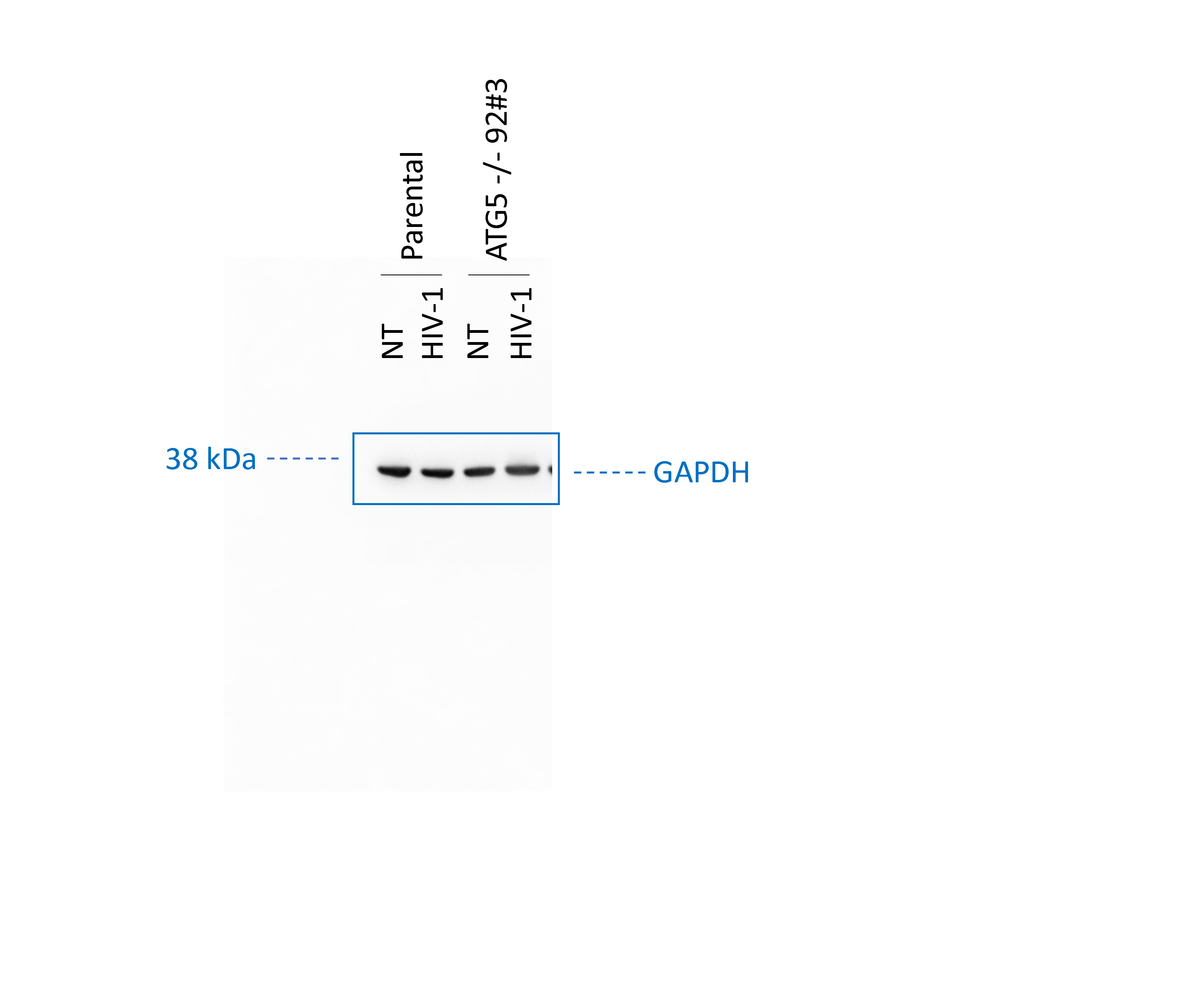

Supplement: Supplementary file 13 — Figure EV4 Source Data [file 44319_2025_607_MOESM13_ESM.zip › Figure EV4 B/figEV4 B_GAPDH.tif]

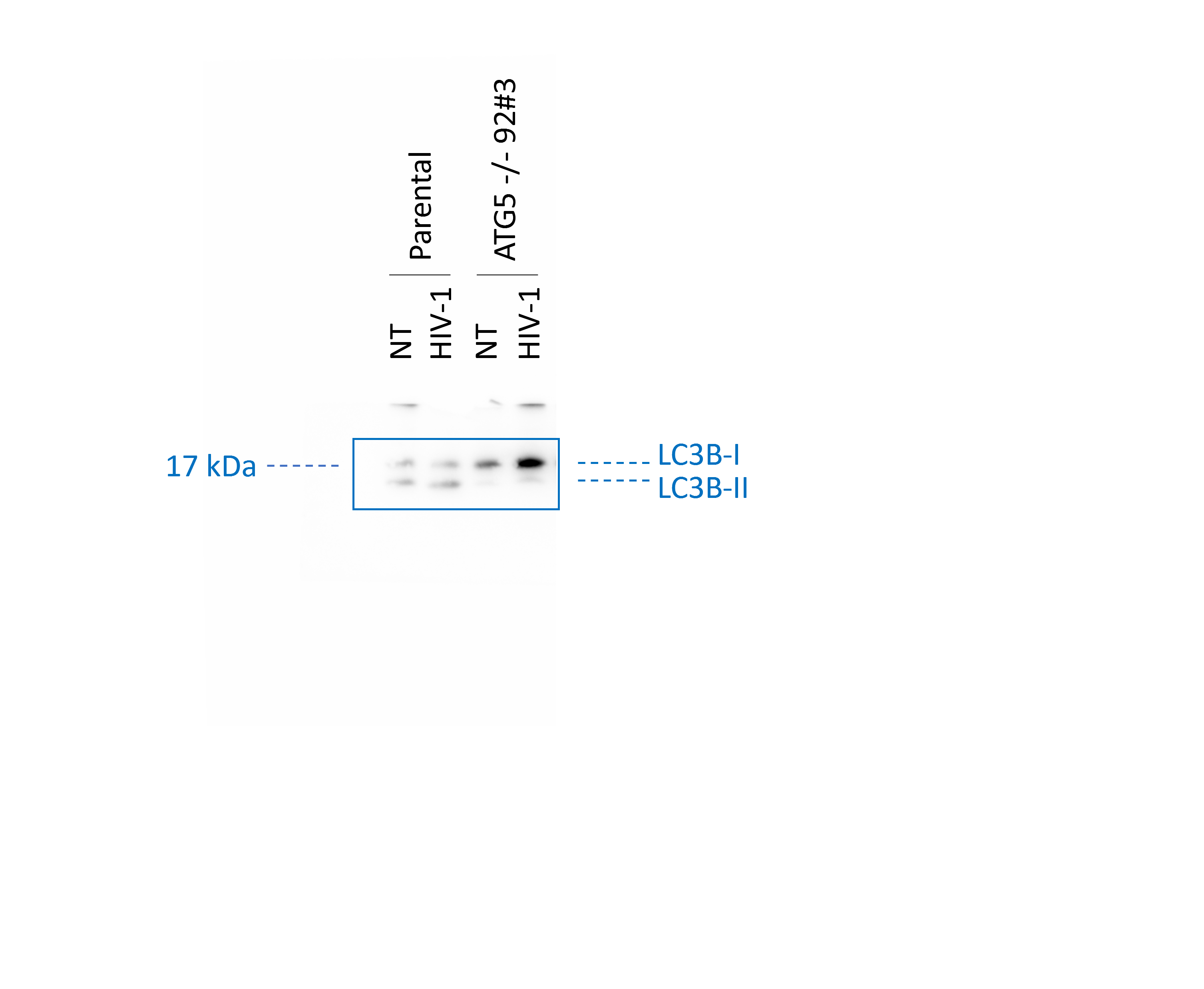

Supplement: Supplementary file 13 — Figure EV4 Source Data [file 44319_2025_607_MOESM13_ESM.zip › Figure EV4 B/figEV4 B_LC3B_cell.tif]

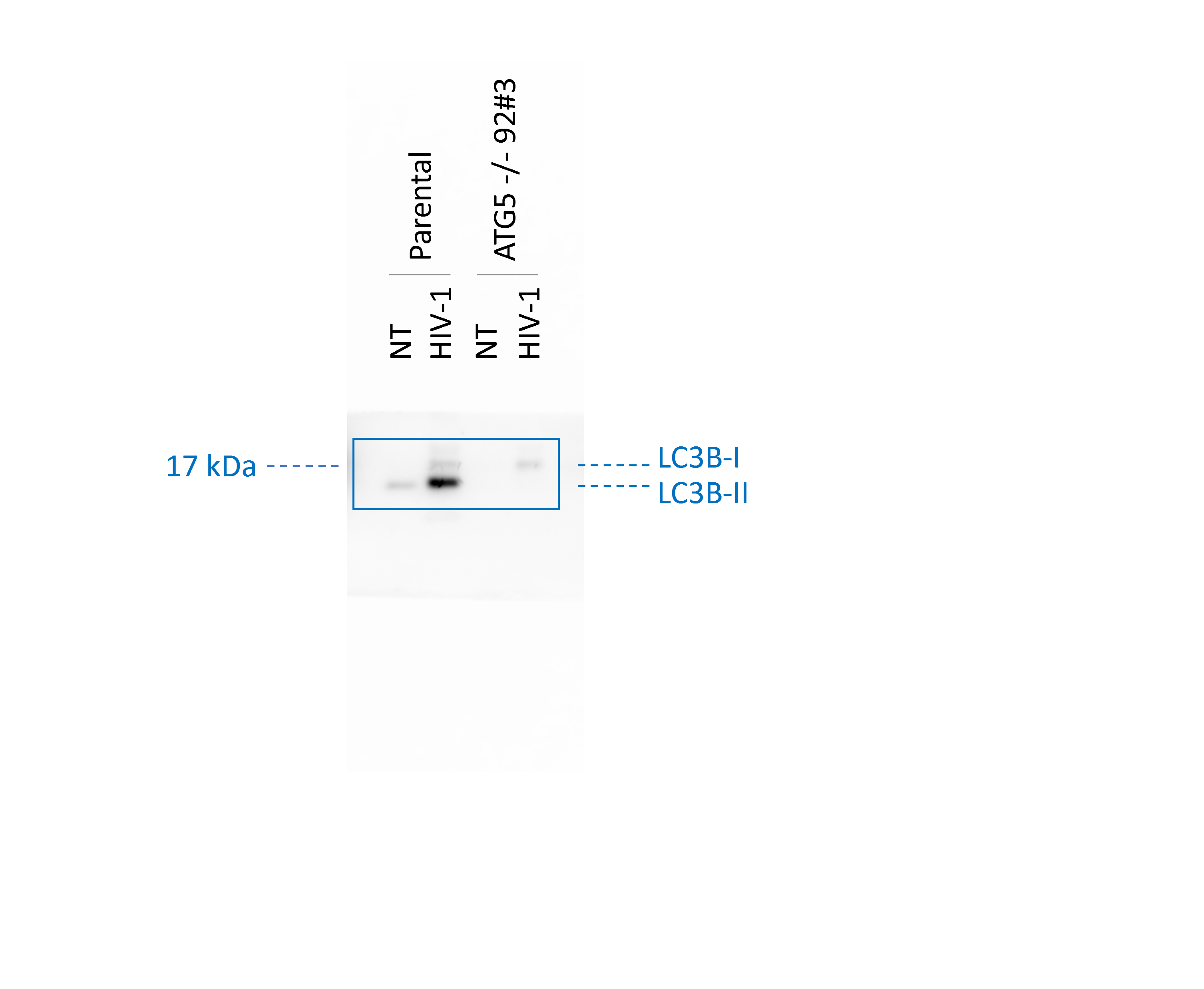

Supplement: Supplementary file 13 — Figure EV4 Source Data [file 44319_2025_607_MOESM13_ESM.zip › Figure EV4 B/figEV4 B_LC3B_virion prep.tif]

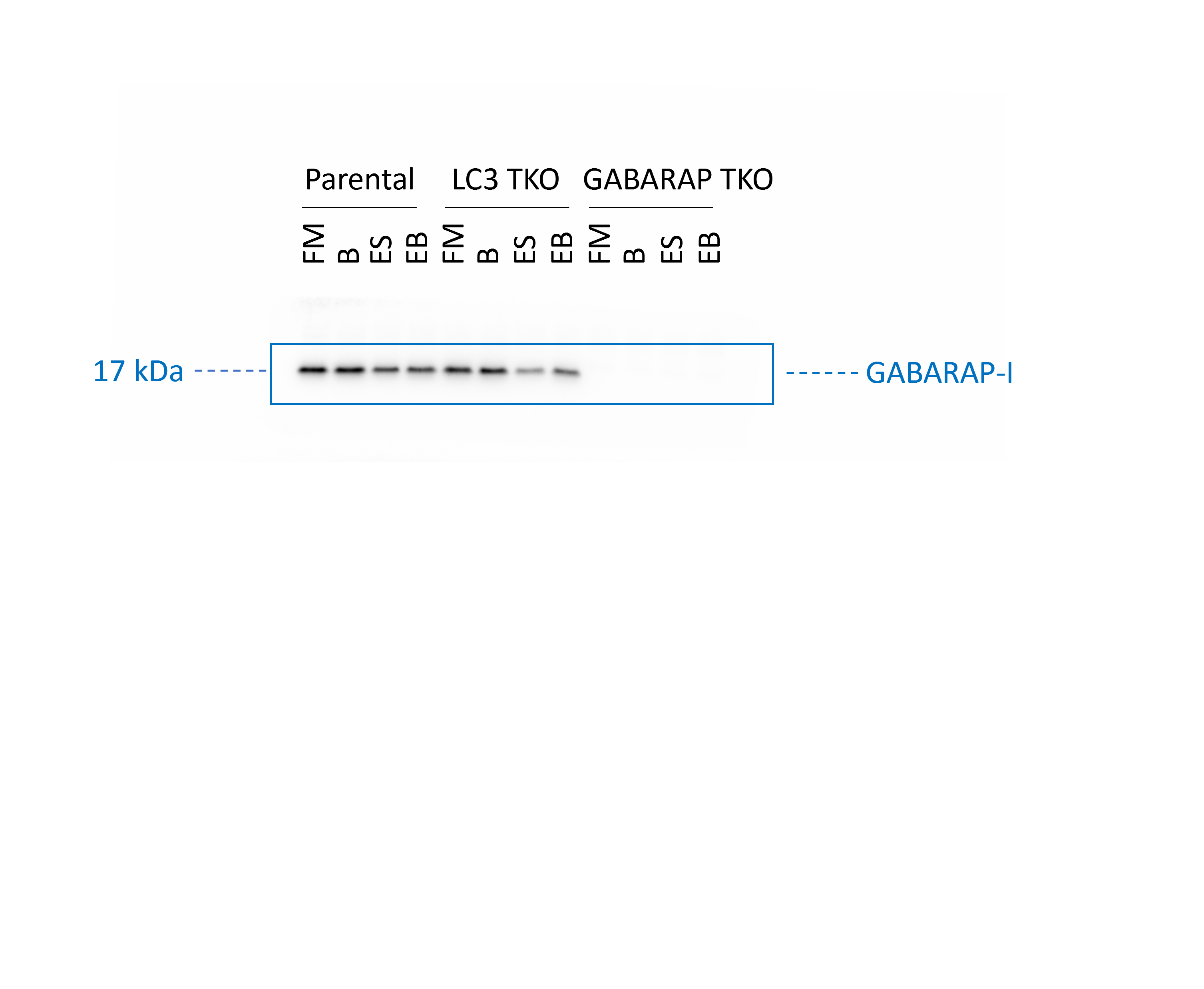

Supplement: Supplementary file 13 — Figure EV4 Source Data [file 44319_2025_607_MOESM13_ESM.zip › Figure EV4 A/figEV4 A_GABARAP.tif]

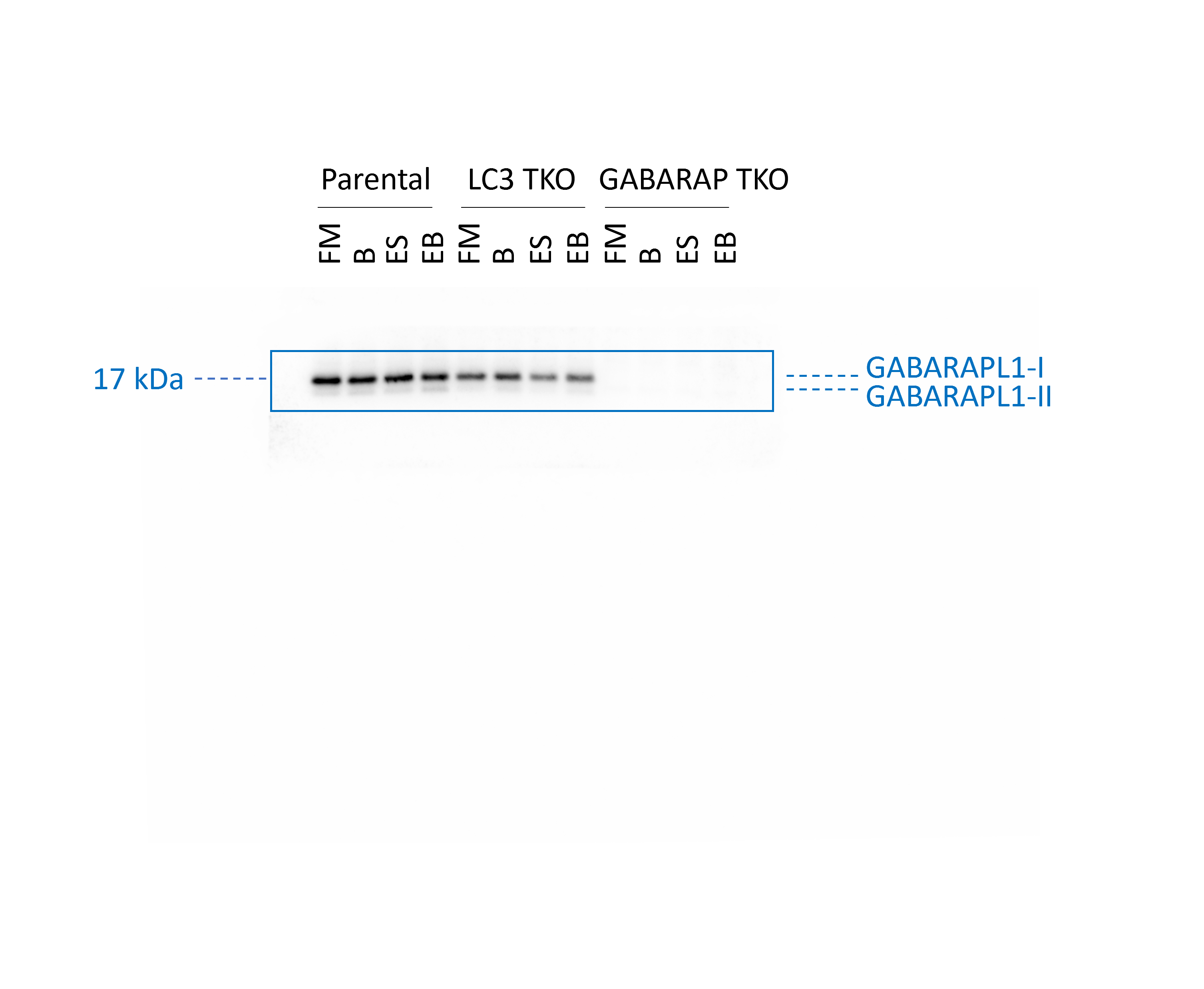

Supplement: Supplementary file 13 — Figure EV4 Source Data [file 44319_2025_607_MOESM13_ESM.zip › Figure EV4 A/figEV4 A_GABARAPL1.tif]

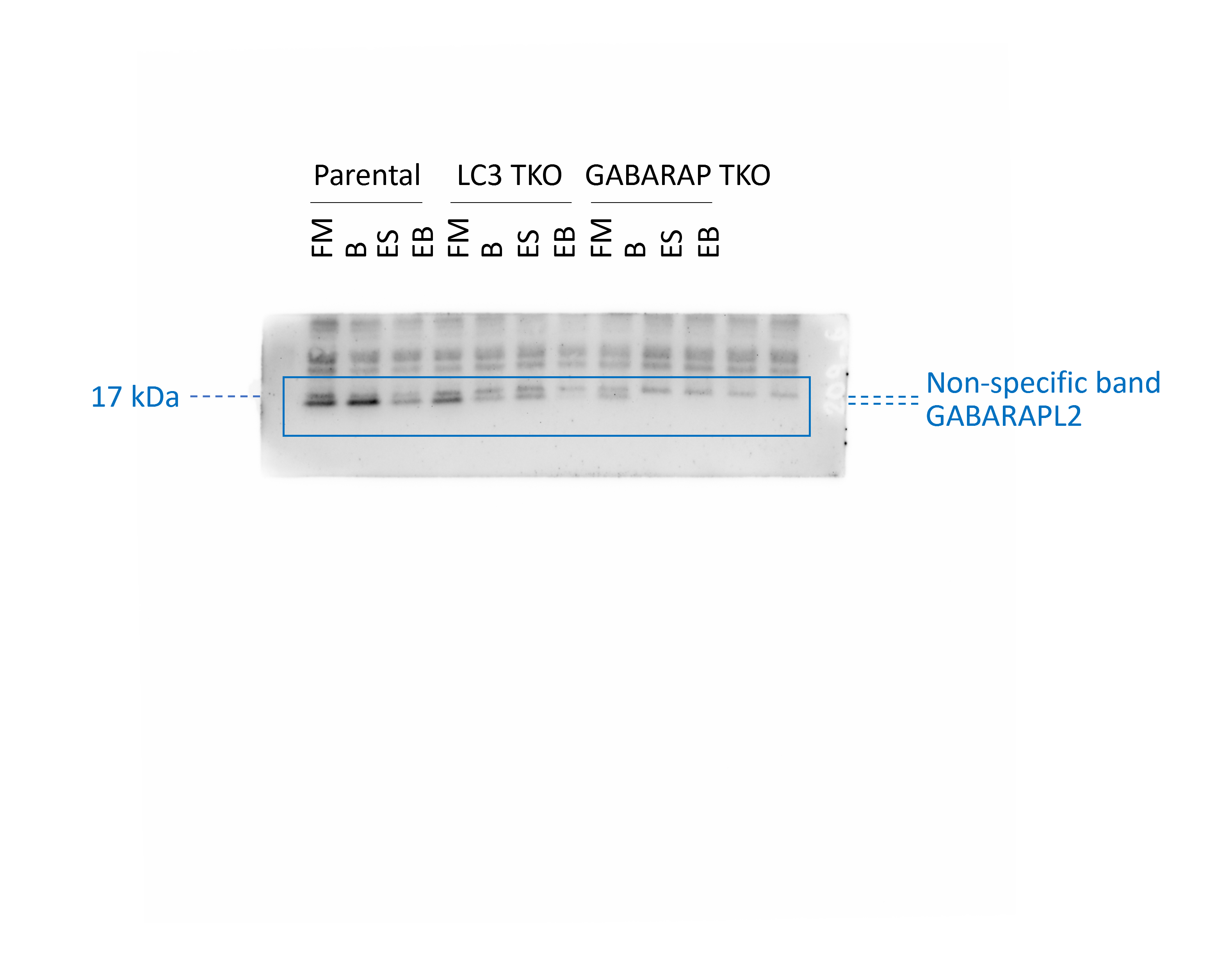

Supplement: Supplementary file 13 — Figure EV4 Source Data [file 44319_2025_607_MOESM13_ESM.zip › Figure EV4 A/figEV4 A_GABARAPL2.tif]

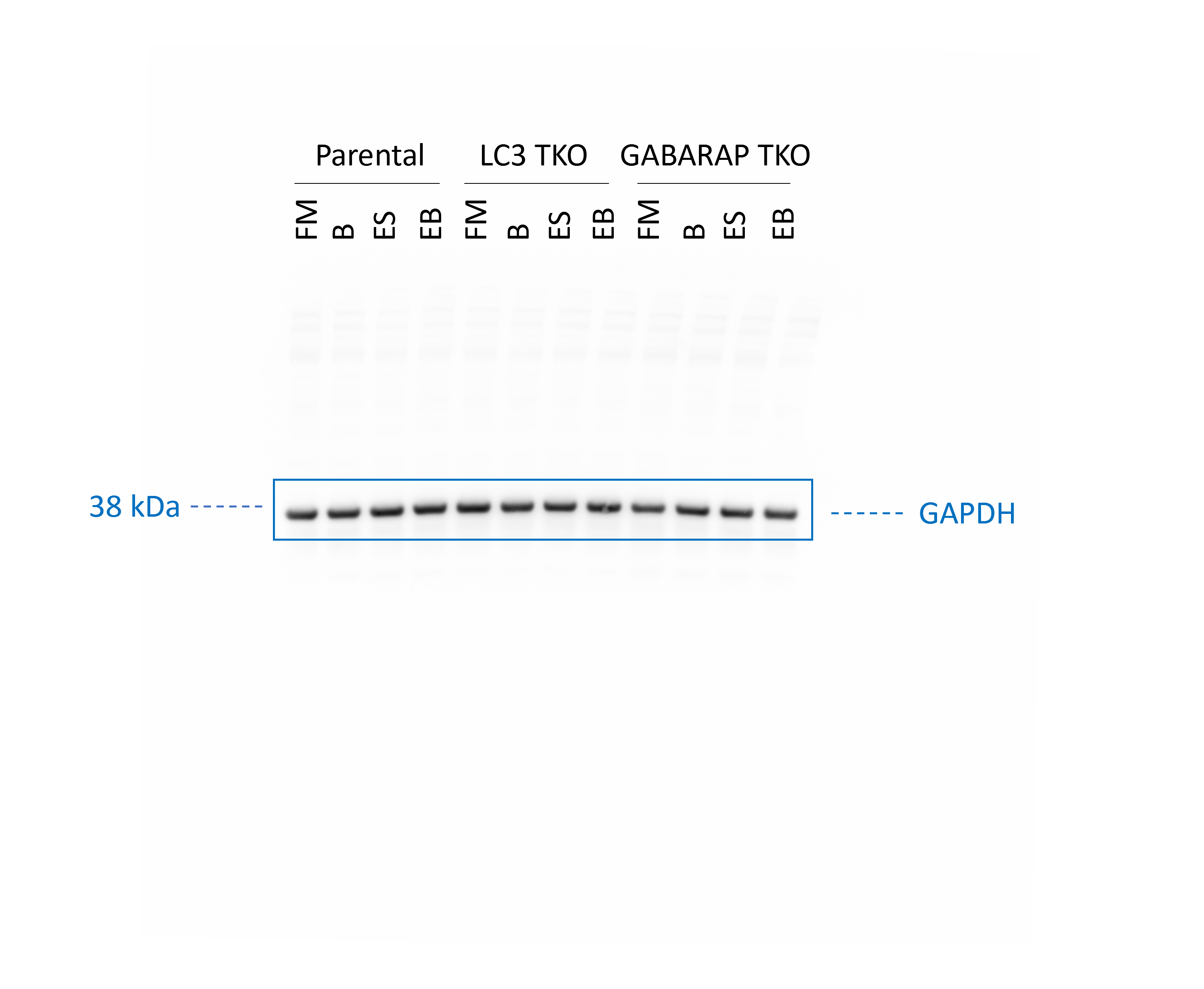

Supplement: Supplementary file 13 — Figure EV4 Source Data [file 44319_2025_607_MOESM13_ESM.zip › Figure EV4 A/figEV4 A_GAPDH.tif]

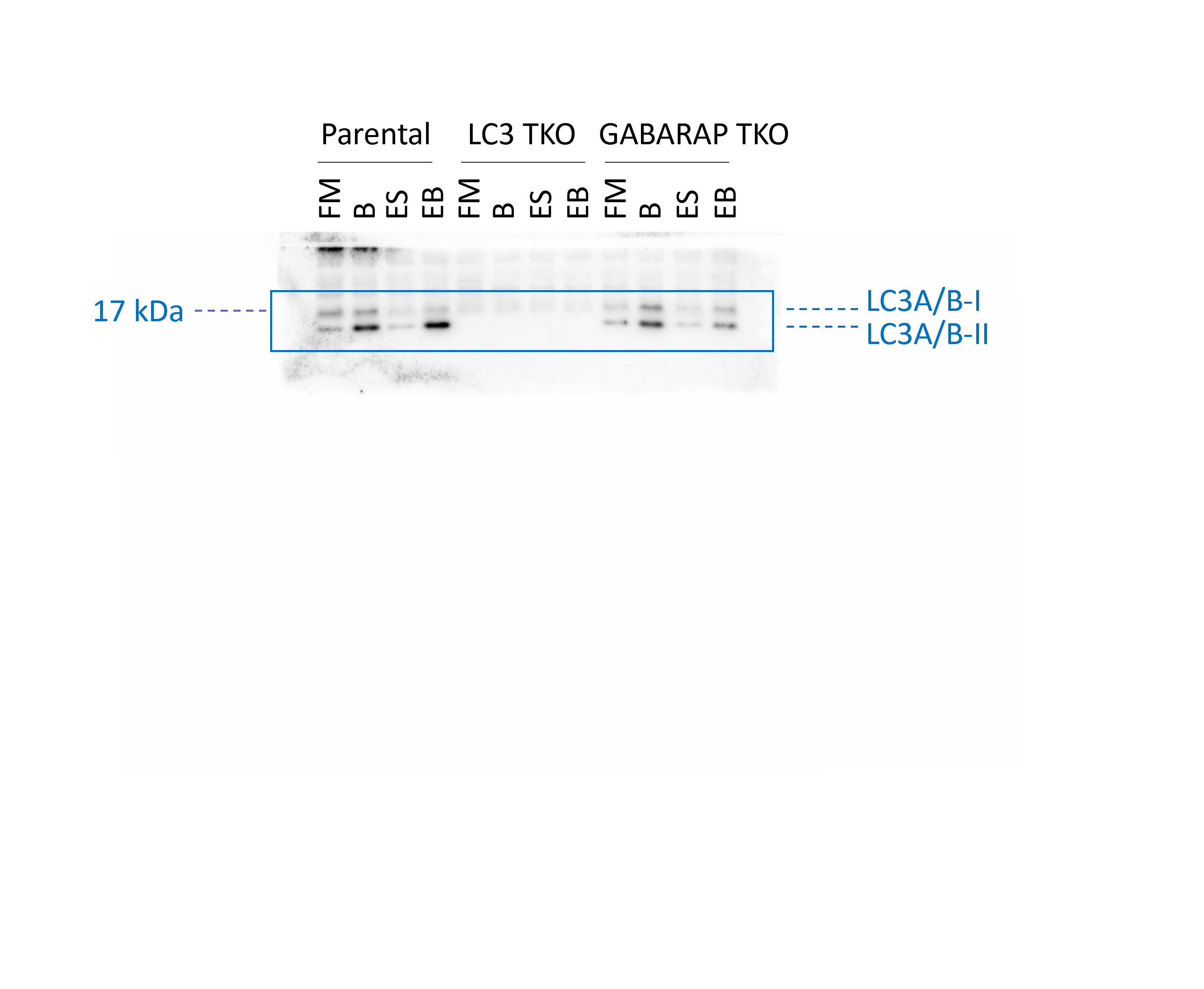

Supplement: Supplementary file 13 — Figure EV4 Source Data [file 44319_2025_607_MOESM13_ESM.zip › Figure EV4 A/figEV4 A_LC3A-B.tif]

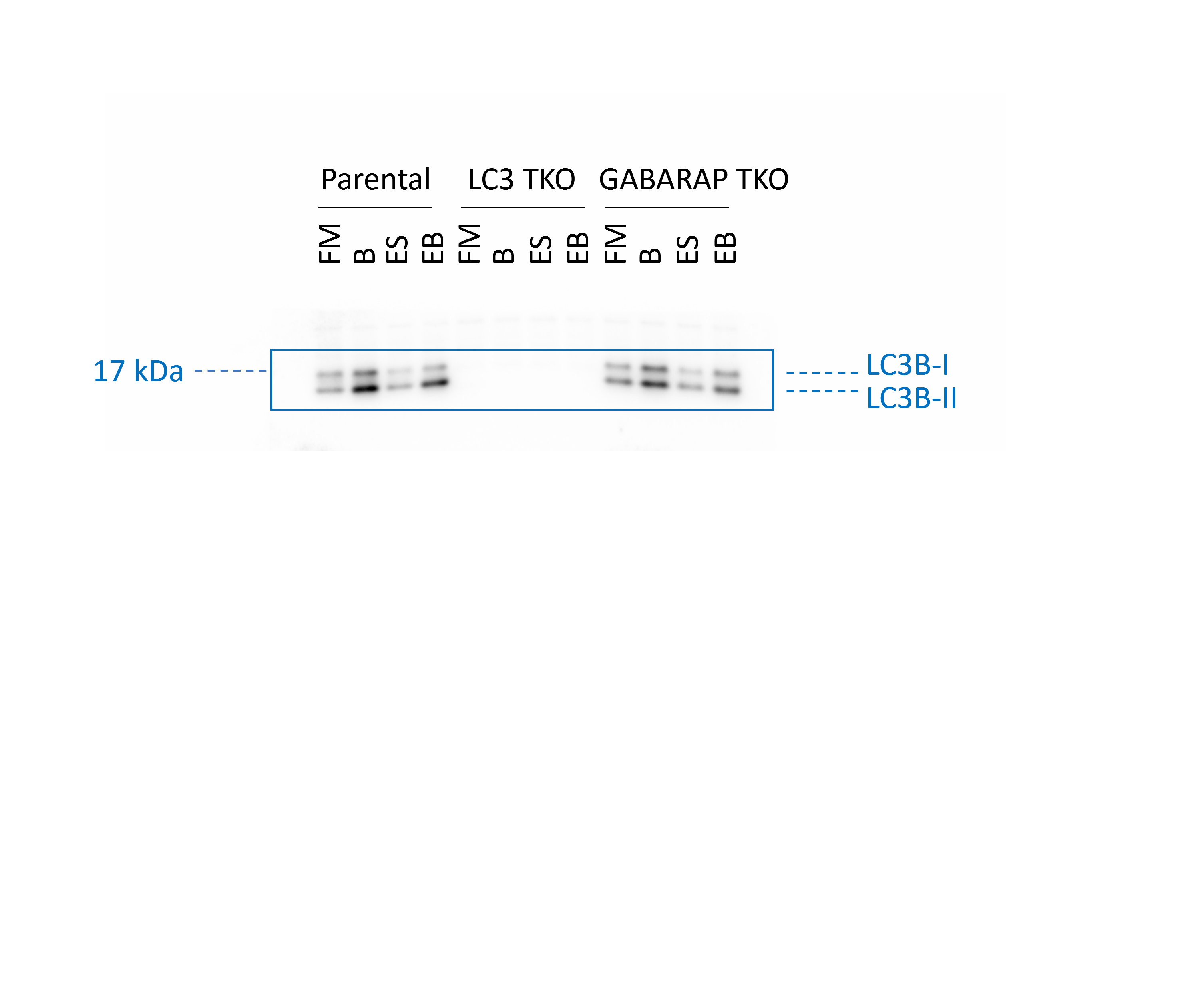

Supplement: Supplementary file 13 — Figure EV4 Source Data [file 44319_2025_607_MOESM13_ESM.zip › Figure EV4 A/figEV4 A_LC3B.tif]

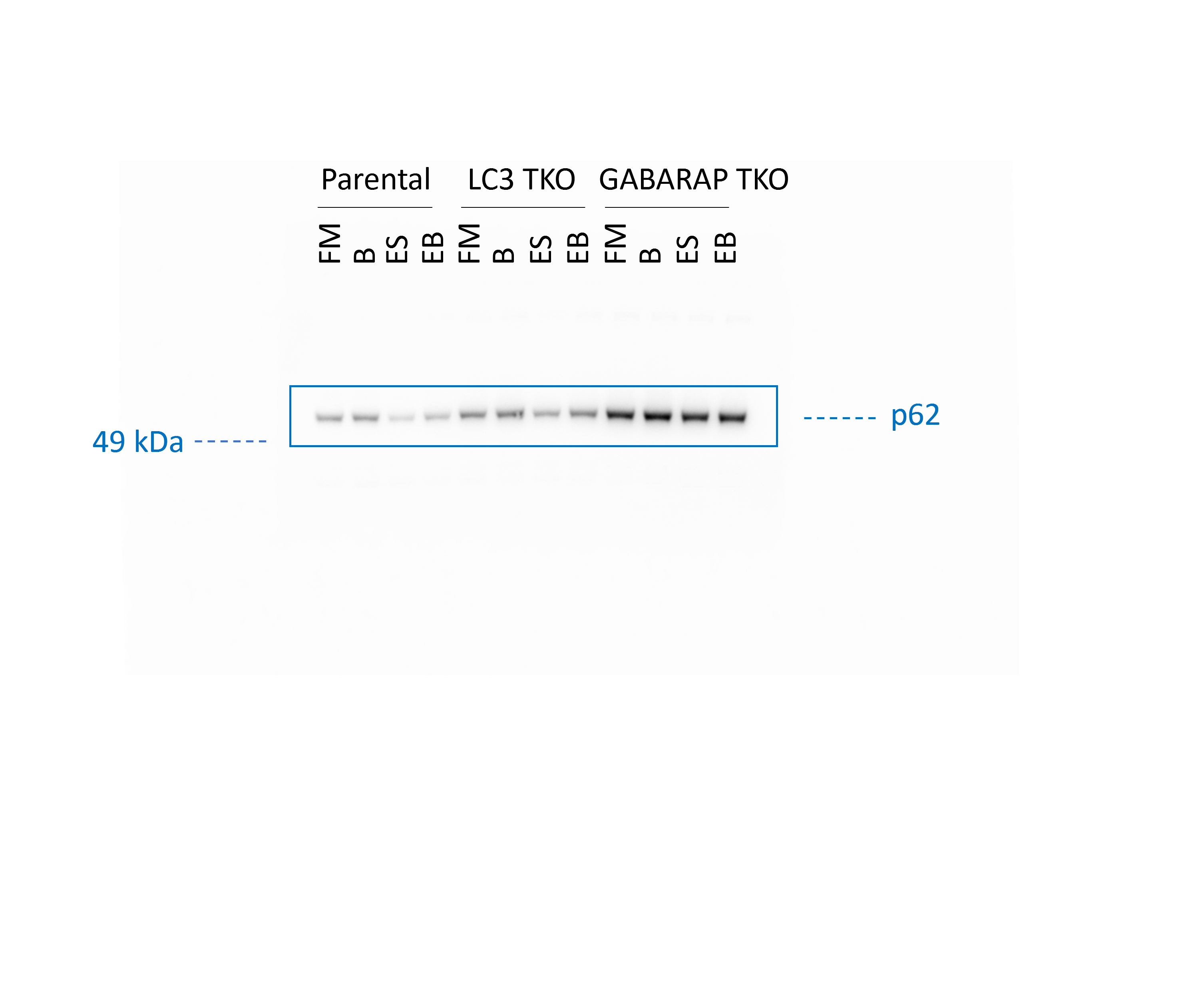

Supplement: Supplementary file 13 — Figure EV4 Source Data [file 44319_2025_607_MOESM13_ESM.zip › Figure EV4 A/figEV4 A_p62.tif]

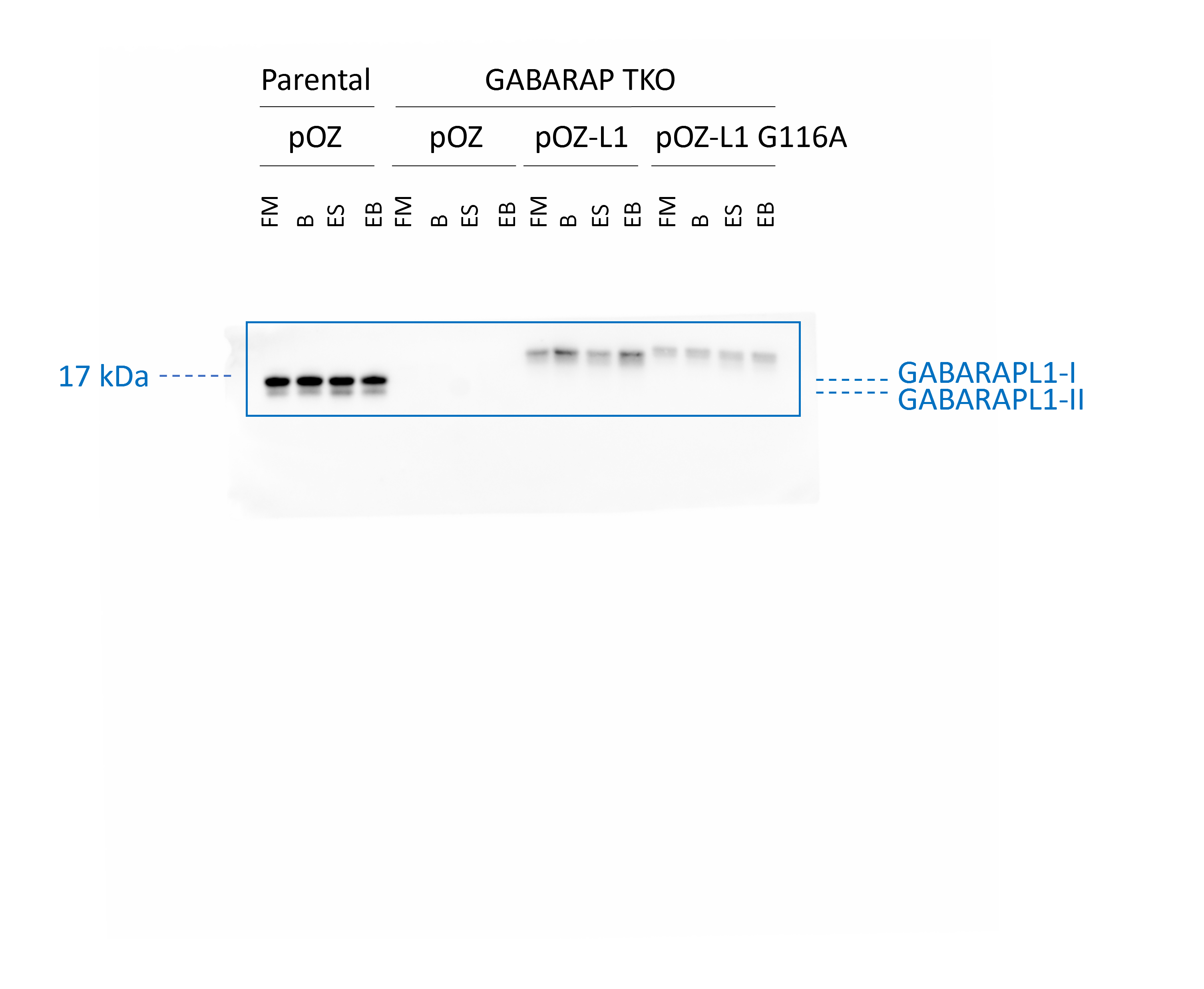

Supplement: Supplementary file 14 — Figure EV5 Source Data [file 44319_2025_607_MOESM14_ESM.zip › Figure EV5/figEV5_GABARAPL1.tif]

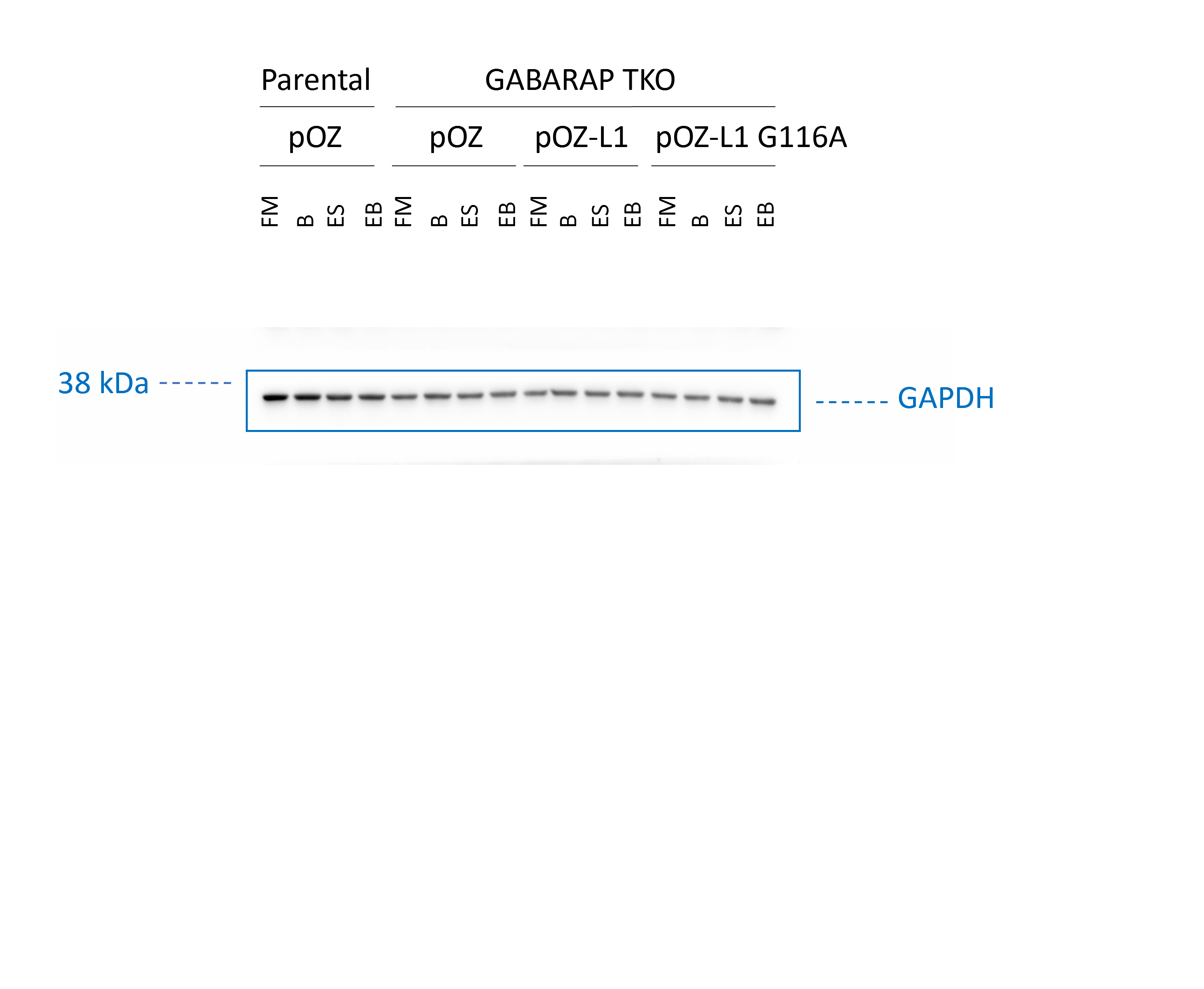

Supplement: Supplementary file 14 — Figure EV5 Source Data [file 44319_2025_607_MOESM14_ESM.zip › Figure EV5/figEV5_GAPDH.tif]

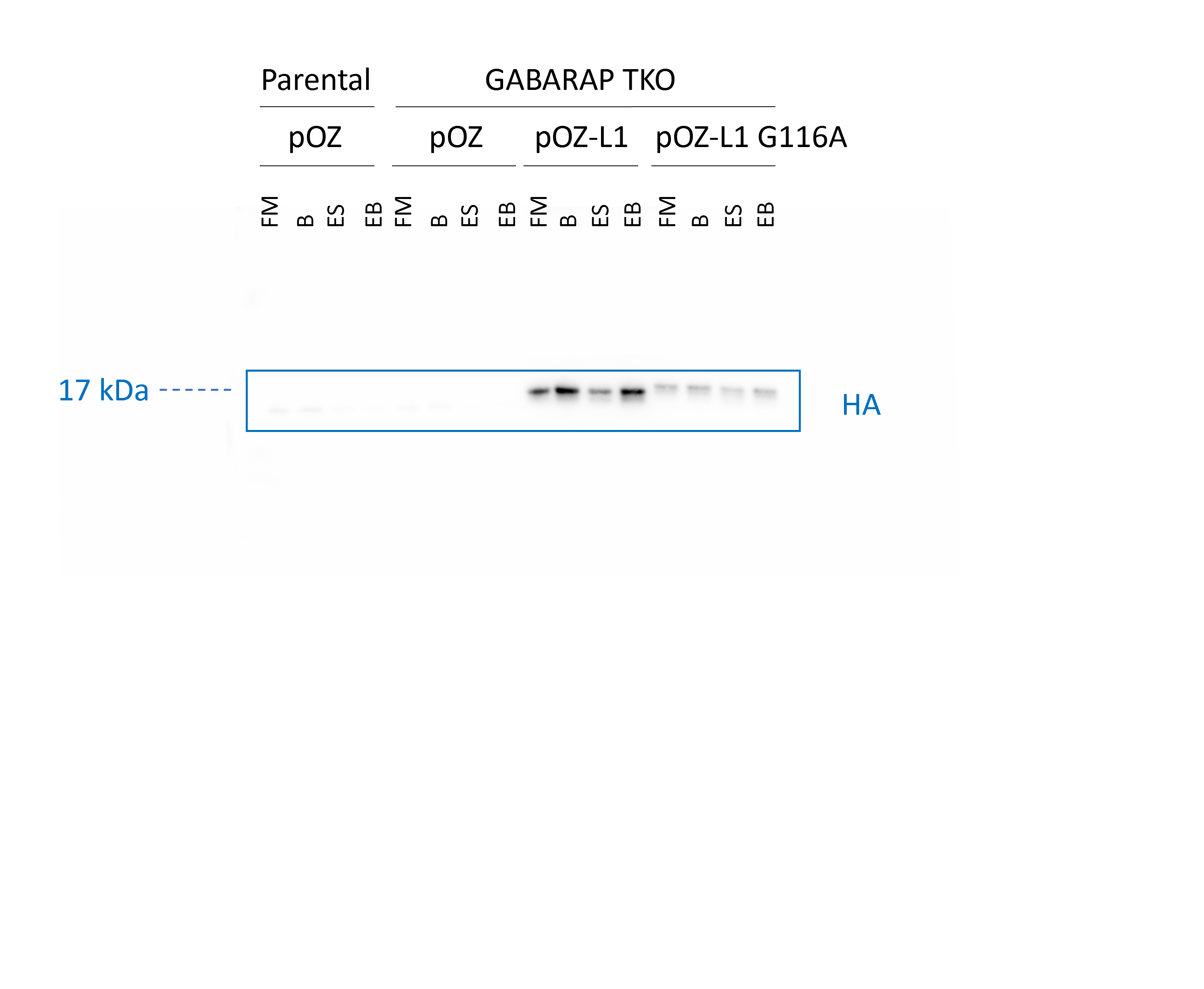

Supplement: Supplementary file 14 — Figure EV5 Source Data [file 44319_2025_607_MOESM14_ESM.zip › Figure EV5/figEV5_HA.tif]

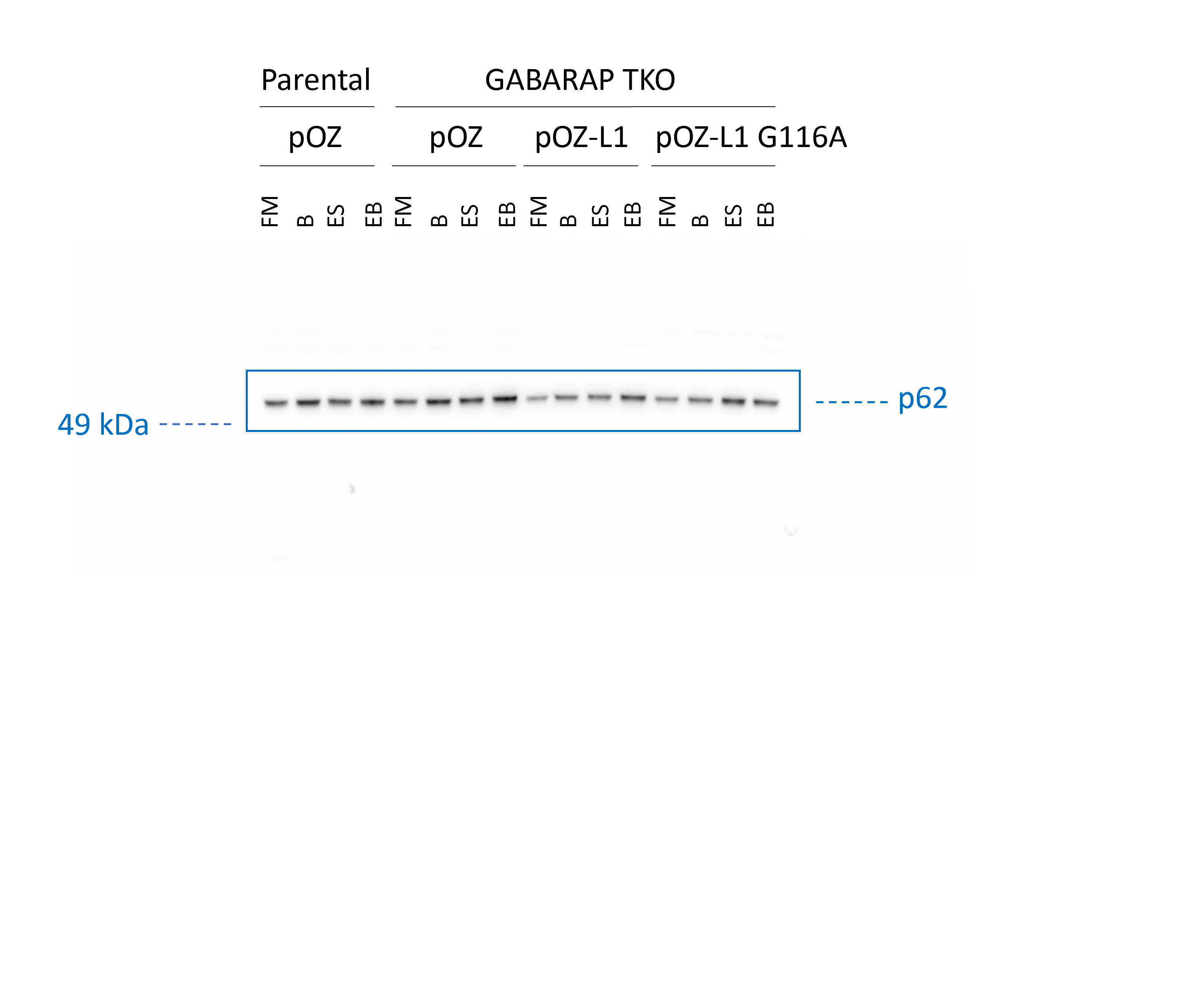

Supplement: Supplementary file 14 — Figure EV5 Source Data [file 44319_2025_607_MOESM14_ESM.zip › Figure EV5/figEV5_p62.tif]
