# Supplementary material for: GABARAP proteins regulate the packaging of HIV-1 genomic RNA into virions
Source: EMBO Rep. 2025 Oct 31;26(23):5826–58. doi: 10.1038/s44319-025-00607-1 (PMC12678799; doi:10.1038/s44319-025-00607-1)
Supplement: Supplementary file 15 — Expanded View Figures [file 44319_2025_607_MOESM15_ESM.pdf]

Expanded View Figures

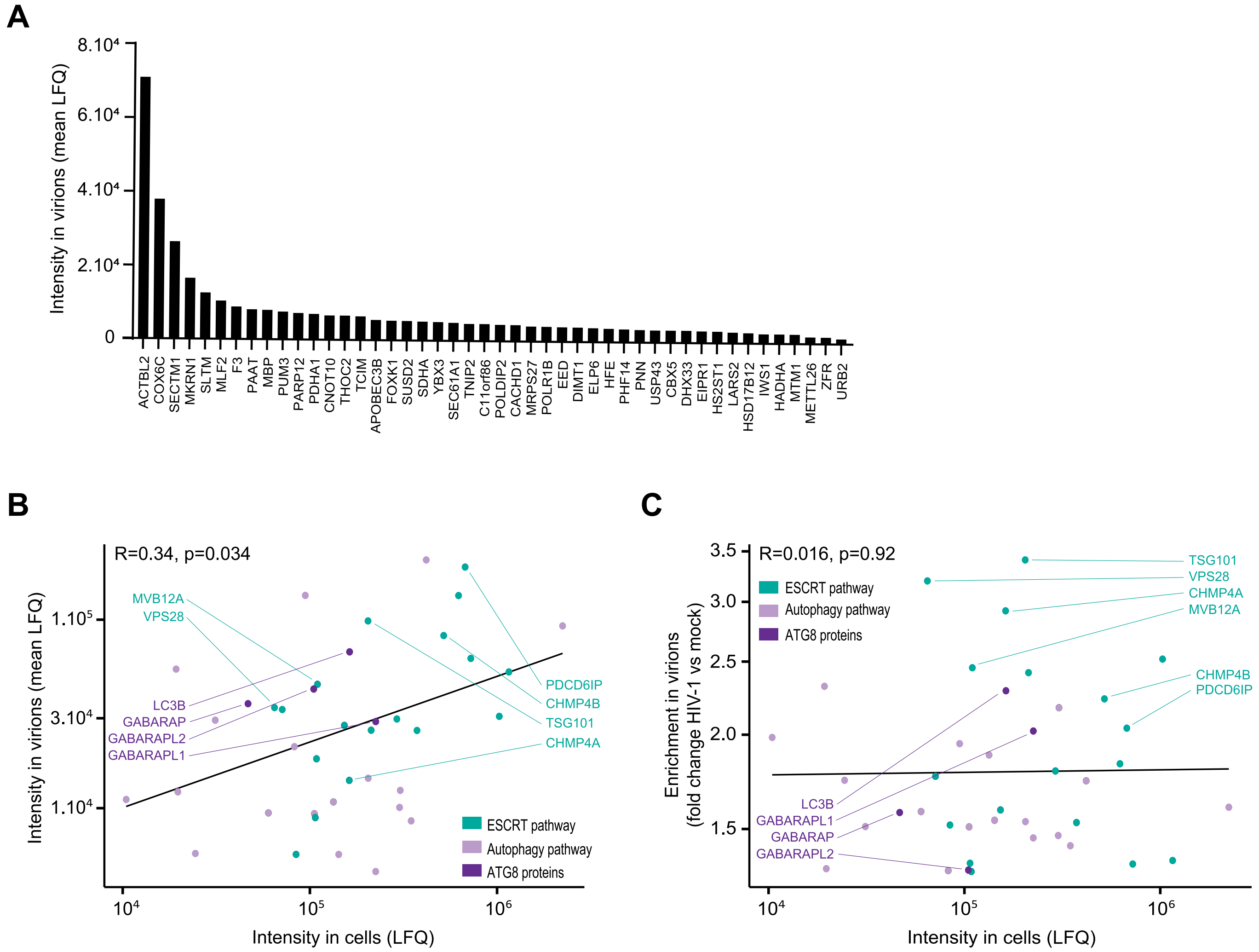

Figure EV1. Proteomic analysis of HIV-1 preparation and total cell lysate.

HeLa cells were transfected with WT NL4-3 HIV-1 proviral DNA or non-transfected as a control for 48 h. Supernatants were collected, filtered and ultracentrifuged on a 20% sucrose cushion. Virions-enriched preparations were subsequently lysed and analyzed by LC-MS. (**A**) LC-MS signal intensities (LFQ) of cellular proteins identified exclusively in four viral preparations from NL4-3 HIV-1 transfected HeLa cells ($n = 4$ biological replicates). (**B**) LC-MS signal intensities (LFQ) of viral preparation-associated ATG-related proteins from four experiments were plotted against signal intensities of these proteins in the total cell lysate from one replicate. (**C**) Enrichment of viral preparation-associated ATG-related proteins compared to control preparation from mock-transfected cells, from four biological replicates, plotted against signal intensities of these proteins in the total cell lysate from one replicate. Spearman correlations were calculated using the GG Scatter Package from the GG PubR library (version 0.6.0.999) in R. Data information: Statistical analysis was performed using a one-tailed paired Student *t*-test. Source data are available online for this figure.

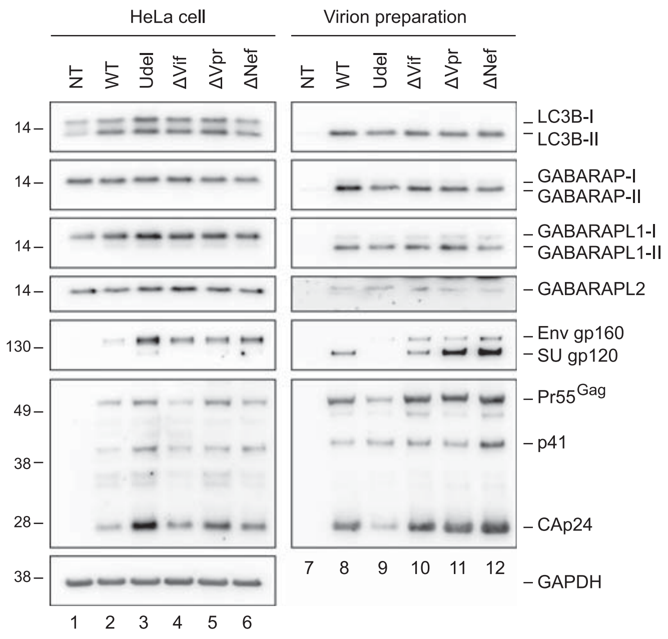

Figure EV2. Effect of the absence of HIV-1 accessory gene expression on the incorporation of LC3/GABARAP proteins into viral particles.

HeLa cells were transfected or not (NT) with WT, Udel, ΔVif, ΔVpr, and ΔNef NL4-3 HIV-1 proviral DNA for 48 h. Supernatants were collected and ultracentrifuged on a 20% sucrose cushion. Western blot analysis of LC3B, GABARAP, GABARAPL1, GABARAL2, HIV-1 SUgp120, HIV-1 Gag, and CAp24 products and GAPDH in producing cells and virion preparations. LC3B-I, GABARAP-I, and GABARAPL1-I correspond to the non-lipidated form of LC3B, GABARAP, and GABARAPL1, and LC3B-II, GABARAP-II, and GABARAPL1-II to the lipidated forms. Source data are available online for this figure.

 © The Author(s)

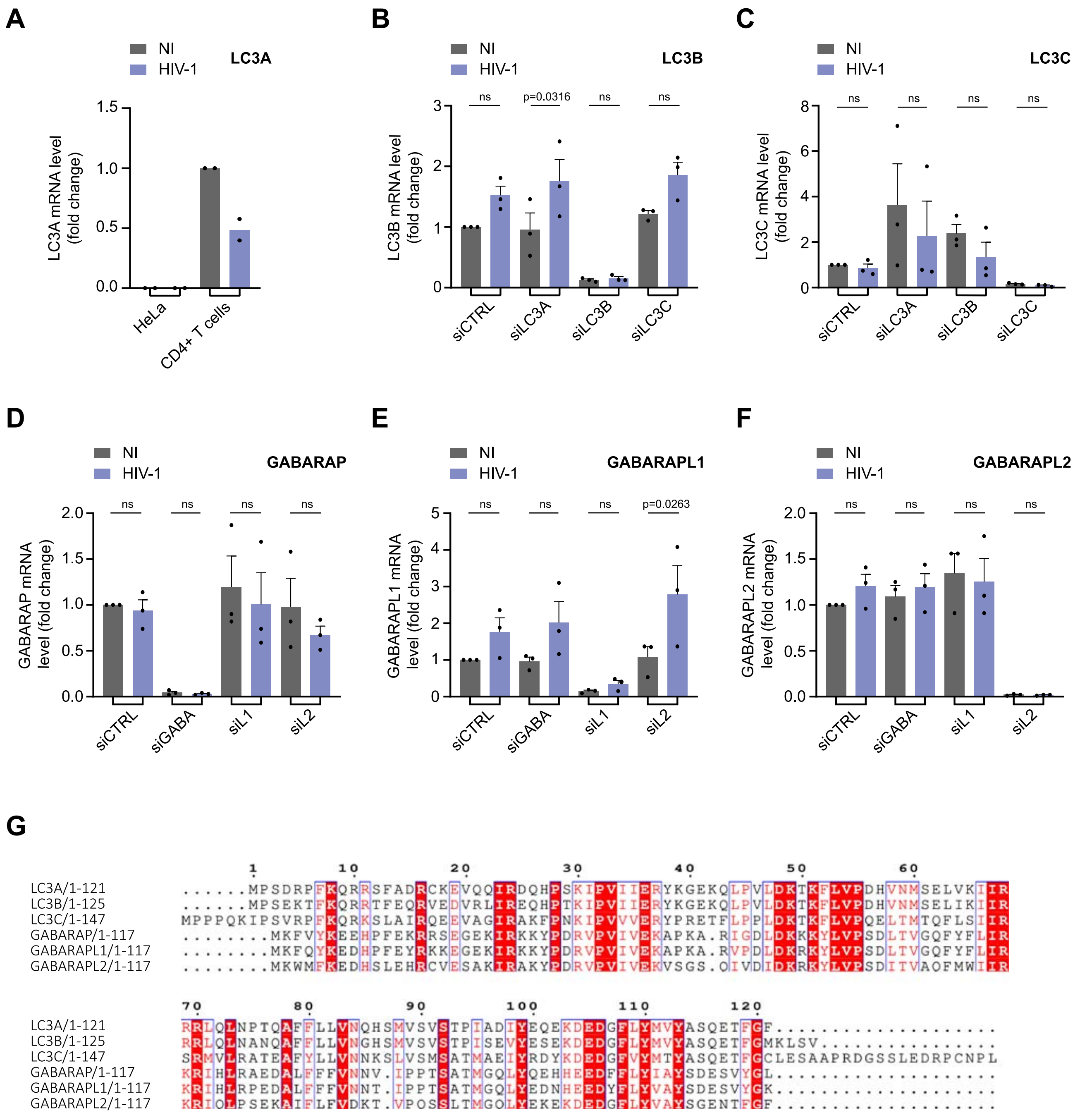

H

	LC3A	LC3B	LC3C	GABA	L1	L2
LC3A	100					
LC3B	90	100				
LC3C	75	70	100			
GABA	60	60	65	100		
L1	60	60	65	95	100	
L2	65	65	65	75	75	100

Figure EV3. Quantification of LC3/GABARAP mRNA expression in HIV-1-infected cells.

The mRNA quantity of LC3A was determined by RT-qPCR of total RNA extracted from HeLa cells or CD4 + T cells. HeLa cells or CD4 + T cells were infected or not (NI) with a VSVg pseudotyped WT NL4-3 HIV-1 at a MOI of 0.5 for 48 h. The mRNA quantity of LC3A was determined by RT-qPCR of total RNA extracted from cells. $n = 2$ biological replicates (**A**). HeLa cells transfected with control siRNA (siCTRL) or siRNA targeting LC3A, LC3B, LC3C, GABARAP, GABARAPL1, or GABARAPL2 were infected or not (NI) with a VSVg pseudotyped WT NL4-3 HIV-1 at a MOI of 0.5 for 48 h. The mRNA quantity of LC3B (**B**), LC3C (**C**), GABARAP (**D**), GABARAPL1 (**E**), or GABARAPL2 (**F**) was determined by RT-qPCR of total RNA extracted from cells. (**G**) Sequence alignment of LC3/GABARAP proteins using ESPript61. Identical residues are in red and similar ones are boxed. (**H**) Percentage of homology between LC3/GABARAP proteins. Data information: in (**B**–**F**), statistical analysis using a one-way ANOVA with Sidak's multiple comparisons test; mean ± SEM; $n = 3$ biological replicates; ns not significant. Source data are available online for this figure.

 © The Author(s)

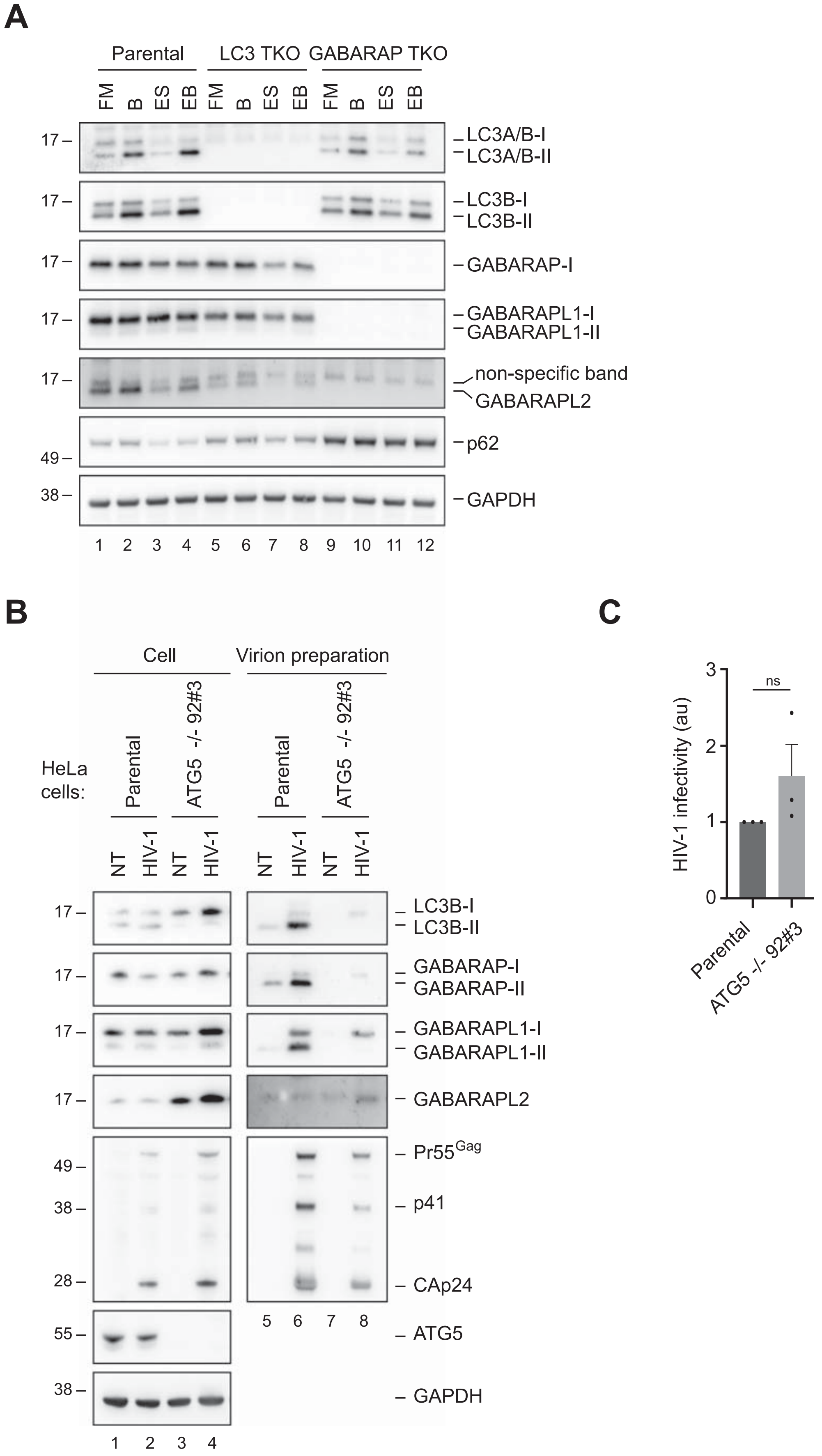
A
Parental
LC3 TKO
GABARAP TKO
FM B ES EB FM B ES EB FM B ES EB
17
LC3A/B-I
LC3A/B-II
17
LC3B-I
LC3B-II
17
GABARAP-I
17
GABARAPL1-I
GABARAPL1-II
17
non-specific band
GABARAPL2
p62
49
38
GAPDH
1 2 3 4 5 6 7 8 9 10 11 12
B
Cell
Virion preparation
HeLa cells:
Parental
ATG5 -/- 92#3
Parental
ATG5 -/- 92#3
NT HIV-1 NT HIV-1 NT HIV-1 NT HIV-1
17
LC3B-I
LC3B-II
17
GABARAP-I
GABARAP-II
17
GABARAPL1-I
GABARAPL1-II
17
GABARAPL2
Pr55Gag
49
38
p41
28
CAp24
5 6 7 8
55
ATG5
38
GAPDH
1 2 3 4
C
HIV-1 infectivity (au)
3
2
1
0
ns
Parental
ATG5 -/- 92#3

Figure EV4. Impact of LC3 and GABARAP knockout on autophagy flux in HeLa cells.

(**A**) Parental, LC3 (LC3 TKO), or GABARAP (GABARAP TKO) knock down cells were incubated in full medium (FM), full medium with Bafilomycin A1 (**B**), EBSS for amino acid depletion without or with Bafilomycin A1 (ES and EB, respectively) for 2 h. Western blot analysis of LC3A/B, LC3B, GABARAP, GABARAPL1, GABARAPL2, and GAPDH in cells. LC3A/B-I, GABARAP-I, and GABARAPL1-I correspond to the non-lipidated form of LC3A/B, GABARAP and GABARAPL1, and LC3A/B-II, GABARAP-II and GABARAPL1-II to the lipidated forms. All western blots are representative of at least three biological replicates. (**B**) Parental or ATG5−/− 92#3 knockout HeLa cells were transfected or not (NT) with WT NL4-3 HIV-1 proviral DNA for 48 h. Cell lysates were recovered, and supernatants were collected, filtered, and ultracentrifuged on a 20% sucrose cushion. Western blot analysis of LC3B, GABARAP, GABARAPL1, GABARAPL2, HIV-1 Gag and CAp24 products, ATG5 and GAPDH in producing cells and virion preparations. All western blots are representative of at least three biological replicates. (**C**) The infectivity of released virus was determined by a β-galactosidase reporter assay and normalized by the quantity of released CAp24. Data information: Statistical analysis using a one-tail unpaired Student's *t*-test; mean ± SEM; $n = 3$ biological replicates; ns not significant. Source data are available online for this figure.

© The Author(s)

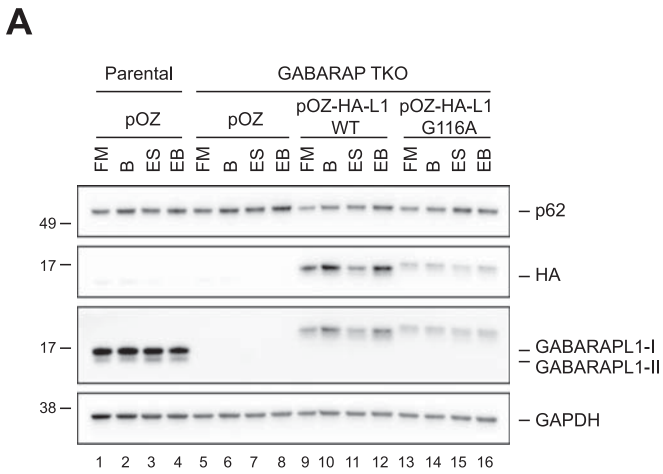

Figure EV5. Transcomplementation of GABARAP TKO cells with wild-type and non-lipidated mutant forms of GABARAPL1.

Parental, GABARAP (GABARAP TKO) knock down and HA-GABARAPL1 WT or G116A trans-complemented TKO cells were incubated in full medium (FM), full medium with Bafilomycin A1 (B), EBSS for amino acid depletion without or with Bafilomycin A1 (ES and EB, respectively) for 2 h. Western blot analysis of HA, GABARAPL1, p62, and GAPDH in cells. Source data are available online for this figure.
